# Supplementary figures and images for: The first juvenile dromaeosaurid (Dinosauria: Theropoda) from Arctic Alaska
Source: PLoS One. 2020 Jul 8;15(7):e0235078. doi: 10.1371/journal.pone.0235078 (PMC7343144; doi:10.1371/journal.pone.0235078)

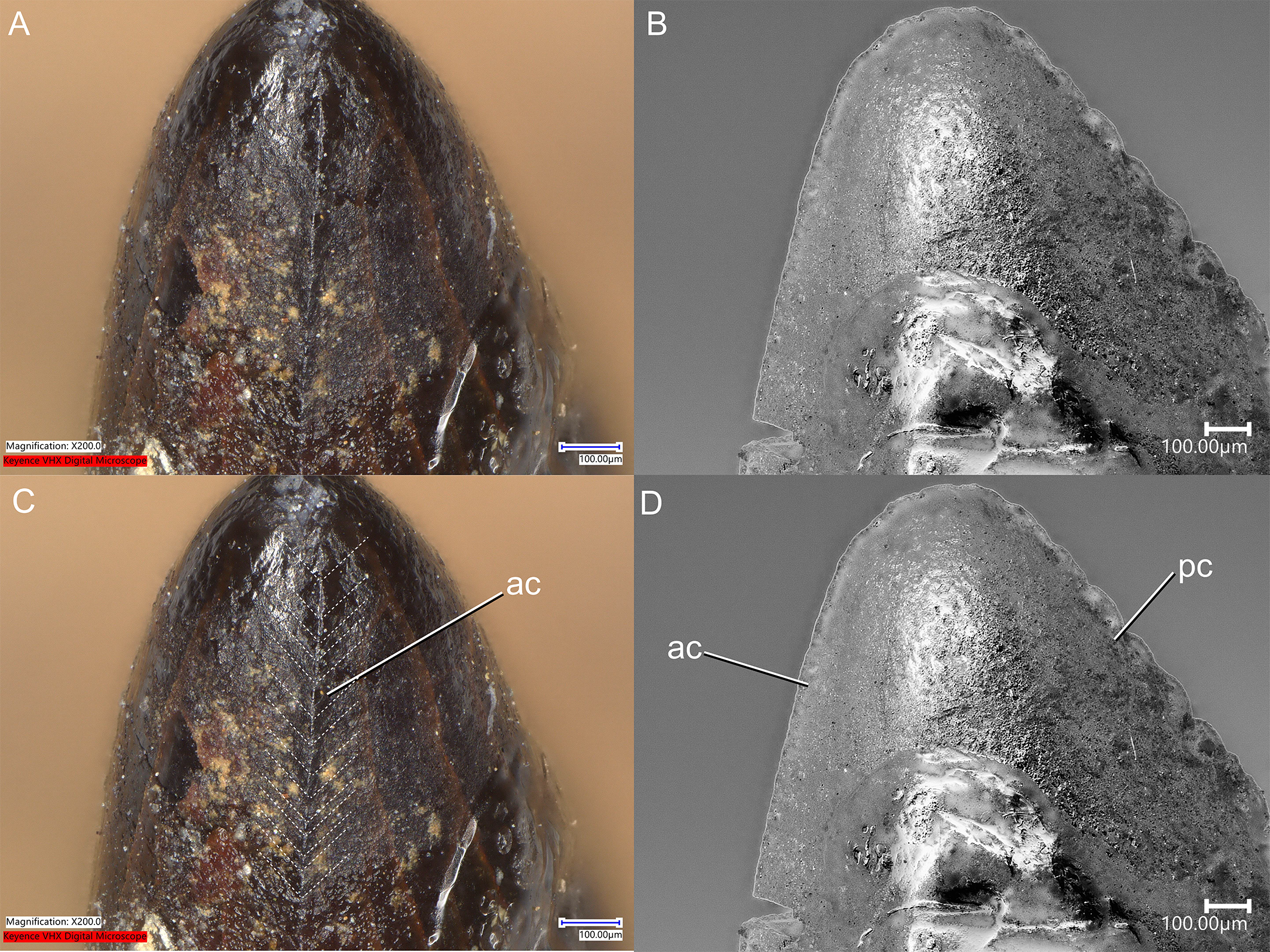

Supplement: S1 Fig — Close-up of the mesial carina in anterior view (A, C), highlighting the denticle-bearing anterior carina (ac). Magnified lingual view of the tooth (B) highlighting the anterior (ac) and posterior (pc) carinae (D). Dotted line (C) highlights the interdenticular sulci. Scale bar: 100 μm. (TIF) [file pone.0235078.s001.tif]

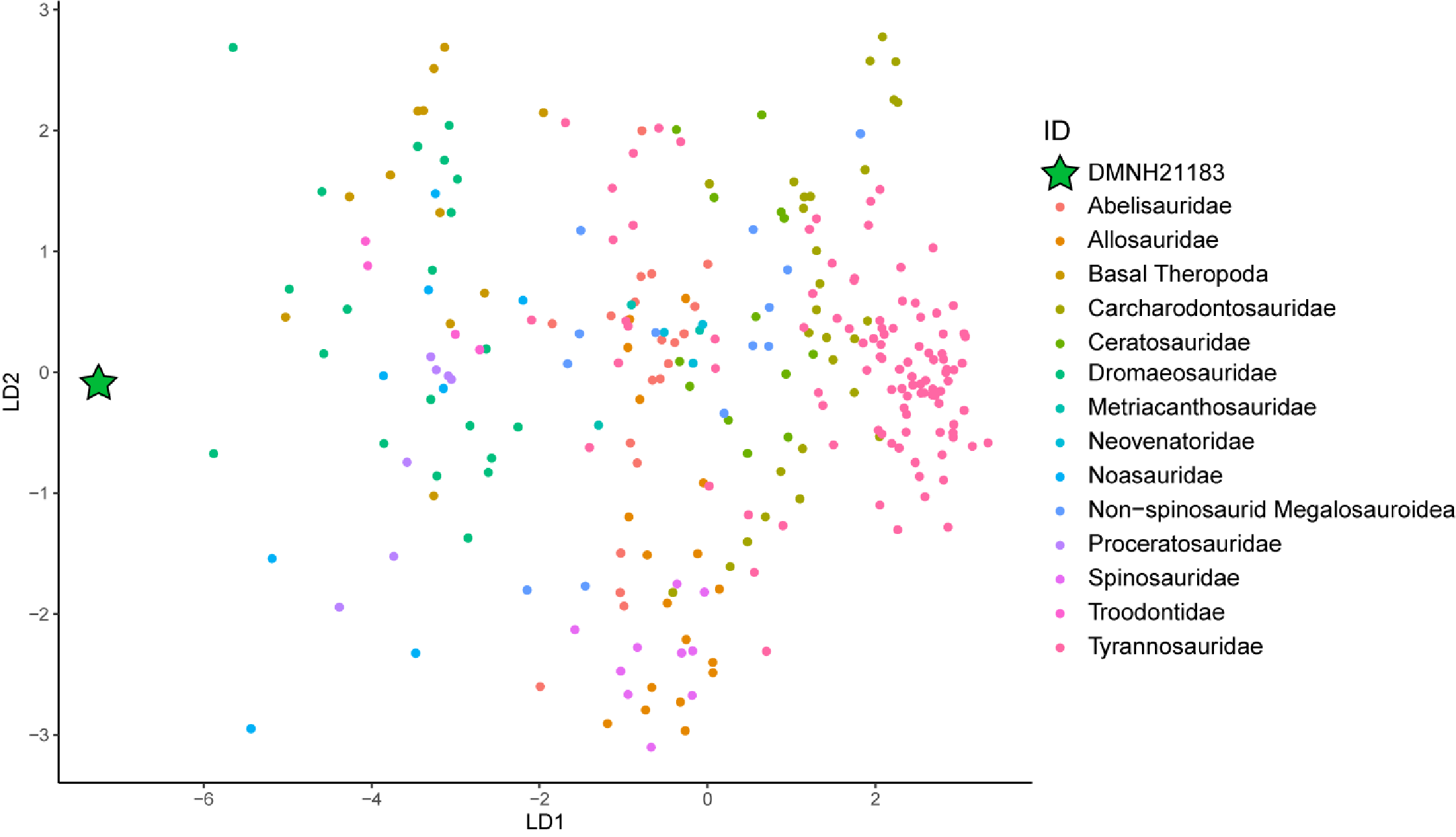

Supplement: S2 Fig — Discriminant Functional Analysis of DMNH 21183 in the theropod teeth morphospace generated with the morphometric dataset provided in Gerke and Wings [39]. Abbreviations: LD, linear dimension. DMNH 21183 indicated by a green star. (TIF) [file pone.0235078.s002.tif]

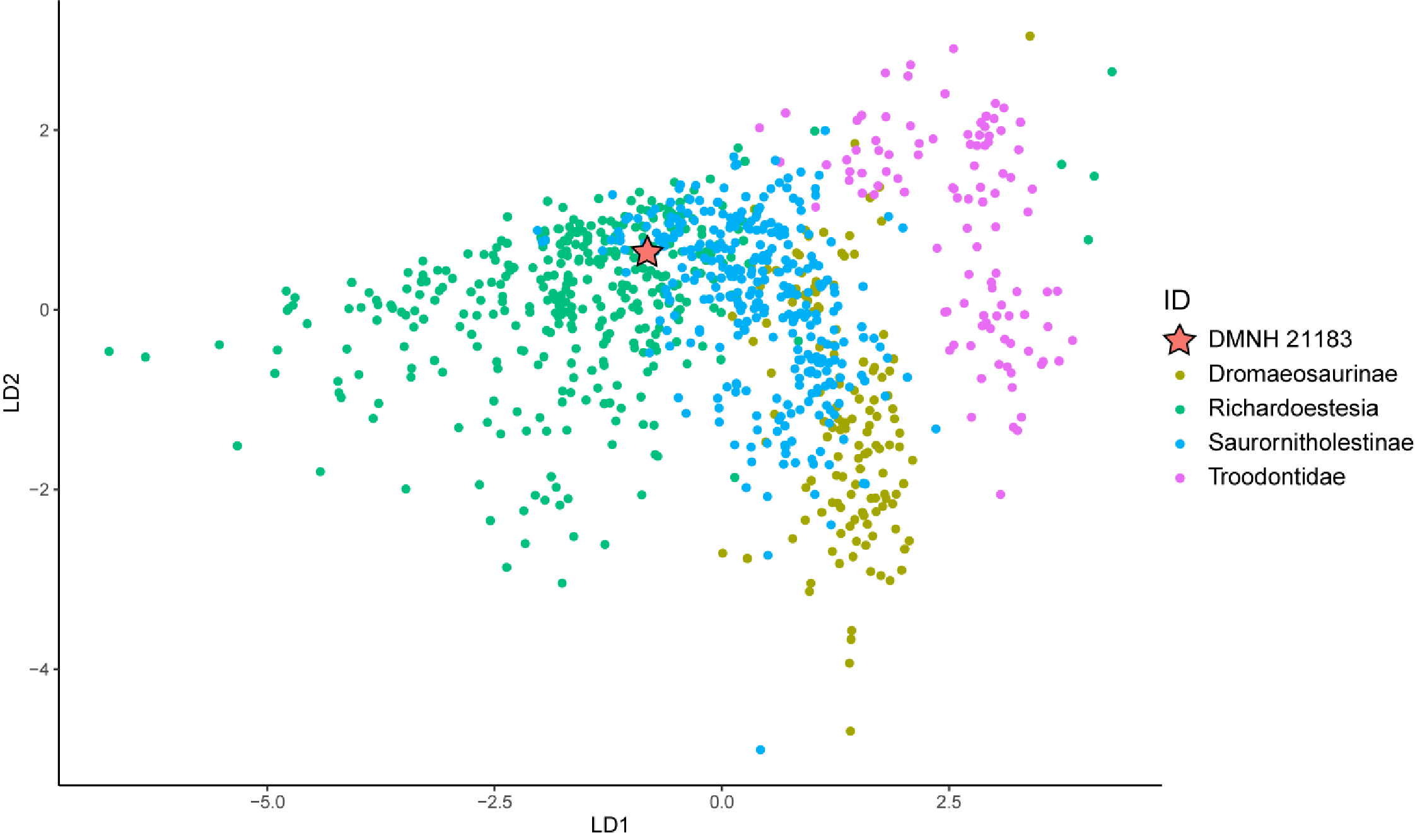

Supplement: S3 Fig — Discriminant Functional Analysis of DMNH 21183 in the deinonychosaurian teeth morphospace generated with the morphometric dataset provided in Larson and Currie [40]. Abbreviations: LD, linear dimension. DMNH 21183 indicated by a pink star. (TIF) [file pone.0235078.s003.tif]

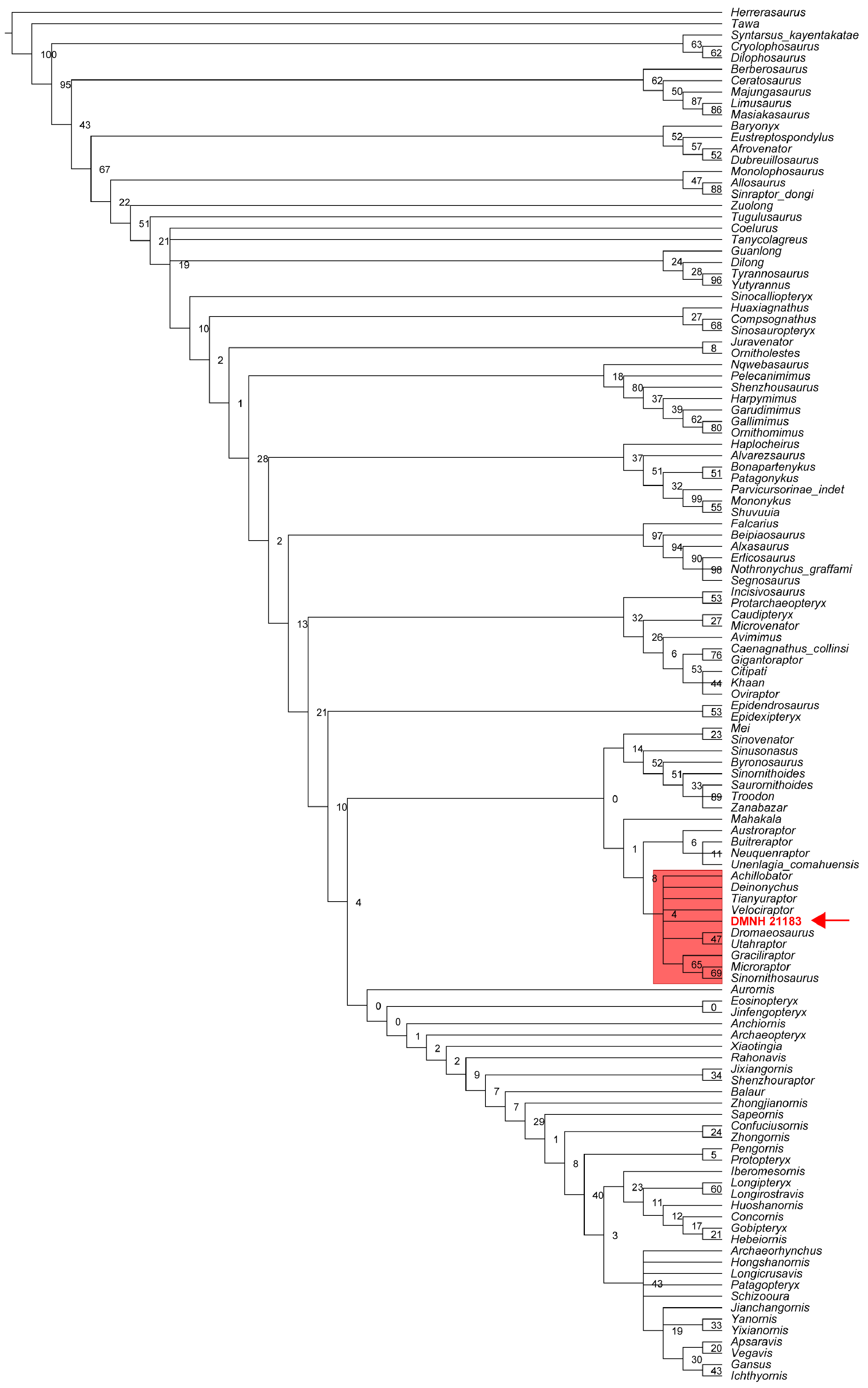

Supplement: S4 Fig — Strict consensus topology of the shortest trees recovered by the parsimony analyses showing the position of DMNH 21183 in the matrix from Lee et al. [33] (384 MPTs, 6043 steps, CI = 0.244, RI = 0.587). Numbers adjacent to nodes are the bootstrap values. Red box highlights the node containing DMNH 21183 (red arrow). (TIF) [file pone.0235078.s004.tif]

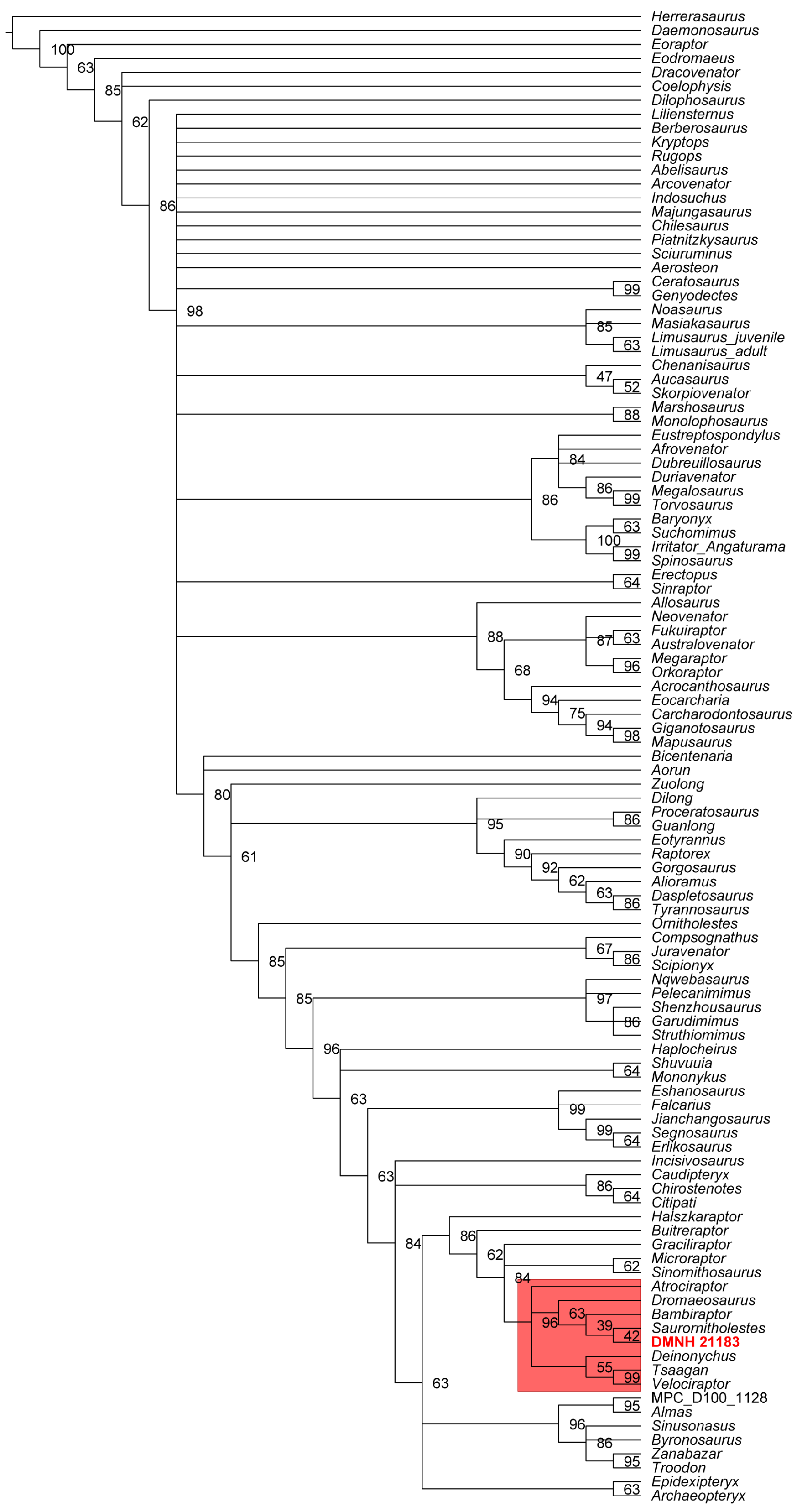

Supplement: S5 Fig — Strict consensus topology of the shortest trees recovered by the parsimony analyses showing the position of DMNH 21183 in the dentition-only character matrix from Hendrickx et al. [35] (2 MPTs, 1314 steps, CI = 0.194, RI = 0.418). The overall topology was constrained with DMNH 21183 allowed to float. Numbers adjacent to nodes are the bootstrap values. Red box highlights the node containing DMNH 21183 (red arrow) in Eudromaeosauria. (TIF) [file pone.0235078.s005.tif]

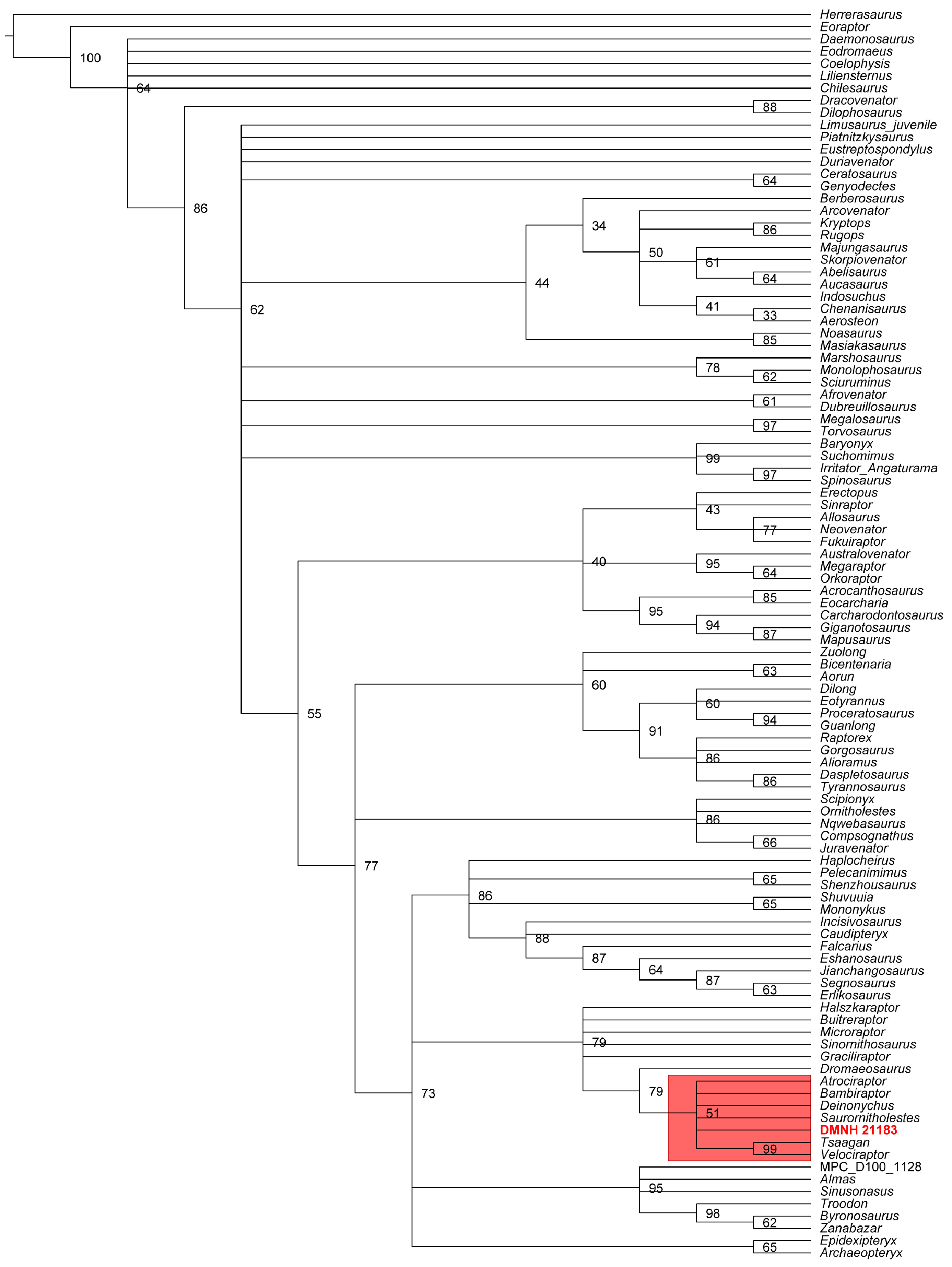

Supplement: S6 Fig — Strict consensus topology of the shortest trees recovered by the parsimony analyses showing the position of DMNH 21183 in the tooth-crown-only character matrix from Hendrickx et al. [35] (5 MPTs, 867 steps, CI = 0.183, RI = 0.439). The overall topology was constrained with DMNH 21183 allowed to float. Numbers adjacent to nodes are the bootstrap values. Red box highlights the more inclusive node containing DMNH 21183 (red arrow) in Eudromaeosauria. (TIF) [file pone.0235078.s006.tif]
