# Supplementary material for: The first juvenile dromaeosaurid (Dinosauria: Theropoda) from Arctic Alaska
Source: PLoS One. 2020 Jul 8;15(7):e0235078. doi: 10.1371/journal.pone.0235078 (PMC7343144; doi:10.1371/journal.pone.0235078)
Supplement: S1 Dataset — Character scoring in the phylogenetic matrices from Lee et al. [33] and Hendrickx et al. [35]. (RTF) [file pone.0235078.s008.rtf]

Supplementary Information
Data S1. Phylogenetic character scorings for DMNH 21183

Codings for DMNH 21183 in the Lee et al. 2014 data-matrix (originally sourced at https://datadryad.org/stash/dataset/doi:10.5061/dryad.jm6pj)

DMNH_21183              ???????????????????????????????????????????0????????????????????????????????????????????????????????????????????????????????????????????????????????????1?0?0?0??00?0????????????????????????????????????????????????????????????????????????????????????????????????????????????????????????????????????????????????????????????????????????????????????????????????????????????????????????????????????????????????????????????????????????????????????????????????????????????????????????????????????????????????????????????????????0?????????????????????????????????????????????????0??????????????????????????1?????????0?????????????????????????????????????????0???????????????????????????????????????????????????0????????????????0100?????????????????????????????????????????????????????0??????????????????????????????????????0???????????????????????????????????????????????????????????????????10???1??????????????????????????0??????????????????????????????????????????????????????????????????????????????????????????????????????????????????????????????????????????????????????????????????????????????????????????????????????????????0?????????????????????????????????????????????????????????????????????1???????????????????????????????????????????????0??????????????????????????????????????????????????????????????????????????????????????0???????????????????????????0??????????????????????????????????????????????0???????????????????????????????????????????????????????????????0???????????????????????????????????????0??????????????????????????????????????????

Codings for DMNH 21183 in the Hendrickx et al. 2020 Dentition-only matrix (originally sourced at https://doi.org/10.1016/j.cretres.2019.104312)

DMNH_21183            
??????????????????????????0?000?00?0?00?0210?31102?02001?(0 1)(1 2)2010000????????????????????????????????????????????????????????????????????????????????

Codings for DMNH 21183 in the Hendrickx et al. 2020 Crown-only matrix (originally sourced at https://doi.org/10.1016/j.cretres.2019.104312)

DMNH_21183	
0????1[0 1]?11[0 1]0??[0 1]2001?[0 1][1 2]20100000???????0?????????????????????????????????????????????????????


References

Lee MSY, Cau A, Naish D, Dyke GJ. Sustained miniaturization and anatomical innovation in the dinosaurian ancestors of birds. Science. 2014; 345(6196): 562-566.

Hendrickx C, Tschopp E, Ezcurra M. d. Taxonomic identification of isolated theropod teeth: the case of the shed tooth crown associated with Aerosteon (Theropoda: Megaraptora) and the dentition of Abelisauridae. Cretaceous Research. 2020; 108: 104312. https://doi.org/10.1016/j.cretres.2019.104312.
