# Supplementary material for: The first juvenile dromaeosaurid (Dinosauria: Theropoda) from Arctic Alaska
Source: PLoS One. 2020 Jul 8;15(7):e0235078. doi: 10.1371/journal.pone.0235078 (PMC7343144; doi:10.1371/journal.pone.0235078)
Supplement: Attachment — Submitted filename: review Hendrickx.pdf [file pone.0235078.s011.pdf]

REVIEW of "*The first juvenile dromaeosaurid (Dinosauria: Theropoda) from Arctic Alaska*"

In their manuscript entitled "*The first juvenile dromaeosaurid (Dinosauria: Theropoda) from Arctic Alaska*", Chiarenza and colleagues report, describe and identify the anterior portion of a dentary (DMNH 21183) from the Early Maastrichtian Prince Creek Formation of northern Alaska. The specimen is referred to a juvenile saurornitholestine dromaeosaurid based on comparative anatomy and a morphometric analysis using the best preserved tooth (d3), an identification I agree with. This paper is well-written, well-organized and most of the figures are very nice. The descriptive part is thorough and the introduction, geological settings and discussion section are sound and detailed. The methodology used to identify the material could certainly be improved though, and I have some problems with the way the text discussing the phylogenetic distribution of the material based on dental features is written. If I believe that this contribution only provides a limited amount of information for our knowledge on theropods/paravian paleogeography and palaeoecology, I would nonetheless suggest to accept Chiarenza et al.'s work with moderate revisions, urging the authors to take into consideration my main recommendations:

- My main problem with this paper lies in the author's method to identify the material. First, I wonder why they did not perform a cladistic analysis using the most recent datamatrix on coelurosaurs/paravian relationships. I understand that their material is very limited but wonder if it does not display enough apomorphic features to help resolve its phylogenetic affinity. At least, the authors should specify why they do not want to conduct a cladistic analysis to explore the phylogenetic distribution of DMNH 21183. Second, they decide to perform a PCA based on crown-measurements of d3 to help in the identification of the material. Yet, I believe there are more appropriate methods to identify theropod teeth such as discriminant and cluster analyses using a dataset of crown-based measurements, or (even better according to me) a cladistic analysis using a dentition-based datamatrix and a fully constrained topological tree (see my paper on the *Aerosteon* tooth and the dentition of Abelisauridae recently published in *Cretaceous Research* on how to perform those analyses). That being said, the major problem I see is that the best preserved tooth of this small portion of mandible, d3, is a partially erupted tooth and I wonder how the authors managed to accurately take measurements of FABL, CH and BW on this crown. In addition, it does not make much sense to me to use a dataset restricted to paravians and to omit specimens from which the mesial carina does not include denticles. I would therefore recommend the authors to perform a first analysis with all taxa and measurements from Larson & Currie's dataset, then use a more restrictive one based on relevant arguments on why tyrannosauroids, for instance, should be excluded. I would also strongly advise them to perform discriminant and cluster analyses on the dataset of crown-based measurements and, ideally, a cladistic analysis using my most recent dentition-based datamatrix (from the *Aerosteon* paper), coding their dental material as coming from

the dentary and the mesial dentition. I am quite confident that the result of the cladistic analysis would further support a saurornitholestine affinity.

- In the methodology section, the authors should specify the result(s) of the cladistic analysis of what publications they follow when commenting on paravian phylogeny and the phylogenetic position of the different dromaeosaurids. Likewise, they should add the fact that it is the third dentary tooth of DMNH 21183 that is included in the PCA.

- In the chapter on morphological remarks (which I would suggest to rename "Phylogenetic position of DMNH 21183"), in the section in which the authors discuss the distribution of dental features displayed by DMNH 21183, they obviously use my recent paper on the distribution of dental features in theropods (*Palaeontologica Electronica*) as a main reference. I thank them for that but, if my paper is often cited, it frustrates me to see that some parts of the text directly comes from my paper without citing it. In many parts of the text, the authors just copied and pasted portions of my PE publication, using the exact same citations and specimen numbers, while I highly doubt that they have personally examined the material I have, or looked at the references the way I have. For fairness (and because sometimes it almost looks like plagiarism), I would really like the authors to rephrase the text that directly comes from my paper using the later as the main bibliographical source, and remove all specimens (those coming directly from my paper) that they did not examine themselves. In this section of the MS, the DSDI for crowns having mesial and distal roughly of the same size is also close to 1 and not lower than 0.9.

- I don't have any comments to make regarding the Introduction, Geological setting and Depositional Environments, and Discussion sections, which really seem to be sound and well-written. I would only recommend to move the part of the text that discuss the juvenile features displayed by the specimen, present in the description section, to the Results or Discussion sections (I always prefer discussing the affinities of a specimen and its ontogenetic stage in the Discussion section, personally).

- Regarding figures, I would only suggest the authors to make the convex hull and the arrows of the variables used in the PCA better visible to the reader, in figure 6. Many taxa must also be italicized, while the capital letters of some titles of articles should be removed, in the bibliography section.

I provided additional corrections, remarks and suggestion in the pdf version of their MS, and ask the authors to take them into consideration.

Kind regards,

Christophe Hendrickx, San Miguel de Tucumán, the 14th of March 2020.

Dirección postal: UEL, Miguel Lillo 251 – San Miguel de Tucumán – CP 4000
Tel: (0381) 4972946 – E-Mail: adm.uel@tucuman-conicet.gob.ar
