# Supplementary material for: The first juvenile dromaeosaurid (Dinosauria: Theropoda) from Arctic Alaska
Source: PLoS One. 2020 Jul 8;15(7):e0235078. doi: 10.1371/journal.pone.0235078 (PMC7343144; doi:10.1371/journal.pone.0235078)
Supplement: Attachment — Submitted filename: Chiarenza et al., 2020. Hendrickx review.pdf [file pone.0235078.s012.pdf]

PLOS ONE

The first juvenile dromaeosaurid (Dinosauria: Theropoda) from Arctic Alaska

--Manuscript Draft--

**Manuscript Number:**	PONE-D-20-05495
**Article Type:**	Research Article
**Full Title:**	The first juvenile dromaeosaurid (Dinosauria: Theropoda) from Arctic Alaska
**Short Title:**	A juvenile Saurornitholestinae theropod from Alaska
**Corresponding Author:**	Alfio Alessandro Chiarenza<br>Perot Museum of Nature and Science<br>Dallas, Texas UNITED STATES
**Keywords:**	Theropoda; Cretaceous; Alaska; Dromaeosauridae; Paleobiology; Arctic; Dinosaurs; Dinosauria; Saurornitholestinae; Paleobiogeography; Paleodiversity; North America
**Abstract:**	Compared to the more complete osteological record of herbivorous dinosaurs from the Prince Creek Formation of northern Alaska, the theropod record has provided relatively fewer remains. While most of the theropod record from this unit comprises isolated teeth, the only non-dental remains known can be attributed to the troodontid cf. Troodon and the tyrannosaurid Nanuqsaurus . The presence of members of the Dromaeosauridae have thus far been limited to isolated teeth. Here we describe a symphyseal portion of a dentary with two ziphodont teeth of diminutive size. Based on teeth shape, denticle morphology, the position of the Meckelian groove, and a morphological comparison based on other theropod taxa we attribute this partial dentary to a saurornitholestine dromaeosaurid. The fibrous bone surface, small size, and higher number of mesial denticles compared to distal ones point to a juvenile status for this individual. Multivariate exploration of theropod teeth morphospace by means of principal component analysis reveals an overlap between the Pediomys Point dentary and Saurornitholestinae dromaeosaurid morphospace. This is the first confirmed non-dental fossil specimen from a member of Dromaeosauridae in the Arctic, expanding on the role of Beringia as a dispersal route for this clade between Asia and North America. Furthermore, the juvenile nature of this individual adds to a growing body of data that suggests that Cretaceous Arctic dinosaurs of Alaska did not undergo long-distance migration, but rather they were year round residents of these paleopolar latitudes.
**Order of Authors:**	Alfio Alessandro Chiarenza
	Anthony R. Fiorillo
	Ronald S. Tykoski
	Paul J. McCarthy
	Peter P. Flaig
**Additional Information:**	
**Question**	**Response**
**Financial Disclosure**<br><br>Enter a financial disclosure statement that describes the sources of funding for the work included in this submission. Review the submission guidelines for detailed requirements. View published research articles from *PLOS ONE* for specific examples.<br><br>This statement is required for submission	ARF received funding for this project through the Perot PaleoClub, a private donation.The Perot Paleo Club played no role in the study design, data collection and analysis, decision to publish, or preparation of the manuscript.

Powered by Editorial Manager® and ProduXion Manager® from Aries Systems Corporation

and **will appear in the published article** if the submission is accepted. Please make sure it is accurate.<br><br>**Unfunded studies**<br>Enter: *The author(s) received no specific funding for this work.*<br><br>**Funded studies**<br>Enter a statement with the following details:<br>• Initials of the authors who received each award<br>• Grant numbers awarded to each author<br>• The full name of each funder<br>• URL of each funder website<br>• Did the sponsors or funders play any role in the study design, data collection and analysis, decision to publish, or preparation of the manuscript?<br>• **NO** - Include this sentence at the end of your statement: *The funders had no role in study design, data collection and analysis, decision to publish, or preparation of the manuscript.*<br>• **YES** - Specify the role(s) played.<br><br>* typeset	
**Competing Interests**<br><br>Use the instructions below to enter a competing interest statement for this submission. On behalf of all authors, disclose any competing interests that could be perceived to bias this work—acknowledging all financial support and any other relevant financial or non-financial competing interests.<br><br>This statement **will appear in the published article** if the submission is accepted. Please make sure it is accurate. View published research articles from *PLOS ONE* for specific examples.	The authors have declared that no competing interests exist.

**NO authors have competing interests**<br><br>Enter: *The authors have declared that no competing interests exist.*<br><br>**Authors with competing interests**<br><br>Enter competing interest details beginning with this statement:<br><br>*I have read the journal's policy and the authors of this manuscript have the following competing interests: [insert competing interests here]*<br><br>* typeset	
**Ethics Statement**<br><br>Enter an ethics statement for this submission. This statement is required if the study involved:<br><br>• Human participants<br>• Human specimens or tissue<br>• Vertebrate animals or cephalopods<br>• Vertebrate embryos or tissues<br>• Field research<br><br>Write "N/A" if the submission does not require an ethics statement.<br><br>General guidance is provided below. Consult the submission guidelines for detailed instructions. **Make sure that all information entered here is included in the Methods section of the manuscript.**	N/A

Powered by Editorial Manager® and ProduXion Manager® from Aries Systems Corporation

**Format for specific study types**<br><br>**Human Subject Research (involving human participants and/or tissue)**<br>• Give the name of the institutional review board or ethics committee that approved the study<br>• Include the approval number and/or a statement indicating approval of this research<br>• Indicate the form of consent obtained (written/oral) or the reason that consent was not obtained (e.g. the data were analyzed anonymously)<br><br>**Animal Research (involving vertebrate animals, embryos or tissues)**<br>• Provide the name of the Institutional Animal Care and Use Committee (IACUC) or other relevant ethics board that reviewed the study protocol, and indicate whether they approved this research or granted a formal waiver of ethical approval<br>• Include an approval number if one was obtained<br>• If the study involved *non-human primates*, add *additional details* about animal welfare and steps taken to ameliorate suffering<br>• If anesthesia, euthanasia, or any kind of animal sacrifice is part of the study, include briefly which substances and/or methods were applied<br><br>**Field Research**<br><br>Include the following details if this study involves the collection of plant, animal, or other materials from a natural setting:<br>• Field permit number<br>• Name of the institution or relevant body that granted permission	
**Data Availability**<br><br>Authors are required to make all data underlying the findings described fully available, without restriction, and from the time of publication. PLOS allows rare exceptions to address legal and ethical concerns. See the PLOS Data Policy and FAQ for detailed information.	Yes - all data are fully available without restriction

A Data Availability Statement describing where the data can be found is required at submission. Your answers to this question constitute the Data Availability Statement and **will be published in the article**, if accepted.<br><br>**Important:** Stating 'data available on request from the author' is not sufficient. If your data are only available upon request, select 'No' for the first question and explain your exceptional situation in the text box.<br><br>Do the authors confirm that all data underlying the findings described in their manuscript are fully available without restriction?	
**Describe where the data may be found in full sentences. If you are copying our sample text, replace any instances of XXX with the appropriate details.**<br><br>• If the data are **held or will be held in a public repository**, include URLs, accession numbers or DOIs. If this information will only be available after acceptance, indicate this by ticking the box below. For example: *All XXX files are available from the XXX database (accession number(s) XXX, XXX.*).<br>• If the data are all contained **within the manuscript and/or Supporting Information files**, enter the following: *All relevant data are within the manuscript and its Supporting Information files.*<br>• If neither of these applies but you are able to provide **details of access elsewhere**, with or without limitations, please do so. For example:<br><br>*Data cannot be shared publicly because of [XXX]. Data are available from the XXX Institutional Data Access / Ethics Committee (contact via XXX) for researchers who meet the criteria for access to confidential data.*<br><br>*The data underlying the results presented in the study are available from (include the name of the third party*	ll relevant data are within the manuscript and its Supporting Information files

Powered by Editorial Manager® and ProduXion Manager® from Aries Systems Corporation

and contact information or URL).<br>• This text is appropriate if the data are owned by a third party and authors do not have permission to share the data.<br><br>* typeset	
Additional data availability information:	

Powered by Editorial Manager® and ProduXion Manager® from Aries Systems Corporation

Manuscript
Click here to download Manuscript Chiarenza et al. Alaskan dromaeosaurid - Submission.docx

The first juvenile dromaeosaurid (Dinosauria: Theropoda) from Arctic Alaska

Alfio Alessandro Chiarenza[1*], Anthony R. Fiorillo[1], Ronald S. Tykoski[1], Paul J. McCarthy[2], Peter P. Flaig[3]

1 Perot Museum of Nature and Science, Dallas, TX, USA, **2** Department of Geosciences, University of Alaska, Fairbanks, AK, USA **3** Jackson School of Geosciences, Bureau of Economic Geology, University of Texas at Austin, Austin, TX, USA

* a.chiarenza15@gmail.com

Abstract

Compared to the more complete osteological record of herbivorous dinosaurs from the Prince Creek Formation of northern Alaska, the theropod record has provided relatively fewer remains. While most of the theropod record from this unit comprises isolated teeth, the only non-dental remains known can be attributed to the troodontid cf. *Troodon* and the tyrannosaurid *Nanuqsaurus*. The presence of members of the Dromaeosauridae have thus far been limited to isolated teeth. Here we describe a symphyseal portion of a dentary with two ziphodont teeth of diminutive size. Based on teeth shape, denticle morphology, the position of the

Meckelian groove, ~~and a morphological comparison based on other theropod taxa~~ we attribute this partial dentary to a saurornitholestine dromaeosaurid. The fibrous bone surface, small size, and higher number of mesial denticles compared to distal ones point to a juvenile status for this individual. Multivariate exploration of theropod teeth morphospace by means of principal component analysis reveals an overlap between the Pediomys Point dentary and Saurornitholestinae dromaeosaurid morphospace. This is the first confirmed non-dental fossil specimen from a member of Dromaeosauridae in the Arctic, expanding on the role of Beringia as a dispersal route for this clade between Asia and North America. Furthermore, the juvenile nature of this individual adds to a growing body of data that suggests that Cretaceous Arctic dinosaurs of Alaska did not undergo long-distance migration, but rather they were year round residents of these paleopolar latitudes.

Competing interests: the authors have declared that no competing interests exist.

Introduction

Dromaeosauridae [1–3] (Table S1) is a group of predatory theropod dinosaurs evolutionarily close to the origin of birds [4, 5]. This clade originated in

the Middle Jurassic [6, 7], and by the Late Cretaceous reached a virtually cosmopolitan distribution [8], remaining, so far, unknown only in Antarctica. Given the small to medium size of most dromaeosaurids, and their fragile, highly pneumatic skeleton that is subject to higher incompleteness bias than many other dinosaur taxa [9, 10], complete remains from this group are generally rare, and confined to exceptionally productive fossil localities [e.g. 11, 12]. North American taxa belong to at least 4 recognized major subclades (Dromaeosaurinae, Microraptorinae, Saurornitholestinae and Velociraptorinae; Table S1) with probable Asian origins based on phylogenetic inference and local abundance of taxa referred to these clades. Since the earliest discoveries of dinosaur remains in the Alaskan North Slope (13–15), the number of studies describing dinosaurs from the Prince Creek Formation and their role in clarifying palaeobiogeographic and palaeoecological characteristics of the Cretaceous Arctic has greatly increased [e.g. 16–23).

Dinosaur teeth often preserve more easily and are more frequently recovered than bones, and the discovery of isolated teeth referable to Dromaeosauridae in many Late Cretaceous microsites has often provided important biogeographic data confirming the presence of the group in areas for which purely osteological remains are unknown [8, 24]. For example, Fiorillo and Gangloff [25] reported on isolated dromaeosaurid teeth from the Prince Creek Formation of Alaska,

tentatively referring them to *Dromaeosaurus* and *Saurornitholestes*. Given the intermediate palaeogeographic position of Alaska (as part of the ancient Beringian landmass), and its role as a land bridge between Asia and North America, additional dromaeosaurid remains with better resolved taxonomical identification have the potential to increase our understanding of the origin and dispersal of these clades through Asiamerica. Here we describe the first non-dental, osteological material belonging to a saurornitholestine dromaeosaurid, representing a purported unique morphotype. This finding supports the presence of a previously unrecorded clade on the basis of osteological remains from the Upper Cretaceous (lower Maastrichtian) Prince Creek Formation on the North Slope of Alaska (70° N, Fig 1).

Materials and Methods

DMNH 21183 is a symphyseal portion of a theropod dentary with a semi-erupted tooth and a replacement tooth preserved. The specimen was studied using a Nikon SMZ motorized stereomicroscope (Nikon Instruments, Inc., Melville, NY, USA), equipped with epi-fluorescence with an X-Cite XYLIS light source (Excelitas Technologies, Waltham, MA, USA) with GFP filter. Imaging of the specimen, including focal stacking and 3-D reconstructions, was completed using an attached Nikon Ri2 color CMOS camera with Nikon's NIS-Elements Acquisition and

Analysis Software. Additional microscope observations and imaging were carried out using a Digital VHX-7000 series microscope. Dental nomenclature and terminology is based on Hendrickx et al. [26]. Terminology regarding ontogenetic characters is mostly, but not exclusively based on Sampson et al. [27], Carr [28], Hone et al. [29]. Anatomical description is based on morphological observation by three of the authors (AAC, AF, RT). Comparisons have been made based on first hand observation on relevant material by three of us (AAC, AF, RT), as well as literature comparisons. Stratigraphic and sedimentological observations were carried out by three of the authors of the manuscript (AT, PF, PM) between 1988 and 2014 (Fig 2). Quantitative measurements on teeth morphometrics were based on a modified dataset from Larson and Currie [30]. This dataset includes measurement data for over 1200 small theropod teeth mainly from the latest Cretaceous of the Western Interior Basin of North America, and have been used for similar studies in the past [e.g. 31, 32]. Principal measurements included fore-aft basal length (FABL), crown height (CH), basal width (BW), anterior (ADM) and posterior denticles per millimeter (PDM). The dataset was modified by removing all entries with at least one variable missing or uncodifiable, like the lack of a measurement caused by the absence of the related structure (e.g. ADM in teeth without mesial denticles or carina). In this dataset, the taxonomy of the identified teeth was referred to the "family-ranked" clade. For example, *Saurornitholestes langstoni* is identified as

Saurornitholestinae. *Atrociraptor marshalli* is referred to Saurornitholestinae. *Zapsalis abradens* is referred to Dromaeosaurinae. The Aquilan cf. *Richardoestesia gilmorei*, the Oldman cf. *Richardoestesia gilmorei*, the Horseshoe Canyon cf. *Richardoestesia*, the Lancian cf. *Richardoestesia*, the Aquilan cf. *Richardoestesia isosceles* and *Richardoestesia isosceles* are all referred to *Richardoestesia*. The examined subclades of theropods included in this dataset, apart from DMNH21183 are: Dromaeosaurinae, *Richardoestesia*, Saurornitholestinae and Troodontidae. We performed a principal components analysis (PCA), a multivariate technique that takes a number of measurements distilling them into a smaller set of values representing the variability of the sample to be plotted in a multivariate space. This allows the dataset of teeth to be plotted in a morphospace, and to quantitatively estimate the degree of overlap and the relative position of DMNH 21183 within the examined clades (e.g., Dromaeosaurinae, Troodontidae).

Institutional Abbreviations

AMNH–American Museum of Natural History, New York City, USA; CAGS–Chinese Academy of Geological Sciences, Beijing, China; DMNH–Perot Museum of Nature and Science, Dallas, Texas, USA; IVPP–Institute for Vertebrate Paleontology and Paleoanthropology, Beijing, China; MPC-D–Institute of Paleontology and Geology, Mongolian Academy of Sciences (formerly known as

IGM), Ulaanbaatar, Mongolia; YFGP–Yizhou Fossil and Geology Park, Liaoning, China; YPM–Yale Peabody Museum of Natural History, Yale, Connecticut, USA; NHMUK PV–Natural History Museum, London, UK; TMP–Royal Tyrrell Museum of Palaeontology, Drumheller, Alberta, Canada

Results

Systematic Paleontology

Dinosauria [33]

Theropoda [34]

Dromaeosauridae [1]

Saurornitholestinae [35] indet.

~~Check Table S1 for systematic definition of clades mentioned here and used throughout the text.~~

Referred specimen

DMNH 21183. The anterior portion of a right dentary, preserving two teeth and four alveoli (Fig 3).

Locality

The specimen was collected from exposures of the Prince Creek Formation, at the Pediomys Point locality (Figs 1 and 2). The locality is located along the Colville River, 8 km upstream from the Liscomb bonebed, North Slope Borough, Alaska, USA (Fig 1). Bulk sediment was collected at the site over multiple field seasons between 1988 and 2014, with screenwashing and sorting of the material conducted at the Perot Museum of Nature and Science (DMNH) in Dallas, Texas, USA.

Geological setting and Depositional Environments

The Prince Creek Formation (PCF) was deposited in the Colville Basin of northern Alaska and provides us with the largest collection of polar dinosaur bones in the world [23]. The PCF was originally sub-divided into two subunits or tongues: an older Tuluvak Tongue, and a younger Kogosukruk Tongue [36]. However, Mull et al. [37] revised this nomenclature based on regional stratigraphic correlations and the Prince Creek Formation was redefined to include only the former Kogosukruk Tongue along with some younger, Paleocene strata. The total thickness of the PCF along the Colville River is approximately 450 m [38, 39]. Biostratigraphic [40–49], and isotopic analyses [50] indicate that the age of the Prince Creek Formation ranges from Campanian to Paleocene. However, all

deposits containing evidence of dinosaurs are Early Maastrichtian in age, or approximately 69-70 million years old [47, 49, 51–53].

The PCF is an alluvial succession composed of conglomerate, sandstone, siltstone, mudstone, carbonaceous shale, coal, and bentonite [49, 51, 54]. The mostly fine-grained sediments record deposition in low energy, suspended load channels and on organic-rich floodplains on a low-gradient coastal plain. Thicker, multi-story meandering trunk channels contain the largest grain sizes and record the highest flow velocities in the area. Smaller meandering and fixed or anastomosed distributary channels formed crevasse splay-complexes adjacent to trunk channels. Abundant organic-rich facies were deposited in low-lying areas between the large channels and splay-complexes. Floodplains contained levees, splays, lakes, ponds, swamps, and soil-forming environments. Volcanic ashfall was common and smectite-rich bentonites were commonly preserved in wet floodplain environments [55]. Trampling of sediments by dinosaurs was common [53]. Weakly-developed paleosols similar to modern *Entisols*, *Inceptisols*, andic soils, and potential acid sulfate soils formed on levees, point bars, crevasse splays and along the margins of floodplain lakes, ponds, and swamps that also supported lowland trees, shrubs, herbs, ferns, moss, and algae [51, 55]. Macroscopic and micromorphological features, and illite-smectite mixed-layer clay minerals in paleosols indicate predominantly waterlogged, reducing conditions interrupted by

oxidizing conditions and periodic drying out of some soils [51, 52, 54, 55]. Soils experienced repeated sediment influx from overbank flooding of nearby distributary channels, and periodic deposition of hyperconcentrated flows [52]. Sediments deposited in the most distal areas of the coastal plain contain evidence of marine influence that includes inclined heterolithic stratification in channel point bars and pyrite, jarosite mottles, jarosite halos surrounding root-traces, and brackish-water fauna in floodplain facies [54, 56].

The 750 m-long outcrop belt at Pediomys Point is located along a slough off the main course of the Colville River (Fig 1). Cretaceous environments preserved at Pediomys Point include a meandering distributary channel that transitions laterally into a silt and mud-filled abandoned channel along with floodplain environments that include crevasse splays, levees, small lakes and swamps, floodplain paleosols, and ashfall deposits [57, Fig 2). Trampled sediments found above a bentonite (Fig 2A) are similar to those described from strata 4.25 kilometers downstream along the Colville River that are interpreted as adult and juvenile hadosaur tracks emplaced along a swamp margin [53]. Rare brackish-water clams (most likely *Nucula* aff. *N. percrassa* Conrad; see [56]) and gastropods found near the top of the stratigraphic succession at Pediomys Point suggest an estuarine or lagoonal environment for those deposits [56]. Interfingering of continental-terrestrial and shallow marine deposits, including those of

floodbasins, interdistributary bays and estuaries were identified in older deposits of the PCF along the Colville River [58] and in younger deposits above the Liscomb Bonebed near Ocean Point [59]. This suggests that the environments at Pediomys Point were transitional between the subaerial coastal plain or delta plain, and shallow marine habitats (Fig 2B).

Description

DMNH 21183 (Fig 3) is an anterior portion of a right dentary with an unerupted anterior-most tooth (d2; Fig 3A–3C) and a posteriorly placed partially erupted tooth (d3; Fig 3). The anterior surface of the dentary is damaged, obscuring details of the symphyseal region. Given the position of the ~~most~~ erupted tooth (d3; Fig 3) above the Meckelian foramina (on the medial surface of the dentary, Fig 3B), and above an anteroventral process of the dentary (ave; Figs 3A and 3B), we identify this as the 3rd tooth in the dentary [e.g. 60, 61], with the anteriorly positioned, less erupted tooth (d2) being identified as the 2nd. Alveoli 2–4 are preserved (Fig 3A–C), although the margins of the latter are obscured by erosion. Both the medial and lateral surfaces of the dentary present a parallel, anteroposteriorly oriented fibrous texture. The anterior margin of the 3rd alveolus (a3; Fig 3A–C) shows a raised anterior rim that extends dorsally up to approximately mid-height of the crown of the relative tooth (a3). There is a well-

preserved subtriangular interdental plate lingual to the septum dividing the 2nd and 3rd alveoli (a2–a3), and a bigger, semicircular one between the 3rd and 4th alveoli (a3–a4). The paradental space is relatively shallow. Laterally, the dorsal rim of the dentary rises into a triangular ridge, which creates a convex surface anterolaterally that sinks at its base into a circular fossa. The alveoli are elliptically shaped, slightly more anteroposteriorly elongated rather than mediolaterally so (Fig 3C). The medial side of the dentary features a shallow Meckelian groove (Fig 3B), set slightly more ventrally than mid-way depth of the bone. The medial side of the dentary features a anteriorly located Meckelian foramen (Fig 3B), which may be paired with a second, more ventrally positioned foramen, but damage to the bone surface in this area makes the identification of this feature uncertain. There is an anteroventral expansion in the margin of the dentary, visible both medially and laterally (Figs 3A and 3B), but excavated by a sub-oval fossa on the lateral side (Fig 3A).

Both preserved teeth in DMNH 21183 are ziphodont. The mesodistal axis of the 2nd tooth is more anteromedially oriented in relation to the lateral margin of the dentary. The 2nd tooth is unerupted, but damaging to the medial surface of the alveolar wall exposes the most apical half of the tooth crown. While the anterior alveolar margin covers the mesial surface of the 2nd tooth, denticles are visible along the distal carina, with 13 denticles (Figs 4A and 4B). The apical-most half of

portion of the 3rd tooth is erupted, with 12 denticles visible on the distal carina (Figs 3A, 3B and 3D).

The preserved teeth show coarser distal denticles than the mesial ones. Although the erupted portion of the crown in both teeth d2 and d3 is exposed only in the apical-most half, with 13 distal denticles (for tooth d2) and 12 denticles (for tooth d3), the unerupted portion of the crown of tooth 3 can be observed through a fracture on the medial side of the dentary (Fig 3B). Taking into account the base of this crown, a hypothetical estimate of ~30 denticles per serrated margin can be inferred.

Unfortunately, wear of the mesial carina destroyed some morphological details of the denticles. However, apart from some denticles, the interdenticular grooves between adjacent denticles are clear, and they are shallow incisions rather than deep sulci (Fig 3D, S1 Fig). In the 2nd tooth this comparison between mesial and distal carina is not possible because the mesial ~~(anterior)~~ margin of the tooth is hidden by sediment and the labial wall of the alveolus (Figs 4A and 4B). The shape of the distal denticles is slightly hooked, with an orientation toward the apex of the crown and with an externally rounded margin rather than pointed (Figs 4A–4C).

Parallel, anterocaudally oriented fibrous texture is present on the surfaces of both the medial and lateral sides of the dentary in DMNH 21183. External textural features in dinosaur surface bone have been shown to change in relation to

ontogeny [29], in a process mirroring internal microscopic remodeling [62]. For example, the skulls of ceratopsian dinosaurs show surface textural changes during ontogeny, from lightly striated to deeply rugose textures [27; 63]. Cortical bone texture with fine grained, longitudinally striated pattern [28] is considered a size-independent criterion as an indication of relative immaturity in non-avian archosaurs [27, 28, 64]. Lightly striated cortical bone grains express nascent ontogenetic characters in theropods [28], as it is particularly clear from *Tyrannosaurus* [28], *Scipionyx* [65], and *Juravenator* [66, 67]. The same immature bone grain texture described in relatively immature individuals of these taxa is also present in DMNH 21183 (Figs 3A and 3B). This textural feature, in consideration of the diminutive size of the specimen (Table 1), points toward a juvenile status of DMNH 21183.

Table 1. Measurements of DMNH 21283

Elements measured	Measurements (mm)
Dentary anteroposterior length (dorsal view)	14.34
Dentary anteroposterior length (lateral view)	14.59
Maximum dentary mediolateral length	6.15
Maximum dentary dorsoventral depth	9.44
Alveolus II mesodistal length	2.95
Alveolus II labiolingual width	1.27
Alveolus III labiolingual width	2.76
Alveolus III mesodistal length	3.71
Tooth II mesodistal width	1.97
Tooth II labiolingual length	1.05

Morphological remarks

DMNH 21183 is an anterior portion of a right dentary. While the specimen is fragmentary, a list of anatomical characters can be traced and compared to identify its systematic position. Although very few non-dental theropod remains have been found in the Prince Creek formation of Alaska, a general comparison with other theropod taxa, with a particular focus on those clades previously recognized in these units (Dromaeosauridae, Tyrannosauridae, Troodontidae), is attempted here. The combination of a ziphodont dentition, presence of interdental plates (e.g. [68]), a Meckelian groove and Meckelian foramina is characteristic of theropod dinosaurs (e.g. [69]). The orientation of the anterior-most tooth socket and its unerupted tooth (both anteromedially oriented), the presence of paired Meckelian foramina, and a ventral expansion laterally pierced by a fossa, point toward the identification of the piece as an anterior portion (almost symphyseal, as also shown by the ventral enlargement of the dentary in lateral view) of a theropod dentary. The shallow Meckelian groove present in the specimen is a derived maniraptoran feature [70], in contrast to deep groove seen in the basal tyrannoraptoran (e.g. Tyrannosauroidea) condition [71]. The Meckelian foramen (Fig 3A) is slit-like and not enlarged and rounded like in Tyrannosauridae (e.g. oral mandibular foramen in *Nanuqsaurus* [21] and foramen intramandibularis oralis in *Tyrannosaurus* [72]), a feature seen in Dromaeosauridae (e.g. *Dromaeosaurus*, [73]; e.g. *Acheroraptor*, [61]). The shallow

paradental space, a Meckelian groove which is set in the lower half of the medial side of the dentary but not directly on the ventral margin, and the hooked distal denticles of the teeth are coelurosaurian combination features [74, 75]. The oval-lenticular shape of the alveoli from dorsal view (Fig 3C; S1 Fig) rather than box shaped (or more squared off) is more similar to the condition in derived coelurosaurians (i.e. Maniraptora) rather than in Tyrannosauroidea [26]. The presence of distinct alveoli rather than a connected dorsal furrow excludes derived troodontids [26]. The triangular ridge on the lateral margin of the dentary (Figs. 3A and 4D) is shared with *Buitreraptor* [76]. The raised rim in front of the relatively better-preserved alveolar rim 4 (Fig 3A) resembles the condition of the anterior alveoli in *Saurornitholestes* (e.g. [60]). Interdental plates are notoriously rare in paravian theropods, and fusion or reduction of these structures has been largely considered synapomorphic for Dromaeosauridae or Deinonychosauria as a whole, except for some taxa like Microraptorinae [77], *Austroraptor* [78], *Acheroraptor* [61], and *Archaeopteryx* [79, 80]. The larger of the preserved interdental plates of DMNH 21183 is broadly triangular in shape (like *Acheroraptor*), with a somewhat arched apex and a broader base. The absence of medial crenulation or dorsoventral furrows in the interdental plate excludes it from assignment to Tyrannosauridae. The presence of interdental plates is unique, between North American Dromaeosauridae, apart from *Acheroraptor*.

The lenticular (more mediolaterally compressed than circular) cross section of the teeth in DMNH 21183 differs from the mesial and lateral dentitions of many troodontids, which have a subcircular cross-sectional outline at the crown base [26]. A non-diminutive first dentary tooth, subequal in size with more distally positioned dentary teeth excludes Tyrannosauridae (e.g. [21]) and is typical of more derived Coelurosauria and paravians [26]. The teeth of DMNH 21183 lack concave surfaces adjacent to both carinae (Fig 4) as has been observed in some mesial teeth of the troodontids *Troodon* [81, 82], *Urbacodon* [83], an indeterminate troodontid taxon from Uzbekistan [83], and the troodontid *Xixiasaurus* [26, 84]. As in most paravians, the teeth preserved in DMNH 21183 exhibit short interdenticular sulci (opposite of the well-developed and deep sulci present in Abelisauridae, Tyrannosauridae, and Allosauroidea; Fanti and Therrien [8, 26], whereas short interdenticular sulci have been observed in the microraptorine specimen IVPP V13476 [85], the eudromaeosaurians *Deinonychus* (YPM 5232), *Saurornitholestes* [82] and *Dromaeosaurus* (AMNH 5356; [82, 86], as well as in some troodontids [87, 88] such as *Troodon* (NHMUK PV R.12568).

The distal denticles with a relative higher density compared to the much coarser denticles in Troodontidae is more similar to the condition in Dromaeosauridae [26]. The presence of coarser distal denticles is a more common feature found in saurornitholestine dromaeosaurids rather than Dromaeosaurinae

[30]. An affinity to the sympatric cf. *Troodon* [89] can be excluded on the basis of serrated mesial teeth, which are un-serrated in this troodontid taxon [90]. An affinity with basal dromaeosaurid taxa like the halszkaraptorines and unenlagiines (e.g. *Halszkaraptor* [91], *Mahakala* [92], *Buitreraptor* [76] and *Austroraptor* [93]) can be excluded, as the dentition of these taxa are devoid of denticles [26]. Other deinonychosaurian taxa like the troodontids *Mei* [94], *Byronosaurus* [95], *Gobivenator* [96], *Urbacodon* [83], *Xixiasaurus* [84], IVPP V20378 and *Jinfengopteryx* [97], *Almas* [98, 99] and MPC-D 100-1128, the anchiornithines *Anchiornis* (IVPP V16055; [100]), *Eosinopteryx* (YFGP-T5197; [101]), *Aurornis* (YFGP-T5198; [102]) and the basal avialan *Archaeopteryx* (e.g., [103–105]) also have non-denticulate tooth crowns all along their jaws [26], excluding a potential affinity with DMNH 21183. It has to be cautioned though that independent reacquisition of denticulated teeth has been shown in some deinonychosaurian taxa like the anchiornithid *Caihong* and *Liaoningvenator*, in the derived Troodontinae (i.e. the node encompassing *Sinovenator* + more derived forms like *Troodon* and *Pectinodon*), and the clade Microraptorinae + Eudromaeosauria (sensu Turner et al. [92]).

The reduction in size of mesial denticles is similar to the condition in Saurornitholestinae rather than Dromaeosaurinae [60, 73, 106–109]. The shape of the denticles is slightly pointed toward the apex of the crown as in Dromaeosauridae

and Troodontidae and not C- or U-shaped like in Tyrannosauridae [26]. The pair of anterior foramina nearby the symphysis recalls a similar condition found in Velociraptorinae like *Acheroraptor* [61].

The reduction or lack of mesial denticulation is shared with some Asian Velociraptorinae like *Tsaagan* [110]. A similar condition to DMNH 21183 where mesial teeth bear un-serrated/absent mesial carinae and denticulated distal carinae is present in many other theropod clades. This feature is observed in the dromaeosaurid *Tsaagan* [110], the troodontids *Linhevenator* [26, 111] and possibly *Saurornithoides* (AMNH 6516 [112]).

Although hooked denticles are primarily present in some derived troodontid taxa [94] as seen in *Pectinodon* [113], *Saurornithoides* [112], *Sinornithoides* [87], *Troodon* (e.g., 81, 82, 113, 114), in indeterminate troodontid taxa from France [108, 115] and Asia [83], and in some anchiornithines like *Caihong* [116], there are also dromaeosaurids that exhibit apically hooked denticles (e.g., [73, 82, 86, 113]). These include the eudromaeosaurians *Atrociraptor* (TMP 1995.166.01; [117]) and *Saurornitholestes* [82, 118]. A main and immediately noticeable difference between denticles in Dromaeosauridae and Troodontidae, is that the latter tend to bear particularly large, bulbous, and widely separated denticles, while Dromaeosauridae have more numerous, smaller and asymmetrically convex or parallelogram-shaped denticles (Fig 4A; [26]). Other deinonychosaurian taxa lack apically hooked

denticles, with a morphology that is more symmetrical and apically convex, such as in Microraptorineae (e.g. *Graciliraptor* [IVPP V2339], *Microraptor* [CAGS-20-7-004] and *Sinornithosaurus* [IVPP V12811]), and in derived eudromaeosaurs such as *Acheroraptor* [61], *Bambiraptor* [119], *Linheraptor* [120], *Tsaagan* [121], *Utahraptor* [122] and *Velociraptor* [11].

The number of 12-13 denticles in the exposed crowns of DMNH 21183 produces an estimated total number of denticles per carina higher than the ~15 present in many troodontids, particularly in taxa more derived than *Sinovenator* (e.g. *Daliansaurus* [123], *Linhevenator* [111], *Pectinodon* [30, 124], *Saurornithoides* [112], *Sinornithoides* [125], *Sinusonasus* [126], *Troodon* [81, 127, 128] and *Zanabazar* [112]). While some tooth crowns of *Saurornitholestes* appear to have less than 15 denticles on the carina [30, 82, 129] quantitative analyses by Larson and Currie [30] indicates that the large majority of *Saurornitholestes* teeth have much more than 15 denticles on the crown (for other remarks on morphological variation in the dental series of this taxon (see [60]). Within Deinonychosauria, taxa with a large number of denticles (≥6 per 1 mm) are *Richardoestesia*, Saurornitholestinae (including *Saurornitholestes*), *Sinovenator* and *Velociraptor* [26, 30, 130–133].

While the position of the mesial margin of the teeth preserved in DMNH 21183 and some degree of surface abrasion preclude detailed morphological observation, the finely serrated mesial carina, with denticles smaller than their distal

serial homologues, can be seen particularly well in the 3rd tooth of the dentary (d3; Fig 3D). The presence of distal denticles larger than mesial ones was long thought to characterize the dentition of Dromaeosauridae, and therefore used as a primary feature to identify Velociraptorinae versus Dromaeosaurinae teeth (e.g., [106–109]). Teeth with fine mesial serrations are usually characterized by a denticle size index (DSDI) higher than 1.2 while teeth that bear carinae with subequal denticle size have usually a DSDI≤0.9. These arbitrary values were proposed by Rauhut and Werner [106] and corresponds in the case of DSDI≈1.2 to approximately more than six mesial denticles for five distal serrations [26]. DMNH 21183 has a quite high DSDI (~2.3; Fig 5), which is well beyond the range of many deinonychosaurian taxa, and in the range of the most finely serrated Saurornitholestinae teeth. The surprisingly high DSDI may be a juvenile trait, since many juvenile theropods have been shown to exhibit particularly fine mesial serrations relative to the distal ones, and evidence from tyrannosaurids [71, 125, 134] has shown progressively decreasing DSDIs through ontogeny [135]. A DSDI >1.2 has been reported in the majority of eudromaeosaurians, such as the microraptorines *Changyuraptor* [136], *Graciliraptor* [126], *Sinornithosaurus* [137] and *Tianyuraptor* [138], the dromaeosaurines *Achillobator* [139], *Atrociraptor* [117], *Dakotaraptor* [140] and *Utahraptor* [122], the velociraptorines *Acheroraptor* [61], *Bambiraptor* [119], *Deinonychus* [141], *Linheraptor* [120], *Saurornitholestes* [82], and *Velociraptor*

[142], and the European dromaeosaurids *Nuthetes* [143] and *Pyroraptor* [144]. Some lateral teeth of the troodontid *Zanabazar* [145] and some isolated crowns assigned to *Troodon* have also a very high DSDI [81]. This is the opposite condition than that typically found in Dromaeosaurinae (DSDI < 0.9), like in the eponym taxon *Dromaeosaurus* (AMNH 5356).

Multivariate analysis results

Entering DMNH 21183 (the 3rd and more exposed-better preserved tooth; d3 in Fig 3D) in a morphometric dataset [30] of deinonychosaurian teeth (see Materials and Methods) and running a PCA analysis returned 4 axes with the following eigenvalues and percentages of total variance explained by each axis: Axis 1 (15.005, 77.175%), Axis 2 (3.584, 18.433%), Axis 3 (0.559, 2.879%), Axis 4 (0.294, 1.513%). Coefficients for the five measurements on each axis are provided in Table 2.

Table 2. PCA coefficients.

Measurement	PC1	PC2	PC3	PC4
FABL	0.352856	-0.03872	0.568433	0.742211
CH	0.838152	0.423491	-0.31658	-0.13392
BW	0.176441	-0.00224	0.739112	-0.65006
PDM	-0.37665	0.90507	0.174281	0.092801

Coefficients of the PCA analysis run on the theropod teeth dataset published by Larson and Currie [30] with the addition of DMNH 21183. ADM, anterior denticles per millimeter; BW, basal width; CH, crown height; FABL, fore-aft basal length; and PDM, posterior denticles per millimeter.

The majority of the variance is comprised in the first two axes of the principal components. The highest variable contribution, similar to previous analyses on this dataset [30–32], is provided by CH (~60%) followed by PDM (~30%), FABL (~15%) and lastly ADM (<5%). A morphospace depicting the position of DMNH 21183 in the first two axes of the deinonychosaurian teeth morphospace is presented in Fig 6. DMNH 21183 overlaps the Saurornitholestinae morphospace, being set further away from the centroid clustering the other analyzed taxon with hooked denticles, Troodontidae. It is well outside the convex hull comprising Dromaeosaurinae, which has mostly subequal, rectangular denticles, and is set within the Saurornitholestinae morphospace in a position slightly toward the *Richardoestesia* morphotype. We therefore tentatively refer DMNH 21183 to the Saurornitholestinae, but do not at this time assign it to currently recognized species within the clade.

One major caveat of these analyses regards the potential bias due to the different ontogenetic stages of the teeth included in the sample [31], and most importantly in relation to the likely juvenile status of DMNH 21183. Because PCA

analysis would simply group observations based on measurements, it is likely that teeth belonging to juvenile and adult individuals will cluster in separate areas of the morphospace. This issue has been shown previously (e.g. [146, 147] to affect previous PCAs of tyrannosauroid teeth, so we use this morphometric line of evidence as a complementary tool to assess the systematic identification of DMNH 21183.

Discussion

The discovery of dinosaur remains at high latitudes, and in particular the abundant dinosaur bone record from the PCF, challenged traditional reptilian models for dinosaurian physiologies and inspired debate centered on the potential for long-distance migrations by dinosaurs [18, 148–150]. Such increased interest showed, through a variety of methods such as biomechanic, isotopic analyses, and osteohistology, that these dinosaurs likely had the necessary adaptations for overwintering in the ancient Arctic [18, 151, 152). Thus far, these discussions have focused almost exclusively on herbivorous taxa, with one exception that was based on a speculative argument that if the dinosaurian prey did not migrate, then the predators were non-migratory as well [25].

In their review of the adaptive benefits of migration for modern mammals, Avgar and others [155] showed that in the terrestrial realm there is a decided

preference for mammalian long-distance migrations to occur among large-bodied herbivores rather than carnivores. One of the suggested reasons for the rarity of migration by mammalian carnivores is that for purposes of energy consumption and mating, these animals need to establish, maintain, and defend territories. Such behavior would preclude the ability to migrate. Using birds as analogue for inferring non-avian theropods' migratory strategies is problematic, as the lack of active flight in the latter preclude an energetically efficient way of covering large distances when constrained by terrestrial locomotion. Large, mostly land-dwelling predatory birds, like for example the shoe-billed stork (*Balaeniceps rex*) or the secretary bird (*Sagittarius serpentarius*), are usually non-migratory and their distribution is more strongly constrained by resource availability [154, 155]. Using these observations for interpreting behavior in predatory Arctic dinosaurs, it is reasonable to assume that Arctic dromaeosaurids employed similar life strategies and did not migrate. Given the rarity of Arctic fossil vertebrate material, it is not surprising that direct evidence from the fossil record on potential migratory habits in non-avian theropods are scant and based on secondary evidence. For example, the higher abundance and relatively large size (compared to similar, more southern assemblages) of a troodontid taxon in Alaska [89] seems to imply a higher success at these latitudes compared to the southerner relatives. This advantage might be due to increased success favored by high-latitude adaptations like bigger eyes, conferring enhanced

yearly activity even at low-light regime, hence increasing resources exploitation for this taxon. The relatively smaller size of the tyrannosaurid *Nanuqsaurus* [21], compared to the average size of other latest Cretaceous tyrannosaurines, might be due to stronger constrained exerted by a lower carrying capacity in this Arctic ecosystem. Under such a scenario, it is not straightforward to infer for non-avian theropods like the herein described Alaskan Saurornitholestinae either 1) perennial habitat occupation, comprising of mating, nesting and foraging or 2) long-distance migratory habits, potentially driven by either resource tracking or reproductive biology. Although direct evidence of nesting and eggs material in Cretaceous Arctic Alaska are lacking, the implication of the biogeography of several dinosaur taxa (e.g., hadrosaurs, ankylosaurs, ceratopsians, pachycephalosaurs, tyrannosaurs, troodontids, and dromaeosaurs) known in both Siberia and Alaska [23, 156], suggests dispersal capabilities through high-latitude land bridges. As both the theropods and ornithischian taxa so far reported in the Arctic are known to build mound nests or practice brooding [157–160, 161], their perennial subsistence at high-latitude seems realistic [160].

The fibrous bone texture of DMNH 21183 strongly suggests that the individual was very young at the time of its death. Given that there is a demonstrable positive correlation between tooth size and body size, at least in some animals [162, 163], it is reasonable to conclude that the DMNH 21183 belonged to a small

individual. Therefore, this specimen of a very young, small individual suggests that dromaeosaurs likely nested in the ancient Arctic and did not migrate. They were year-round inhabitants of the ancient high-latitude north.

Teeth assigned to other Dromaeosaurinae and *Saurornitholestes* have been reported from the PCF [89], implying that these clades were likely inhabitants of the paleoarctic region throughout the year rather than seasonal migrants. The higher abundance of Dromaeosaurinae in these northern assemblages relative to Saurornitholestinae (based solely on dental material), led Fiorillo [89] to the hypothesis that the generalist bauplan in Dromaeosaurinae [73] might have been ideal of a low-resources ecosystem as the one in this region during the Late Cretaceous. The frequency of similar taxa in the Judith River Formation of Montana and Alberta [164] implies some form of niche partitioning in similarly sized North American predatory paravians, with the more abundant Saurornitholestinae marginalizing the other taxa in the predatory guild (mostly cfr. *Richardoestesia*, troodontids and *Dromaeosaurus*). In the polar assemblages on the other hand, Saurornitholestinae appear rarer, with troodontids standing out not only for relative frequency but also for larger size in this assemblage [17, 89]. The extreme rarity of Saurornitholestinae in this assemblage may be due to competitive exclusion with the other two main sympatric paravian taxa. While the "generalist bauplan interpretation" attributed to Dromaeosaurinae by Currie [73] might just be due to an

older intepretation based on a lower taxonomic sampling than is available today, their higher frequency (taken at face value) might reflect a higher ecological success. The larger size attained by multiple taxa in this clade (e.g. *Atrociraptor*, *Achillobator*, *Utahraptor*, *Dakotaraptor* [80, 81, 139, 141]), and the dentition reminiscent of larger non-maniraptoran theropods (e.g. low DSDI; [26]) might suggest a higher tendency to hypercarnivory than the smaller saurornitholestine. In addition, the hooked denticulation exhibited in this clade may imply a different ecomorphology in this clade relative to Dromaeosaurinae. Originally interpreted as evidence of hypercarnivory [82], the functional implication of hooked denticles in theropod teeth has been differently evaluated. Fowler et al [165] suggested a role in enhancing grip in smaller feathered or furry animals, within taxa having a high numbers of denticles, while the coarser dentition in troodontids is only superficially convergent but implies a different functional role, for example exploitation of smaller and softer preys, like insects or other invertebrates [166] or omnivory [114]. Torices et al [166] suggested for the fine, apically hooked serration of taxa like *Saurornitholestes*, an adaptation for active hypercarnivory of struggling prey. The potential overlap in prey exploitation between Saurornitholestinae and Dromaeosaurinae, but the higher success in developing larger size of the latter, might have caused the marginalization of the smaller saurornitholestines to secondary trophic levels in this low-resource environment of Arctic Alaska. Similarly, the

preadaptation to low-light conditions in troodontids and their larger size might have played an advantageous role in segregating this group from other paravians. Further work has the potential not only to provide the first direct evidence of nesting, early life history and development of Arctic dromaeosaurs, but also of casting more light on the systematic and taxonomy of the Alaskan Saurornitholestinae and their trophic role in this extinct Arctic ecosystem.

The identification of the first osteological remains attributed to Saurornitholestinae supports previous observations on the paleoecology and ecosystem structure of Late Cretaceous communities in Arctic Alaska. Previous workers have suggested that gregarious herbivorous dinosaur groups were non-migratory. The specimen described here (DMNH 21183) is particularly important because it represents a very young individual with little or limited ability to engage in long-distance travel or migrations. As there is a decided preference for modern mammalian long-distance migrations to occur among large-bodied herbivores rather than carnivores, we infer that DMNH 21183 implies a perennial residency of this dromaeosaur clade (Saurornitholestinae) in the Arctic. This new data point adds further weight to the palaeobiogeographical connection between closely related Asian and North American eudromaeosaur taxa (with sister clades present in both Asia and Western Interior Basin). DMNH 21183 is too fragmentary to provide more specific taxonomic distinction within the current record of known

dromaeosaurids, but is most similar to sauronitholestines. Given the geological age of *Saurornitholestes langstoni* and the wide-ranging tooth-form taxon *Richardoestesia* (Late Campanian-Late Maastrichtian), we predict that additional specimens and data may eventually provide evidence supporting establishment of a new dromaeosaurid taxon in the Early to early Late Maastrichtian of Arctic Alaska.

Acknowledgments

We would like to acknowledge Renata Tully (Nikon, Inc.) and Ryan Clubb (Keyence Corp. of America) for microscope and software support. Dori Contreras (Perot Museum of Nature and Science) is also thanked for assistance in data collection and photo editing.

References

1. Matthew WD, Brown B. The family Deinodontidae, with notice of a new genus from the Cretaceous of Alberta. Bulletin of the American Museum of Natural History. 1922; 46: 367–385.

2. Colbert EH, Russell DA. The small Cretaceous dinosaur *Dromaeosaurus*. American Museum Novitates 1969; 2380:1–49.

3. Sereno PC. A rationale for phylogenetic definitions, with application tothe higher–level taxonomy of Dinosauria. Neues Jahrbuch für Geologieund Paläontologie Abhandlungen. 1998; 210: 41–83.

4. Ostrom JH. The ancestry of birds. Nature. 1973; 242:136.

5. Gauthier JA. Saurischian monophyly and the origin of birds. Memoires of the California Academy of Sciences. 1986; 8:1–55.

6. Metcalf SJ, Vaughan RF, Benton MJ, Cole J, Simms MJ, Dartnall DL. A new Bathonian (Middle Jurassic) microvertebrate site, within the Chipping Norton Limestone Formation at Hornsleaslow Quarry, Gloucestershire. Proceedings of the

Geologists' Association. 1992; 103 (4): 321–342. doi:10.1016/S0016-7878(08)80129-0

7. Cau A. The assembly of the avian body plan: a 160-million-year long process, in Bollettino della Società Paleontologica Italiana. 57 (1): 1–25. doi:10.4435/BSPI.2018.01

8. Fanti F, Therrien F. Theropod tooth assemblages from the Late Cretaceous Maevarano Formation and the possible presence of dromaeosaurids in Madagascar. Acta Palaeontologica Polonica. 2007; 52 (1): 155–166.

9. Brown CM, Evans DC, Campione NE, O'Brien LJ, Eberth DA. Evidence for taphonomic size bias in the Dinosaur Park Formation (Campanian, Alberta), a model Mesozoic terrestrial alluvial- paralic system. Palaeogeography, palaeoclimatology, palaeoecology. 2013; 372, 108-122. doi: 10.1016/j.palaeo.2012.06.027

10. Cashmore DD, Butler RJ. Skeletal completeness of the non- avian theropod dinosaur fossil record. Palaeontology. 2019; 62: 951–981. doi:10.1111/pala.12436

11. Norell MA, Makovicky PJ. Important features of the dromaeosaurid skeleton II: information from newly collected specimens of Velociraptor mongoliensis, in American Museum Novitates. 1999; 3282: 1–45

12. Lü J, Brusatte SL. A large, short-armed, winged dromaeosaurid (Dinosauria: Theropoda) from the Early Cretaceous of China and its implications for feather evolution. Scientific Reports. 2015; 5: 11775. doi:10.1038/srep11775

13. Clemens WA, Allison CW. Late Cretaceous terrestrial vertebrate fauna, North Slope, Alaska. Geological Society of America Abstracts with Programs. 1985; 17(7):548.

14. Brouwers EM, Clemens WA, Spicer RA, Ager TA, Carter DL, Sliter WV. Dinosaurs on the North Slope of Alaska: high latitude, latest Cretaceous environments. Science. 1987; 237:1608–1610.

15. Davies K. Duck-bill dinosaurs (Hadrosauridae, Ornithischia) from the north slope of Alaska. J. Paleont. 1987; 61: 198–200

16. Gangloff RA, Fiorillo AR, Norton DW. The first pachycephalosaurine (Dinosauria) from the Arctic of Alaska and its paleogeographic implications. Journal of Paleontology. 2005; 79: 997–1001.

17. Fiorillo AR, Tykoski RS, Currie PJ, Mccarthy PJ, Flaig P. Description of two partial Troodon braincases from the Prince Creek Formation (Upper Cretaceous), North Slope Alaska. Journal of Vertebrate Paleontology. 2009; 29 (1): 178–187. doi:10.1080/02724634.2009.10010370

18. Gangloff RA, Fiorillo AR. The record of Arctic dinosaurs from northern Alaska, paleogeographic and paleoecologic implications. J Vertebr Paleontol. 2003; 23: 53A

19. Brown CM, Druckenmiller P. Basal ornithopod (Dinosauria: Ornithischia) teeth from the Prince Creek Formation (early Maastrichtian) of Alaska. Canadian Journal of Earth Sciences. 2011; 48 (9): 1342–1354. doi:10.1139/e11-017. ISSN 0008-4077

20. Fiorillo AR, Tykoski RS. A new Maastrichtian species of the centrosaurine ceratopsid *Pachyrhinosaurus* from the North Slope of Alaska. Acta

Palaeontologica Polonica. 2012; 57(3): 561-573. doi: http://dx.doi.org/10.4202/app.2011.0033

21. Fiorillo AR, Tykoski RS. A diminutive new tyrannosaur from the top of the world. PLoS ONE. 2014; 9(3): e91287. doi:10.1371/journal.pone.0091287

22. Watanabe A, Erickson GM, Druckenmiller PS. An ornithomimosaurian from the upper Cretaceous Prince Creek Formation of Alaska. J Vertebr Paleontol. 2013; 33: 1169–1175.

23. Fiorillo AR. Alaska Dinosaurs: an ancient Arctic world book. CRC Press, Boca Raton. 2018; 224 p.

24. Torices A, Currie PJ, Canudo JI, Pereda-Suberbiola X. Theropod dinosaurs from the Upper Cretaceous of the South Pyrenees Basin of Spain. Acta Palaeontologica Polonica. 2015; 60 (3): 611–626.

25. Fiorillo AR, Gangloff RA. Theropod teeth from the Prince Creek Formation (Cretaceous) of northern Alaska, with speculations on arctic dinosaur paleoecology. J Vertebr Paleontol. 2000; 20: 675–682.

26. Hendrickx C, Mateus O, Araújo R, Choiniere J. The distribution of dental features in non-avian theropod dinosaurs: Taxonomic potential, degree of homoplasy, and major evolutionary trends. Palaeontologia Electronica. 2019; 22.3.74: 1–110. doi: https://doi.org/10.26879/820

27. Sampson SD, Ryan MJ, Tanke DH. Craniofacial ontogeny in centrosaurine dinosaurs (Ornithischia: Ceratopsidae): taxonomic and behavioral implications. Zool. J. Linn. Soc. 1997; 121: 293–337. (doi:10.1111/j.1096-3642.1997.tb00340.

28. Carr TD. Craniofacial ontogeny in Tyrannosauridae (Dinosauria, Coelurosauria). J Vertebr Paleontol. 1999, 19: 497–520

29. Hone DWE, Farke AA, Wedel MJ. Ontogeny and the fossil record: what, if anything, is an adult dinosaur? Biol Lett. 2016;12:20150947.

30. Larson DW, Currie PJ. Multivariate analyses of small theropod dinosaur teeth and implications for paleoecological turnover through time. PLoS ONE. 2013; 8:e54329. https://doi.org/10.1371/journal.pone.0054329

31. Williamson TE, Brusatte SL. Small Theropod Teeth from the Late Cretaceous of the San Juan Basin, Northwestern New Mexico and Their Implications for Understanding Latest Cretaceous Dinosaur Evolution. PLoS ONE. 2014; 9(4): e93190. https://doi.org/10.1371/journal.pone.0093190

32. Brownstein CD. New records of theropods from the latest Cretaceous of New Jersey and the Maastrichtian Appalachian fauna. Royal Society Open Science. 2019; 6(11):191206.

33. Owen R. Report on British Fossil Reptiles. Report of the British Association for the Advancement of Science. 1842; 11: 60–204. Part 2.

34. Marsh OC. 1881. Classification of the Dinosauria. American Journal of Science 23:241–244

35. Longrich NR, Currie PJ. A microraptorine (Dinosauria–Dromaeosauridae) from the Late Cretaceous of North America. PNAS. 2009; 106 (13): 5002–7. doi:10.1073/pnas.0811664106

36. Gryc G, Patton WW Jr, Payne TG. 1951. Present Cretaceous stratigraphic nomenclature of northern Alaska Journal of the Washington Academy of Sciences, 41: 159-167

37. Mull GG, Houseknecht DW, Bird KJ. Revised Cretaceous and Tertiary stratigraphic nomenclature in the Colville Basin, northern Alaska: U.S. Geological Survey Professional Paper. 2003; 1673, 36 p.

38. Detterman RL, Bickel RS, Grvc G. Geology of the Chandler River region, Alaska. U.S., Geol Surv Profess Papers. 1963; 303-E:219-324.

39. Brosge WP, Whittington CL. Geology of the Umiat-Maybe Creek region, Alaska: U.S. Geological Survey Professional Paper. 1966; 303-H, 501–638,

40. Frederiksen NO, Ager TA, Edwards LE. Comment on "Early Tertiary • marine fossils from northern Alaska: hnplications for Arctic Ocean paleogeography and faunal evolution": Geology. 1986; 14: 802-803.

41. Frederiksen NO. Sporomorph biostratigraphy, floral changes, and paleoclimatology, Eocene and earliest Oligocene of the eastern Gulf Coast U.S. Geol Surv Prof Pap. 1988; 1448: 68

42. Frederiksen NO, Edwards LE, Ager TA, Sheehan TP. Palynology of Eocene strata in the Sagavanirktok and Canning Formations on the North Slope of Alaska. Palynology. 2002; 26: 59-93.

43. Parrish JT, Spicer RA. Late Cretaceous terrestrial vegetation: a near-polar temperature curve. Geology 1988; 16: 22–25.

44. Frederiksen NO. Pollen zonation and correlation of Maastrichtian marine beds and associated strata, Ocean Point dinosaur locality, North Slope, Alaska: U.S. Geological Survey Bulletin. 1991; 1990-E: E1-E24.

45. Brouwers EM, De Deckker P. Late Maastrichtian and Danian Ostracode Faunas from Northern Alaska: Reconstructions of Environment and Paleogeography. Palaios. 1993; 8: 140–154

46. Frederiksen NO, McIntyre DJ. Palynomorph biostratigraphy of mid(?)-Campanian to upper Maastrichtian strata along the Colville River, North Slope of Alaska: U.S. Geological Survey Open-File Report. 2000; 2000-493: 36

47. Flores RM, Myers MD, Houseknecht DW, Stricker GD, Brizzolara DW, Ryherd TJ, Takahashi KI. Stratigraphy and facies of Upper Cretaceous Schrader Bluff and Prince Creek Formations in the Colville River Bluffs, North Slope, Alaska: U.S. Geological Survey Professional Paper. 2007; 1748: 52

48. Brandlen E. Paleoenvironmental reconstruction of the Late Cretaceous (Maastrichtian) Prince Creek Formation, near the Kikak-Tegoseak dinosaur quarry, North Slope, Alaska: University of Alaska Fairbanks, M.S. thesis. 2008; p. 225.

49. Fiorillo AR, McCarthy PJ, Flaig PP. Taphonomic and sedimentologic interpretations of the dinosaur-bearing Upper Cretaceous strata of the Prince Creek Formation, Northern Alaska: Insights from an ancient high-latitude terrestrial ecosystem. Palaeogeography, Palaeoclimatology, Palaeoecology, 2010; 295:376-388.

50. Conrad JE, McKee EH, Turrin BD. Age of tephra beds at the Ocean Point dinosaur locality, North Slope, Alaska, based on K-Ar and 40Ar/39Ar analyses. US Geological Survey Bulletin, 1992; 1990: 1–12.

51. Flaig PP, McCarthy PJ, Fiorillo AR. Anatomy, evolution, and paleoenvironmental interpretation of an ancient arctic coastal plain: Integrated paleopedology and palynology from the upper Cretaceous (Maastrichtian) Prince Creek Formation, North Slope, Alaska, USA: in Driese, S.G. and Nordt, L.C., eds.: New Frontiers in Paleopedology and Terrestrial Paleoclimatology, Paleosols and Soil Surface Analogue Systems, SEPM Special Publication. , 2013; 114:179–230.

52. Flaig PP, Fiorillo AR, McCarthy PJ, Dinosaur-bearing hyperconcentrated flows of Cretaceous Arctic Alaska: Recurring catastrophic event beds on a distal paleopolar coastal plain: Palaios. 2014; 29: 594-611. doi: 10.2110/palo.2013.133.

53. Flaig PP, Hasiotis ST, Fiorillo AR. A paleopolar dinosaur track site in the Cretaceous (Maastrichtian) Prince Creek Formation of Arctic Alaska: Track characteristics and probable trackmakers: Ichnos. 2018; 25: 208–220. doi: 10.1080/10420940.2017.1337011

54. Flaig PP, McCarthy PJ, Fiorillo AR. A tidally influenced, high-latitude coastal-plain: the Upper Cretaceous (Maastrichtian) Prince Creek Formation, North Slope, Alaska, in Davidson S, Leleu S, North C. eds., From river to rock record: the preservation of fluvial sediments and their subsequent Interpretation: SEPM Special Publication. 2011; 97: 233–264.

55. Salazar-Jaramillo S, McCarthy PJ, Trainor T, Fowell SJ, Fiorillo AR. Origin of clay minerals in alluvial paleosols, Prince Creek Formation, North Slope, Alaska, USA: influence of volcanic ash on pedogenesis in the Late Cretaceous Arctic. Journal of Sedimentary Research. 2015; 85: 192-208.

56. Suarez CA, Flaig PP, Ludvigson GA, Gonzalez LA, Tian R, Zhou H, McCarthy PJ, Van der Kolk DA., Fiorillo AR. Reconstructing the paleohydrology of a Cretaceous Alaskan paleopolar coastal plain from stable isotopes of bivalves. Paleogeography, Palaeoclimatology, Palaeoecology. 2016; 441, 339-351. doi: 10.1016/j.palaeo.2015.07.025.

57. Eberle JJ, Clemens WA, McCarthy PJ, Fiorillo AR, Erickson GM, Druckenmiller PS. Northern-most record of the Metatheria: a new Late Cretaceous

pediomyid from the North Slope of Alaska. Journal of Systematic Palaeontology. 2019; 1–20, doi: 10.1080/14772019.2018.1560369.

58. van der Kolk DA, Flaig PP, Hasiotis ST. Paleoenvironmental reconstruction of a Late Cretaceous, muddy, river-dominated polar deltaic system: Schrader Bluff - Prince Creek Formation transition, Shivugak Bluffs, North Slope of Alaska, U.S.A: Journal of Sedimentary Research. 2015; 85: 903–936. doi: 10.2110/jsr.2015.58.

59. Phillips RL. Depositional environments and processes in Upper Cretaceous nonmarine andmarine sediments, Ocean Point dinosaur locality, North Slope, Alaska. Cretac Res. 2003; 24, 499–523.

60. Currie PJ, Evans DC. Cranial Anatomy of New Specimens of *Saurornitholestes langstoni* (Dinosauria, Theropoda, Dromaeosauridae) from the Dinosaur Park Formation (Campanian) of Alberta. Anat Rec. 2020; doi:10.1002/ar.24241

61. Evans DC, Larson DW, Currie PJ. 2013. A new dromaeosaurid (Dinosauria: Theropoda) with Asian affinities from the latest Cretaceous of North America. Naturwissenschaften. 100 (11): 1041-1049. doi: 10.1007/s00114-013-1107-5

62. Tumarkin-Deratzian AR. Histological evaluation of ontogenetic bone surface texture changes in the frill of *Centrosaurus apertus*. In New Perspectives on Horned Dinosaurs, the Royal Tyrrell Museum Ceratopsian Symp. Bloomington, Indiana University Press. 2010; 251–263.

63. Brown CM, Russell AP, Ryan MJ. Pattern and transition of surficial bone texture of the centrosaurine frill and their ontogenetic and taxonomic implications. J. Vertebr. Paleontol. 2009; 29: 132–141. doi:10.1671/039.029.0119

64. Bennett SC. The ontogeny of Pteranodon and other pterosaurs. Paleobiology. 1993; 19 (1): 92–106.

65. Dal Sasso C, Maganuco S. *Scipionyx samniticus* (Theropoda: Compsognathidae) from the Lower Cretaceous of Italy: Osteology, ontogenetic assessment, phylogeny, soft tissue anatomy, taphonomy, and palaeobiology, Memorie della Società italiana di Scienze naturali e del Museo civico di Storia naturale di Milano, 2011; 34 (1).

66. Göhlich UB, Chiappe LM. A new carnivorous dinosaur from the Late Jurassic Solnhofen archipelago. Nature. 2006; 440 (7082): 329–332. doi:10.1038/nature04579.

67. Chiappe LM, Göhlich, U.B. 2010. Anatomy of Juravenator starki (Theropoda: Coelurosauria) from the Late Jurassic of Germany. Neues Jahrbuch für Geologie und Paläontologie - Abhandlungen. 258 (3): 257–296. doi:10.1127/0077-7749/2010/0125

68. Araújo R, Castanhinha R, Martins R, Mateus O, Hendrickx C, Beckmann F et al. Filling the gaps of dinosaur eggshell phylogeny: Late Jurassic Theropod clutch with embryos from Portugal. Sci Rep. 2013; 3: 1924. https://doi.org/10.1038/srep01924

69. Brusatte SL, Benson RBJ, Hutt S. 2008. The osteology of *Neovenator salerii* (Dinosauria: Theropoda) from the Wealden group (Barremian) of the Isle of Wight. Monograph of the Palaeontographical Society. 2008;162(631):1–166.

70. Xu X. Avian Ancestors - A Review of the Phylogenetic Relationships of the Theropods Unenlagiidae, Microraptoria, *Anchiornis* and Scansoriopterygidae. Ameghiniana. 2013; 50(4), 469.

71. Currie PJ. Cranial anatomy of tyrannosaurid dinosaurs from the Late Cretaceous of Alberta, Canada.Acta Palaeontologica Polonica. 2003; 48 (2): 191–226

72. Brochu CR. Osteology of *Tyrannosaurus rex*: insights from a nearly complete skeleton and high-resolution computed tomographic analysis of the skull. Memoirs of the Society of Vertebrate Paleontology. 2003; 7: 1–138. doi: 10.2307/3889334.

73. Currie PJ. New information on the anatomy and relationships of *Dromaeosaurus albertensis* (Dinosauria: Theropoda) Journal of Vertebrate Paleontology. 1995; 15(3):576-591

74. Senter P. A new look at the phylogeny of Coelurosauria (Dinosauria: Theropoda.) Journal of Systematic Palaeontology. 2007, 5(4): 429-463

75. Choiniere JN, Clark JM, Forster CA, Xu X. A basal coelurosaur (Dinosauria: Theropoda) from the Late Jurassic (Oxfordian) of the Shishugou Formation in Wucaiwan, People's Republic of China, Journal of Vertebrate Paleontology. 2010; 30(6): 1773-1796, doi: 10.1080/02724634.2010.520779

76. Gianechini FA, Makovicky PJ, Apesteguía S. The teeth of the unenlagiine theropod *Buitreraptor* from the Cretaceous of Patagonia, Argentina, and the unusual dentition of the Gondwanan dromaeosaurids. Acta Palaeontologica Polonica. 2011; 56:279-290.

77. Xu X, Wang X-L, Wu X-C. A dromaeosaurid dinosaur with a filamentous integument from the Yixian Formation of China. Nature 1999; 401 (6750): 262–266. doi:10.1038/45769

78. Agnolín FL, Novas FE. Unenlagiid theropods: are they members of the Dromaeosauridae (Theropoda, Maniraptora)? Anais da Academia Brasileira de Ciências. 2011;83(1):117–162. doi: 10.1590/s0001-37652011000100008.

79. Rauhut OWM, Foth C, Tischlinger H. The oldest Archaeopteryx (Theropoda: Avialiae): a new specimen from the Kimmeridgian/Tithonian boundary of Schamhaupten, Bavaria. PeerJ 2018; 6: e4191. doi:10.7717/peerj.4191

80. Turner AH, Pol D, Clarke J, Erickson G, Norell M. A basal dromaeosaurid and size evolution preceding avian flight. Science. 2007; 317:1378–1381.

81. Currie PJ. 1987. Bird-like characteristics of the jaws and teeth of troodontid theropods (Dinosauria, Saurischia). Journal of Vertebrate Paleontology. 1987; 7: 72–81

82. Currie PJ, Rigby JK Jr, Sloan R. Theropod teeth from theJudith River Formation of southern Alberta, Canada; 1990; 107–125 in K. Carpenter and P. J. Currie (eds.), Dinosaur Systematics: Approaches and Perspectives. Cambridge University Press, New York.

83. Averianov AO, Sues H-D. A new troodontid (Dinosauria: Theropoda) from the Cenomanian of Uzbekistan, with a review of troodontid records from the territories of the former Soviet Union. Journal of Vertebrate Paleontology. 2007; 27 (1): 87–98. doi:10.1671/0272-4634(2007)27[87:ANTDTF]2.0.CO;2

84. Lü J, Xu L, Liu Y, Zhang X, Jia S, Ji Q. A new troodontid theropod from the Late Cretaceous of central China, and the radiation of Asian troodontids. Acta Palaeontologica Polonica, 2010; 55:381-388.

85. Xu X, Li F. A new microraptorine specimen (Theropoda: Dromaeosauridae) with a brief comment on the evolution of compound bones in theropods. Vertebrata PalAsiatica, 2016; 54:269-285.

86. Larson DW. Diversity and variation of theropod dinosaur teeth from the uppermost Santonian Milk River Formation (Upper Cretaceous), Alberta: a quantitative method supporting identification of the oldest dinosaur tooth assemblage in Canada. Canadian Journal of Earth Sciences. 2008; 45:1455-1468. doi: 10.1139/e08-070

87. Currie PJ, Dong Z. 2001. New information on *Shanshanosaurus huoyanshanensis*, a juvenile tyrannosaurid (Theropoda, Dinosauria) from the Late Cretaceous of China. Canadian Journal of Earth Sciences. 2991; 38:1729-1737. doi: 10.1139/e01-042

88. Sankey JT. Diversity of Latest Cretaceous (Late Maastrichtian) small theropods and birds: teeth from the Lance and Hell Creek Formations, USA. 2008; p. 117-134. In Sankey, JT, Baszio, S. (eds.), Vertebrate Microfossil Assemblages: Their Role in Paleoecology and Paleobiogeography. Indiana University Press, Bloomington, Indiana.

89. Fiorillo AR. On the occurrence of exceptionally large teeth of Troodon (Dinosauria: Saurischia) from the Late Cretaceous of northern Alaska. PALAIOS. 2008; 23:322–328.

90. Varricchio DJ, Horner JR, Jackson FD. Embryos and eggs for the Cretaceous theropod dinosaur Troodon formosus. Journal of Vertebrate Paleontology, 2002; 22:564-576. Doi: 10.1671/0272-4634(2002)022[0564:eaeftc]2.0.co;2

91. Cau A, Beyrand V, Voeten, D, Fernandez V, Tafforeau P, Stein K, et al. Synchrotron scanning reveals amphibious ecomorphology in a new clade of bird-like dinosaurs. Nature. 2017; 552 (7685): 395–399. doi: 10.1038/nature24679

92. Turner AH, Makovicky PJ, Norell M. A review of dromaeosaurid systematics and paravian phylogeny. Bulletin of the American Museum of Natural History. 2012; 371: 1–206. doi: 10.1206/748.1

93. Novas FE, Pol D, Canale JI, Porfiri JD, Calvo JO. A bizarre Cretaceous theropod dinosaur from Patagonia and the evolution of Gondwanan dromaeosaurids. Proceedings of the Royal Society B: Biological Sciences. 2009; 276:1101-1107. doi: 10.1098/rspb.2008.1554

94. Xu X, Norell MA. A new troodontid dinosaur from China with avian-like sleeping posture. Nature, 2004; 431:838-841. doi: 10.1038/nature02898

95. Makovicky PJ, Norell MA, Clark JM, Rowe T. Osteology and relationships of *Byronosaurus jaffei* (Theropoda: Troodontidae). American Museum Novitates. 2003; 3402:1-32. https://doi.org/10.1206/0003-0082(2003)402%3C0001:oarobj%3E2.0.co;2

96. Tsuihiji T, Barsbold R, Watabe M, Tsogtbaatar K, Chinzorig T, Fujiyama Y, Suzuki S. An exquisitely preserved troodontid theropod with new information on

the palatal structure from the Upper Cretaceous of Mongolia. Naturwissenschaften. 2014; 101:131-142. doi: 10.1007/s00114-014-1143-9

97. Ji Q, Ji S, Lü J, You H, Chen W, Liu Y, Liu Y. First avialian bird from China. Geological Bulletin of China. 2005; 24:197-210.

98. Pei R, Li Q, Meng Q, Norell M, Gao K. 2017. New specimens of *Anchiornis huxleyi* (Theropoda, Paraves) from the late Jurassic of northeastern China. Bulletin of the American Museum of Natural History, 411:1–66. doi: 10.1206/0003-0090-411.1.1

99. Pei R, Norell MA, Barta DE, Bever GS, Pittman M, Xu X. Osteology of a new Late Cretaceous troodontid specimen from Ukhaa Tolgod, Ömnögovi Aimag, Mongolia. American Museum Novitates, 2017; 3889:1–47. doi: 10.1206/3889.1

100. Hu D, Hou L, Zhang L, Xu X. A pre- Archaeopteryx troodontid theropod from China with long feathers on the metatarsus. Nature, 2009; 461:640–643. doi: 10.1038/nature08322

101. Godefroit P, Demuynck H, Dyke G, Hu D, Escuillié F, Claeys P. Reduced plumage and flight ability of a new Jurassic paravian theropod from China. Nature Communications. 2013; 4:1394. doi: 10.1038/ncomms2389

102. Godefroit P, Cau A, Dong-Yu H, Escuillié F, Wenhao W, Dyke G. A Jurassic avialan dinosaur from China resolves the early phylogenetic history of birds. Nature. 2013; 498:359-362. doi: 10.1038/nature12168

103. Martin LD, Stewart JD, Whetstone KN. The origin of birds: structure of the tarsus and teeth. The Auk. 1980; 97:86-93.

104. Elzanowski A, Wellnhofer P. Cranial morphology of Archaeopteryx: evidence from the seventh skeleton. Journal of Vertebrate Paleontology. 1996; 16: 81-94. doi: 10.1080/02724634.1996.10011286

105. Mayr G, Pohl B, Hartman S, Peters DS. The tenth skeletal specimen of *Archaeopteryx*. Zoological Journal of the Linnean Society. 2007; 149:97-116. doi: 10.1111/j.1096-3642.2006.00245.x

106. Rauhut OWM, Werner C. First record of the family Dromaeosauridae (Dinosauria: Theropoda) in the Cretaceous of Gondwana (Wadi Milk Formation, northern Sudan). Paläontologische Zeitschrift. 1995; 69:475-489. doi: 10.1007/BF02987808

107. Sweetman SC. 2004. The first record of velociraptorine dinosaurs (Saurischia, Theropoda) from the Wealden (Early Cretaceous, Barremian) of southern England. Cretaceous Research, 25:353–364. doi: 10.1016/j.cretres.2004.01.004

108. Vullo R, Néraudeau, D, Lenglet T. Dinosaur teeth from the Cenomanian of Charentes, Western France: evidence for a mixed Laurasian-Gondwanan assemblage. Journal of Vertebrate Paleontology. 2007; 27:931-943. doi10.1671/0272-4634(2007)27[931:dtftco]2.0.co;2

109. van der Lubbe T, Richter U, Knötschke N. Velociraptorine dromaeosaurid teeth from the Kimmeridgian (Late Jurassic) of Germany. Acta Palaeontologica Polonica. 2009; 54:401-408. doi: 10.4202/app.2008.0007

110. Norell MA, Clark JM, Turner AH, Makovicky PJ, Barsbold R, Rowe T. A new dromaeosaurid theropod from Ukhaa Tolgod (Ömnögov, Mongolia).

American Museum Novitates. 2006; 3545:1-51. doi: 10.1206/0003-0082(2006)3545[1:andtfu]2.0.co;2

111. Xu X, Tan Q, Sullivan C, Han F, Xiao D. 2011. A short-armed troodontid dinosaur from the Upper Cretaceous of Inner Mongolia and its implications for troodontid evolution. PLoS ONE. 2011; 6:e22916. https://doi.org/10.1371/journal.pone.0022916

112. Norell MA, Makovicky PJ, Bever GS, Balanoff AM, Clark JM, Barsbold R, Rowe T. A review of the Mongolian Cretaceous dinosaur Saurornithoides (Troodontidae: Theropoda). American Museum Novitates. 2009; 3654:1-63. https://doi.org/10.1206/648.1

113. Longrich N. Small theropod teeth from the Lance Formation of Wyoming, USA. 2008; 135-158. In Sankey, J.T. and Baszio, S. (eds.), Vertebrate Microfossil Assemblages: Their Role in Paleoecology and Paleobiogeography. Indiana University Press, Bloomington, Indiana.

114. Holtz TRJ, Brinkman DL, Chandler CL. Denticle morphometrics and a possibly omnivorous feeding habit for the theropod dinosaur *Troodon*. Gaia, 1998; 15:159-166.

115. Vullo R, Néraudeau D. Additional dinosaur teeth from the Cenomanian (Late Cretaceous) of Charentes, southwestern France. Comptes Rendus Palevol. 2010; 9:121-126. doi: 10.1016/j.crpv.2010.03.001

116. Hu D, Clarke JA, Eliason CM, Qiu R, Li Q, Shawkey MD, et al. A bony-crested Jurassic dinosaur with evidence of iridescent plumage highlights complexity in early paravian evolution. Nature Communications. 2018; 9:217. doi: 10.1038/s41467-017-02515-y

117. Currie PJ, Varricchio DJ. A new dromaeosaurid from the Horseshoe Canyon Formation (Upper Cretaceous) of Alberta, Canada. 2004; 112–132. In Currie PJ, Koppelhus EB, Shugar MA and Wright JL. (eds.) Feathered Dragons. Indianapolis: Indiana University Press.

118. Sankey JT. 2001. Late Campanian southern dinosaurs, Aguja Formation, Big Bend, Texas. Journal of Paleontology, 75:208-215. doi: 10.1017/s0022336000031991

119. Burnham DA. New Information on Bambiraptor feinbergi (Theropoda: Dromaeosauridae) from the Late Cretaceous of Montana. 2004; 67-111. In Currie PJ, Koppelhus EB, Shugar MA and Wright JL. (eds.) Feathered Dragons. Indianapolis: Indiana University Press.

120. Xu X, Pittman MD, Sullivan C, Choiniere JN, Tan QW, Clark JM, Norell MA, Wang S. The taxonomic status of the Late Cretaceous dromaeosaurid Linheraptor exquisitus and its implications for dromaeosaurid systematics. Vertebrata PalAsiatica. 2015; 53:29-62.

121. Norell MA, Clark JM, Turner AH, Makovicky PJ, Barsbold R, Rowe T. A new dromaeosaurid theropod from Ukhaa Tolgod (Omnogov, Mongolia). American Museum Novitates. 2006; 3545: 1-51.

122. Kirkland JI, Gaston R, Burge D. A large dromaeosaur (Theropoda) from the Lower Cretaceous of eastern Utah. Hunteria. 1993; 2:1–16.

123. Shen C, Lü J, Liu S, Kundrát M, Brusatte SL, Gao H. A new troodontid dinosaur from the Lower Cretaceous Yixian Formation of Liaoning Province, China. Acta Geologica Sinica-English Edition. 2017; 91:763-780. https://doi.org/10.1111/1755-6724.13307

124. Carpenter K. Baby dinosaurs from the Late Cretaceous Lance and Hell Creek formations and a description of a new species of theropod, in Contributions to Geology, University of Wyoming. 1982; 20(2): 123–134

125. Currie PJ, Dong Z. New information on Cretaceous troodontids (Dinosauria, Theropoda) from the People's Republic of China. Canadian Journal of Earth Sciences. 2001; 38:1753–1766. doi: 10.1139/e01-065

126. Xu X, Wang X-L. A New Dromaeosaur (Dinosauria: Theropoda) from the Early Cretaceous Yixian Formation of Western Liaoning. Vertebrata PalAsiatica. 2004; 42(2): 11–119.

127. Leidy J. Notice of remains of extinct reptiles and fishes, discovered by Dr. Hayden FV in the Bad Lands of the Judith River, Nebraska Territory. PNAS. 1856; 8:72-73.

128. Russell LS. The dentary of *Troödon*, a genus of theropod dinosaurs. Journal of Paleontology. 1948; 22:625-629.

129. Baszio S. Systematic palaeontology of isolated dinosaur teeth from the latest Cretaceous of south Alberta, Canada. Courier Forschungsinstitut Senckenberg. 1997; 196:33-77.

130. Smith JB, Vann DR, Dodson P. 2005. Dental morphology and variation in theropod dinosaurs: Implications for the taxonomic identification of isolated teeth. The Anatomical Record Part A: Discoveries in Molecular, Cellular, and Evolutionary Biology, 285A:699-736. doi: 10.1002/ar.a.20206

131. Zanno LE. A taxonomic and phylogenetic re-evaluation of Therizinosauria (Dinosauria: Maniraptora). Journal of Systematic Palaeontology. 2010; 8:503-543. doi: 10.1080/14772019.2010.488045

132. Rauhut OWM, Foth C, Tischlinger H, Norell MA. Exceptionally preserved juvenile megalosauroid theropod dinosaur with filamentous integument from the Late Jurassic of Germany. Proceedings of the National Academy of Sciences. 2012; 109:11746-11751. doi: 10.1073/pnas.1203238109

133. Choiniere JN, Clark JM, Forster CA, Norell MA, Eberth DA, Erickson GM, et al. A juvenile specimen of a new coelurosaur (Dinosauria: Theropoda) from the Middle-Late Jurassic Shishugou Formation of Xinjiang, People's Republic of China. Journal of Systematic Palaeontology. 2014; 12:177-215. doi: 10.1080/14772019.2013.781067

134. Tsuihiji T, Watabe M, Tsogtbaatar K, Tsubamoto T, Barsbold R, Suzuki S et al. Cranial osteology of a juvenile specimen of *Tarbosaurus bataar* (Theropoda, Tyrannosauridae) from the Nemegt Formation (Upper Cretaceous) of Bugin Tsav, Mongolia. Journal of Vertebrate Paleontology. 2011; 31:497-517. https://doi.org/10.1080/02724634.2011.557116

135. Carr TD, Williamson TE. Diversity of late Maastrichtian Tyrannosauridae (Dinosauria: Theropoda) from western North America. Zoological Journal of the Linnean Society. 2004; 142:479-523. doi: 10.1111/j.1096-3642.2004.00130.x

136. Han G, Chiappe LM, Ji S-A, Habib M, Turner AH, Chinsamy A et al. 2014. A new raptorial dinosaur with exceptionally long feathering provides insights into dromaeosaurid flight performance. Nature Communications, 5:1-9. doi: 10.1038/ncomms5382

137. Xu X, Wu X-C. Cranial morphology of *Sinornithosaurus millenii* Xu et al. 1999 (Dinosauria: Theropoda: Dromaeosauridae) from the Yixian Formation of Liaoning, China. Canadian Journal of Earth Sciences. 2001; 38:1739-1752. doi: 10.1139/e01-082

138. Zheng X, Xu X, You H, Zhao Q, Dong Z. A short-armed dromaeosaurid from the Jehol Group of China with implications for early dromaeosaurid evolution. Proceedings of the Royal Society B: Biological Sciences. 2010; 277:211-217. doi: 10.1098/rspb.2009.1178.

139. Perle A, Norell M, Clark JM. A new maniraptoran theropod, *Achillobator giganticus* (Dromaeosauridae), from the Upper Cretaceous of Burkhant, Mongolia. Contributions from the Geology and Mineralogy Chair, National Museum of Mongolia. 1999; 101:1-105.

140. DePalma RA, Burnham DA, Martin LD, Larson PL, Bakker RT. The first giant raptor (Theropoda: Dromaeosauridae) from the Hell Creek Formation. Paleontological Contributions, 2015; 1-16. doi: 10.17161/paleo.1808.18764

141. Ostrom JH. Osteology of *Deinonychus antirrhopus*, an unusual theropod from the Lower Cretaceous of Montana. Bulletin Peabody Museum of Natural History: Peabody Museum of Natural History, Yale University. 1969; 30:1-165.

142. Barsbold R, Osmólska H. The skull of *Velociraptor* (Theropoda) from the Late Cretaceous of Mongolia. Acta Palaeontologica Polonica. 1999; 44:189–219.

143. Milner AC. Theropod dinosaurs of the Purbeck limestone group, Southern England. Special Papers in Palaeontology. 2002; 68:191-202.

144. Allain R, Taquet P. A new genus of Dromaeosauridae (Dinosauria, Theropoda) from the Upper Cretaceous of France. Journal of Vertebrate Paleontology. 2000; 20:404-407. doi: 10.1671/0272-4634(2000)020[0404:angodd]2.0.co;2

145. Barsbold R. Saurornithoididae, a new family of small theropod dinosaurs from central Asia and North America. Palaeontologia Polonica, 1974; 30:5–22.

146. Samman T, Powell GL, Currie PJ, Hills LV. Morphometry of the teeth of western North American tyrannosaurids and its applicability to quantitative classification. Acta Palaeontologica Polonica. 2005; 50: 757–776.

147. Buckley LG, Larson DW, Reichel M, Samman T. Quantifying tooth variation within a single population of *Albertosaurus sarcophagus* (Theropoda: Tyrannosauridae) and implications for identifying isolated teeth of tyrannosaurids. Canadian Journal of Earth Science. 2010; 47: 1227–1251.

148. Russell DA. The environments of Canadian dinosaurs. Canadian Geographical Journal. 1973; 87: 4–11.

149. Paul GS. Migration. In The Encyclopedia of Dinosaurs, Edited by: Currie P, Padian K. 1997; 444–446. San Diego, CA: Academic Press

150. Bell PR, Snively E. 2008. Polar dinosaurs on parade: a review of dinosaur migration, Alcheringa: An Australasian Journal of Palaeontology, 2008; 32(3): 271–284, doi: 10.1080/03115510802096101

151. Gangloff RA, Fiorillo AR. Taphonomy and paleoecology of a bonebed from the Prince Creek Formation, North Slope, Alaska. Palaios. 2010; 25: 299–317.

152. Suarez CA, Ludvigson GA, Gonzalez LA, Fiorillo AR, Flaig PP, McCarthy PJ. 2013. Use of multiple isotope proxies for elucidating Arctic Cretaceous paleohydrology. In Bojar AV, Melinte-Dobrinescu MC, Smit J. (eds.). Isotopic Studies in Cretaceous Research: Geological Society of London Special Publication. 2013; 382:185–202.

153. Avgar T, Street G, Fryxell JM, On the adaptive benefits of mammal migration. Canadian Journal of Zoology. 2014; 92(6): 481-490.

154. Guillet A. Distribution and conservation of the shoebill (*Balaeniceps rex*) in the southern Sudan. Biological Conservation. 1978; 13: 39-49.

155. Curry-Lindahl K. Bird Migration in Africa: Movements between Six Continents. 1981; 2. New York: Academic Press

156. Fiorillo AR. The dinosaurs of arctic Alaska. Sci Am. 2004; 291: 84–91.

157. Horner JR, Makela R. Nest of juveniles provides evidence of family structure among dinosaurs. Nature. 1979; 282(5736):296-298.

158. Horner JR. Evidence of colonial nesting and 'site fidelity' among ornithischian dinosaurs. Nature. 1982; 297(5868):675-676.

159. Norell MA, Clark JM, Chiappe LM, Dashzeveg D. A nesting dinosaur. Nature. 1995; 378:774-776.

160. Tanaka K, Zelenitsky DK, Therrien F, Kobayashi Y. Nest substrate reflects incubation style in extant archosaurs with implications for dinosaur nesting habits. Sci Rep. 2018; 8, 3170. doi: 10.1038/s41598-018-21386-x

161. Varricchio DJ. Reproduction and Parenting in Paul, G.S. (ed.). The Scientific American Book of Dinosaurs. New York: St. Martin's Press. 2000; 279-293.

162. Mateo JA, Lopez- Jurado LF. Dental ontogeny in *Lacerta lepida* (Sauria, Lacertidae) and its relationship to diet. Copeia 1997; 461–463.

163. Townsend V, Akin J, Felgenhauer B, Dauphine J, Kidder S. Dentition of the Ground Skink, *Scincella lateralis* (Sauria, Scincidae). Copeia, 1999; (3): 783–788. doi:10.2307/1447615

164. Fiorillo AR, Currie PJ. Theropod teeth from the Judith River Formation (Upper Cretaceous) of south-central Montana. Journal of Vertebrate Paleontology. 1994; 14 (1): 74–80. doi: 10.1080/02724634.1994.10011539

165. Fowler DW, Freedman EA, Scannella JB, Kambic RE. The Predatory ecology of *Deinonychus* and the origin of flapping in birds. PLoS ONE. 2011; 6(12): e28964. doi: 10.1371/journal.pone.0028964

166. Torices A, Wilkinson R, Arbour VM, Ruiz-Omeñaca JI, Currie PJ. Puncture-and-pull biomechanics in the teeth of predatory coelurosaurian dinosaurs. Current Biology. 2018; 28:1467-1474.e2. doi: 10.1016/j.cub.2018.03.042

Figure captions

Figure 1. Locality map (A) of Pediomys point (red star) in the North Slope of Alaska, USA. Coordinates: N 70.018667°, W 151.591488°. Stratigraphic sections schematised in Fig 2 are reported here in B, with PdP representing the fossil bone-bearing section.

Figure 2. Geological setting. Stratigraphic sections at Pediomys point. Black star represents DMNH 21183.

Figure 3. DMNH 21183. Anterior portion of a dromaeosaurid dentary in lateral (A), medial (B), dorsal view (C) views and close up of the interdental plates and 3rd tooth in medial view (D). Curved arrows represent features hidden on that view. Abbreviations: a2, 2nd alveolus; a3. 3rd alveolus; d2, 2nd tooth; d3, 3rd tooth; d4, 4th tooth; ave, antero-ventral process; idp, interdental plate; lr, lateral ridge; mg, Meckelian groove; mf, Meckelian foramen. Scale bar: 2 mm.

Figure 4. Closeup of the morphological details in DMNH 21183. Detail of the 2nd tooth highlighting the distal carina under normal light (A) and fluorescent microscopy (B). Details of the denticles (C) and closeup of the lateral

ridge (lr) close to the alveolar margin in lateral view (D). Abbreviation: lr, lateral ridge. Scale bar: 0.5 mm.

Figure 5. Denticle size index comparison. Box plot showing a comparison of the relative denticle size index (DSDI) between DMNH 21183 and four clades of deinonychosaurian theropods. DMNH 21183 is marked as a star. Dots represent outlier of each clade's distribution.

Figure 6. Position of DMNH 21183 in the theropod teeth morphospace. Principal components analysis of theropod teeth morphospace based on the dataset from Larson and Currie [30]. DMNH 21183 is marked as a blue star. Abbreviations: fore-aft basal length (FABL), crown height (CH), basal width (BW), anterior (ADM) and posterior denticles per millimetre (PDM). Morphometric dataset prepared as explained in methods and including measurements from DMNH 21183 reported in Supplementary Information (Data S1).

Table 1. Measurements of DMNH 21183

Table 2. Coefficients of the PCA analysis run on the theropod teeth dataset published by Larson and Currie [30].

Supporting information

S1 Fig. Magnified close-up views of the 2nd tooth in DMNH 21183. Close-up of the mesial carina in anterior view (A, C), highlighting the the denticle-bearing anterior carina (ac). Magnified lingual view of the tooth (B) highlighting the anterior (ac) and posterior (pc) carinae (D). Dotted line (C) highlights the interdenticular sulci. Scale bar: 100 µm.

S1 Table. Systematics used in this study. Systematics names and relative phylogenetic definition used in this study.

Figure 1 Click here to download Figure Figure 1.tif

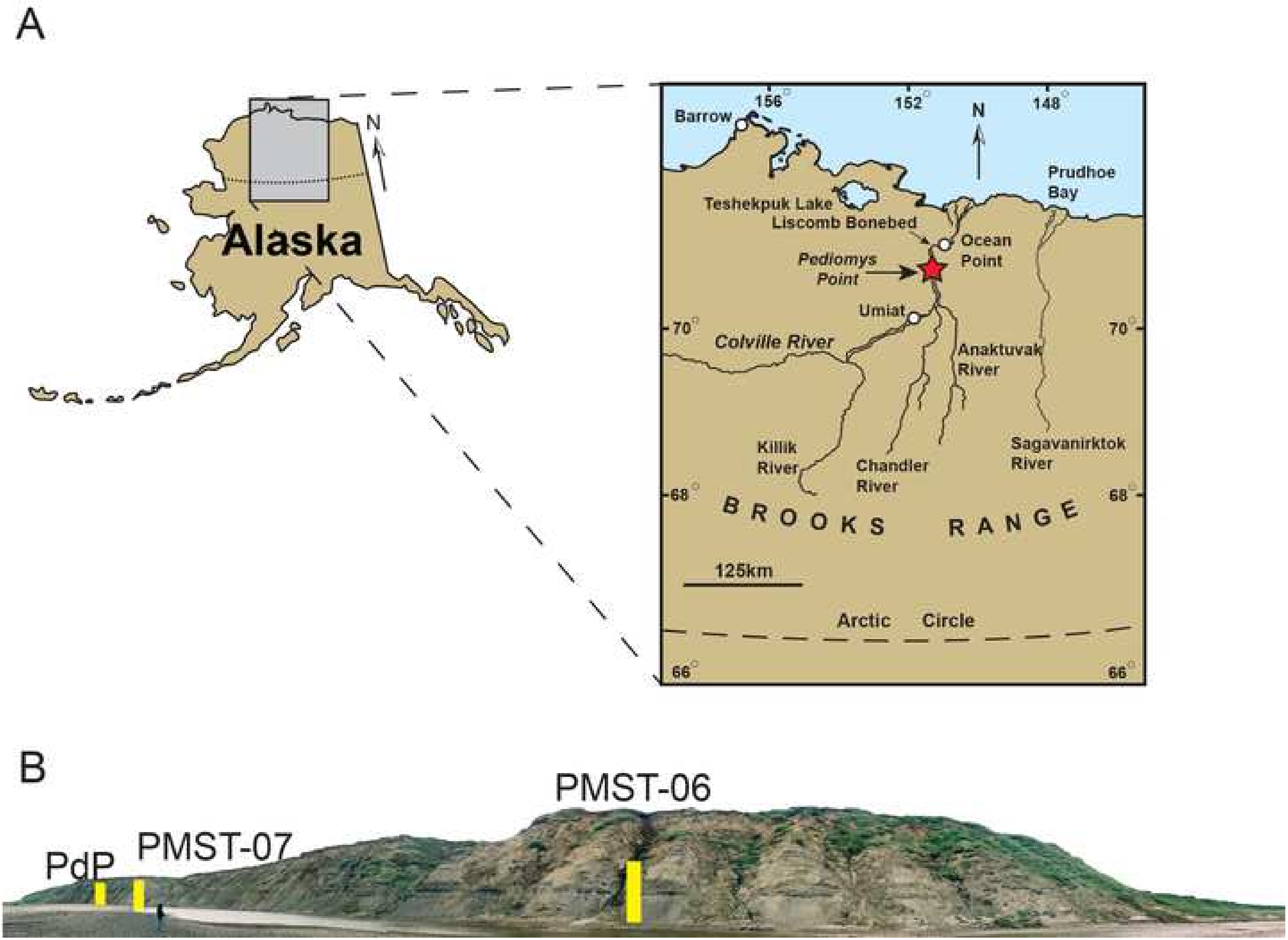

Figure 2 Click here to download Figure Figure 2.tif

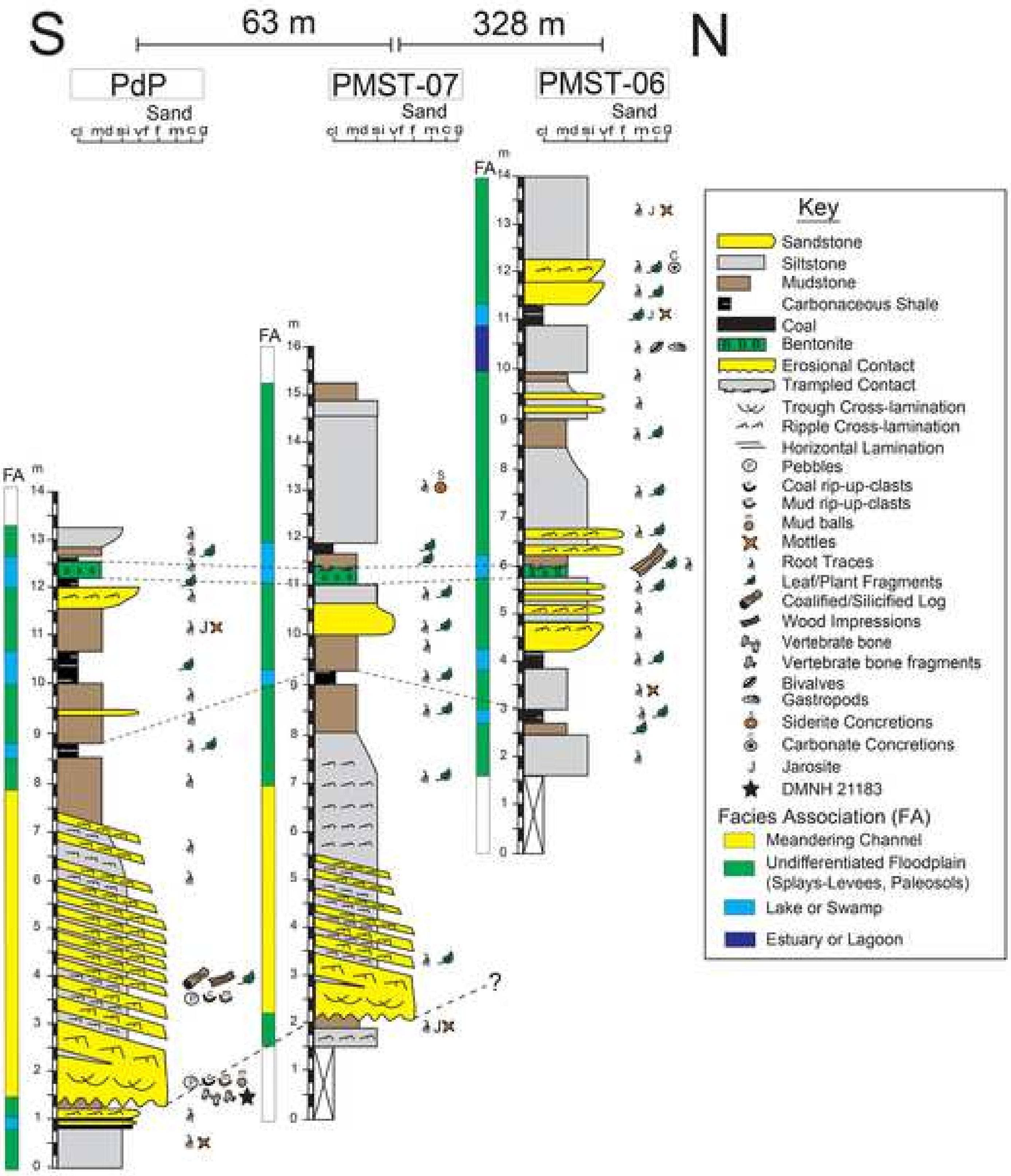

Figure 3

Click here to download Figure Figure 3.tif

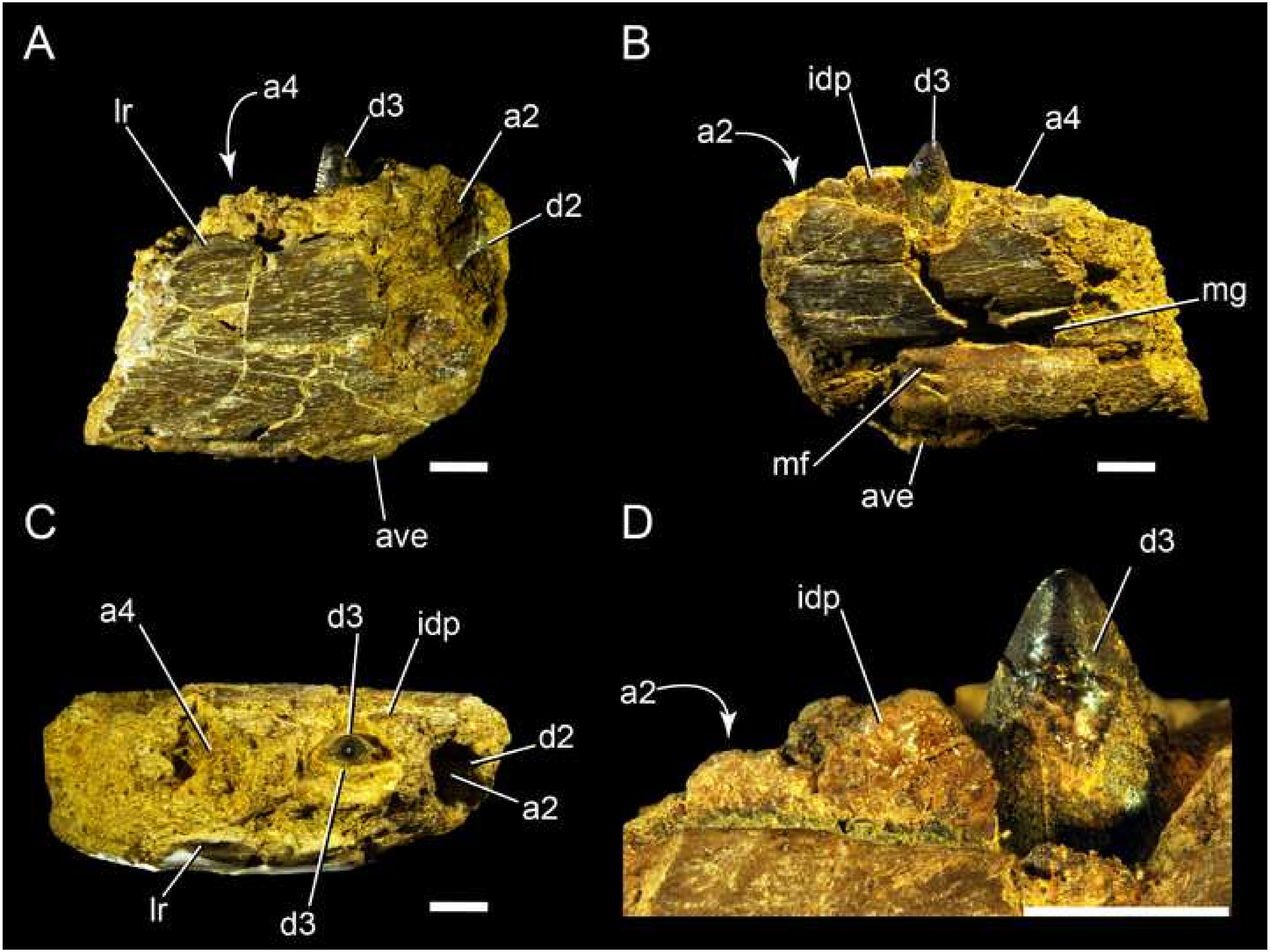

Figure 4

Click here to download Figure Figure 4.tif

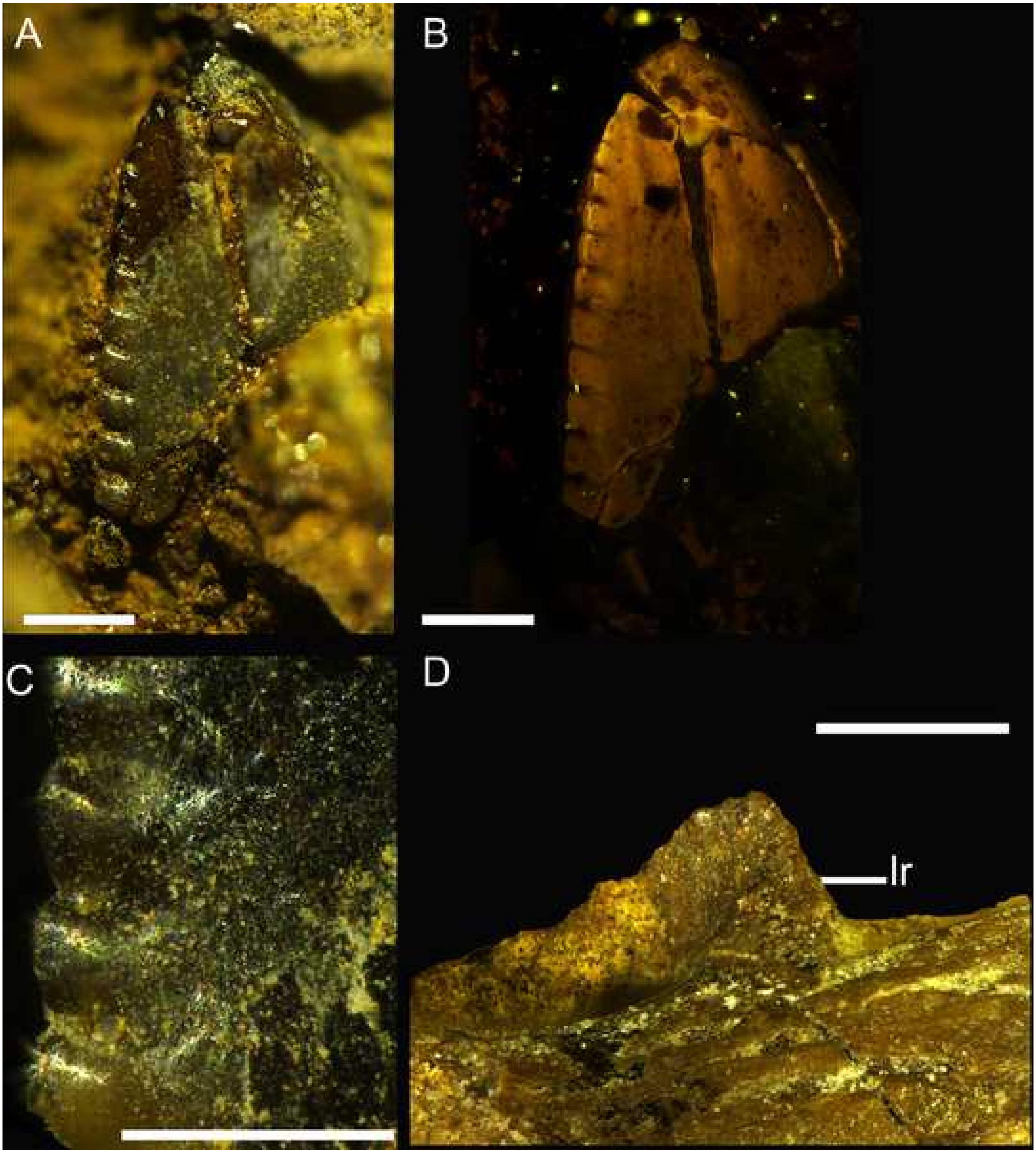

Figure 5

Click here to download Figure Figure 5.tif

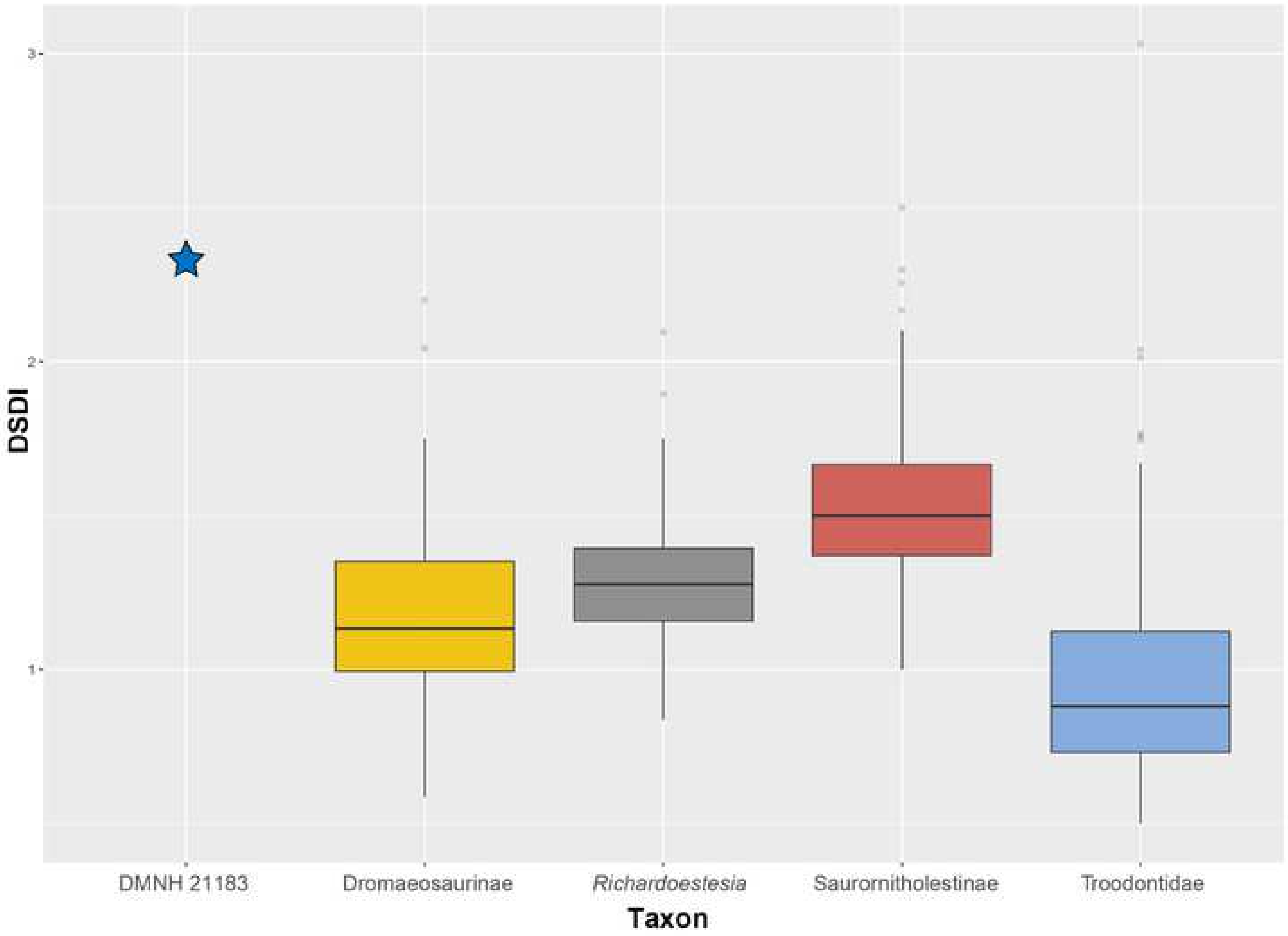

Figure 6

Click here to download Figure Figure 6.tif

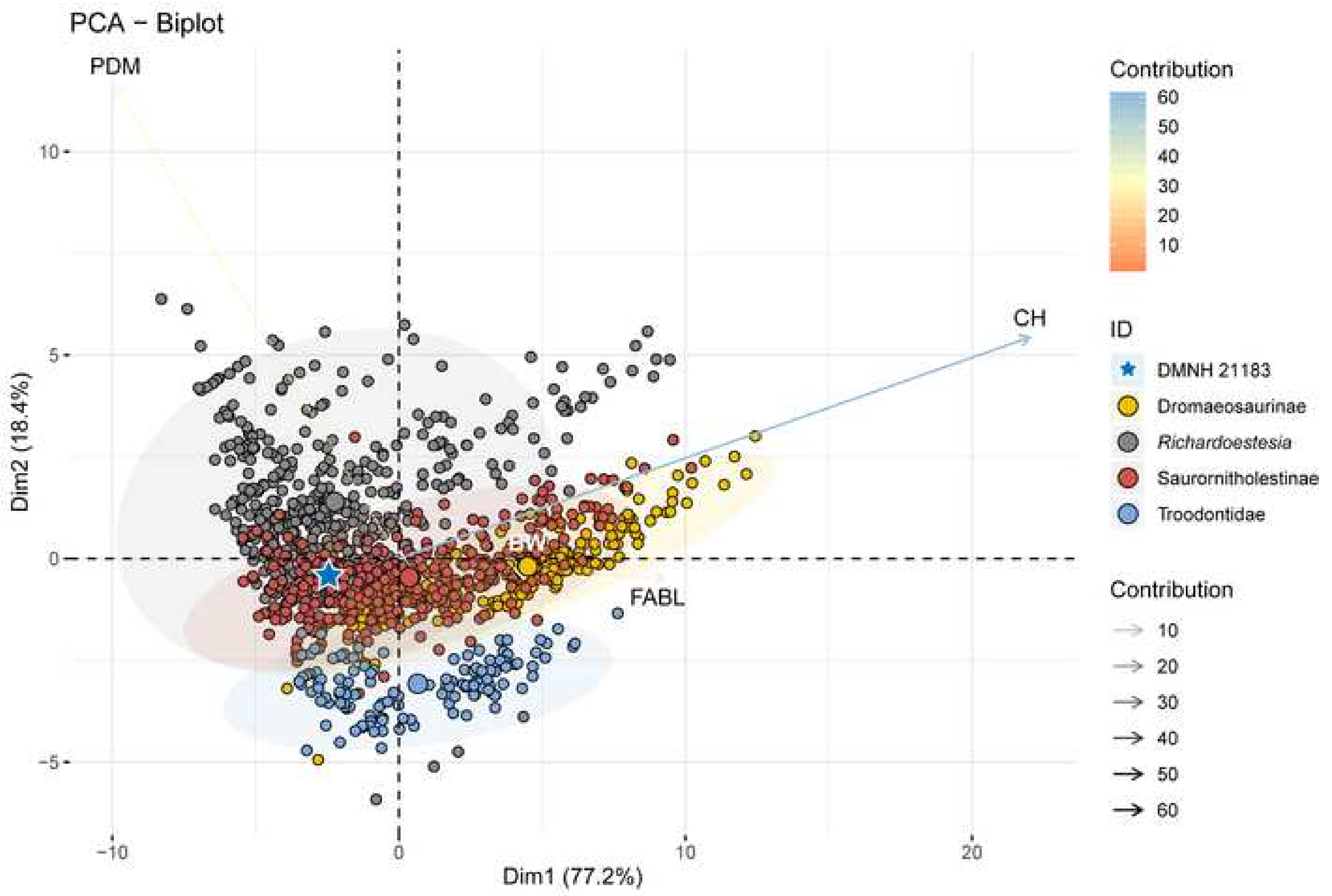

Morphometric dataset for PCA

Click here to access/download
Supporting Information
Dataset S1.xlsx

Supplementary figure for magnified details of denticles-wearing carinae

Click here to access/download
Supporting Information
Figure S1.tif

Supplementary table 1 with systematic definitions used in this study

Click here to access/download
Supporting Information
Table S1. Systematic table.xlsx
