# Supplementary material for: The first juvenile dromaeosaurid (Dinosauria: Theropoda) from Arctic Alaska
Source: PLoS One. 2020 Jul 8;15(7):e0235078. doi: 10.1371/journal.pone.0235078 (PMC7343144; doi:10.1371/journal.pone.0235078)
Supplement: Attachment — Submitted filename: PONE-D-20-05495_R1_reviewer_CH.pdf [file pone.0235078.s014.pdf]

PLOS ONE

The first juvenile dromaeosaurid (Dinosauria: Theropoda) from Arctic Alaska

--Manuscript Draft--

**Manuscript Number:**	PONE-D-20-05495R1
**Article Type:**	Research Article
**Full Title:**	The first juvenile dromaeosaurid (Dinosauria: Theropoda) from Arctic Alaska
**Short Title:**	A juvenile Saurornitholestinae theropod from Alaska
**Corresponding Author:**	Alfio Alessandro Chiarenza<br>Perot Museum of Nature and Science<br>Dallas, Texas UNITED STATES
**Keywords:**	Theropoda; Cretaceous; Alaska; Dromaeosauridae; Paleobiology; Arctic; Dinosaurs; Dinosauria; Saurornitholestinae; paleobiogeography; Paleodiversity; North America
**Abstract:**	Compared to the osteological record of herbivorous dinosaurs from the Late Cretaceous Prince Creek Formation of northern Alaska, there are relatively fewer remains of theropods. The theropod record from this unit is mostly comprised of isolated teeth, and the only non-dental remains known can be attributed to the troodontid cf. Troodon and the tyrannosaurid Nanuqsaurus . Thus far, the presence of members of Dromaeosauridae has been limited to isolated teeth. Here we describe a symphyseal portion of a small dentary with two ziphodont teeth. Based on tooth shape, denticle morphology, and the position of the Meckelian groove, we attribute this partial dentary to a saurornitholestine dromaeosaurid. The fibrous bone surface, small size, and higher number of mesial denticles compared to distal ones point to a juvenile growth stage for this individual. Multivariate comparison of theropod teeth morphospace by means of principal component analysis reveals an overlap between this dentary and Saurornitholestinae dromaeosaurid morphospace, a result supported by phylogenetic analyses. This is the first confirmed non-dental fossil specimen from a member of Dromaeosauridae in the Arctic, expanding on the role of Beringia as a dispersal route for this clade between Asia and North America. Furthermore, the juvenile nature of this individual adds to a growing body of data that suggests Cretaceous Arctic dinosaurs of Alaska did not undergo long-distance migration, but rather they were year-round residents of these paleopolar latitudes.
**Order of Authors:**	Alfio Alessandro Chiarenza
	Anthony R. Fiorillo
	Ronald S. Tykoski
	Paul J. McCarthy
	Peter P. Flaig
	Dori L. Contreras
**Response to Reviewers:**	Dear Editor,<br>Thanks for editing our manuscript entitled “The first juvenile dromaeosaurid (Dinosauria: Theropoda) from Arctic Alaska”. Attached to this letter we provide a revised version of the Manuscript with updated figures and Supplementary material. Given the comments from the reviewers, we accommodated their requests by: 1) adding a phylogenetic systematics section; 2) expanding the morphometric dataset with a more inclusive group of theropod taxa for additional analysis, adding also discriminant function analyses to the pool of analytical tools employed in the study; 3) streamling and partially reworking the discussion, broadening the section with more paleoecological and biogeographic insights but focusing the section. We added an additional author, Dr. Dori Contreras to the team behind the study: other than offering additional insights throughout the manuscript and assisting with the phylogenetic analysis, she provided important paleobotanical information for the new Figure 10. We strongly appreciated the input from the reviewers, which greatly improved the quality of this manuscript. All figures in this paper have been firsthand produced by our authorial team, including the map and panoramic view in Figure 1 which has not been produced

Powered by Editorial Manager® and ProduXion Manager® from Aries Systems Corporation

with the aid of any external software (e.g. Google Maps) but has been photographed and assembled by one of our coauthors (Paul J. McCarthy).The artistic reconstruction in Figure 10 was commissioned by our team to scientific illustrator Andrey Atuchin, hence we own the rights to the illustration.
Below is outlined a point by point response to both Reviewers. Whereas their text has been highlighted in italics, our response has been written in bold (see attached word document for this formating).

Best Regards,

Alfio Alessandro Chiarenza
On behalf of all coauthors.

5. Review Comments to the Author

Please use the space provided to explain your answers to the questions above. You may also include additional comments for the author, including concerns about dual publication, research ethics, or publication ethics. (Please upload your review as an attachment if it exceeds 20,000 characters)

Reviewer #1: In their manuscript entitled “The first juvenile dromaeosaurid (Dinosauria: Theropoda) from Arctic Alaska”, Chiarenza and colleagues report, describe and identify the anterior portion of a dentary (DMNH 21183) from the Early Maastrichtian Prince Creek Formation of northern Alaska. The specimen is referred to a juvenile saurornitholestine dromaeosaurid based on comparative anatomy and a morphometric analysis using the best preserved tooth (d3), an identification I agree with. This paper is well-written, well-organized and most of the figures are very nice. The descriptive part is thorough and the introduction, geological settings and discussion section are sound and detailed. The methodology used to identify the material could certainly be improved though, and I have some problems with the way the text discussing the phylogenetic distribution of the material based on dental features is written. If I believe that this contribution only provides a limited amount of information for our knowledge on theropods/paravian paleogeography and palaeoecology, I would nonetheless suggest to accept Chiarenza et al.’s work with moderate revisions, urging the authors to take into consideration my main recommendations:

- My main problem with this paper lies in the author’s method to identify the material. First, I wonder why they did not perform a cladistic analysis using the most recent datamatrix on coelurosaurs/paravian relationships. I understand that their material is very limited but wonder if it does not display enough apomorphic features to help resolve its phylogenetic affinity. At least, the authors should specify why they do not want to conduct a cladistic analysis to explore the phylogenetic distribution of DMNH 21183. Second, they decide to perform a PCA based on crown-measurements of d3 to help in the identification of the material. Yet, I believe there are more appropriate methods to identify theropod teeth such as discriminant and cluster analyses using a dataset of crown-based measurements, or (even better according to me) a cladistic analysis using a dentition-based datamatrix and a fully constrained topological tree (see my paper on the Aerosteon tooth and the dentition of Abelisauridae recently published in Cretaceous Research on how to perform those analyses). That being said, the major problem I see is that the best preserved tooth of this small portion of mandible, d3, is a partially erupted tooth and I wonder how the authors managed to accurately take measurements of FABL, CH and BW on this crown. In addition, it does not make much sense to me to use a dataset restricted to paravians and to omit specimens from which the mesial carina does not include denticles. I would therefore recommend the authors to perform a first analysis with all taxa and measurements from Larson & Currie’s dataset, then use a more restrictive one based on relevant arguments on why tyrannosauroids, for instance, should be excluded. I would also strongly advise them to perform discriminant and cluster analyses on the dataset of crown-based measurements and, ideally, a cladistic analysis using my most recent dentition-based datamatrix (from the Aerosteon paper), coding their dental material as coming from the dentary and the mesial dentition. I am quite confident that the result of the cladistic analysis would further support a saurornitholestine affinity.

Powered by Editorial Manager® and ProduXion Manager® from Aries Systems Corporation

	We appreciate the positive comments from the Reviewer. We decided to largely follow his recommendation in proceeding with a set of phylogenetic tests before a quantitative-morphometric analysis of the material. In this resubmission we first included DMNH 21183 in the phylogenetic dataset from Lee et al. (2014), a dataset with a wide taxonomic sampling but also with a large pool of dentary characters to maximize the scorings for our fragmentary specimen. We then included the dental coding from the specimen in the dentition-only and crown-tooth-only data matrices from Hendrickx et al. (2020), an additional set of analyses which strengthen our previous interpretation based on comparative anatomy and morphometrics. We also embraced the recommendations related to morphometric by first performing multivariate analyses on the teeth morphometric dataset by Gerke and Wings (2016) and then moving on the less inclusive Larson and Currie (2016). We want to point out that the choice of Gerke and Wings (2016) was based on the need for a purely morphometric (that is quantitative) dataset to compare our specimen, as we wanted to keep consistency with the measurements from Larson and Currie (2013) but also wanted to have an independent test which could exclude any qualitative observations (making it fully independent from the comparative and phylogenetic results). We strongly acknowledge the reviewer in his suggestions, as these additional analyses not only strengthened our interpretation, but also provided scope for expanded discussion on this specimen.<br><br>- In the methodology section, the authors should specify the result(s) of the cladistic analysis of what publications they follow when commenting on paravian phylogeny and the phylogenetic position of the different dromaeosaurids. Likewise, they should add the fact that it is the third dentary tooth of DMNH 21183 that is included in the PCA.<br>We thank the Reviewer for this suggestion, this is now been included in the Material and Methods section.<br><br>- In the chapter on morphological remarks (which I would suggest to rename “Phylogenetic position of DMNH 21183”), in the section in which the authors discuss the distribution of dental features displayed by DMNH 21183, they obviously use my recent paper on the distribution of dental features in theropods (Palaeontologica Electronica) as a main reference. I thank them for that but, if my paper is often cited, it frustrates me to see that some parts of the text directly comes from my paper without citing it. In many parts of the text, the authors just copied and pasted portions of my PE publication, using the exact same citations and specimen numbers, while I highly doubt that they have personally examined the material I have, or looked at the references the way I have. For fairness (and because sometimes it almost looks like plagiarism), I would really like the authors to rephrase the text that directly comes from my paper using the later as the main bibliographical source, and remove all specimens (those coming directly from my paper) that they did not examine themselves. In this section of the MS, the DSDI for crowns having mesial and distal roughly of the same size is also close to 1 and not lower than 0.9.<br><br>We thank the reviewer for these suggestions: we proceeded in renaming the section, paraphrasing further the content referring to his work, adding additional citations to them and reporting all of his edits (e.g. related to the DSDI of some taxa). We hope that this resubmission fully reflects the credit due to his scientific production.<br><br>- I don’t have any comments to make regarding the Introduction, Geological setting and Depositional Environments, and Discussion sections, which really seem to be sound and well-written. I would only recommend to move the part of the text that discuss the juvenile features displayed by the specimen, present in the description section, to the Results or Discussion sections (I always prefer discussing the affinities of a specimen and its ontogenetic stage in the Discussion section, personally).<br>Thanks for the appreciative comments. We also agreed with the recommendation regarding the rearrangement of the text and proceeded doing so in this resubmission.<br><br>- Regarding figures, I would only suggest the authors to make the convex hull and the arrows of the variables used in the PCA better visible to the reader, in figure 6. Many taxa must also be italicized, while the capital letters of some titles of articles should be removed, in the bibliography section.<br><br>Thanks for these observations, we modified these figures and followed the same for

the new ones added to this resubmission.

I provided additional corrections, remarks and suggestion in the pdf version of their MS, and ask the authors to take them into consideration.
We thank the reviewer for these thorough edits. We accepted and modified all relevant portions of the main manuscript text accordingly.

Kind regards,

Christophe Hendrickx, San Miguel de Tucumán, the 14th of March 2020.

Reviewer #2: March 30, 2020

Dear Editor,

I submit to you my review of PONE-D-20-05495, “The first juvenile dromaeosaurid (Dinosauria: Theropoda) from Arctic Alaska” by Chiarenza et al. The authors report on the first skeletal material of a dromaeosaurid from the Arctic, which is an important data point; fortunately, the specimen, although fragmentary, has some important features that enables comparisons with other taxa. I think that the article is publishable with minor revisions. Most of the fixes center on writing style.

In terms of major concerns, I find the second paragraph of the Discussion to be somewhat convoluted and difficult to follow; I think it can be redrafted into a more straightforward style so that the main points aren’t lost to the reader.

The third paragraph makes the claim that the Arctic saurornitholestines were nonmigratory; in my view, the evidence is mute on that point and the authors should present a more critical assessment of such claims; for example, what evidence is required to support the claim? Is that evidence available based on the fossil at hand, really?

Finally, the fourth paragraph continues the argument, but from what strikes me as – at best - tangential lines of evidence. Again, I encourage the authors to re-visit this section with a critical bent of mind. Other comments on the discussion appear in the specific comments below.

Unless I missed it, there is one significant gap in the discussion, and that is comparison with the dromaeosaurid fauna of the Horseshoe Canyon Formation of Alberta, which is equivalent in geological age to the PCF. I suggest that the authors make an explicit comparison with the HCF dromaeosaurids to arrive at a snapshot of the distribution of the clade at that time in the northern half of Laurasia. I think that is the opportunity that will lock in future citations of the article, once it is published.

Finally, the authors identify a feature (lateral ridge of the dentary) in the new fossil that is also only seen elsewhere in the Gondwanan Buitreraptor, but later in the ms, they consider the fossil referable to Saurornitholestinae based on a morphometric analysis of teeth. I ask that the authors acknowledge these conflicting hypotheses of identity and to explicitly provide their rationale for preferring one hypothesis over the other, if that is what they decide, in the end, to do.

My statistical experience does not extend to PCA analyses, so I am unable to comment on that part of their work.

The authors may know my identity: Thomas D. Carr

We would thank the Reviewer for his suggestions. We proceeded in this resubmission with a double check in writing style and grammar. Given also the recommendations from Reviewer 1, we decided to expand the study with additional phylogenetic and morphometric analyses. As a consequence, the Discussion section has been now partially reworded and reorganized, hoping to make its style more readable by the audience. We appreciate the suggestion to expand our discussion on paleoecology and biogeography of other contemporary dromaeosaurids. We did so not only by expanding the relevant discussion paragraphs, but also reporting some additional data

Powered by Editorial Manager® and ProduXion Manager® from Aries Systems Corporation

(Dataset S3) to compare more easily the temporal and geographic distribution of North American dromaeosaurids during the latest Cretaceous. Regarding the anatomical observations, after we expanded our search because of the suggestions from Reviewer 1, we found that the structure we previously considered a potential synapomorphy between Buitreraptor and DMNH 21183 as to be more widespread in Dromaeosauridae. In particular we noticed the presence of similar lateral ridges in many other dentary rims of dromaeosaurids (e.g. Velociraptor AMNH 6515 right side, Tsaagan IGM 100/1015 right side) which are partially emphasized by damaging of the bone surface. We also want to remark that the absence of denticulation in Unenlaginae (as reported in the former draft of the paper) already excludes the identification of DMNH 21183 as a closer taxon to, for example Buitreraptor, than to more derived dromaeosaurids (e.g. Eudromaeosauria). We then modified the text accordingly to reflect these observations. We also added more osteological information that popped up while scoring the specimen for phylogenetic analyses, strengthening our previous interpretation based on comparative anatomy. We are also sure that the reviewer will appreciate the efforts in providing a suite of phylogenetic tests to our hypotheses, supporting our previous interpretation related to the systematic position of this Alaskan theropod.
Lastly we expanded the section related to the paleoecology of Arctic dromaeosaurs which the reviewer previously considered speculative. We provided more context based particularly on comparison with modern theropods' migratory habits in the Arctic region. We also added information from the literature on growth rates and other indicative physiological observations that would back up our interpretation that these Arctic theropods indeed would spend their full solar year in the Arctic without migrating to southern latitudes. We removed, as the Reviewer recommended, some more speculative ecomorphological content, in order to simplify and focus further our Discussion section. We furthermore appreciated the effort in proof-reading our manuscript, and provided below a point by point check of the edits added to this resubmission.
Specific comments:
Line 17: delete "the"; taxonomic names are formal names and so are not preceded by "the."
Done

Line 19: replace "diminutive" with "small"; avoid jargon.

Done.
Line 20: delete "morphological", replace "based on" with "with."
Done

Line 23: replace "status" with "growth stage"; as far as I can tell relative maturity isn't a status. Replace "exploration" with "comparison."
Done

Line 62: Replace "taxonomical" with "taxonomic."
Done

Line 65: delete "purported", unless you are doubtful of its uniqueness.
Done

Line 95: replace "like" with "such as."
Done

Line 104: replace "examined" with "studied first hand" and modify the sentence accordingly.

Edit done and sentence rephrased to: "The subclades of theropods included in this dataset, apart from specimen studied first hand (DMNH 21183) are:"
Line 106: replace "performed" with "did."
Done

Line 107: insert a comma after "measurements"; find an accurate replacement for "distilling".
Replaced with 'converting"

Powered by Editorial Manager® and ProduXion Manager® from Aries Systems Corporation

Line 108: replace “representing” with “that represents”; delete “to be.”
Done

Line 110: replace “estimate…of” with “compare”; replace “within” with “with.”
Done.

Line 208: is it “foramen” or “foramina”; the figure labels only one foramen. Please clarify.
Thanks for spotting this, it is foramina: clarified and edited in the relevant figure caption (Fig. 3)

Line 211: is there any evidence for the first alveolus? How do you justify the identification of the first preserved tooth as the second tooth in sequence? Clarify.

We reported in the description this explanation, following direct comparison with the condition in both Acheroraptor (Evans and Larson, 2013) and Saurornitholestes (Currie and Evans, 2020): “Given the position of the most erupted tooth (d3; Fig 3¬¬¬¬) above the Meckelian foramina (on the medial surface of the dentary, Fig 3B), and above an anteroventral process of the dentary (ave; Figs 3A and 3B), we identify this as the 3rd tooth in the dentary [e.g. 60, 61], with the anteriorly positioned, less erupted tooth (d2) being identified as the 2nd.”

Refs:

Currie PJ, Evans DC. Cranial Anatomy of New Specimens of Saurornitholestes langstoni (Dinosauria, Theropoda, Dromaeosauridae) from the Dinosaur Park Formation (Campanian) of Alberta. Anat Rec. 2020; doi:10.1002/ar.24241

Evans DC, Larson DW, Currie PJ. 2013. A new dromaeosaurid (Dinosauria: Theropoda) with Asian affinities from the latest Cretaceous of North America. Naturwissenschaften. 100 (11): 1041-1049. doi: 10.1007/s00114-013-1107-5

Line 215: replace “shows” with “has.”
Done

Line 216: delete “of…tooth.”
Done.

Line 217: replace “dividing” with “separating.”
Done

Line 222: rephrase, “slightly anteroposteriorly longer than mediolaterally wide.”
Done

Line 224: replace “mid-way depth” with “midheight.”
Done

Line 225: replace “a” with “an.”
Done

Line 229: replace “, but” with “that is”; the word “but” is used to introduce an exception or contradiction, not a similarity or an elaboration upon a point.
Fixed accordingly

Line 231: replace “mesodistal” with “mesiodistal.”
Done

Line 233: replace “damaging” with “damage.”
Done

Line 235: insert “13” ahead of “denticles.”
Done

	Line 236: delete ", with 13 denticles."<br><br>Done<br>Line 239: word choice – does "coarser" mean "larger"? If so, say so to remove the ambiguity.<br>Changed accordingly with larger.<br><br>Line 242: replace "observed" with "seen."<br>Done<br><br>Line 244: replace "a hypothetical" with "an"; how was this estimate arrived at? Briefly justify.<br>Fixed and added: accounting for the half of the carina being inset in the tooth socket: for clarity.<br><br>Line 246: delete "morphological."<br>Done<br><br>Line 253: what, exactly, is rounded and not pointed? I suspect this is a grammatical issue; please fix.<br>Replaced "rounded" with "with a sharp tip"<br><br>Line 263: replace "grains" with "grain."<br>Done<br><br>Line 266. Replace "in" with "along with."<br>Done<br><br>Line 267: replace "points…21183" with "is evidence that DMNH 21183 is a juvenile." Avoid passive voice – in science, it is ok to be declarative! In contrast, passive voice gives the impression that you are backing away from your evidence, which defeats the purpose of your hard work.<br>Thanks for pointing that out, changed accordingly.<br><br>Line 274: delete "traced and."<br>Done.<br><br>Line 294: delete "combination."<br>Done<br><br>Lines 294,295: "oval" and "lenticular" mean the same thing; pick one to avoid redundancy.<br>Replaced by just lenticular<br><br>Lines 300,301: replace "relatively better" with "well."<br>Done.<br><br>Line 310: replace "between" with "among."<br>Done<br><br>Lines 315,316: how is it that you can comment on the form of the first dentary tooth after you've made it clear that it is completely missing? Clarify.<br>Sentence removed.<br><br>Line 324: replace "observed" with "reported."<br>Done.<br><br>Line 328: replace "with" with "have"; replace "compared" with "in contrast." Also, does "coarser" just mean "larger?" Clarify.<br>All changed accordingly and larger chosen over "coarser".<br><br>Line 333: replace "this" with "the"; delete "taxon."<br>Done

Line 335: what about the ridge?!
Please see comment above.

Line 339: make sure that “anchiornithines” is the correct moniker to use. I.e., is there an “Anchiornithinae”?
Replaced with anchiornithid as Anchiornithidae has been favored by Hu et al. 2018 over the original definition of “Anchiornithinae” by Xu et al. 2016
Refs:
Xu; et al. (2016). "An Updated Review of the Middle-Late Jurassic Yanliao Biota: Chronology, Taphonomy, Paleontology and Paleoecology". Acta Geologica Sinica. 90 (6): 2229–2243. doi:10.1111/1755-6724.13033
Dongyu Hu; Julia A. Clarke; Chad M. Eliason; Rui Qiu; Quanguo Li; Matthew D. Shawkey; Cuilin Zhao; Liliana D’Alba; Jinkai Jiang; Xing Xu (2018). "A bony-crested Jurassic dinosaur with evidence of iridescent plumage highlights complexity in early paravian evolution". Nature Communications. 9 (1): Article number 217.
Line 343: replace “It….that” with “However,”; replace “been shown” with “occurred.”
Done

Line 345: pluralize “anchiornithid.”
Done

Lines 245 to 346: delete “, in….Eudromaeosauria.”
Done

Line 349: replace “reduction in” with “small” since there is no evolutionary or developmental process described here – you really don’t know if the size is plesiomorphic or the juvenile condition; avoid process-based terms when describing static, context-free morphology.
Thanks for the tip, and changed accordingly.

Line 353: replace “recalls a similar” with “is similar to the.”
Done

Line 354: pluralize “Velociraptorinae.”
Done

Line 357: “in-serrated/absent” are equivalent terms – pick one to reduce the annoyance of your reader. Concision is always appreciated in heavy osteodental descriptions!
Changed with unserrated.

Line 358: replace “observed” with “also seen in”; again, avoid passive voice.
Changed.

Line 365: replace “exhibit” with “have.”
Done.

Line 387: replace “are” with “include.’
Changed.

Line 392: delete “of the dentary.” Avoid redundancy.
Done

Line 394: replace “therefore” with “this criterion was”; delete “as…feature.”
Done

Line 395: delete “versus Dromaeosaurinae.”
Done

Line 397: briefly define DSDI/
Added “the ratio between number of mesial and distal denticles:

Line 402: is there a citation for the saurornitholestine teeth? What is their value?
Added and referenced relevant figure with DSDI plot

Lines 410-411: move these up to line 402 so that your reader can see how the saurornitholestine values compare right away. By the way, is there a difference between Saurornitholestinae and Velociraptorinae? Is see that Saurornitholestes is a velociraptorine, so Velociraptor is not a saurornitholestine?
Moved and modified also accordingly to some "in text" requests from Reviewer 1

Line 414: what is the DSDI? Give the value!
Added "outliers with DSDI around 2-3" and referenced relevant figure.

Lines 419 to 422: methods are best written in past tense. Please fix.
Fixed

Line 424: replace "provided" with "given."
Done

Lines 434 to 437: fix the commas.
Commas removed and replaced by bracketed explanation

Line 436: replace "provided" with" represented."
Done

Line 438: replace "presented" with "given."
Done

Lines 444 to 446: didn't you earlier state that the lateral ridge allies the specimen with Buitreraptor? How do you weight the dental data against the osteological evidence? Justify, especially since you are dealing with a juvenile specimen.
As mentioned in the response above, this has now been changed after new observations and reanalysis consequent to phylogenetic scoring.
Line 449: please stop using "status" for "growth stage."
Fixed
Lines 480 to 482: Given that dromaeosaurids are part of a clade of flying theropods, why couldn't have they just flown along their migratory routes? Explain.
While we believe it is un-parsimonious to attribute volant habits to members of Eudromaeosauria, we improved the context of this section of the Discussion, backing up our interpretation that it is unlikely that these theropods nested in southern latitudes, then migrated to northern latitudes in the summer. The occurrence of a young individual of such a small-size itself is evidence that this individual wouldn't have come from so far away as a migration entails, as it would have been dimensionally incapable of covering such large distance (e.g. Fiorillo & Gangloff 2001). The individual would not have at attained the minimum physical maturity necessary for such journey. Similar reasoning has been historically presented for hadrosaurid hatchlings by Horner (1982). While some basal members of Dromaeosauridae might have exhibited some sort of rudimentary (i. e. non-active flight), that might have been at best something close to what has been postulated for members of Microraptorinae (e.g. Sinornithosaurus and Zhenyuanlong (Xu et al, 1999; Alexander et al. 2010, Dyke et al. 2013), no such arguments have been made for saurornitholestines. Further, the anatomy of most dromaeosaurids, especially larger species, argues for a flightless lifestyle (Turner et al. 2007). That eliminates flight as an energy efficient means for these small predatory animals to cover the vast distances required to migrate across large wide geographic areas. Lastly, in response to a comment by one of the reviewers about the possibility of the young being volant, we point out that it is unlikely that the young forms migrated and the adult forms did not as there is no modern analog for such a model. We briefly discuss that within modern birds, the adults teach their young the necessary tools for migration (e.g. Baker, 1980).

Ref:
Horner JR. Evidence of colonial nesting and 'site fidelity' among ornithischian dinosaurs. Nature. 1982; 297(5868):675-676.

Dyke G, de Kat R, Palmer C, van der Kindere J, Naish D, Ganapathisubramani, B. Aerodynamic performance of the feathered dinosaur Microraptor and the evolution of

feathered flight. Nature Communications. 2013; 4 (2489). doi:10.1038/ncomms3489
Alexander, D. E., Gong, E., Martin, L. D., Burnham, D. A. & Falk, A. R. Model tests of gliding with different hindwing configurations in the four-winged dromaeosaurid Microraptor gui. Proceedings of the National Academy of Science. 2010; 107, 2972-2976.
Xu X, Wang X-L, Wu X-C. A dromaeosaurid dinosaur with filamentous integument from the Yixian Formation of China. Nature. 1999; 401:262–266.
Turner AH, Pol D, Clarke J, Erickson G, Norell M. A basal dromaeosaurid and size evolution preceding avian flight. Science. 2007; 317:1378–1381.

Line 488: replace “southerner” with “southern.”
Done

Lines 485 to 488: why is large size evidence for “success”? Justify. What is meant by “success”? Clarify.
We removed this section as we in the end agreed with the consideration that most of this reasoning was too speculative and outside the scope of the study.

Line 493: change “stronger constrained” to “greater constraints.” What sort of constraints? Clarify.
Same as above

Lines 494 to 495: convoluted; fix.
Fixed.

Line 498: “eggs” should be singular.
Fixed

Lines 494 to 505: perhaps it is because it is late in the day, but I find this section to be very difficult to follow. Please redraft with a clearer argument and straightforward sentence structure.
Thanks for the suggestion. We redrafted this section to simplify the reasoning and expunge the speculative content as recommended by the reviewer.

Line 506: avoid passive voice; fix.
Fixed.

Lines 510 to 512: this can be shortened significantly; fix.
Done

Lines 513 to 515: the mere presence of taxon does not imply migration any more than it doesn’t imply migration. Fix.
Done

Line 523: should there be an “r” in “cfr”? Isn’t it just “cf.”? Where’s the period? Fix.
Fixed

Line 525: replace “appear” with “are.”
Fixed.

Line 526: replace “but” with “and” since you aren’t marking a difference or exception.
Fixed

Line 544: a period should follow “al”; e.g., Osborn et al. (1905); replace “for” with “that.”
Done

Line 545: insert “is” after the taxon name.
Added

Lines 513 to 555: I find this section to be, uncomfortably, speculative, but I leave it up to the judgment of the author and co-authors whether or not it needs to be reined in. Perhaps more citations in this section would put any concerns to rest. It just strikes me as a bridge too far when all of it is merely based on the relative abundance of teeth and – as far as I can tell - inconclusive functional inferences of tooth morphology. Please

	reconsider this section in a more critical light.<br><br>See comments above.<br><br>Lines 560 to 562: You don't really know that it wasn't migratory; it could be that all small dromaeosaurids were fully capable of flight early in growth. Please reconsider this point more critically – does the inference really have support?<br>See comments above.<br><br>Lines 564 to 565: again, you have no rationale to think this – if dromaeosaurids were volant, there's literally nothing to stop them from migrating. It's possible flight brought deinonychosaurians their global distribution. Regardless, I don't think you have the evidence to make the claim, unless there's something obvious that I missed earlier.<br>See comments above.<br><br>Figure 3: "a3" is not labeled; label both interdental plates; label the circular fossa.<br>Done.
Additional Information:	
**Question**	**Response**
**Financial Disclosure**<br><br>Enter a financial disclosure statement that describes the sources of funding for the work included in this submission. Review the submission guidelines for detailed requirements. View published research articles from *PLOS ONE* for specific examples.<br><br>This statement is required for submission and **will appear in the published article** if the submission is accepted. Please make sure it is accurate.<br><br>**Unfunded studies**<br>Enter: *The author(s) received no specific funding for this work.*<br><br>**Funded studies**<br>Enter a statement with the following details:<br>• Initials of the authors who received each award<br>• Grant numbers awarded to each author<br>• The full name of each funder<br>• URL of each funder website<br>• Did the sponsors or funders play any role in the study design, data collection and analysis, decision to publish, or preparation of the manuscript?<br>• **NO** - Include this sentence at the end of your statement: *The funders had no role in study design, data collection and analysis, decision to publish, or preparation of the manuscript.*<br>• **YES** - Specify the role(s) played.	ARF received funding for this project through the Perot PaleoClub, a private donation.The Perot Paleo Club played no role in the study design, data collection and analysis, decision to publish, or preparation of the manuscript.

* typeset	
**Competing Interests**<br><br>Use the instructions below to enter a competing interest statement for this submission. On behalf of all authors, disclose any competing interests that could be perceived to bias this work—acknowledging all financial support and any other relevant financial or non-financial competing interests.<br><br>This statement **will appear in the published article** if the submission is accepted. Please make sure it is accurate. View published research articles from *PLOS ONE* for specific examples.<br><br>**NO authors have competing interests**<br><br>Enter: *The authors have declared that no competing interests exist.*<br><br>**Authors with competing interests**<br><br>Enter competing interest details beginning with this statement:<br><br>*I have read the journal's policy and the authors of this manuscript have the following competing interests: [insert competing interests here]*<br><br>* typeset	The authors have declared that no competing interests exist.
**Ethics Statement**<br><br>Enter an ethics statement for this submission. This statement is required if the study involved:<br><br>• Human participants<br>• Human specimens or tissue<br>• Vertebrate animals or cephalopods<br>• Vertebrate embryos or tissues<br>• Field research<br><br>Write "N/A" if the submission does not require an ethics statement.	N/A

Powered by Editorial Manager® and ProduXion Manager® from Aries Systems Corporation

General guidance is provided below. Consult the submission guidelines for detailed instructions. **Make sure that all information entered here is included in the Methods section of the manuscript.**<br><br>**Format for specific study types**<br><br>**Human Subject Research (involving human participants and/or tissue)**<br>• Give the name of the institutional review board or ethics committee that approved the study<br>• Include the approval number and/or a statement indicating approval of this research<br>• Indicate the form of consent obtained (written/oral) or the reason that consent was not obtained (e.g. the data were analyzed anonymously)<br><br>**Animal Research (involving vertebrate animals, embryos or tissues)**<br>• Provide the name of the Institutional Animal Care and Use Committee (IACUC) or other relevant ethics board that reviewed the study protocol, and indicate whether they approved this research or granted a formal waiver of ethical approval<br>• Include an approval number if one was obtained<br>• If the study involved *non-human primates*, add *additional details* about animal welfare and steps taken to ameliorate suffering<br>• If anesthesia, euthanasia, or any kind of animal sacrifice is part of the study, include briefly which substances and/or methods were applied<br><br>**Field Research**<br><br>Include the following details if this study involves the collection of plant, animal, or other materials from a natural setting:<br>• Field permit number<br>• Name of the institution or relevant body that granted permission	
**Data Availability**<br><br>Authors are required to make all data	Yes - all data are fully available without restriction

underlying the findings described fully available, without restriction, and from the time of publication. PLOS allows rare exceptions to address legal and ethical concerns. See the PLOS Data Policy and FAQ for detailed information.<br><br>A Data Availability Statement describing where the data can be found is required at submission. Your answers to this question constitute the Data Availability Statement and **will be published in the article**, if accepted.<br><br>**Important:** Stating 'data available on request from the author' is not sufficient. If your data are only available upon request, select 'No' for the first question and explain your exceptional situation in the text box.<br><br>Do the authors confirm that all data underlying the findings described in their manuscript are fully available without restriction?	
**Describe where the data may be found in full sentences. If you are copying our sample text, replace any instances of XXX with the appropriate details.**<br><br>• If the data are **held or will be held in a public repository**, include URLs, accession numbers or DOIs. If this information will only be available after acceptance, indicate this by ticking the box below. For example: *All XXX files are available from the XXX database (accession number(s) XXX, XXX.)*.<br>• If the data are all contained **within the manuscript and/or Supporting Information files**, enter the following: *All relevant data are within the manuscript and its Supporting Information files.*<br>• If neither of these applies but you are able to provide **details of access elsewhere**, with or without limitations, please do so. For example:<br><br>*Data cannot be shared publicly because of [XXX]. Data are available from the*	All relevant data are within the manuscript and its Supporting Information files

XXX Institutional Data Access / Ethics Committee (contact via XXX) for researchers who meet the criteria for access to confidential data.<br><br>*The data underlying the results presented in the study are available from (include the name of the third party and contact information or URL).*<br>• This text is appropriate if the data are owned by a third party and authors do not have permission to share the data.<br><br>* typeset	
Additional data availability information:	

Powered by Editorial Manager® and ProduXion Manager® from Aries Systems Corporation

Manuscript
Click here to access/download;Manuscript;Chiarenza et al. resubmission-Clean Copy.docx

The first juvenile dromaeosaurid (Dinosauria: Theropoda) from Arctic Alaska

Alfio Alessandro Chiarenza[1*], Anthony R. Fiorillo[1], Ronald S. Tykoski[1], Paul J. McCarthy[2], Peter P. Flaig[3], Dori L. Contreras[1]

[1] Perot Museum of Nature and Science, Dallas, TX, USA, [2] Department of Geosciences, University of Alaska, Fairbanks, AK, USA [3] Jackson School of Geosciences, Bureau of Economic Geology, University of Texas at Austin, Austin, TX, USA

* a.chiarenza15@gmail.com

Abstract

Compared to the osteological record of herbivorous dinosaurs from the Late Cretaceous Prince Creek Formation of northern Alaska, there are relatively fewer remains of theropods. The theropod record from this unit is mostly comprised of isolated teeth, and the only non-dental remains known can be attributed to the troodontid cf. *Troodon* and the tyrannosaurid *Nanuqsaurus*. Thus far, the presence of members of Dromaeosauridae has been limited to isolated teeth. Here we describe a symphyseal portion of a small dentary with two ziphodont teeth. Based on tooth shape, denticle morphology, and the position of the Meckelian groove, we

attribute this partial dentary to a saurornitholestine dromaeosaurid. The fibrous bone surface, small size, and higher number of mesial denticles compared to distal ones point to a juvenile growth stage for this individual. Multivariate comparison of theropod teeth morphospace by means of principal component analysis reveals an overlap between this dentary and Saurornitholestinae dromaeosaurid morphospace, a result supported by phylogenetic analyses. This is the first confirmed non-dental fossil specimen from a member of Dromaeosauridae in the Arctic, expanding on the role of Beringia as a dispersal route for this clade between Asia and North America. Furthermore, the juvenile nature of this individual adds to a growing body of data that suggests Cretaceous Arctic dinosaurs of Alaska did not undergo long-distance migration, but rather they were year-round residents of these paleopolar latitudes.

Competing interests: the authors have declared that no competing interests exist.

Introduction

Dromaeosauridae [1–3] (Table S1) is a group of predatory theropod dinosaurs evolutionarily close to the origin of birds [4, 5]. This clade likely originated in the Middle Jurassic [6, 7], with the first definitive dromaeosaurids

recovered from Cretaceous deposits [8]. By the Late Cretaceous they reached a virtually cosmopolitan distribution [9], so far remaining unknown only in Antarctica. Given the small to medium size of most dromaeosaurids, and their fragile, highly pneumatic skeletons that are subject to greater incompleteness bias than many other dinosaur taxa [10, 11], complete remains of this group are generally rare and confined to exceptionally productive fossil localities [e.g. 12, 13]. North American taxa belong to at least 4 recognized major subclades (Dromaeosaurinae, Microraptorinae, Saurornitholestinae, and Velociraptorinae; Table S1 [14]) with probable Asian origins based on phylogenetic inference and local abundance of taxa referred to these clades [8]. Since the earliest discoveries of dinosaur remains on the Alaskan North Slope (15–17), the number of studies describing dinosaurs from the Prince Creek Formation and their role in clarifying paleobiogeographic and paleoecological aspects of the Cretaceous Arctic has greatly increased (e.g. 18–25).

Dinosaur teeth often preserve more easily and are more frequently recovered than bones [26], and the discovery of isolated teeth referable to Dromaeosauridae in many Late Cretaceous microsites has often provided important biogeographic data confirming the presence of the group in areas for which purely osteological remains are unknown [9, 27]. For example, Fiorillo and Gangloff [28] reported on isolated dromaeosaurid teeth from the Prince Creek Formation of Alaska,

tentatively referring them to *Dromaeosaurus* and *Saurornitholestes*. Given the intermediate paleogeographic position of Alaska (as part of the ancient Beringian landmass), and its role as a land bridge between Asia and North America, additional dromaeosaurid remains with better resolved taxonomic identification have the potential to increase our understanding of the origin and dispersal of these clades through Asiamerica. Here we describe the first non-dental, osteological material of a saurornitholestine dromaeosaurid from Alaska, representing a unique morphotype. This find supports the presence of this clade in the Upper Cretaceous (lower Maastrichtian) Prince Creek Formation on the North Slope of Alaska (70° N, Fig 1).

Materials and Methods

DMNH 21183 is a symphyseal portion of a theropod dentary with a semi-erupted tooth and a replacement tooth preserved. The specimen was studied using a Nikon SMZ motorized stereomicroscope (Nikon Instruments, Inc., Melville, NY, USA), equipped with epi-fluorescence with an X-Cite XYLIS light source (Excelitas Technologies, Waltham, MA, USA) and GFP filter. Imaging of the specimen, including focal stacking and 3-D reconstructions, was completed using an attached Nikon Ri2 color CMOS camera with Nikon's NIS-Elements Acquisition and Analysis Software. Additional microscope observations and

imaging were carried out using a Keyence Digital VHX-7000 series microscope (Keyence Corporation of America, Itasca, IL, USA). Dental nomenclature and terminology is based on Hendrickx et al. [29]. Terminology regarding ontogenetic characters is mostly, but not exclusively based on Sampson et al. [30], Carr [31], and Hone et al. [32]. Anatomical description is based on morphological observation by three of the authors (AAC, AF, RT). Comparisons were made based on first hand observations of relevant material by AAC, AF, and RT, as well as literature comparisons. Stratigraphic and sedimentological observations were carried out by three of the authors (AF, PF, PM) between 2005 and 2014 (Fig 2). Cladistic analyses and character evaluation were conducted by authors AAC, DC, RT, and AF.

To assess the systematic position of DMNH 21183 within Theropoda, we performed three different phylogenetic analyses. The first analysis, based on osteological and dental characters, used the dataset from Lee et al. [33], which included 120 operational taxonomic units (OTUs) and 1529 characters. See Lee et al. [33] and Cau et al. [34] for further information on character choice and coding. Our updated matrix differed only in the addition of DMNH 21183 as an OTU. The second and third analyses followed the protocols outlined by Hendrickx et al. [35] to identify isolated theropod teeth. DMNH 21183 was scored in one matrix based on dentition characters, and another on tooth-crown-only characters. Details on

character choice and rationale can be found in Hendrickx et al. [35], where original datasets are also reported as supplementary material. The dentition-based matrix consists of 107 taxa and 146 characters. The tooth-crow-based matrix includes 102 taxa and 91 characters. These dental datasets were analyzed by additionally forcing a topological constraint following Hendrickx et al. [35], reflecting a previously recovered tree topology for Theropoda (e.g. Rauhut and Carrano [36] and Brusatte et al. [37]), with DMNH 21183 as a floating OTU. Character scorings for DMNH 21183 in all three phylogenetic datasets are reported in Supplementary Information Document (S1 Dataset). Characters were all equally-weighted and treated as unordered or ordered following the source literature. The phylogenetic analyses were run using the software TNT [38]. For each dataset, we performed a "New Technology" search that included a combination of random and constraint sectorial searches, ratchet, tree-drifting, and tree-fusing, with ten search replications as the starting point for each hit and searches carried out until 100 hits of the same minimum tree length were achieved (TNT command used is "xmult= hits 100 replic 10 css rss ratchet 5 drift 5 fuse 5"). The most parsimonious trees (MPTs) obtained were subjected to two rounds of TBR branch swapping (command "bbreak =TBR"). Strict consensus trees were generated from the resulting set of MPTs Nodal support was calculated for the consensus by running a standard bootstrap analysis with 1000 replicates.

To test the qualitative observations reported in the anatomical description section, we performed multivariate analyses of theropod tooth measurements including those retrieved by the erupted tooth in DMNH 21183 (S2 Dataset). Although this tooth is not fully erupted, the breaks on the medial side of the dentary around the area of the relative alveolus and observations under microscopy allowed measurement of the crown height of the tooth. Measurements on tooth morphometrics are from Gerke and Wings [39] and a modified dataset from Larson and Currie [40] (S2 Dataset). The dataset from Gerke and Wings [39] includes measurement data for 335 theropod teeth for which entries were modified to reflect a higher systematic rank or clade compared to the original genus-level classification (e.g. Abelisauridae, Dromaeosauridae, Tyrannosauridae). Measurements for this dataset include crown basal length (CBL), crown height (CH), crown basal width (CBW), and the ratio between mesial (anterior) and distal (posterior) denticles (DSDI). This dataset has been used previously in other studies (e.g. [35]).

The dataset from Larson and Currie [40] includes measurement data for over 1200 small theropod teeth, mainly from the latest Cretaceous of the Western Interior Basin of North America, and has also been used for similar studies in the past [e.g. 41, 42]. Principal measurements included fore-aft basal length (FABL), crown height (CH), basal width (BW), and anterior (ADM) and posterior denticles

per millimeter (PDM). This dataset was modified by removing all entries with at least one variable missing or unable to be evaluated, such as the lack of a measurement caused by the absence of the related structure (e.g. ADM in teeth without mesial denticles or carina). In this dataset, the taxonomy of the identified teeth was referred to the "family-ranked" clade with the exception of *Richardoestesia*. For example, *Saurornitholestes langstoni* and *Atrociraptor marshalli* are both referred to Saurornitholestinae, following [14]. The Milk River cf. *Zapsalis* is referred to *Saurornitholestes* following Currie and Evans [43]. The Aquilan cf. *Richardoestesia gilmorei*, the Oldman cf. *Richardoestesia gilmorei*, the Horseshoe Canyon cf. *Richardoestesia*, the Lancian cf. *Richardoestesia*, the Aquilan cf. *Richardoestesia isosceles*, and *Richardoestesia isosceles* are all referred to *Richardoestesia*. The subclades of theropods included in this dataset, apart from the specimen studied first hand (DMNH 21183) are: Dromaeosaurinae, *Richardoestesia*, Saurornitholestinae and Troodontidae.

We performed two principal components analyses (PCA), a multivariate technique that takes a number of measurements and converts them into a smaller set of values that represent the variability of the sample plotted in a multivariate space. Following the example of similar studies (e.g. [9, 35, 39]), the same measurements were also used for a linear discriminant function analyses (DFA) on both the datasets. This method provides a value to assess the degree of confidence

on the classification of the clusters in the morphospace, where 0.5 is no better than random in model accuracy while 1 represents perfect accuracy (100% accuracy [44]). Both these methods allow the dataset of teeth to be plotted in a morphospace, and to quantitatively compare the degree of overlap and the relative position of DMNH 21183 with 1) the main clades of Theropoda from the Gerke and Wings [39] dataset and 2) the main deinonychosaurian subclades (e.g., Dromaeosaurinae, Troodontidae) from the Larson and Currie [40] dataset. Multivariate analyses were performed in the software "R" with the "MASS" package [45]. PCA outcomes are reported as results in the relevant section while DFA plots are provided in Supplementary Information (S2-S3 Figs). See Table S1 for systematic definitions of clades and lineages mentioned here and used throughout the text.

Institutional Abbreviations

AMNH–American Museum of Natural History, New York City, USA; DMNH–Perot Museum of Nature and Science, Dallas, Texas, USA; IVPP–Institute for Vertebrate Paleontology and Paleoanthropology, Beijing, China; MPC-D–Institute of Paleontology and Geology, Mongolian Academy of Sciences (formerly known as IGM), Ulaanbaatar, Mongolia; YPM–Yale Peabody Museum of Natural History, Yale, Connecticut, USA; NHMUK PV–Natural History

Museum, London, UK; TMP–Royal Tyrrell Museum of Palaeontology, Drumheller, Alberta, Canada

Results

Locality

The specimen was collected from exposures of the Prince Creek Formation, at the Pediomys Point locality (Figs 1 and 2). The locality is along the Colville River, 8 km upstream from the Liscomb bonebed, North Slope Borough, Alaska, USA (Fig 1). Bulk sediment was collected at the site over multiple field seasons between 2005-2007, 2012, and 2014, with screenwashing and sorting of the material conducted at the Perot Museum of Nature and Science (DMNH) in Dallas, Texas, USA.

Geological Setting and Depositional Environments

The Prince Creek Formation (PCF) was deposited in the Colville Basin of northern Alaska and provides us with the largest collection of polar dinosaur bones in the world [25]. The PCF was originally sub-divided into two subunits or tongues: an older Tuluvak Tongue and a younger Kogosukruk Tongue [46]. However, Mull et al. [47] revised this nomenclature based on regional stratigraphic correlations and the Prince Creek Formation was redefined to include only the

former Kogosukruk Tongue along with some younger, Paleocene strata. The total thickness of the PCF along the Colville River is approximately 450 m [48, 49]. Biostratigraphic [50–59], and isotopic analyses [60] indicate that the age of the Prince Creek Formation ranges from Campanian to Paleocene. All deposits containing evidence of dinosaurs are Early Maastrichtian in age and approximately 68.5-70 million years old [57, 59, 61–63].

The PCF is an alluvial succession composed of conglomerate, sandstone, siltstone, mudstone, carbonaceous shale, coal, and bentonite [59, 61, 64]. The mostly fine-grained sediments record deposition in low energy, suspended load channels and on organic-rich floodplains on a low-gradient coastal plain. Thicker, multi-story meandering trunk channels contain the largest grain sizes and record the highest flow velocities in the area. Smaller meandering and fixed or anastomosed distributary channels formed crevasse splay-complexes adjacent to trunk channels. Abundant organic-rich facies were deposited in low-lying areas between the large channels and splay-complexes. Floodplains contained levees, splays, lakes, ponds, swamps, and soil-forming environments. Volcanic ashfall was common and smectite-rich bentonites were commonly preserved in wet floodplain environments [65]. Trampling of sediments by dinosaurs was common [63]. Weakly-developed paleosols similar to modern *Entisols*, *Inceptisols*, andic soils, and potential acid sulfate soils formed on levees, point bars, crevasse splays, and

along the margins of floodplain lakes, ponds, and swamps that also supported lowland trees, shrubs, herbs, ferns, moss, and algae [61, 65]. Macroscopic and micromorphological features, and illite-smectite mixed-layer clay minerals in paleosols indicate predominantly waterlogged, reducing conditions interrupted by oxidizing conditions and periodic drying out of some soils [61, 62, 64, 65]. Soils experienced repeated sediment influx from overbank flooding of nearby distributary channels, and periodic deposition of hyperconcentrated flows [62]. Sediments deposited in the most distal areas of the coastal plain contain evidence of marine influence that includes inclined heterolithic stratification in channel point bars and pyrite, jarosite mottles, jarosite halos surrounding root-traces, and brackish-water fauna in floodplain facies [64, 66].

The 750 m-long outcrop belt at Pediomys Point is located near the upriver end of a slough off the main course of the Colville River (Fig 1). Cretaceous environments preserved at Pediomys Point include a meandering distributary channel that transitions laterally into a silt and mud-filled abandoned channel along with floodplain environments that include crevasse splays, levees, small lakes and swamps, floodplain paleosols, and ashfall deposits ([67], Fig 2). Trampled sediments found above a bentonite (Fig 2) are similar to those described from strata 4.25 kilometers downstream along the Colville River that are interpreted as adult and juvenile hadrosaur tracks emplaced along a swamp margin [63]. Rare

brackish-water clams (most likely *Nucula* aff. *N. percrassa* Conrad; see [66]) and gastropods found near the top of the stratigraphic succession at Pediomys Point suggest an estuarine or lagoonal environment for those deposits [66]. Interfingering of continental-terrestrial and shallow marine deposits, including those of floodbasins, interdistributary bays and estuaries were identified in older deposits of the PCF along the Colville River [68] and in younger deposits above the Liscomb Bonebed near Ocean Point [69]. This suggests that the environments at Pediomys Point were transitional between the subaerial coastal plain or delta plain, and shallow marine habitats (Fig 2).

Systematic Paleontology

Dinosauria [70]

Theropoda [71]

Dromaeosauridae [1]

Eudromaeosauria [14]

Saurornitholestinae [14] indet.

Referred specimen

DMNH 21183. The anterior portion of a right dentary, preserving two teeth and four alveoli (Fig 3).

Description

DMNH 21183 (Fig 3) (Table 1) is an anterior portion of a right dentary with an unerupted mesial-most tooth (d2; Fig 3A–3C) and a distally placed partially erupted tooth (d3; Fig 3). The anterior surface of the dentary is damaged, obscuring details of the symphyseal region. Given the position of the erupted tooth (d3; Fig 3) above the Meckelian foramina (on the medial surface of the dentary, Fig 3B), and above an the anteroventral process of the dentary (ave; Figs 3A and 3B), we identify this as the 3rd dentary tooth in the dentary [e.g. 43, 72], with the more mesially positioned, unerupted tooth (d2) being identified as the 2nd. Alveoli 2–4 are preserved (Fig 3A–C), although the margins of the latter (fourth) are obscured by erosion. Both the medial and lateral surfaces of the dentary present a parallel, anteroposteriorly-oriented, fibrous bone texture. The anterior margin of the 3rd alveolus (a3; Fig 3A–C) has a raised anterior rim that extends dorsally up to approximately mid-height of the crown (a3). There is a well-preserved subtriangular interdental plate lingual to the septum separating the 2nd and 3rd alveoli (a2–a3), and a bigger, semicircular one between the 3rd and 4th alveoli (a3–a4). The paradental space is relatively dorsoventrally short. Laterally, the dorsal rim of the dentary rises into a triangular ridge, which creates a convex surface anterolaterally that sinks at its base into a circular fossa. The alveoli are elliptically

shaped, being slightly anteroposteriorly longer than mediolaterally wide (Fig 3C). The medial side of the dentary features a shallow Meckelian groove (Fig 3B), set slightly more ventrally than mid-height of the bone. The medial side of the dentary features an anteriorly located Meckelian foramen (Fig 3B), which may be paired with a second, more ventrally positioned foramen, but damage to the bone surface in this area makes the identification of this feature uncertain. There is an anteroventral expansion in of the dentary margin of the dentary, visible both medially and laterally (Figs 3A and 3B) that is excavated by a sub-oval fossa on the lateral side (Fig 3A).

Both preserved teeth in DMNH 21183 are ziphodont. The mesodistal axis of the 2nd tooth (d2) is more anteromedially oriented in relation to the lateral margin of the dentary. The 2nd tooth is unerupted, but damage to the medial surface of the alveolar wall exposes the most apical half of the tooth crown. While the anterior alveolar margin covers the mesial surface of the 2nd tooth (d2), 13 denticles are visible along the distal carina (Figs 4A and 4B). The apical-most half of portion of the 3rd tooth (d3) is erupted, with 12 denticles visible on the distal carina (Figs 3A, 3B and 3D).

The teeth have larger distal denticles than the mesial ones. The unerupted portion of the third tooth crown can be seen through a fracture on the medial side

of the dentary (Fig 3B). Taking into account the base of this crown, an estimate of ~30 denticles per serrated carina can be inferred for the tooth.

Unfortunately, wear of the mesial carina destroyed some details of the denticles. However, apart from some denticles, most of the interdenticular grooves between adjacent denticles are clear, and they are shallow incisions rather than deep sulci (Fig 3D, S1 Fig). In the 2nd tooth this comparison between mesial and distal carina is not possible because the mesial margin of the tooth is hidden by sediment and the labial wall of the alveolus (Figs 4A and 4B). The shape of the apices of the distal denticles is slightly hooked, with an orientation toward the apex of the crown and with an externally rounded margin rather than with a sharp tip (Figs 4A–4C).

Table 1. Measurements of DMNH 21183

Elements measured	Measurements (mm)
Dentary anteroposterior length (dorsal view)	14.34
Dentary anteroposterior length (lateral view)	14.59
Maximum dentary mediolateral length	6.15
Maximum dentary dorsoventral depth	9.44
Alveolus II mesodistal length	2.95
Alveolus II labiolingual width	1.27
Alveolus III labiolingual width	2.76
Alveolus III mesodistal length	3.71
Tooth II mesodistal width	1.97
Tooth II labiolingual length	1.05

Phylogenetic analysis results

Analysis of the Lee et al. [33] data matrix with DMNH 21183 added resulted in 384 MPTs that are 6043 steps long, with a Consistency Index (CI) = 0.244 and a Retention Index (RI) = 0.587. The strict consensus (Fig 5; S4 Fig) reproduced the topology hypothesized by Lee et al. [33]. DMNH 21183 is recovered in Dromaeosauridae excluding Unenlaginae, in a polytomy with most other eudromaeosaurs (*Dromaeosaurus* + *Utahraptor*) and Microraptorinae clades.

The addition of DMNH 21183 to the dental-only character matrix of Hendrickx et al. [35] produced 2 MPTs (1314 steps long, CI = 0.194, RI = 0.418). The Strict Consensus of the two trees recovers Paraves as a trichotomy between Avialae, Troodontidae and Dromaeosauridae (Fig. 6A; S5 Fig). DMNH 21183 is recovered as the sister OTU of *Saurornitholestes*, in a partially resolved Eudromaeosauria (*sensu* Longrich and Currie [14]). This dromaeosaurid clade is represented by a polytomy between *Atrociraptor*, a monophyletic clade with *Deinonychus*, *Tsaagan* and *Velociraptor*, and another monophyletic clade with *Dromaeosaurus* and *Bambiraptor* as successively closer taxa to the clade *Saurornitholestes* + DMNH 21183.

Lastly, the analysis that includes DMNH 21183 in the tooth-crown-based data matrix of Hendrickx et al. [35] recovers 5 MPTs (867 steps, CI = 0.183; RI = 0.439). The strict consensus tree (Fig 6B; S6 Fig) produces a polytomy between alvarezsaurs, therizinosaurs, oviraptorosaurs and the rest of Maniraptora (Avialae,

Troodontidae and Dromaeosauridae). DMNH 21183 is recovered within Eudromaeosauria (*sensu* Longrich and Currie [14]), in a polytomy between *Atrociraptor*, *Bambiraptor*, *Deinonychus*, *Saurornitholestes*, and an Asian Velociraptorinae clade (*Velociraptor* + *Tsaagan*).

Multivariate analysis results

The dentition of DMNH 21183 (the 3rd and more exposed-better preserved tooth; d3 in Fig 3D) was assessed in a morphometric dataset of theropod teeth [39], with a PCA analysis returning four axes with the following eigenvalues and percentages of total variance explained by each axis: Axis 1 (0.377, 96.583%), Axis 2 (0.006, 1.594%), Axis 3 (0.004, 1.13%), Axis 4 (0.003, 0.69%). Coefficients for the five measurements on each axis are given in Table 2. The majority of the variance is captured in the first two axes of the principal components. The highest variable contribution is represented by CBW (37.79%), followed by CH (~33.70%), CBL (~28.12%), and lastly DSDI (0.38%). The position of DMNH 21183 in the first two axes of the theropod teeth morphospace is shown in Fig 7. DMNH 21183 overlaps the dromaeosaurid morphospace in the lower left quadrant of the plot. This convex hull partially overlaps with those of troodontids, noasaurids, and basal theropods among others. This cluster is set on the opposite side from the centroids of allosauroids, ceratosaurids, and

spinosaurids, which occupy most of the center and right area of the teeth morphospace. DMNH 21183 is in the opposite area of the morphospace than tyrannosaurids (Fig 7). A similar spatial arrangement is obtained with the DFA analysis (S2 Fig), in which model accuracy for classification of DMNH 21183 as a dromaeosaurid is of 0.65. To further explore the position of DMNH 21183 between Dromaeosauridae and the other taxa that were morphometrically close in this first set of multivariate analyses, we used a dataset [40] of deinonychosaurian teeth (see Materials and Methods) and performed a PCA analysis. The analysis returned four axes with the following eigenvalues and percentages of total variance explained by each axis: Axis 1 (15.005, 77.175%), Axis 2 (3.584, 18.433%), Axis 3 (0.559, 2.879%), Axis 4 (0.294, 1.513%). Coefficients for the five measurements on each axis are given in Table 3.

Table 2. PCA coefficients for morphometric analysis with all theropods.

Measurement	PC1	PC2	PC3	PC4
**CBL**	0.530305	-0.207765	0.108537	0.814757
**CBW**	0.614766	0.7250023	-0.251823	-0.181711
**CH**	0.580509	-0.605216	0.065003	-0.540829
**DSDI**	-0.062033	-0.254791	0.959468	0.1032189

Coefficients of the PCA analysis run on the theropod teeth dataset published by Gerke and Wings [39] with the addition of DMNH 21183. ADM, anterior denticles per millimeter; BW, basal width; CBL, crown base length (in mm); CBW: crown base width; CDA: crown distal

angle; CH: crown height (in mm); CH, crown height; DSDI: denticle size difference index, denticles in mesial carina divided by those in distal carina.

Table 3. PCA coefficients for morphometric analysis with deinonychosaurian taxa.

Measurement	PC1	PC2	PC3	PC4
**FABL**	0.352856	-0.03872	0.568433	0.742211
**CH**	0.838152	0.423491	-0.31658	-0.13392
**BW**	0.176441	-0.00224	0.739112	-0.65006
**PDM**	-0.37665	0.90507	0.174281	0.092801

Coefficients of the PCA analysis run on the deinonychosaurian teeth dataset published by Larson and Currie [40] with the addition of DMNH 21183. ADM, anterior denticles per millimeter; BW, basal width; CH, crown height; FABL, fore-aft basal length; and PDM, posterior denticles per millimeter.

The majority of the variance is explained by the first two axes of the principal components. The highest variable contribution (similarly to previous analyses on this dataset [40–42]) is represented by CH (~60%) followed by PDM (~30%), FABL (~15%) and lastly ADM (<5%). The position of DMNH 21183 in the first two axes of the deinonychosaurian teeth morphospace is shown in Fig 8. DMNH 21183 overlaps the Saurornitholestinae morphospace, being set further away from the centroid clustering the other analyzed taxon with hooked denticles, Troodontidae. It is outside the convex hull comprising Dromaeosaurinae, which

has mostly subequal, rectangular denticles, and is set within the Saurornitholestinae morphospace in a position slightly toward the *Richardoestesia* morphotype. DFA results in a spatial arrangement of data points similar to the one of the PCA (S3 Fig), and the analyses provides a model accuracy of 0.8 for classification of DMNH 21183 as a saurornitholestine deinonychosaur. This outcome is comparable to those generated by the phylogenetic analyses, and we confidently refer DMNH 21183 to the Saurornitholestinae. We do not at this time assign it to any currently recognized species within the clade.

One major caveat of these analyses regards the potential bias due to the different ontogenetic stages of the teeth included in the sample [41], and most importantly in relation to the likely juvenile growth stage of DMNH 21183. Because PCA analysis would simply group observations based on measurements, it is likely that teeth belonging to juvenile and adult individuals will cluster in separate areas of the morphospace (see Discussion for a more in depth comparison of denticle size). This issue has been shown in the literature (e.g. [73, 74]) to affect previous PCAs of tyrannosauroid teeth, so we use this morphometric line of evidence as a complementary tool to assess the systematic identification of DMNH 21183.

Discussion

Morphological comparisons and the phylogenetic position of DMNH 21183

While DMNH 21183 is fragmentary, there are enough anatomical characters preserved in it to indicate its likely systematic position. Although very few non-dental theropod remains have been found in the Prince Creek Formation of Alaska, a general comparison with other contemporaneous theropod taxa, with a particular focus on those clades previously recognized in the formation (Dromaeosauridae, Tyrannosauridae, Troodontidae) is attempted here. The combination of ziphodont dentition, presence of interdental plates (e.g. [75]), a Meckelian groove, and Meckelian foramina are characteristic of theropod dinosaurs (e.g. [76]). The orientation of the anterior-most tooth socket and its unerupted tooth (both anteromedially oriented), the presence of paired Meckelian foramina, and a ventral expansion laterally pierced by a fossa, point toward the identification of the specimen as an anterior portion (almost symphyseal, as also shown by the ventral enlargement of the dentary in lateral view) of a theropod dentary. The shallow Meckelian groove present in the specimen is a derived maniraptoran feature [77], in contrast to the deep groove seen in basal tyrannoraptorans (e.g. Tyrannosauroidea) [78]. The Meckelian foramen (Fig 3B) is slit-like and not enlarged and rounded like in Tyrannosauridae (e.g. oral mandibular foramen in *Nanuqsaurus* [23] and foramen intramandibularis oralis in *Tyrannosaurus* [79]), a feature seen in Dromaeosauridae (e.g. *Dromaeosaurus*, [80]; e.g. *Acheroraptor*,

[72]). The pair of anterior foramina nearby the symphysis is similar to the condition found in velociraptorines like *Acheroraptor* [72]. The shallow paradental space, a Meckelian groove that is set in the lower half of the medial side of the dentary but not directly on the ventral margin, and the hooked distal denticles of the teeth are coelurosaurian features [81, 82]. The lenticular shape of the alveoli from dorsal view (Fig 3C; S1 Fig), rather than box shaped (or more squared off), is more similar to the condition in derived coelurosaurians (i.e. Maniraptora) rather than in Tyrannosauroidea [83]. The presence of distinct alveoli rather than a connected dorsal furrow excludes derived troodontids [83]. The triangular ridge on the lateral margin of the dentary (Figs. 3A and 4D) is shared with *Buitreraptor* [84], *Velociraptor* (AMNH 6515) and *Tsaagan* [85], although in all these cases the presence of this structure may be an artifact of the damaged lateral rim of the dentary. The raised rim in front of the well-preserved alveolar rim 4 (Fig 3A) resembles the condition of the anterior alveoli in *Saurornitholestes* [43].

Interdental plates are notoriously rare in paravian theropods, and fusion or reduction of these structures has been largely considered synapomorphic for Dromaeosauridae or Deinonychosauria as a whole, except for some taxa like Microraptorinae [86], *Austroraptor* [87], *Acheroraptor* [72], and *Archaeopteryx* [88, 89]. The larger of the preserved interdental plates of DMNH 21183 is triangular (like *Acheroraptor*), with a somewhat arched apex and a broader base.

The absence of medial crenulation or dorsoventral furrows in the interdental plate excludes it from assignment to Tyrannosauridae. The presence of interdental plates fused to the margin of the dentary is shared with *Atrociraptor* [90] and *Saurornitholestes* [43].

The lenticular (more mediolaterally compressed than circular) cross section of the teeth in DMNH 21183 differs from the mesial and lateral dentitions of many troodontids, which have a subcircular cross-sectional outline at the crown base [83]. The teeth of DMNH 21183 lack concave surfaces adjacent to both carinae (Fig 4), as has been observed in some mesial teeth of the troodontids *Troodon* [91, 92], *Urbacodon* [93], an indeterminate troodontid taxon from Uzbekistan [93], and the troodontid *Xixiasaurus* [83, 94] as reported by [83]. As in most paravians, the teeth preserved in DMNH 21183 exhibit short interdenticular sulci (opposite of the well-developed and deep sulci present in Abelisauridae, Tyrannosauridae, and Allosauroidea [9, 83], whereas short interdenticular sulci have been observed by Hendrickx et al. [83] in the microraptorine specimen IVPP V13476 [95], the eudromaeosaurians *Deinonychus* (YPM 5232), *Saurornitholestes* [43], and *Dromaeosaurus* (AMNH 5356; [92, 96], as well as in some troodontids [97, 98] such as *Troodon* (NHMUK PV R.12568).

The distal denticles have a relative higher density in contrast to the much larger denticles in Troodontidae, and are more similar to the condition in

Dromaeosauridae [83]. The presence of coarser distal denticles than mesial denticles is a more common feature found in saurornitholestine dromaeosaurids rather than Dromaeosaurinae [40]. An affinity to the sympatric cf. *Troodon* [26] can be excluded on the basis of serrated mesial teeth, which are not serrated in the troodontid [99]. An affinity with basal dromaeosaurid taxa like the halszkaraptorines and unenlagiines (e.g. *Halszkaraptor* [100], *Mahakala* [101], *Buitreraptor* [84] and *Austroraptor* [102]) can be excluded, as the dentition of these taxa are devoid of denticles [83]. Other deinonychosaurian taxa like the troodontids *Mei* [103], *Byronosaurus* [104], *Gobivenator* [105], *Urbacodon* [93], *Xixiasaurus* [94], IVPP V20378 and *Jinfengopteryx* [106], *Almas* [107, 108] and MPC-D 100-1128, the anchiornithid *Anchiornis* [109], *Eosinopteryx* [110], *Aurornis* [111], and the basal avialan *Archaeopteryx* (e.g., [112–114]) also have non-denticulate tooth crowns all along their jaws [83], excluding a potential affinity with DMNH 21183. It has to be cautioned though that independent reacquisition of denticulated teeth has been shown in some deinonychosaurian taxa like the anchiornithids *Caihong* and *Liaoningvenator* [83].

The small size of mesial denticles in DMNH 21183 is more similar to the condition in Saurornitholestinae rather than Dromaeosaurinae [43, 80, 115–118]. The shape of the denticles is slightly pointed toward the apex of the crown as in Dromaeosauridae and Troodontidae and not C- or U-shaped like in

Tyrannosauridae [83]. The reduction or lack of mesial denticulation is shared with some Asian Velociraptorinae like *Tsaagan* [85]. A similar condition to DMNH 21183 where mesial teeth bear unserrated mesial carinae and denticulated distal carinae is present in many other theropod clades. This feature is also seen in the dromaeosaurid *Tsaagan* [85], the troodontids *Linhevenator* [83, 119] and possibly *Saurornithoides* (AMNH 6516 [120]).

Although hooked denticles are primarily present in some derived troodontid taxa [83, 103], there are also dromaeosaurids that have apically hooked denticles (e.g., [80, 92, 96, 121]). These include the eudromaeosaurians *Atrociraptor* and *Saurornitholestes* [83, 92, 122]. An immediately noticeable difference between denticles in Dromaeosauridae and Troodontidae is that the latter tend to bear particularly large, bulbous, and widely separated denticles, while Dromaeosauridae have more numerous, smaller and asymmetrically convex or parallelogram-shaped denticles (Fig 4A; [83]). Other deinonychosaurian taxa lack apically hooked denticles, with a morphology that is more symmetrical and apically convex, such as in Microraptorinae and in some derived eudromaeosaurs such as *Acheroraptor*, *Bambiraptor*, *Linheraptor*, *Tsaagan*, *Utahraptor* and *Velociraptor* ([83] and references therein).

The number of denticles (12 to 13) in the partially exposed crowns of DMNH 21183 indicates that the total number of denticles per carina is greater than

the ~15 present in many troodontids, particularly in taxa more derived than *Sinovenator* [26]. As Hendrickx [83] pointed out, while some tooth crowns of *Saurornitholestes* appear to have less than 15 denticles on the carina [40, 92, 123] quantitative analyses by Larson and Currie [40] indicates that the large majority of *Saurornitholestes* teeth have many more than 15 denticles on the crown (for other remarks on morphological variation in the dental series of this taxon see [43]). Within Deinonychosauria, taxa with a large number of denticles (≥6 per 1 mm) include *Richardoestesia*, Saurornitholestinae (including *Saurornitholestes*), *Sinovenator*, and *Velociraptor* [40, 83, 124–127].

While the position of the mesial margin of the teeth preserved in DMNH 21183 and the degree of surface abrasion preclude detailed morphological observation, the finely serrated mesial carina, with denticles smaller than their distal serial homologues, can be seen particularly well in the 3rd tooth (d3; Fig 3D). The presence of distal denticles larger than mesial ones was long thought to characterize the dentition of Dromaeosauridae, and this criterion was used to identify velociraptorine teeth (e.g., [83, 115–118]). Teeth with fine mesial serrations are usually characterized by a denticle size index (DSDI: the ratio between number of mesial and distal denticles) higher than 1.2 while teeth that bear carinae with subequal denticle size have usually a DSDI close to 1. These arbitrary values were proposed by Rauhut and Werner [115] and corresponds in the

case of DSDI≈1.2 to approximately more than six mesial denticles for five distal serrations [83]. DMNH 21183 has a quite high DSDI (~2.3; Fig 9), which is well beyond the range of many deinonychosaurian taxa, and in the range of the most finely serrated saurornitholestine teeth (between 1 and 2.5; Fig 9). A DSDI >1.2 has been reported in the majority of eudromaeosaurians ([83] and references therein). Some lateral teeth of the troodontid *Zanabazar* [128] and some isolated crowns assigned to *Troodon* have also a very high DSDI (outliers with DSDI around 2-3; Fig 9 [40, 83, 91]. This is the opposite condition than that typically found in Dromaeosaurinae (DSDI ~ 1), like in the eponymous taxon *Dromaeosaurus* [83]. The surprisingly high DSDI of DMNH 21183 may be a juvenile trait, since many juvenile theropods have been shown to exhibit particularly fine mesial serrations relative to the distal ones, and evidence from tyrannosaurids [78, 129, 130] shows progressively decreasing DSDIs through ontogeny [135].

External textural features in dinosaur surface bone have been shown to change in relation to ontogeny [32], in a process mirroring internal microscopic remodeling [132]. For example, the skulls of ceratopsian dinosaurs show surface textural changes during ontogeny, from lightly striated to deeply rugose textures [30; 133]. Cortical bone texture with fine-grained, longitudinally striated pattern [31] is considered a size-independent criterion as an indication of relative

immaturity in non-avian archosaurs [30, 31, 134]. Lightly striated cortical bone grain express nascent ontogenetic characters in theropods [31], as it is particularly clear from *Tyrannosaurus* [31], *Scipionyx* [135], and *Juravenator* [136, 137]. The same striated, fibrous bone grain texture described in relatively immature individuals of these other theropod taxa is also present in DMNH 21183 (Figs 3A and 3B). This textural feature, in combination with the diminutive size of the specimen (Table 1), is evidence that DMNH 21183 is a juvenile.

After morphological, morphometric, and phylogenetic analyses, DMNH 21183 is here interpreted as specimen of Saurornitholestinae. The recurrent clustering of DMNH 21183 with Eurasian dromaeosaurids (a clade including Eudromaeosauria + Microraptorinae; Figs 5-6) and excluding Unenlaginae (in [33]), the sister-taxon relationship with *Saurornitholestes* (Fig 6A) in the analysis based on the dentition-only matrix from Hendrickx et al. [83], as well as in a eudromaeosaur clade more exclusive than that containing *Dromaeosaurus* in the analysis based on the tooth-crown-only matrix from Hendrickx et al. [33], all strengthen the saurornitholestine interpretation of the specimen based on morphological comparisons and teeth morphometrics.

Saurornitholestinae was erected by Longrich and Currie [14] as a subclade of Dromaeosauridae including *Saurornitholestes*, *Bambiraptor* and *Atrociraptor*. Not all phylogenetic analyses focused on deinonychosaurian interrelationships

recover this clade (e.g. [101, 138]), but others do [34, 37] or partially do so [139], and we recognize this clade. Saurornitholestinae is that lineage of eudromaeosaurs closer to *Saurornitholestes* than to *Dromaeosaurus* and *Velociraptor*, and this more inclusive, stem-based concept of the name is useful when discussing isolated dental material which has often been assigned to *Saurornitholestes* or cf. *Saurornitholestes* (e.g. [40, 96, 140]). There are several referrals to saurornitholestine dromaeosaurs in the latest Cretaceous of North America, including: the Milk River saurornitholestine [96] from the latest Santonian–earliest Campanian; the Early Campanian Menefee Formation saurornitholestine [41]; the Early Campanian Foremost Formation saurornitholestine [141]; and the Middle-Late Campanian Oldman Formation saurornitholestine [141] (Table S3).

More relevant for comparative purposes to taxa in the Prince Creek Formation, *Atrociraptor marshalli* was recovered in phylogenetic analyses of paravian interrelationships as a derived member of Dromaeosauridae and close to the node comprising *Saurornitholestes* (e.g. [37, 60]). In the dental-characters-only phylogenetic analysis, DMNH 21183 clusters separately from *Atrociraptor* (Fig 6A), while the crown-only-characters and full-osteological results recover both *Atrociraptor* and DMNH 21183 in a polytomy within Eudromaeosauria (Figs 5 and 6B). There are similarities in the apically-hooked denticles on the teeth of DMNH 21183, *Saurornitholestes,* and *Atrociraptor*, but the generally larger denticles of

the latter, particularly in the mesial carina [90, 142], sets it apart from *Saurornitholestes* and DMNH 21183. However, the implications of ontogenetic stage on relative denticle size should also be considered in this case. On the other hand, the interdental plates in DMNH 21183 more closely resemble those of *Atrociraptor* (TMP 95.166.1 [90]) than those of *Saurornitholestes* (TMP 1988.121.0039 [60]) in having a narrower base. *Atrociraptor* and *Saurornitholestes* are recovered in a sister-group relationship by Currie and Evans [60], and the shared similarities between DMNH 21183 and these two taxa may prove predictive should more complete dromaeosaurid material be found in the PCF.

Paleoecological and paleobiogeographical implications of a juvenile Arctic Saurornitholestinae

The discovery of dinosaur remains at high latitudes, and in particular the abundant dinosaur bone record from the PCF, challenged traditional reptilian models for dinosaurian physiologies and inspired debate centered on the potential for long-distance migrations by dinosaurs [20, 143–145]. Given the breadth of migration patterns in extant animals (e.g. [146]), to focus our discussion it is relevant to point out that these migrations for Arctic dinosaurs were inferred to cover latitudinal distances rather than trans-Arctic migrations, which are not even observed with animals today [147]. Increased subsequent interest showed, through

a variety of methods such as biomechanic, isotopic analyses, and osteohistology, that these dinosaurs likely had the necessary adaptations for overwintering in the ancient Arctic [20, 148, 149] and need not have moved to more southerly latitudes. Thus far, these discussions have focused almost exclusively on herbivorous taxa, with one exception that was based on the argument that if the dinosaurian prey did not migrate, then the predators were non-migratory as well [28].

In their review of the adaptive benefits of migration for modern mammals, Avgar and others [150] showed that in the terrestrial realm there is a decided preference for mammalian long-distance migrations to occur among large-bodied herbivores rather than carnivores. One of the suggested reasons for the rarity of migration by mammalian carnivores is that for purposes of energy consumption and mating, these animals need to establish, maintain, and defend territories. Such behavior would preclude the ability to migrate.

Dromaeosaurids were evolutionarily close to avians, and there is some discussion of the flight capabilities of some taxa within this group [151]. At best, some taxa may have been able to exhibit some rudimentary flight skills (e.g. *Sinornithosaurus* and *Zhenyuanlong* [152–154]). Further, the anatomy of most dromaeosaurids, especially larger species, argues for a flightless lifestyle [89]. That eliminates flight as an energy efficient means for a predatory animal to cover the vast distances required to migrate across large geographic areas.

The fibrous bone texture of DMNH 21183 strongly suggests that the individual was very young at the time of its death. Given that there is also a demonstrable positive correlation between tooth size and body size, at least in some theropods (such as dromaeosaurids, but does not apply to basal ornithomimosaurs and therizinosaurs with extremely tiny teeth compared to body size [Hendrickx pers. comm.]) and other diapsids [155, 156], it is reasonable to conclude that the DMNH 21183 belonged to a very small individual. Further, similar (if not stronger) biomechanical constraints for long-range migrations would apply for these small theropods, as has been advocated for ornithischian dinosaurs [157, 158]. Therefore, this specimen of a young, small individual suggests that dromaeosaurs likely nested in the ancient Arctic or in the close proximity, behavior different than long-distance migratory animals.

The identification of the first osteological remains attributed to Saurornitholestinae from the North Slope supports previous observations on the paleoecology and ecosystem structure of Late Cretaceous communities in Arctic Alaska. Previous workers suggested that gregarious herbivorous dinosaur groups inhabiting the Cretaceous Arctic were non-migratory [158]. The specimen described here (DMNH 21183) is particularly important because it represents a very small and young individual with little or limited ability to engage in long-distance travel or migrations. In modern migratory birds, it is often the case that

young birds lack experience and they must rely on learning migratory habits from the adults [159]. Also, as another analog, there is a decided preference for modern mammalian long-distance migrations to occur among large-bodied herbivores rather than carnivores. Taken together, we infer that DMNH 21183 implies a perennial residency of this dromaeosaur clade (Saurornitholestinae) in the Arctic.

DMNH 21183 adds further weight to the paleobiogeographical connection between closely related Asian and North American eudromaeosaur taxa (with sister clades present in both Asia and the Western Interior Basin of North America). DMNH 21183 is too fragmentary to provide more specific taxonomic distinction within the current record of known dromaeosaurids, but is most similar to saurornitholestines. Given the geological age of *Saurornitholestes langstoni* and *Atrociraptor marshalli*, and the wide-ranging tooth-form taxon *Richardoestesia* (Late Campanian-Late Maastrichtian), we predict that additional specimens and data may eventually provide evidence supporting the establishment of a new dromaeosaurid taxon in the Early to early Late Maastrichtian of Arctic Alaska (Fig 10).

Acknowledgments

Andrey Atuchin is acknowledged for the commissioned reconstruction in Fig 10. Thomas Carr (Carthage College, USA) and Christhophe Hendrickx

(Unidad Ejecutora Lillo, CONICET-Fundación Miguel Lillo) are also thanked for their thorough reviews which greatly improved the quality of this manuscript. We acknowledge Renata Tully (Nikon, Inc.) and Ryan Clubb (Keyence Corp. of America) for microscope and software support. The phylogenetic software TNT is available through the supporting and sponsorship of the Willi Hennig Society. The specimen used in this study was collected with funding from the National Science Foundation (OPP 0424594 to Fiorillo) and the National Geographic Society (W221-12 to Fiorillo). Additional funding was provided by the Perot Paleo Club. Lastly, the Arctic Management Unit of the Bureau of Land Management provided administrative support. The specimen discussed here was collected under BLM permit number AA-86864.

References

1. Matthew WD, Brown B. The family Deinodontidae, with notice of a new genus from the Cretaceous of Alberta. Bulletin of the American Museum of Natural History. 1922; 46: 367–385.
2. Colbert EH, Russell DA. The small Cretaceous dinosaur *Dromaeosaurus*. American Museum Novitates. 1969; 2380:1–49.
3. Sereno PC. A rationale for phylogenetic definitions, with application to the higher−level taxonomy of Dinosauria. Neues Jahrbuch für Geologie und Paläontologie Abhandlungen. 1998; 210: 41–83.
4. Ostrom JH. The ancestry of birds. Nature. 1973; 242:136.
5. Gauthier JA. Saurischian monophyly and the origin of birds. Memoires of the California Academy of Sciences. 1986; 8:1–55.
6. Metcalf SJ, Vaughan RF, Benton MJ, Cole J, Simms MJ, Dartnall DL. A new Bathonian (Middle Jurassic) microvertebrate site, within the Chipping Norton Limestone Formation at Hornsleaslow Quarry, Gloucestershire. Proceedings of the Geologists' Association. 1992; 103 (4): 321–342. doi:10.1016/S0016-7878(08)80129-0
7. Cau A. The assembly of the avian body plan: a 160-million-year long process. Bollettino della Società Paleontologica Italiana. 2018; 57 (1): 1–25. doi:10.4435/BSPI.2018.01

8. Hendrickx C., Hartman S. A., Mateus O. An overview of non-avian theropod discoveries and classification. PalArch's Journal of Vertebrate Palaeontology. 2015; 12 (1): 1-73.
9. Fanti F, Therrien F. Theropod tooth assemblages from the Late Cretaceous Maevarano Formation and the possible presence of dromaeosaurids in Madagascar. Acta Palaeontologica Polonica. 2007; 52 (1): 155–166.
10. Brown CM, Evans DC, Campione NE, O'Brien LJ, Eberth DA. Evidence for taphonomic size bias in the Dinosaur Park Formation (Campanian, Alberta), a model Mesozoic terrestrial alluvial- paralic system. Palaeogeography, palaeoclimatology, palaeoecology. 2013; 372, 108-122. doi: 10.1016/j.palaeo.2012.06.027
11. Cashmore DD, Butler RJ. Skeletal completeness of the non- avian theropod dinosaur fossil record. Palaeontology. 2019; 62: 951–981. doi:10.1111/pala.12436
12. Norell MA, Makovicky PJ. Important features of the dromaeosaurid skeleton II: information from newly collected specimens of *Velociraptor mongoliensis*, in American Museum Novitates. 1999; 3282: 1–45
13. Lü J, Brusatte SL. A large, short-armed, winged dromaeosaurid (Dinosauria: Theropoda) from the Early Cretaceous of China and its implications for feather evolution. Scientific Reports. 2015; 5: 11775. doi:10.1038/srep11775

14. Longrich NR, Currie PJ. A microraptorine (Dinosauria–Dromaeosauridae) from the Late Cretaceous of North America. PNAS. 2009; 106 (13): 5002–7. doi:10.1073/pnas.0811664106

15. Clemens WA, Allison CW. Late Cretaceous terrestrial vertebrate fauna, North Slope, Alaska. Geological Society of America Abstracts with Programs. 1985; 17(7):548.

16. Brouwers EM, Clemens WA, Spicer RA, Ager TA, Carter DL, Sliter WV. Dinosaurs on the North Slope of Alaska: high latitude, latest Cretaceous environments. Science. 1987; 237:1608–1610.

17. Davies K. L. Duck-bill dinosaurs (Hadrosauridae, Ornithischia) from the north slope of Alaska. Journal of Paleontology. 1987; 61: 198–200. doi: https://doi.org/10.1017/S0022336000028341

18. Gangloff RA, Fiorillo AR, Norton DW. The first pachycephalosaurine (Dinosauria) from the Arctic of Alaska and its paleogeographic implications. Journal of Paleontology. 2005; 79: 997–1001.

19. Fiorillo AR, Tykoski RS, Currie PJ, McCarthy PJ, Flaig P. Description of two partial *Troodon* braincases from the Prince Creek Formation (Upper Cretaceous), North Slope Alaska. Journal of Vertebrate Paleontology. 2009; 29 (1): 178–187. doi:10.1080/02724634.2009.10010370

20. Gangloff RA, Fiorillo AR. The record of Arctic dinosaurs from northern Alaska, paleogeographic and paleoecologic implications. Journal of Vertebrate Paleontology 2003; 23: 53A

21. Brown CM, Druckenmiller P. Basal ornithopod (Dinosauria: Ornithischia) teeth from the Prince Creek Formation (early Maastrichtian) of Alaska. Canadian Journal of Earth Sciences. 2011; 48 (9): 1342–1354. doi:10.1139/e11-017. ISSN 0008-4077

22. Fiorillo AR, Tykoski RS. A new Maastrichtian species of the centrosaurine ceratopsid *Pachyrhinosaurus* from the North Slope of Alaska. Acta Palaeontologica Polonica. 2012; 57(3): 561-573. doi: http://dx.doi.org/10.4202/app.2011.0033

23. Fiorillo AR, Tykoski RS. A diminutive new tyrannosaur from the top of the world. PLoS ONE. 2014; 9(3): e91287. doi:10.1371/journal.pone.0091287

24. Watanabe A, Erickson GM, Druckenmiller PS. An ornithomimosaurian from the upper Cretaceous Prince Creek Formation of Alaska. Journal of Vertebrate Paleontology. 2013; 33: 1169–1175.

25. Fiorillo AR. Alaska Dinosaurs: an Ancient Arctic World Book. CRC Press, Boca Raton. 2018; 224 p.

26. Fiorillo AR. On the occurrence of exceptionally large teeth of *Troodon* (Dinosauria: Saurischia) from the Late Cretaceous of northern Alaska. PALAIOS. 2008; 23:322–328.

27. Torices A, Currie PJ, Canudo JI, Pereda-Suberbiola X. Theropod dinosaurs from the Upper Cretaceous of the South Pyrenees Basin of Spain. Acta Palaeontologica Polonica. 2015; 60 (3): 611–626.

28. Fiorillo AR, Gangloff RA. Theropod teeth from the Prince Creek Formation (Cretaceous) of northern Alaska, with speculations on arctic dinosaur paleoecology. Journal of Vertebrate Paleontology. 2000; 20: 675–682.

29. Hendrickx C, Mateus O, Araújo R. A proposed terminology of theropod teeth (Dinosauria, Saurischia). Journal of Vertebrate Paleontology. 2015; 35: e982797. doi:10.1080/02724634.2015.982797

30. Sampson SD, Ryan MJ, Tanke DH. Craniofacial ontogeny in centrosaurine dinosaurs (Ornithischia: Ceratopsidae): taxonomic and behavioral implications. Zool. J. Linn. Soc. 1997; 121: 293–337. doi:10.1111/j.1096-3642.1997.tb00340

31. Carr TD. Craniofacial ontogeny in Tyrannosauridae (Dinosauria, Coelurosauria). Journal of Vertebrate Paleontology. 1999, 19: 497–520

32. Hone DWE, Farke AA, Wedel MJ. Ontogeny and the fossil record: what, if anything, is an adult dinosaur? Biology Letters. 2016; 12:20150947.

33. Lee MSY, Cau A, Naish D, Dyke GJ. Sustained miniaturization and anatomical innovation in the dinosaurian ancestors of birds. Science. 2014; 345(6196): 562-566.

34. Cau A, Brougham T, Naish D. The phylogenetic affinities of the bizarre Late Cretaceous Romanian theropod *Balaur bondoc* (Dinosauria, Maniraptora): dromaeosaurid or flightless bird? PeerJ. 2015; 3:e1032. https://doi.org/10.7717/peerj.1032

35. Hendrickx C, Tschopp E, Ezcurra M. d. Taxonomic identification of isolated theropod teeth: the case of the shed tooth crown associated with *Aerosteon* (Theropoda: Megaraptora) and the dentition of Abelisauridae. Cretaceous Research. 2020; 108: 104312. https://doi.org/10.1016/j.cretres.2019.104312.

36. Rauhut OWM, Carrano MT. The theropod dinosaur *Elaphrosaurus bambergi* Janensch, 1920, from the Late Jurassic of Tendaguru, Tanzania. Zoological Journal of the Linnean Society. 2016; 178 (3): 546-610

37. Brusatte S, Lloyd G, Wang S, Norell M. Gradual assembly of avian body plan culminated in rapid rates of evolution across the dinosaur–bird transition. Current Biology. 2014; 24:2386-2392.

38. Goloboff PA, Farris JS, Nixon KC. TNT, a free program for phylogenetic analysis. Cladistics. 2008; 24:774-786.

39. Gerke O, Wings O. Multivariate and cladistic analyses of isolated teeth reveal sympatry of theropod dinosaurs in the Late Jurassic of northern Germany. PLoS ONE. 2016; 11(7): e0158334. https://doi.org/10.1371/journal.pone.0158334
40. Larson DW, Currie PJ. Multivariate analyses of small theropod dinosaur teeth and implications for paleoecological turnover through time. PLoS ONE. 2013; 8:e54329. https://doi.org/10.1371/journal.pone.0054329
41. Williamson TE, Brusatte SL. Small theropod teeth from the Late Cretaceous of the San Juan Basin, northwestern New Mexico and their implications for understanding latest Cretaceous dinosaur evolution. PLoS ONE. 2014; 9(4): e93190. https://doi.org/10.1371/journal.pone.0093190
42. Brownstein CD. New records of theropods from the latest Cretaceous of New Jersey and the Maastrichtian Appalachian fauna. Royal Society Open Science. 2019; 6(11):191206.
43. Currie PJ, Evans DC. Cranial anatomy of new specimens of *Saurornitholestes langstoni* (Dinosauria, Theropoda, Dromaeosauridae) from the Dinosaur Park Formation (Campanian) of Alberta. The Anatomical Record. 2020; doi:10.1002/ar.24241
44. Moore MK. Sex estimation and assessment. In: Elizabeth A. DiGangi, Megan K. Moore, editors. Research Methods: Human Skeletal Biology.

Academic Press. 2013; pp. 91-116. doi: https://doi.org/10.1016/B978-0-12-385189-5.00004-2

45. Venables W, Ripley B. Modern Applied Statistics with S. 4th ed. New York: Springer. 2002; pp. 138.

46. Gryc G, Patton WW Jr, Payne TG. Present Cretaceous stratigraphic nomenclature of northern Alaska Journal of the Washington Academy of Sciences. 1951; 41: 159-167.

47. Mull GG, Houseknecht DW, Bird KJ. Revised Cretaceous and Tertiary stratigraphic nomenclature in the Colville Basin, northern Alaska: U.S. Geological Survey Professional Paper. 2003; 1673, 36.

48. Detterman RL, Bickel RS, Grvc G. Geology of the Chandler River region, Alaska. U.S., Geological Survey Professional Papers. 1963; 303-E:219-324.

49. Brosge WP, Whittington CL. Geology of the Umiat-Maybe Creek region, Alaska: U.S. Geological Survey Professional Paper. 1966; 303-H, 501–638.

50. Frederiksen NO, Ager TA, Edwards LE. Comment on "Early Tertiary marine fossils from northern Alaska: implications for Arctic Ocean paleogeography and faunal evolution": Geology. 1986; 14: 802-803.

51. Frederiksen NO. Sporomorph biostratigraphy, floral changes, and paleoclimatology, Eocene and earliest Oligocene of the eastern Gulf Coast U.S. Geological Survey Professional Paper. 1988; 1448: 68.

52. Frederiksen NO, Edwards LE, Ager TA, Sheehan TP. Palynology of Eocene strata in the Sagavanirktok and Canning Formations on the North Slope of Alaska. Palynology. 2002; 26: 59-93.
53. Parrish JT, Spicer RA. Late Cretaceous terrestrial vegetation: a near-polar temperature curve. Geology 1988; 16: 22–25.
54. Frederiksen NO. Pollen zonation and correlation of Maastrichtian marine beds and associated strata, Ocean Point dinosaur locality, North Slope, Alaska: U.S. Geological Survey Bulletin. 1991; 1990-E: E1-E24.
55. Brouwers EM, De Deckker P. Late Maastrichtian and Danian ostracode faunas from Northern Alaska: reconstructions of environment and paleogeography. Palaios. 1993; 8: 140–154.
56. Frederiksen NO, McIntyre DJ. Palynomorph biostratigraphy of mid(?)-Campanian to upper Maastrichtian strata along the Colville River, North Slope of Alaska: U.S. Geological Survey Open-File Report. 2000; 2000-493: 36.
57. Flores RM, Myers MD, Houseknecht DW, Stricker GD, Brizzolara DW, Ryherd TJ, Takahashi KI. Stratigraphy and facies of Upper Cretaceous Schrader Bluff and Prince Creek Formations in the Colville River Bluffs, North Slope, Alaska: U.S. Geological Survey Professional Paper. 2007; 1748: 52

58. Brandlen E. Paleoenvironmental reconstruction of the Late Cretaceous (Maastrichtian) Prince Creek Formation, near the Kikak-Tegoseak dinosaur quarry, North Slope, Alaska: University of Alaska Fairbanks, M.S. thesis. 2008; p. 225.

59. Fiorillo AR, McCarthy PJ, Flaig PP. Taphonomic and sedimentologic interpretations of the dinosaur-bearing Upper Cretaceous strata of the Prince Creek Formation, Northern Alaska: Insights from an ancient high-latitude terrestrial ecosystem. Palaeogeography, Palaeoclimatology, Palaeoecology, 2010; 295:376-388.

60. Conrad JE, McKee EH, Turrin BD. Age of tephra beds at the Ocean Point dinosaur locality, North Slope, Alaska, based on K-Ar and 40Ar/39Ar analyses. US Geological Survey Bulletin, 1992; 1990: 1–12.

61. Flaig PP, McCarthy PJ, Fiorillo AR. Anatomy, evolution, and paleoenvironmental interpretation of an ancient arctic coastal plain: Integrated paleopedology and palynology from the upper Cretaceous (Maastrichtian) Prince Creek Formation, North Slope, Alaska, USA: in Driese, S.G. and Nordt, L.C., eds.: New Frontiers in Paleopedology and Terrestrial Paleoclimatology, Paleosols and Soil Surface Analogue Systems, SEPM Special Publication. 2013; 114:179–230.

62. Flaig PP, Fiorillo AR, McCarthy PJ, Dinosaur-bearing hyperconcentrated flows of Cretaceous Arctic Alaska: Recurring catastrophic event beds on a distal paleopolar coastal plain: Palaios. 2014; 29: 594-611. doi: 10.2110/palo.2013.133.

63. Flaig PP, Hasiotis ST, Fiorillo AR. A paleopolar dinosaur track site in the Cretaceous (Maastrichtian) Prince Creek Formation of Arctic Alaska: Track characteristics and probable trackmakers: Ichnos. 2018; 25: 208–220. doi: 10.1080/10420940.2017.1337011

64. Flaig PP, McCarthy PJ, Fiorillo AR. A tidally influenced, high-latitude coastal-plain: the Upper Cretaceous (Maastrichtian) Prince Creek Formation, North Slope, Alaska, in Davidson S, Leleu S, North C. eds., From river to rock record: the preservation of fluvial sediments and their subsequent Interpretation: SEPM Special Publication. 2011; 97: 233–264.

65. Salazar-Jaramillo S, McCarthy PJ, Trainor T, Fowell SJ, Fiorillo AR. Origin of clay minerals in alluvial paleosols, Prince Creek Formation, North Slope, Alaska, USA: influence of volcanic ash on pedogenesis in the Late Cretaceous Arctic. Journal of Sedimentary Research. 2015; 85: 192-208.

66. Suarez CA, Flaig PP, Ludvigson GA, Gonzalez LA, Tian R, Zhou H, McCarthy PJ, Van der Kolk DA., Fiorillo AR. Reconstructing the paleohydrology of a Cretaceous Alaskan paleopolar coastal plain from stable

isotopes of bivalves. Paleogeography, Palaeoclimatology, Palaeoecology. 2016; 441, 339-351. doi: 10.1016/j.palaeo.2015.07.025.

67. Eberle JJ, Clemens WA, McCarthy PJ, Fiorillo AR, Erickson GM, Druckenmiller PS. Northern-most record of the Metatheria: a new Late Cretaceous pediomyid from the North Slope of Alaska. Journal of Systematic Palaeontology. 2019; 1–20, doi: 10.1080/14772019.2018.1560369.

68. van der Kolk DA, Flaig PP, Hasiotis ST. Paleoenvironmental reconstruction of a Late Cretaceous, muddy, river-dominated polar deltaic system: Schrader Bluff - Prince Creek Formation transition, Shivugak Bluffs, North Slope of Alaska, U.S.A: Journal of Sedimentary Research. 2015; 85: 903–936. doi: 10.2110/jsr.2015.58.

69. Phillips RL. Depositional environments and processes in Upper Cretaceous non-marine and marine sediments, Ocean Point dinosaur locality, North Slope, Alaska. Cretaceous Research. 2003; 24, 499–523.

70. Owen R. Report on British Fossil Reptiles. Report of the British Association for the Advancement of Science. 1842; 11: 60–204. Part 2.

71. Marsh OC. 1881. Classification of the Dinosauria. American Journal of Science 23:241–244

72. Evans DC, Larson DW, Currie PJ. 2013. A new dromaeosaurid (Dinosauria: Theropoda) with Asian affinities from the latest Cretaceous of North America. Naturwissenschaften. 100 (11): 1041-1049. doi: 10.1007/s00114-013-1107-5
73. Samman T, Powell GL, Currie PJ, Hills LV. Morphometry of the teeth of western North American tyrannosaurids and its applicability to quantitative classification. Acta Palaeontologica Polonica. 2005; 50: 757–776.
74. Buckley LG, Larson DW, Reichel M, Samman T. Quantifying tooth variation within a single population of *Albertosaurus sarcophagus* (Theropoda: Tyrannosauridae) and implications for identifying isolated teeth of tyrannosaurids. Canadian Journal of Earth Science. 2010; 47: 1227–1251.
75. Araújo R, Castanhinha R, Martins R, Mateus O, Hendrickx C, Beckmann F et al. Filling the gaps of dinosaur eggshell phylogeny: Late Jurassic theropod clutch with embryos from Portugal. Scientific Reports. 2013; 3: 1924. https://doi.org/10.1038/srep01924
76. Brusatte SL, Benson RBJ, Hutt S. The osteology of *Neovenator salerii* (Dinosauria: Theropoda) from the Wealden group (Barremian) of the Isle of Wight. Monograph of the Palaeontographical Society. 2008;162(631):1–166.

77. Xu X. Avian ancestors - a review of the phylogenetic relationships of the theropods Unenlagiidae, Microraptoria, *Anchiornis* and Scansoriopterygidae. Ameghiniana. 2013; 50(4), 469.

78. Currie PJ. Cranial anatomy of tyrannosaurid dinosaurs from the Late Cretaceous of Alberta, Canada. Acta Palaeontologica Polonica. 2003; 48 (2): 191–226

79. Brochu CR. Osteology of *Tyrannosaurus rex*: insights from a nearly complete skeleton and high-resolution computed tomographic analysis of the skull. Memoirs of the Society of Vertebrate Paleontology. 2003; 7: 1–138. doi: 10.2307/3889334.

80. Currie PJ. New information on the anatomy and relationships of *Dromaeosaurus albertensis* (Dinosauria: Theropoda). Journal of Vertebrate Paleontology. 1995; 15(3):576-591

81. Senter P. A new look at the phylogeny of Coelurosauria (Dinosauria: Theropoda.) Journal of Systematic Palaeontology. 2007, 5(4): 429-463

82. Choiniere JN, Clark JM, Forster CA, Xu X. A basal coelurosaur (Dinosauria: Theropoda) from the Late Jurassic (Oxfordian) of the Shishugou Formation in Wucaiwan, People's Republic of China, Journal of Vertebrate Paleontology. 2010; 30(6): 1773-1796, doi: 10.1080/02724634.2010.520779

83. Hendrickx C, Mateus O, Araújo R, Choiniere J. The distribution of dental features in non-avian theropod dinosaurs: Taxonomic potential, degree of homoplasy, and major evolutionary trends. Palaeontologia Electronica. 2019; 22.3.74: 1–110. doi: https://doi.org/10.26879/820

84. Gianechini FA, Makovicky PJ, Apesteguía S. The teeth of the unenlagiine theropod *Buitreraptor* from the Cretaceous of Patagonia, Argentina, and the unusual dentition of the Gondwanan dromaeosaurids. Acta Palaeontologica Polonica. 2011; 56:279-290.

85. Norell MA, Clark JM, Turner AH, Makovicky PJ, Barsbold R, Rowe T. 2006. A new dromaeosaurid theropod from Ukhaa Tolgod (Ömnögov, Mongolia). American Museum Novitates. 3545:1-51. doi: 10.1206/0003-0082(2006)3545[1:andtfu]2.0.co;2

86. Xu X, Wang X-L, Wu X-C. A dromaeosaurid dinosaur with a filamentous integument from the Yixian Formation of China. Nature 1999; 401 (6750): 262–266. doi:10.1038/45769

87. Agnolín FL, Novas FE. Unenlagiid theropods: are they members of the Dromaeosauridae (Theropoda, Maniraptora)? Anais da Academia Brasileira de Ciências. 2011;83(1):117–162. doi: 10.1590/s0001-37652011000100008.

88. Rauhut OWM, Foth C, Tischlinger H. The oldest *Archaeopteryx* (Theropoda: Avialiae): a new specimen from the Kimmeridgian/Tithonian boundary of Schamhaupten, Bavaria. PeerJ 2018; 6: e4191. doi:10.7717/peerj.4191
89. Turner AH, Pol D, Clarke J, Erickson G, Norell M. A basal dromaeosaurid and size evolution preceding avian flight. Science. 2007; 317:1378–1381.
90. Currie PJ, Varricchio DJ. A new dromaeosaurid from the Horseshoe Canyon Formation (Upper Cretaceous) of Alberta, Canada. 2004; 112–132. In Currie PJ, Koppelhus EB, Shugar MA and Wright JL. (eds.) Feathered Dragons. Indianapolis: Indiana University Press.
91. Currie PJ. 1987. Bird-like characteristics of the jaws and teeth of troodontid theropods (Dinosauria, Saurischia). Journal of Vertebrate Paleontology. 1987; 7: 72–81
92. Currie PJ, Rigby JK Jr, Sloan R. Theropod teeth from the Judith River Formation of southern Alberta, Canada; 1990; 107–125 in K. Carpenter and P. J. Currie (eds.), Dinosaur Systematics: Approaches and Perspectives. Cambridge University Press, New York.
93. Averianov AO, Sues H-D. A new troodontid (Dinosauria: Theropoda) from the Cenomanian of Uzbekistan, with a review of troodontid records from the territories of the former Soviet Union. Journal of Vertebrate Paleontology.

2007; 27 (1): 87–98. doi:10.1671/0272-4634(2007)27[87:ANTDTF]2.0.CO;2

94. Lü J, Xu L, Liu Y, Zhang X, Jia S, Ji Q. A new troodontid theropod from the Late Cretaceous of central China, and the radiation of Asian troodontids. Acta Palaeontologica Polonica, 2010; 55:381-388.

95. Xu X, Li F. A new microraptorine specimen (Theropoda: Dromaeosauridae) with a brief comment on the evolution of compound bones in theropods. Vertebrata PalAsiatica, 2016; 54:269-285.

96. Larson DW. Diversity and variation of theropod dinosaur teeth from the uppermost Santonian Milk River Formation (Upper Cretaceous), Alberta: a quantitative method supporting identification of the oldest dinosaur tooth assemblage in Canada. Canadian Journal of Earth Sciences. 2008; 45:1455-1468. doi: 10.1139/e08-070

97. Currie PJ, Dong Z. 2001. New information on *Shanshanosaurus huoyanshanensis*, a juvenile tyrannosaurid (Theropoda, Dinosauria) from the Late Cretaceous of China. Canadian Journal of Earth Sciences. 2991; 38:1729-1737. doi: 10.1139/e01-042

98. Sankey JT. Diversity of Latest Cretaceous (Late Maastrichtian) small theropods and birds: teeth from the Lance and Hell Creek Formations, USA. 2008; p. 117-134. In Sankey, JT, Baszio, S. (eds.), Vertebrate Microfossil

Assemblages: Their Role in Paleoecology and Paleobiogeography. Indiana University Press, Bloomington, Indiana.

99. Varricchio DJ, Horner JR, Jackson FD. Embryos and eggs for the Cretaceous theropod dinosaur *Troodon formosus*. Journal of Vertebrate Paleontology, 2002; 22:564-576. Doi: 10.1671/0272-4634(2002)022[0564:eaeftc]2.0.co;2

100. Cau A, Beyrand V, Voeten, D, Fernandez V, Tafforeau P, Stein K, et al. Synchrotron scanning reveals amphibious ecomorphology in a new clade of bird-like dinosaurs. Nature. 2017; 552 (7685): 395–399. doi: 10.1038/nature24679

101. Turner AH, Makovicky PJ, Norell M. A review of dromaeosaurid systematics and paravian phylogeny. Bulletin of the American Museum of Natural History. 2012; 371: 1–206. doi: 10.1206/748.1

102. Novas FE, Pol D, Canale JI, Porfiri JD, Calvo JO. A bizarre Cretaceous theropod dinosaur from Patagonia and the evolution of Gondwanan dromaeosaurids. Proceedings of the Royal Society B: Biological Sciences. 2009; 276:1101-1107. doi: 10.1098/rspb.2008.1554

103. Xu X, Norell MA. A new troodontid dinosaur from China with avian-like sleeping posture. Nature. 2004; 431:838-841. doi: 10.1038/nature02898

104. Makovicky PJ, Norell MA, Clark JM, Rowe T. Osteology and relationships of *Byronosaurus jaffei* (Theropoda: Troodontidae). American Museum Novitates. 2003; 3402:1-32. https://doi.org/10.1206/0003-0082(2003)402%3C0001:oarobj%3E2.0.co;2

105. Tsuihiji T, Barsbold R, Watabe M, Tsogtbaatar K, Chinzorig T, Fujiyama Y, Suzuki S. An exquisitely preserved troodontid theropod with new information on the palatal structure from the Upper Cretaceous of Mongolia. Naturwissenschaften. 2014; 101:131-142. doi: 10.1007/s00114-014-1143-9

106. Ji Q, Ji S, Lü J, You H, Chen W, Liu Y, Liu Y. First avialian bird from China. Geological Bulletin of China. 2005; 24:197-210.

107. Pei R, Li Q, Meng Q, Norell M, Gao K. 2017. New specimens of *Anchiornis huxleyi* (Theropoda, Paraves) from the late Jurassic of northeastern China. Bulletin of the American Museum of Natural History, 411:1–66. doi: 10.1206/0003-0090-411.1.1

108. Pei R, Norell MA, Barta DE, Bever GS, Pittman M, Xu X. Osteology of a new Late Cretaceous troodontid specimen from Ukhaa Tolgod, Ömnögovi Aimag, Mongolia. American Museum Novitates, 2017; 3889:1–47. doi: 10.1206/3889.1

109. Hu D, Hou L, Zhang L, Xu X. A pre-*Archaeopteryx* troodontid theropod from China with long feathers on the metatarsus. Nature, 2009; 461:640–643. doi: 10.1038/nature08322

110. Godefroit P, Demuynck H, Dyke G, Hu D, Escuillié F, Claeys P. Reduced plumage and flight ability of a new Jurassic paravian theropod from China. Nature Communications. 2013; 4:1394. doi: 10.1038/ncomms2389

111. Godefroit P, Cau A, Dong-Yu H, Escuillié F, Wenhao W, Dyke G. A Jurassic avialan dinosaur from China resolves the early phylogenetic history of birds. Nature. 2013; 498:359-362. doi: 10.1038/nature12168

112. Martin LD, Stewart JD, Whetstone KN. The origin of birds: structure of the tarsus and teeth. The Auk. 1980; 97:86-93.

113. Elzanowski A, Wellnhofer P. Cranial morphology of *Archaeopteryx*: evidence from the seventh skeleton. Journal of Vertebrate Paleontology. 1996; 16: 81-94. doi: 10.1080/02724634.1996.10011286

114. Mayr G, Pohl B, Hartman S, Peters DS. The tenth skeletal specimen of *Archaeopteryx*. Zoological Journal of the Linnean Society. 2007; 149:97-116. doi: 10.1111/j.1096-3642.2006.00245.x

115. Rauhut OWM, Werner C. First record of the family Dromaeosauridae (Dinosauria: Theropoda) in the Cretaceous of Gondwana (Wadi Milk

Formation, northern Sudan). Paläontologische Zeitschrift. 1995; 69:475-489. doi: 10.1007/BF02987808

116. Sweetman SC. 2004. The first record of velociraptorine dinosaurs (Saurischia, Theropoda) from the Wealden (Early Cretaceous, Barremian) of southern England. Cretaceous Research, 25:353–364. doi: 10.1016/j.cretres.2004.01.004

117. Vullo R, Néraudeau, D, Lenglet T. Dinosaur teeth from the Cenomanian of Charentes, Western France: evidence for a mixed Laurasian-Gondwanan assemblage. Journal of Vertebrate Paleontology. 2007; 27:931-943. doi10.1671/0272-4634(2007)27[931:dtftco]2.0.co;2

118. van der Lubbe T, Richter U, Knötschke N. Velociraptorine dromaeosaurid teeth from the Kimmeridgian (Late Jurassic) of Germany. Acta Palaeontologica Polonica. 2009; 54:401-408. doi: 10.4202/app.2008.0007

119. Xu X, Tan Q, Sullivan C, Han F, Xiao D. 2011. A short-armed troodontid dinosaur from the Upper Cretaceous of Inner Mongolia and its implications for troodontid evolution. PLoS ONE. 2011; 6:e22916. https://doi.org/10.1371/journal.pone.0022916

120. Norell MA, Makovicky PJ, Bever GS, Balanoff AM, Clark JM, Barsbold R, Rowe T. A review of the Mongolian Cretaceous dinosaur

Saurornithoides (Troodontidae: Theropoda). American Museum Novitates. 2009; 3654:1-63. https://doi.org/10.1206/648.1

121. Longrich N. Small theropod teeth from the Lance Formation of Wyoming, USA. 2008. In Sankey, J.T. and Baszio, S. editors. Vertebrate Microfossil Assemblages: Their Role in Paleoecology and Paleobiogeography. Indiana University Press, Bloomington, Indiana. pp. 135-158.

122. Sankey JT. Late Campanian southern dinosaurs, Aguja Formation, Big Bend, Texas. Journal of Paleontology. 2001; 75:208-215. doi: 10.1017/s0022336000031991

123. Baszio S. Systematic palaeontology of isolated dinosaur teeth from the latest Cretaceous of south Alberta, Canada. Courier Forschungsinstitut Senckenberg. 1997; 196:33-77.

124. Smith JB, Vann DR, Dodson P. Dental morphology and variation in theropod dinosaurs: Implications for the taxonomic identification of isolated teeth. The Anatomical Record Part A: Discoveries in Molecular, Cellular, and Evolutionary Biology. 2005; 285A:699-736. doi: 10.1002/ar.a.20206

125. Zanno LE. A taxonomic and phylogenetic re-evaluation of Therizinosauria (Dinosauria: Maniraptora). Journal of Systematic Palaeontology. 2010; 8:503-543. doi: 10.1080/14772019.2010.488045

126. Rauhut OWM, Foth C, Tischlinger H, Norell MA. Exceptionally preserved juvenile megalosauroid theropod dinosaur with filamentous integument from the Late Jurassic of Germany. Proceedings of the National Academy of Sciences. 2012; 109:11746-11751. doi: 10.1073/pnas.1203238109

127. Choiniere JN, Clark JM, Forster CA, Norell MA, Eberth DA, Erickson GM, et al. A juvenile specimen of a new coelurosaur (Dinosauria: Theropoda) from the Middle-Late Jurassic Shishugou Formation of Xinjiang, People's Republic of China. Journal of Systematic Palaeontology. 2014; 12:177-215. doi: 10.1080/14772019.2013.781067

128. Barsbold R. Saurornithoididae, a new family of small theropod dinosaurs from central Asia and North America. Palaeontologia Polonica, 1974; 30:5–22.

129. Currie PJ, Dong Z. New information on Cretaceous troodontids (Dinosauria, Theropoda) from the People's Republic of China. Canadian Journal of Earth Sciences. 2001; 38:1753–1766. doi: 10.1139/e01-065

130. Tsuihiji T, Watabe M, Tsogtbaatar K, Tsubamoto T, Barsbold R, Suzuki S et al. Cranial osteology of a juvenile specimen of *Tarbosaurus bataar* (Theropoda, Tyrannosauridae) from the Nemegt Formation (Upper Cretaceous) of Bugin Tsav, Mongolia. Journal of Vertebrate Paleontology. 2011; 31:497-517. https://doi.org/10.1080/02724634.2011.557116

131. Carr TD, Williamson TE. Diversity of late Maastrichtian Tyrannosauridae (Dinosauria: Theropoda) from western North America. Zoological Journal of the Linnean Society. 2004; 142:479-523. doi: 10.1111/j.1096-3642.2004.00130.x

132. Tumarkin-Deratzian AR. Histological evaluation of ontogenetic bone surface texture changes in the frill of *Centrosaurus apertus*. In New Perspectives on Horned Dinosaurs, the Royal Tyrrell Museum Ceratopsian Symp. Bloomington, Indiana University Press. 2010; 251–263.

133. Brown CM, Russell AP, Ryan MJ. Pattern and transition of surficial bone texture of the centrosaurine frill and their ontogenetic and taxonomic implications. Journal of Vertebrate Paleontology. 2009; 29: 132–141. doi:10.1671/039.029.0119

134. Bennett SC. The ontogeny of *Pteranodon* and other pterosaurs. Paleobiology. 1993; 19 (1): 92–106.

135. Dal Sasso C, Maganuco S. *Scipionyx samniticus* (Theropoda: Compsognathidae) from the Lower Cretaceous of Italy: Osteology, ontogenetic assessment, phylogeny, soft tissue anatomy, taphonomy, and palaeobiology, Memorie della Società italiana di Scienze naturali e del Museo civico di Storia naturale di Milano, 2011; 34 (1).

136. Göhlich UB, Chiappe LM. A new carnivorous dinosaur from the Late Jurassic Solnhofen archipelago. Nature. 2006; 440 (7082): 329–332. doi:10.1038/nature04579.

137. Chiappe LM, Göhlich, U.B. Anatomy of *Juravenator starki* (Theropoda: Coelurosauria) from the Late Jurassic of Germany. Neues Jahrbuch für Geologie und Paläontologie - Abhandlungen. 2010; 258 (3): 257–296. doi:10.1127/0077-7749/2010/0125

138. Hartman S, Mortimer M, Wahl WR, Lomax DR, Lippincott J, Lovelace DM. A new paravian dinosaur from the Late Jurassic of North America supports a late acquisition of avian flight. PeerJ. 2019; 7:e7247 https://doi.org/10.7717/peerj.7247

139. Jasinski SE, Sullivan RM, Dodson P. New dromaeosaurid dinosaur (Theropoda, Dromaeosauridae) from New Mexico and biodiversity of dromaeosaurids at the end of the Cretaceous. Scientific Reports, 2020; 10, 5105. https://doi.org/10.1038/s41598-020-61480-7

140. Fanti F, Miyashita T. A high latitude vertebrate fossil assemblage from the Late Cretaceous of west–central Alberta, Canada: evidence for dinosaur nesting and vertebrate latitudinal gradient. Palaeogeography, Palaeoclimatology, Palaeoecology. 2009; 275(1–4):37–53.

141. Peng J, Russell AP, Brinkman DB. Vertebrate microsite assemblages (exclusive of mammals) from the Foremost and Oldman Formations of the Judith River Group (Campanian) of southeastern Alberta: an illustrated guide. Provincial Museum of Alberta, Natural History Occasional Paper. 2001; 25:1–54.

142. Larson, D. W.; Brinkman, D. B.; Bell, P. R. Faunal assemblages from the upper Horseshoe Canyon Formation, an early Maastrichtian cool-climate assemblage from Alberta, with special reference to the *Albertosaurus sarcophagus* bonebed. Canadian Journal of Earth Sciences. 2010; 47 (9): 1159–1181. doi:10.1139/e10-005

143. Russell DA. The environments of Canadian dinosaurs. Canadian Geographical Journal. 1973; 87: 4–11.

144. Paul GS. Migration. In The Encyclopedia of Dinosaurs, Edited by: Currie P, Padian K. 1997; 444–446. San Diego, CA: Academic Press

145. Bell PR, Snively E. Polar dinosaurs on parade: a review of dinosaur migration, Alcheringa: An Australasian Journal of Palaeontology. 2008; 32(3): 271–284, doi: 10.1080/03115510802096101

146. Rappole, J.H. The avian migrant: the biology of bird migration. Columbia University Press. 2013.

147. Gudmundsson, G.A. and Alerstam, T. Why is there no transpolar bird migration? Journal of Avian Biology. 2018; 93-96.

148. Gangloff RA, Fiorillo AR. Taphonomy and paleoecology of a bonebed from the Prince Creek Formation, North Slope, Alaska. Palaios. 2010; 25: 299–317.

149. Suarez CA, Ludvigson GA, Gonzalez LA, Fiorillo AR, Flaig PP, McCarthy PJ. 2013. Use of multiple isotope proxies for elucidating Arctic Cretaceous paleohydrology. In Bojar AV, Melinte-Dobrinescu MC, Smit J. editors. Isotopic Studies in Cretaceous Research: Geological Society of London Special Publication. 2013; 382:185–202.

150. Avgar T, Street G, Fryxell JM, On the adaptive benefits of mammal migration. Canadian Journal of Zoology. 2014; 92(6): 481-490.

151. Xu X, Zhou Z, Wang X, Kuang X, Zhang F, Du X. Four winged dinosaurs from China. Nature. 2003; 421:335–340.

152. Dyke G, de Kat R, Palmer C, van der Kindere J, Naish D, Ganapathisubramani, B. Aerodynamic performance of the feathered dinosaur *Microraptor* and the evolution of feathered flight. Nature Communications. 2013; 4 (2489). doi:10.1038/ncomms3489

153. Alexander, D. E., Gong, E., Martin, L. D., Burnham, D. A. & Falk, A. R. Model tests of gliding with different hindwing configurations in the four-winged dromaeosaurid *Microraptor gui*. Proceedings of the National Academy of Science. 2010; 107, 2972-2976.

154. Xu X, Wang X-L, Wu X-C. A dromaeosaurid dinosaur with filamentous integument from the Yixian Formation of China. Nature. 1999; 401:262–266.

155. Mateo JA, Lopez- Jurado LF. Dental ontogeny in *Lacerta lepida* (Sauria, Lacertidae) and its relationship to diet. Copeia. 1997; 461–463.

156. Townsend V, Akin J, Felgenhauer B, Dauphine J, Kidder S. Dentition of the ground skink, *Scincella lateralis* (Sauria, Scincidae). Copeia. 1999; (3): 783–788. doi:10.2307/1447615

157. Horner JR. Evidence of colonial nesting and 'site fidelity' among ornithischian dinosaurs. Nature. 1982; 297(5868):675-676.

158. Fiorillo AR, Gangloff RA. The caribou migration model for Arctic hadrosaurs (Ornithischia: Dinosauria): a reassessment. Historical Biology. 2001; 15:323-334.

159. Baker RR. The significance of the lesser black-backed gull to models of bird migration. Bird Study, 1980; 27(1), pp.41-50.

Figure captions

Figure 1. Locality map (A) of Pediomys point (red star) in the North Slope of Alaska, USA. Coordinates: N 70.018667°, W 151.591488°. Stratigraphic sections schematised in Fig 2 are reported here in B, with PdP representing the fossil bone-bearing section.

Figure 2. Geological setting. Stratigraphic sections at Pediomys point. Black star represents DMNH 21183.

Figure 3. DMNH 21183. Anterior portion of a dromaeosaurid dentary in lateral (A), medial (B), dorsal view (C) views and close up of the interdental plates and 3rd tooth in medial view (D). Curved arrows represent features hidden on that view. Abbreviations: a2, 2nd alveolus; a3. 3rd alveolus; d2, 2nd dentary tooth; d3, 3rd dentary tooth; d4, 4th dentary tooth; ave, antero-ventral process; idp, interdental plate; lr, lateral ridge; mg, Meckelian groove; mf, Meckelian foramina. Scale bar: 2 mm.

Figure 4. Closeup on the dentary teeth and lateral ridge in DMNH 21183. Detail of the 2nd dentary tooth highlighting the distal carina under normal light (A) and fluorescent microscopy (B). Details of the distal denticles (C) and

close-up of the lateral ridge (lr) close to the alveolar margin in lateral views (D). Abbreviation: lr, lateral ridge. Scale bar: 0.5 mm.

Figure 5. Phylogenetic position of DMNH 21183. Strict consensus topology of the shortest trees recovered by the parsimony analyses of the phylogenetic dataset of Lee et al. [33] with the addition of DMNH 21183 (384 MPTs, 6043 steps, CI= 0.244, RI= 0.587). The main clades of Theropoda outside Deinonychosauria are collapsed for space constraints. Full topology available in S4 Fig. Numbers adjacent to nodes are the bootstrap values. Red box highlights the node containing DMNH 21183 (red arrow).

Figure 6. Phylogenetic position of DMNH 21183 in Hendrickx et al. [35]. Strict consensus topology of the shortest trees recovered by the parsimony analyses showing the position of DMNH 21183 in (A) the dentition-only character matrix from Hendrickx et al. [35] (2 MPTs, 1314 steps, CI= 0.194, RI= 0.418), and (B) the tooth-crown-only character matrix from Hendrickx et al. [35] (5 MPTs, 867 steps, CI = 0.183, RI = 0.439). The overall topology was constrained in both analyses, with DMNH 21183 allowed to float. The main clades of Theropoda outside Deinonychosauria are collapsed for space constraints. Full topology

available in S5-S6 Figs. Numbers adjacent to nodes are the bootstrap values. Red box highlights the node containing DMNH 21183 (red arrow) in Eudromaeosauria.

Figure 7. Position of DMNH 21183 in the theropod teeth morphospace.

Principal components analysis of theropod teeth morphospace based on the dataset from Gerke and Wings [39]. DMNH 21183 is marked by a yellow star. Abbreviations: crown height (CH), crown basal width (CBW), ratio between mesial (anterior) and distal (posterior) denticles (DSDI). Morphometric dataset prepared as explained in methods and including measurements from DMNH 21183 reported in Supplementary Information (S2 Dataset).

Figure 8. Position of DMNH 21183 in the paravian teeth morphospace.

Principal components analysis of theropod teeth morphospace based on the dataset from Larson and Currie [30]. DMNH 21183 is marked by a blue star. Abbreviations: fore-aft basal length (FABL), crown height (CH), basal width (BW), anterior (ADM) and posterior denticles per millimeter (PDM). Morphometric dataset prepared as explained in methods and including measurements from DMNH 21183 reported in Supplementary Information (Dataset S2).

Figure 9. Denticle size index comparison between deinonychosaurian taxa. Box plot showing a comparison of the relative denticle size index (DSDI) between DMNH 21183 and four clades of deinonychosaurian theropods from the dataset in Larson and Currie [30]. DMNH 21183 is marked as a star. Dots represent outlier of each clade's distribution.

Figure 10. Life reconstruction of the Alaskan saurornitholestine in its environment. Artistic restoration by scientific illustrator Andrey Atuchin depicts a riparian setting in the Prince Creek Formation, matching the geological evidence described in this paper. DMNH 21183 comes from the juvenile dromaeosaurid on the branch close to the adult, while a subadult (foreground) stalks an individual of *Unnuakomys hutchisoni* [67], a methatherian known from this locality. Individuals of the sympatric ceratopsid *Pachyrhinosaurus perotorum* [22] rest in the background.

Table 1. Measurements of DMNH 21183.

Table 2. Coefficients of the PCA analysis run on the theropod teeth dataset published by Gerke and Wings [39].

Table 3. Coefficients of the PCA analysis run on the deinonychosaurian teeth dataset published by Larson and Currie [30].

Supporting information

S1 Fig. Magnified close-up views of the 2nd tooth in DMNH 21183. Close-up of the mesial carina in anterior view (A, C), highlighting the denticle-bearing anterior carina (ac). Magnified lingual view of the tooth (B) highlighting the anterior (ac) and posterior (pc) carinae (D). Dotted line (C) highlights the interdenticular sulci. Scale bar: 100 µm.

S2 Fig. Discriminant Functional Analysis of DMNH 21183 in Gerke and Wings [39]. Discriminant Functional Analysis of DMNH 21183 in the theropod teeth morphospace generated with the morphometric dataset provided in Gerke and Wings [39]. Abbreviations: LD, linear dimension. DMNH 21183 indicated by a green star.

S3 Fig. Discriminant Functional Analysis of DMNH 21183 in Larson and Currie [40]. Discriminant Functional Analysis of DMNH 21183 in the deinonychosaurian teeth morphospace generated with the morphometric dataset provided in Larson and Currie [40]. Abbreviations: LD, linear dimension. DMNH 21183 indicated by a pink star.

S4 Fig. Phylogenetic position of DMNH 21183 in Lee et al. [33]. Strict consensus topology of the shortest trees recovered by the parsimony analyses showing the position of DMNH 21183 in the matrix from Lee et al. [33] (384 MPTs, 6043 steps, CI= 0.244, RI= 0.587). Numbers adjacent to nodes are the bootstrap values. Red box highlights the node containing DMNH 21183 (red arrow).

S5 Fig. Phylogenetic position of DMNH 21183 in Hendrickx et al. [35] Strict consensus topology of the shortest trees recovered by the parsimony analyses showing the position of DMNH 21183 in the dentition-only character matrix from Hendrickx et al. [35] (2 MPTs, 1314 steps, CI= 0.194, RI= 0.418). The overall topology was constrained with DMNH 21183 allowed to float. Numbers adjacent to nodes are the bootstrap values. Red box highlights the node containing DMNH 21183 (red arrow) in Eudromaeosauria.

S6 Fig. Phylogenetic position of DMNH 21183 in Hendrickx et al. [35] Strict consensus topology of the shortest trees recovered by the parsimony analyses showing the position of DMNH 21183 in the tooth-crown-only character matrix from Hendrickx et al. [35] (5 MPTs, 867 steps, CI = 0.183, RI = 0.439). The overall topology was constrained with DMNH 21183 allowed to float. Numbers

adjacent to nodes are the bootstrap values. Red box highlights the more inclusive node containing DMNH 21183 (red arrow) in Eudromaeosauria.

S1 Table. Systematics used in this study. Systematics names and relative phylogenetic definition used in this study.

S1 Dataset. Phylogenetic character scoring of DMNH 21183. Character scoring in the phylogenetic matrices from Lee et al. [33] and Hendrickx et al. [35].

S2 Dataset. Morphometric data. Morphometric scoring for DMNH 21183 and modified datasets for multivariate analyses (PCA and DFA) in Gerke and Wings [39] and Larson and Currie [40].

S3 Dataset. Latest Cretaceous dromaeosaurids in North America. Faunal list compilation of all dromaeosaurid taxa in the latest Cretaceous (84.5–66.043 million years ago) with geographic, stratigraphic, chronological, and literature information attached.

Figure 1

Click here to access/download;Figure;Figure 1.tif

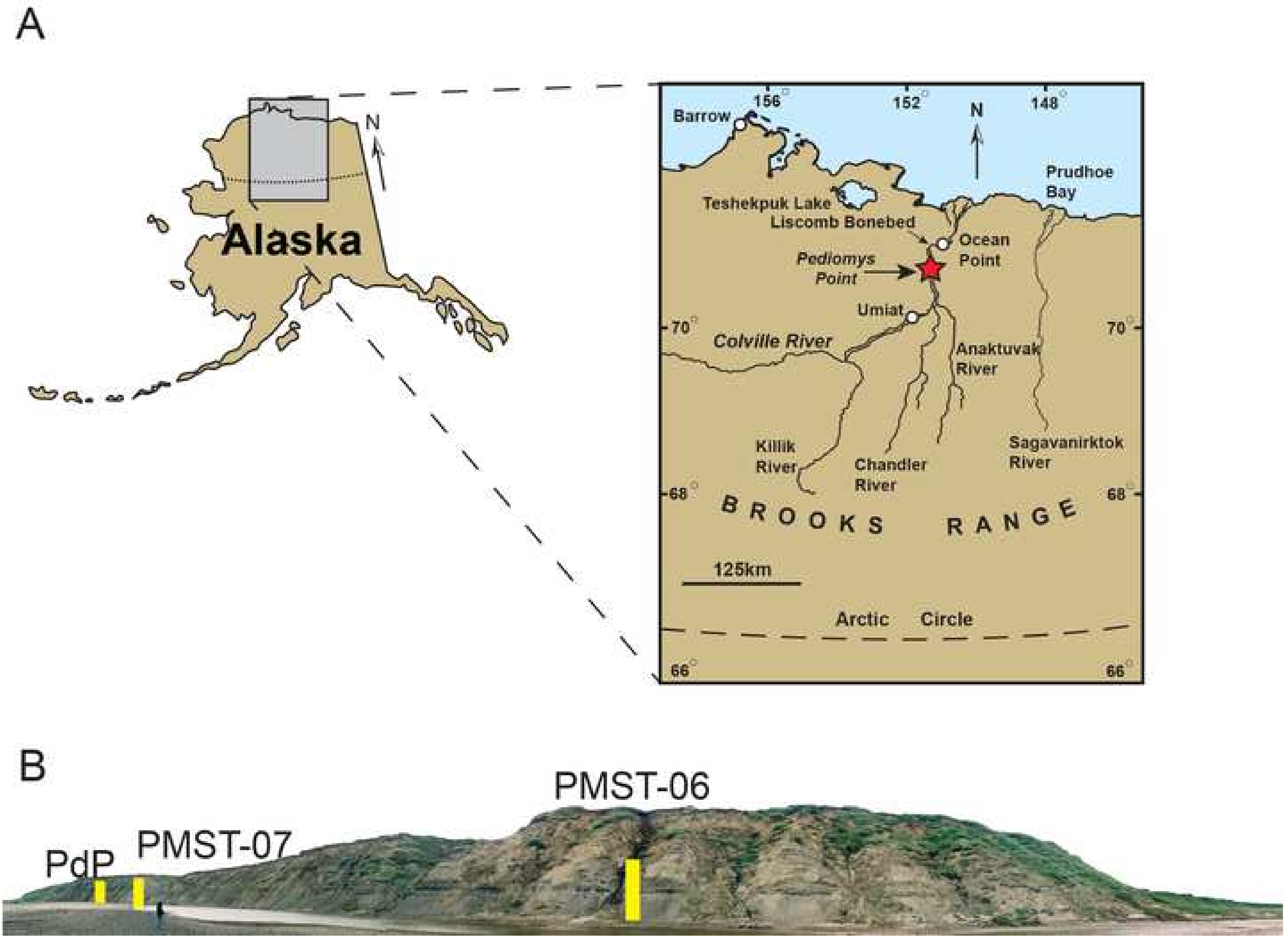

Figure 2 Click here to access/download;Figure;Figure 2.tif

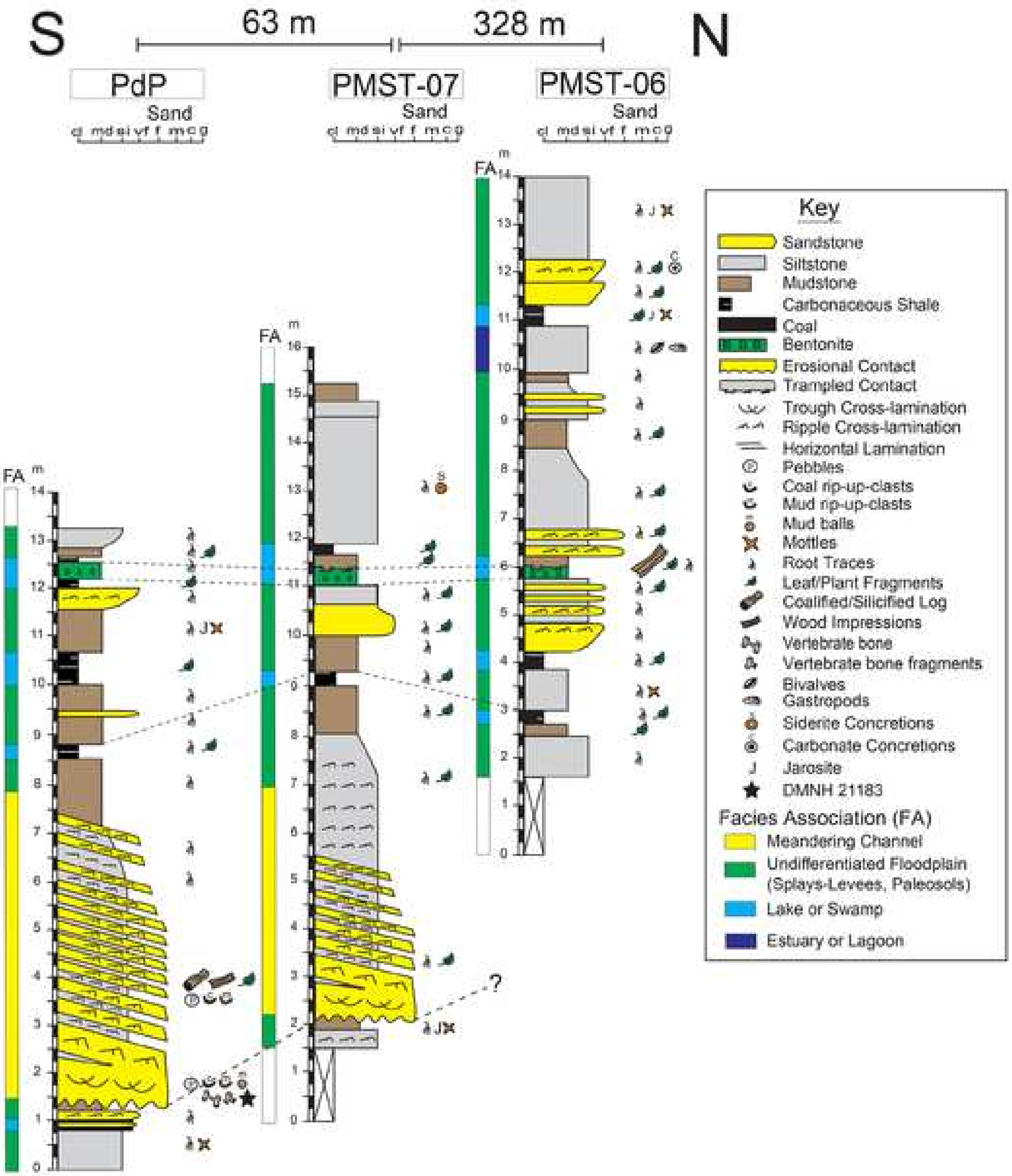

Figure 3 Click here to access/download;Figure;Figure 3.tif

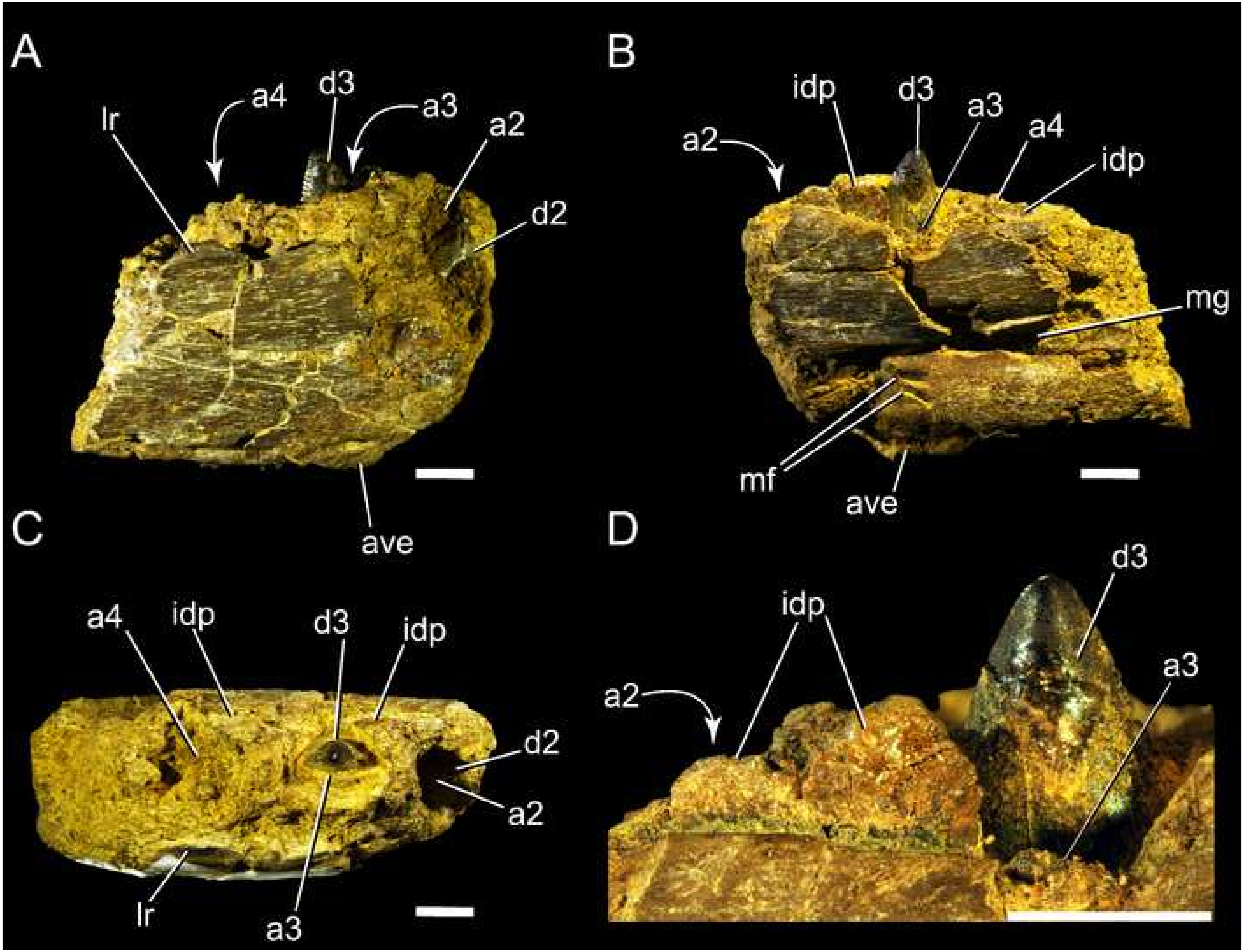

Figure 4

Click here to access/download;Figure;Figure 4.tif

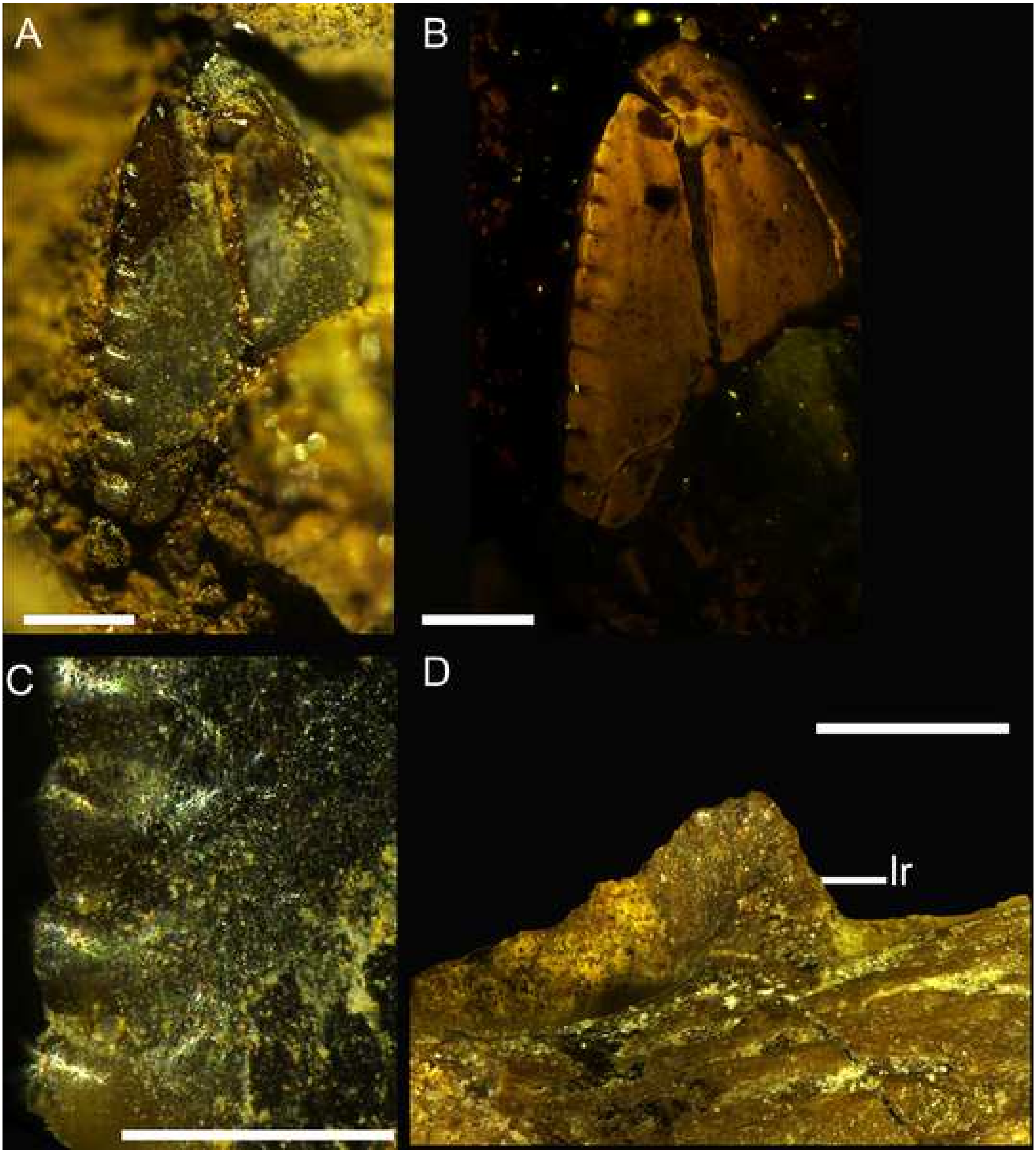

Figure 5

Click here to access/download;Figure;Figure 5.tif

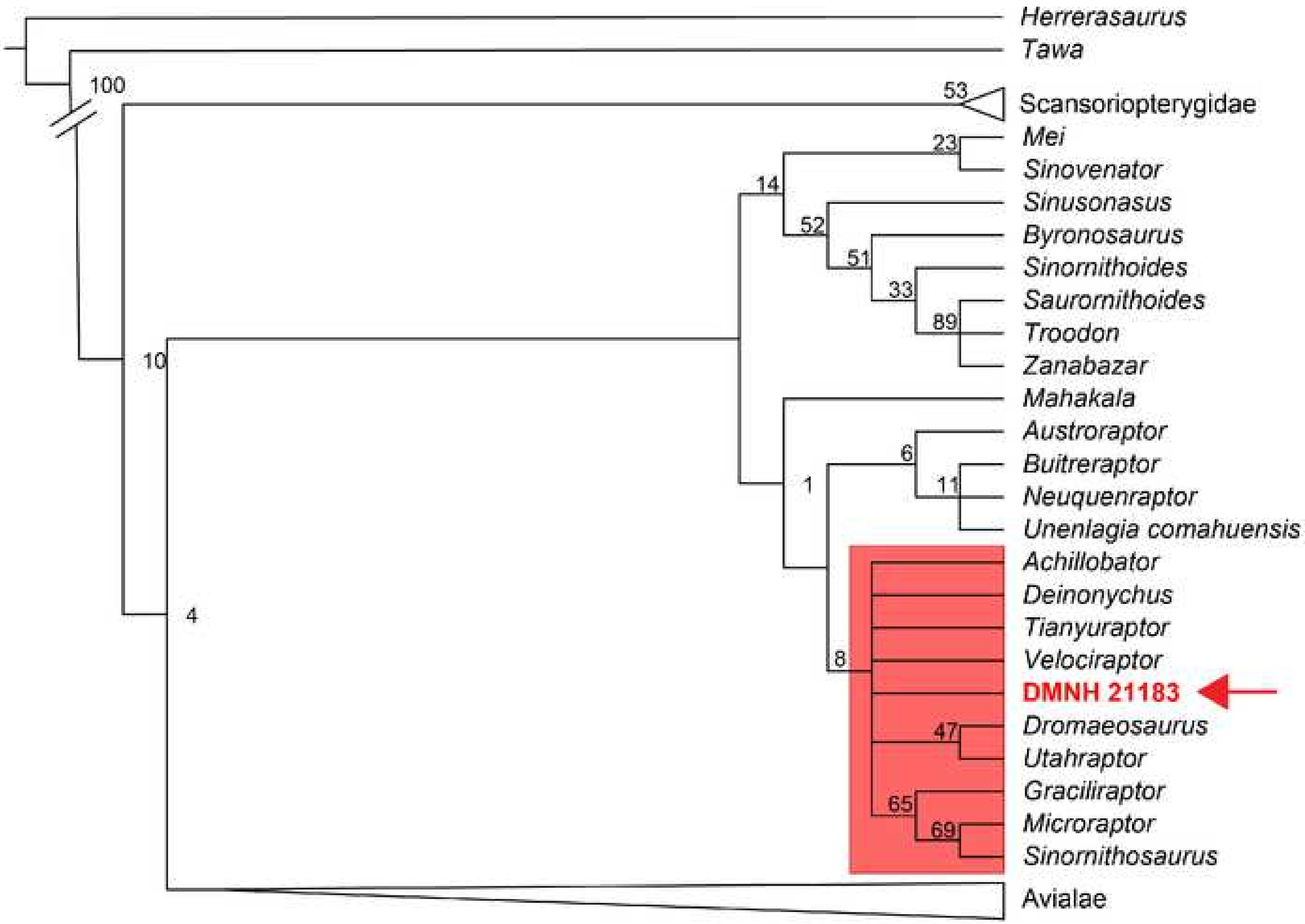

Figure 6 Click here to access/download;Figure;Figure 6.tif

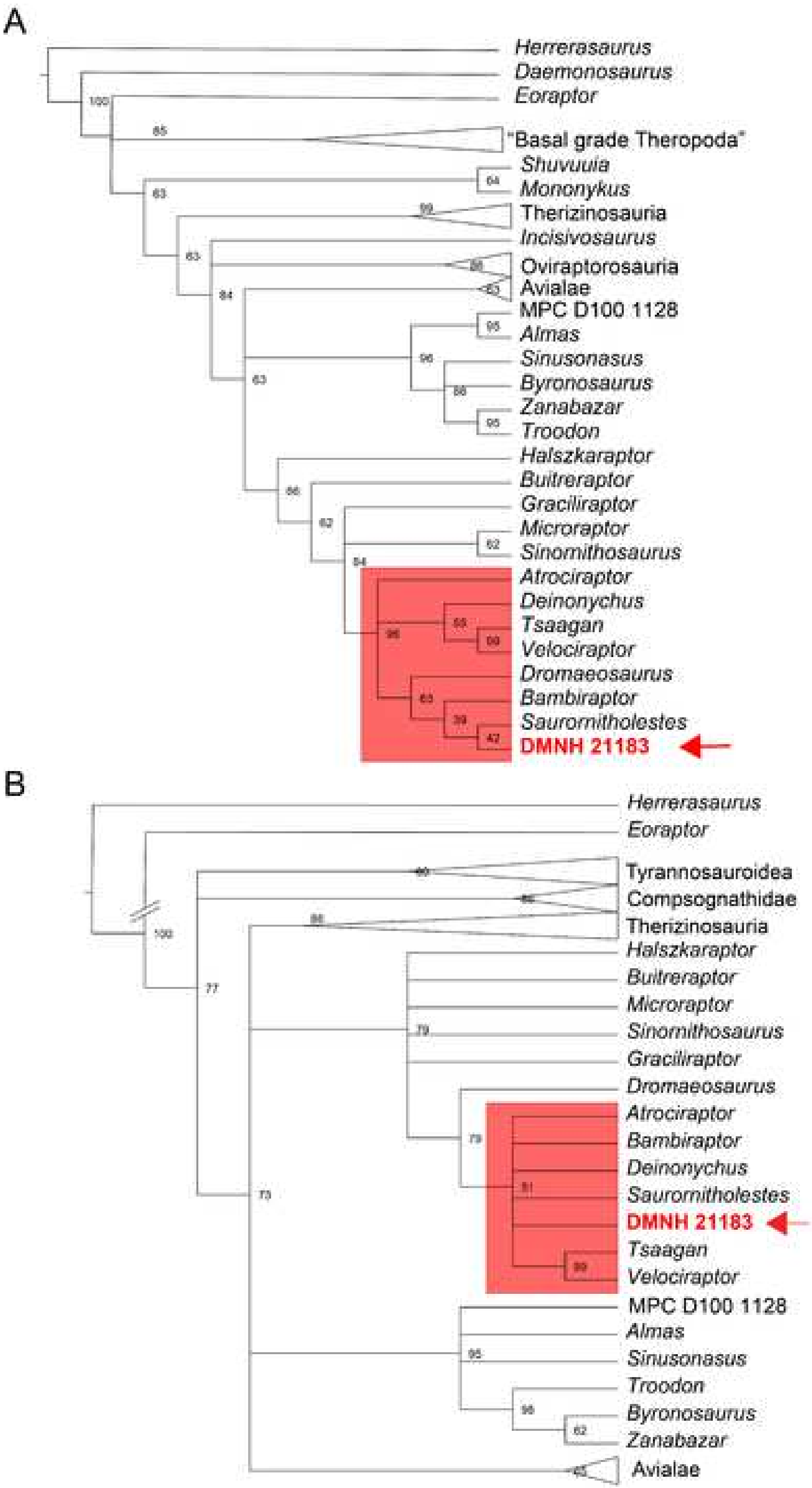

Figure 7

Click here to access/download;Figure;Figure 7.tif

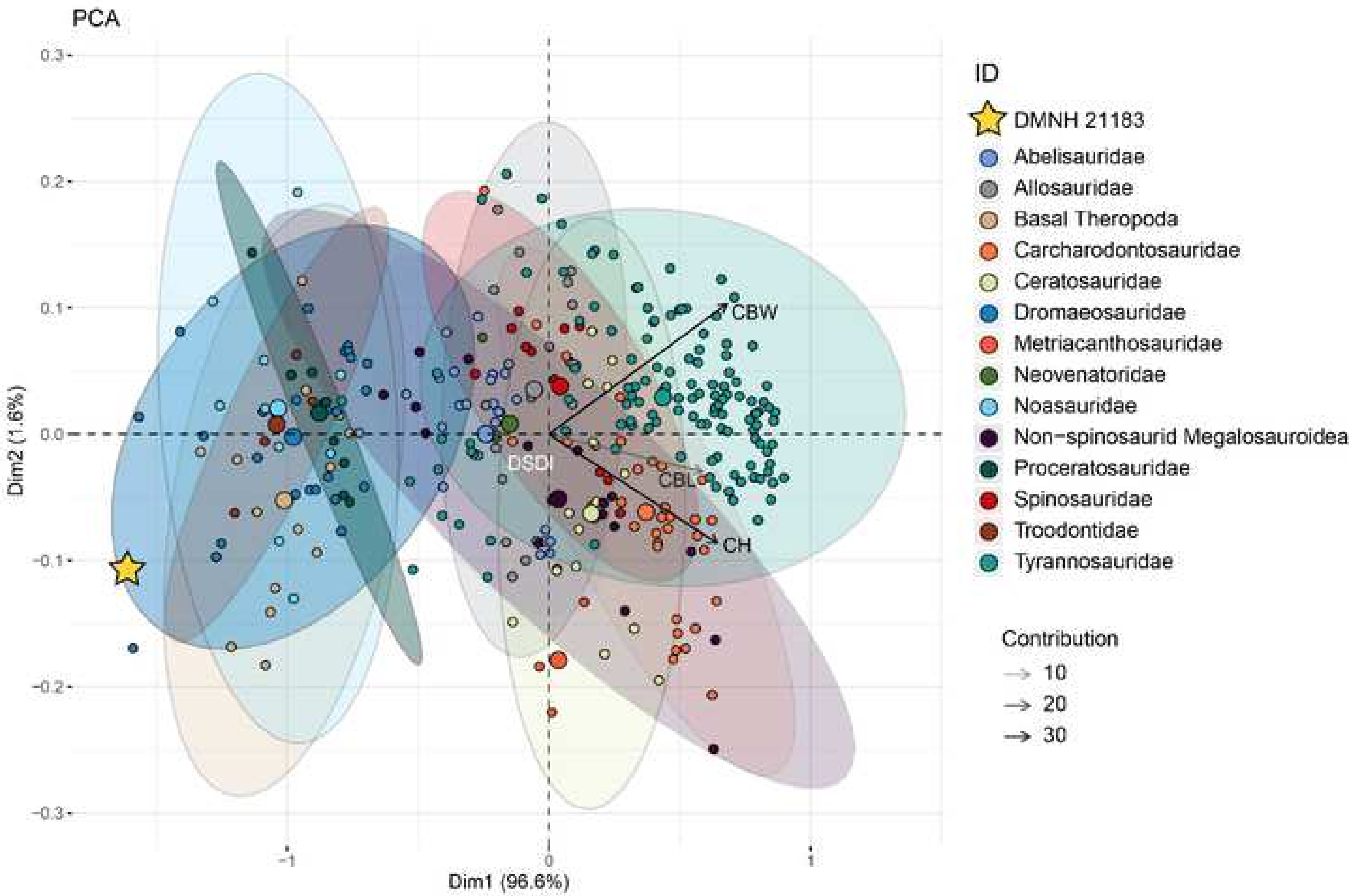

Figure 8

Click here to access/download;Figure;Figure 8.tif

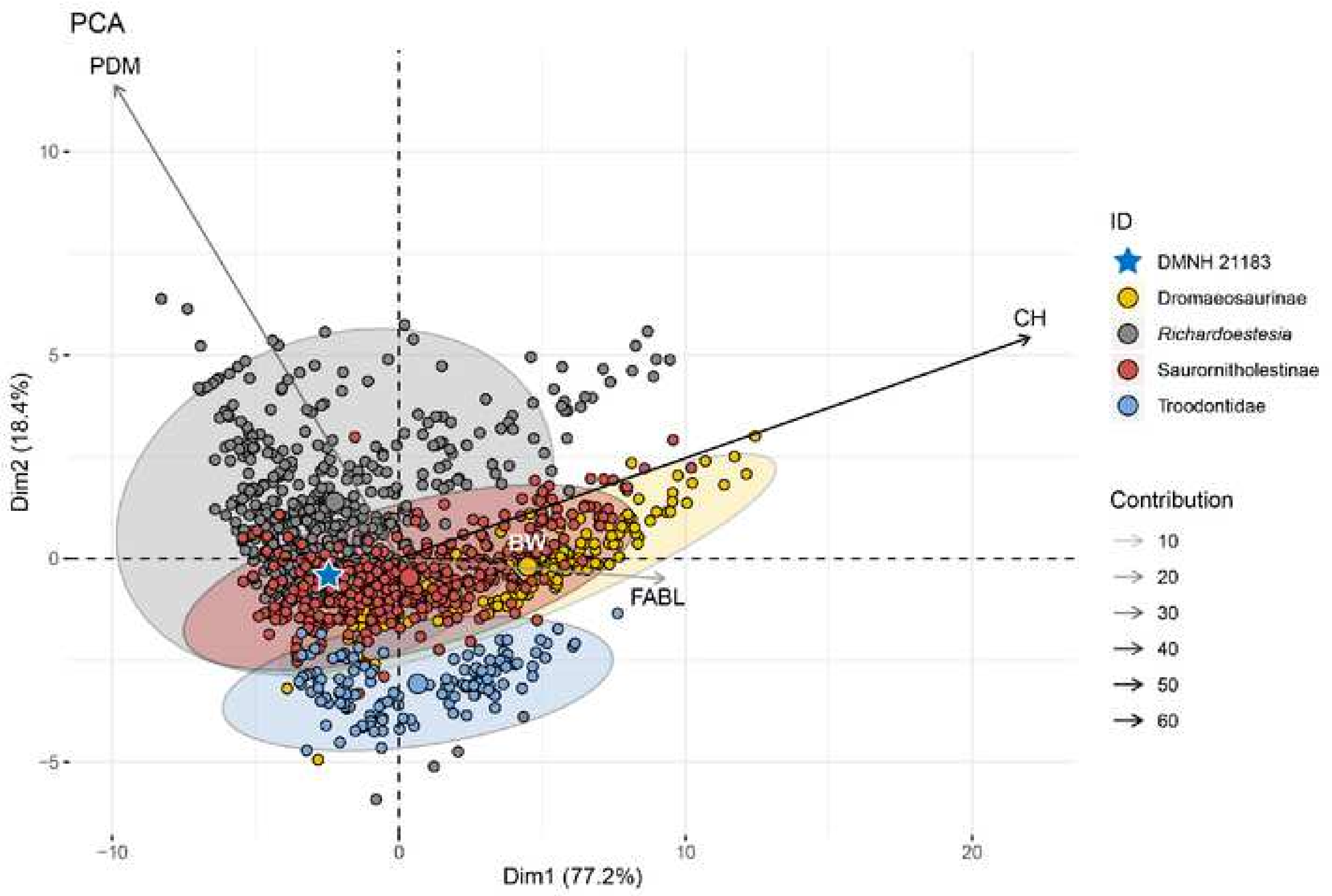

Figure 9

Click here to access/download;Figure;Figure 9.tif

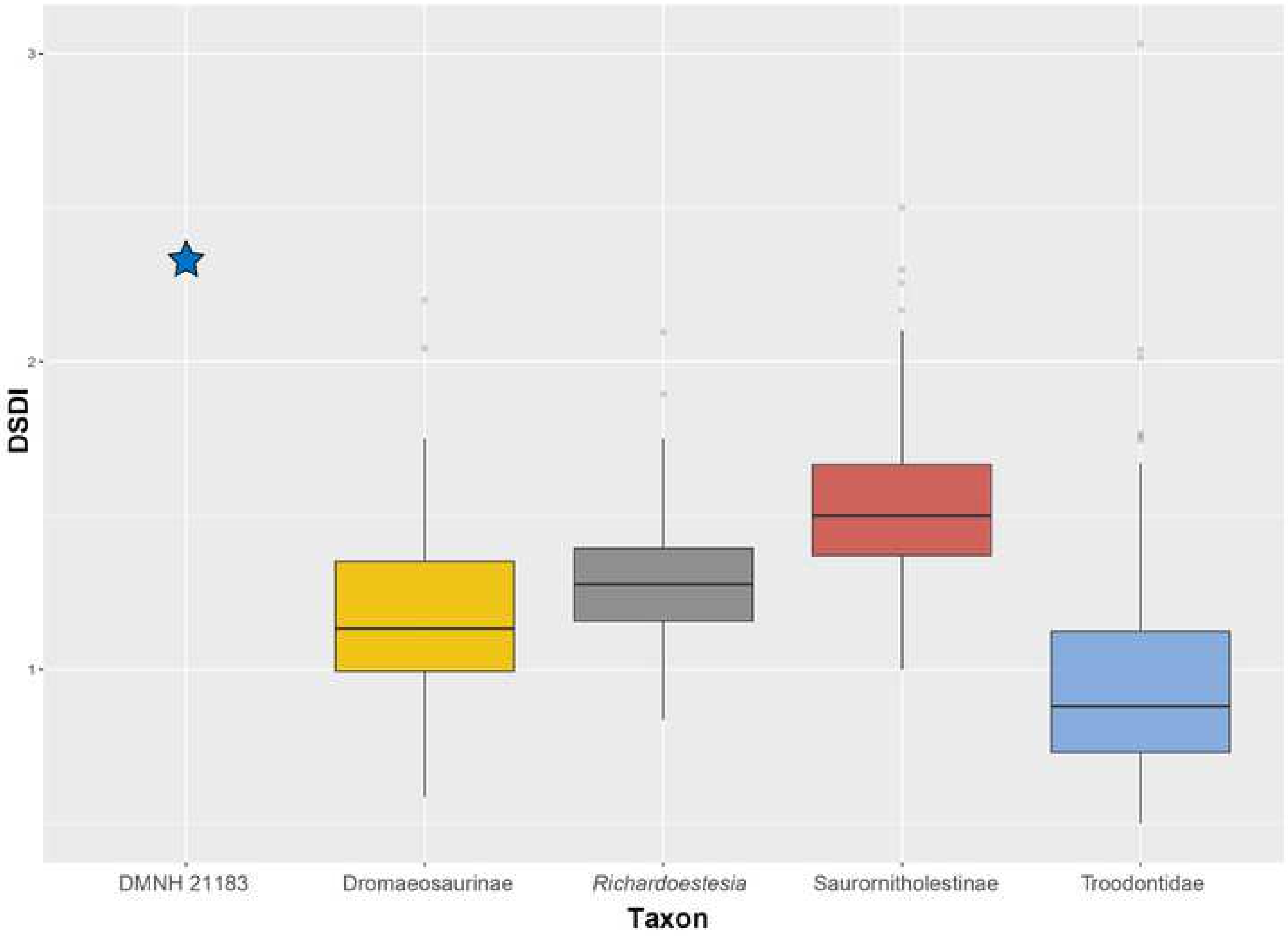

Figure 10

Click here to access/download;Figure;Figure 10.tif

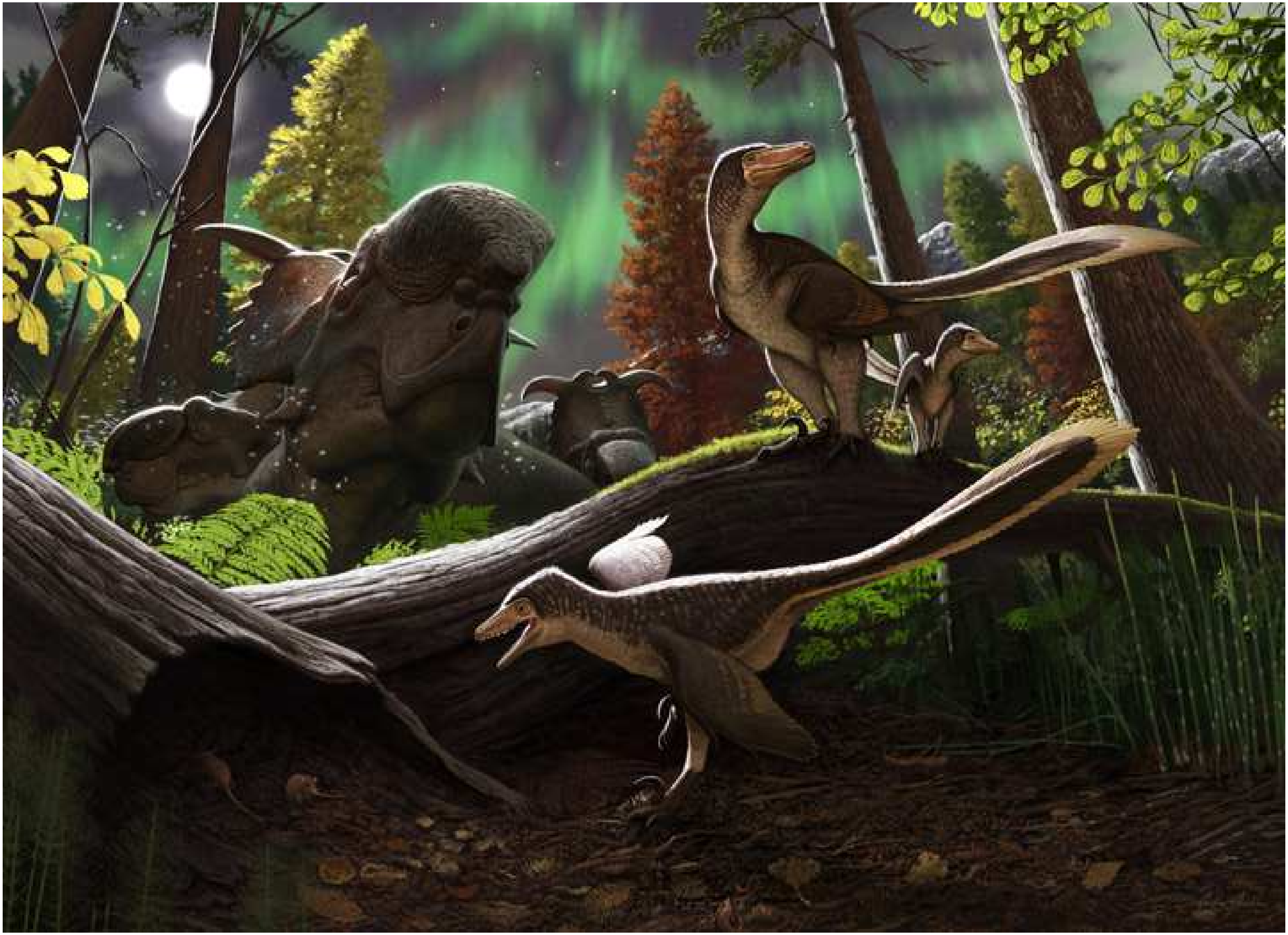

Dataset S1

Click here to access/download
Supporting Information
Dataset S1.rtf

Dataset S2

Click here to access/download
Supporting Information
Dataset S2.xlsx

Dataset S3

Click here to access/download
Supporting Information
Dataset S3.xlsx

Figure S2

Click here to access/download
Supporting Information
Figure S2.tif

Figure S3

Click here to access/download
Supporting Information
Figure S3.tif

Figure S4

Click here to access/download
Supporting Information
Figure S4.tif

Figure S5

Click here to access/download
Supporting Information
Figure S5.tif

Figure S6

Click here to access/download
Supporting Information
Figure S6.tif

Table S1

Click here to access/download
Supporting Information
Table S1.xlsx

The first juvenile dromaeosaurid (Dinosauria: Theropoda) from Arctic Alaska

Alfio Alessandro Chiarenza[1*], Anthony R. Fiorillo[1], Ronald S. Tykoski[1], Paul J. McCarthy[2], Peter P. Flaig[3], Dori L. Contreras[1]

Formatted: Superscript

[1] Perot Museum of Nature and Science, Dallas, TX, USA, [2] Department of Geosciences, University of Alaska, Fairbanks, AK, USA [3] Jackson School of Geosciences, Bureau of Economic Geology, University of Texas at Austin, Austin, TX, USA

Formatted: Superscript

Formatted: Superscript

Formatted: Superscript

* a.chiarenza15@gmail.com

Abstract

Compared to the more complete osteological record of herbivorous dinosaurs from the Late Cretaceous Prince Creek Formation of northern Alaska, there are theropod record has provided relatively fewer remains of theropods. While Tmost of the theropod record from this unit is mostly compriseds of isolated teeth, and the only non-dental remains known can be attributed to the troodontid cf. *Troodon* and the tyrannosaurid *Nanuqsaurus*. Thus far, the presence of members of the Dromaeosauridae hasve thus far been limited to isolated teeth. Here we describe a symphyseal portion of a small dentary with two ziphodont teeth. of diminutive

~~small size.~~ Based on t~~ee~~ooth shape, denticle morphology,~~, ~~ and the position of the Meckelian groove,~~, and a morphological comparison based on other theropod taxa~~ we attribute this partial dentary to a saurornitholestine dromaeosaurid. The fibrous bone surface, small size, and higher number of mesial denticles compared to distal ones point to a juvenile ~~status ~~growth stage for this individual. Multivariate ~~exploration ~~comparison of theropod teeth morphospace by means of principal component analysis reveals an overlap between ~~the Pediomys Poin~~thist dentary and Saurornitholestinae dromaeosaurid morphospace, a result supported by phylogenetic analyses. This is the first confirmed non-dental fossil specimen from a member of Dromaeosauridae in the Arctic, expanding on the role of Beringia as a dispersal route for this clade between Asia and North America. Furthermore, the juvenile nature of this individual adds to a growing body of data that suggests ~~that~~ Cretaceous Arctic dinosaurs of Alaska did not undergo long-distance migration, but rather they were year~~-~~-round residents of these paleopolar latitudes.

Competing interests: the authors have declared that no competing interests exist.

Introduction

Dromaeosauridae [1–3] (Table S1) is a group of predatory theropod dinosaurs evolutionarily close to the origin of birds [4, 5]. This clade likely originated in the Middle Jurassic [6, 7], with the first definitive dromaeosaurids recovered from Cretaceous deposits [8]. and Bby the Late Cretaceous they reached a virtually cosmopolitan distribution [89], so far remaining , so far, unknown only in Antarctica. Given the small to medium size of most dromaeosaurids, and their fragile, highly pneumatic skeletons that areis subject to higher greater incompleteness bias than many other dinosaur taxa [910, 1011], complete remains from of this group are generally rare, and confined to exceptionally productive fossil localities [e.g. 1112, 1213]. North American taxa belong to at least 4 recognized major subclades (Dromaeosaurinae, Microraptorinae, Saurornitholestinae, and Velociraptorinae; Table S1 [14]) with probable Asian origins based on phylogenetic inference and local abundance of taxa referred to these clades [8]. Since the earliest discoveries of dinosaur remains oin the Alaskan North Slope (1315–1517), the number of studies describing dinosaurs from the Prince Creek Formation and their role in clarifying palaeobiogeographic and palaeoecological characteristics aspects of the Cretaceous Arctic has greatly increased [(e.g. 1618–2325).

Formatted: Not Highlight

Dinosaur teeth often preserve more easily and are more frequently recovered than bones [26], and the discovery of isolated teeth referable to Dromaeosauridae

Formatted: Not Highlight

in many Late Cretaceous microsites has often provided important biogeographic data confirming the presence of the group in areas for which purely osteological remains are unknown [~~8~~9, ~~24~~27]. For example, Fiorillo and Gangloff [~~25~~28] reported on isolated dromaeosaurid teeth from the Prince Creek Formation of Alaska, tentatively referring them to *Dromaeosaurus* and *Saurornitholestes*. Given the intermediate pal~~a~~eogeographic position of Alaska (as part of the ancient Beringian landmass), and its role as a land bridge between Asia and North America, additional dromaeosaurid remains with better resolved taxonomic~~al~~ identification have the potential to increase our understanding of the origin and dispersal of these clades through Asiamerica. Here we describe the first non-dental, osteological material ~~belonging to~~of a saurornitholestine dromaeosaurid from Alaska, representing a ~~purported~~ unique morphotype. This ~~finding of the first osteological remains~~find supports the presence of ~~a previously unrecorded~~this clade ~~on the basis of osteological remains from~~in the Upper Cretaceous (lower Maastrichtian) Prince Creek Formation on the North Slope of Alaska (70° N, Fig 1).

Materials and Methods

DMNH 21183 is a symphyseal portion of a theropod dentary with a semi-erupted tooth and a replacement tooth preserved. The specimen was studied using a

Nikon SMZ motorized stereomicroscope (Nikon Instruments, Inc., Melville, NY, USA), equipped with epi-fluorescence with an X-Cite XYLIS light source (Excelitas Technologies, Waltham, MA, USA) ~~with~~ and GFP filter. Imaging of the specimen, including focal stacking and 3-D reconstructions, was completed using an attached Nikon Ri2 color CMOS camera with Nikon's NIS-Elements Acquisition and Analysis Software. Additional microscope observations and imaging were carried out using a Keyence Digital VHX-7000 series microscope (Keyence Corporation of America, Itasca, IL, USA). Dental nomenclature and terminology is based on Hendrickx et al. [29]~~26]~~. Terminology regarding ontogenetic characters is mostly, but not exclusively based on Sampson et al. [~~2~~30~~7~~], Carr [~~2~~31~~8~~], and Hone et al. [~~29~~32]. Anatomical description is based on morphological observation by three of the authors (AAC, AF, RT). Comparisons ~~have been~~were made based on first hand observations ~~on~~ of relevant material by ~~three of us (~~AAC, AF, and RT~~)~~, as well as literature comparisons. Stratigraphic and sedimentological observations were carried out by three of the authors ~~of the manuscript~~ (AF~~T~~, PF, PM) between 2005~~1988~~ and 2014 (Fig 2). Cladistic analyses and character evaluation were conducted by ~~three~~ ~~additional~~ authors AAC, DC, RT, and AF.

To assess the systematic position of DMNH 21183 within Theropoda, we performed three different phylogenetic analyses. ~~For t~~The first analysis, based on

~~both~~ osteological and dental characters, ~~we~~ used the dataset from Lee et al. [33]~~.~~, ~~This phylogenetic dataset~~which included 120 operational taxonomic units (OTUs) and 1529 characters. See Lee et al. [33] and Cau et al. [34] for further information on character choice and ~~logical basis in character treatement~~coding. Our updated matrix ~~include 1529 characters and~~differed only in the addition of ~~121 taxa (comprising of~~ DMNH 21183 as an OTU~~)~~. The second and third analyses ~~F~~follow~~ing~~ed the protocols outlined by Hendrickx et al. [35] to identify isolated theropod teeth.~~, we scored~~ DMNH 21183 was scored in ~~two additional phylogenetic datasets:~~one matrix based on dentition characters, and another ~~one~~ on tooth-crown-only characters. Details on character choice and rationale can be found in Hendrickx et al. [35], where original datasets are also reported as supplementary material. The dentition-based matrix ~~comprises~~consists of 107 taxa and 146 characters. The tooth-crow-based matrix includes 102 taxa and 91 characters. These dental datasets were analys~~z~~ed by additionally forcing a topological constraint ~~[Hendrickx et al. 2020]. F~~following Hendrickx et al. [35], ~~the constrained tree~~ reflect~~s~~ing a ~~standard~~ previously recovered -tree topology for Theropoda (e.g. Rauhut and Carrano [36] and Brusatte et al. [37]), with ~~.~~DMNH 21183 as a floating OTU. Character scorings for DMNH 21183 in all three phylogenetic datasets are reported in Supplementary Information Document (S1 Dataset). Characters were all equally-weighted and treated as unordered or ordered

following the source literature. ~~For all t~~The phylogenetic analyses were ~~used~~run using the software TNT [38]. For each dataset, ~~As reported in Following Hendrickx et al. [35], w~~w~~w~~e performed ~~100~~ a “New Technology” search that included a combination of random and constraint sectorial searches, ratchet, tree-drifting, and tree-fusing,~~replicates using a tree-search protocol including: Wagner trees, TBR branch swapping, sectorial searches, Ratchet (perturbation phase stopped after 20 substitutions) and Tree Fusing (5 rounds~~ with ten search replications as the starting point for each hit and searches carried out ~~),~~ until 100 hits of the same minimum tree length were achieved (~~The~~ TNT command used is “xmult= hits 100 replic 10 css rss ratchet 5 drift 5 fuse 5”)~~.~~. The most parsimonious trees (MPTs) obtained were subjected to two~~a final~~ rounds of TBR branch swapping (command “bbreak =TBR"). Strict consensus trees were generated from the resulting set of MPTs ~~The TNT command used is “xmult= hits 100 replic 10 css rss ratchet 5 drift 5 fuse 5”. In the tooth-crwoown and dental-only analyses from Hendrickx et al. [35], DMNH 21183 was selected as a floating OTU.~~ Nodal support was calculated for the consensus by running a standard bootstrap analysis with~~on~~ 1000 replicates.

To test the qualitative observations reported in the anatomical description section, we performed multivariate analyses of~~n~~ theropod teeooth measurements including those retrieved by the erupted tooth in DMNH 21183 (S2 Dataset).

WhileAlthough this tooth is not fully erupted, the breakages on the medial side of the dentary around the area of the relative alveolousalveolus and observations under microscopy could allowed tomeasurement of the crown height of the tooth. Quantitative mMeasurements on teeooth morphometrics were are based onfrom a modified dataset fromGerke and Wings [39] and a modified dataset from Larson and Currie [3040] (S2 Dataset).- The dataset from Gerke and Wings [39] includes measurement data for 335 theropod teeth for which entries were modified to reflect a higher systematic rank or clade systematics compared to the original genus-level classification (e.g. Abelisauridae, Dromaeosauridae, Tyrannosauridae). Measurements for this dataset include crown basal length (CBL), crown height (CH), crown basal width (CBW), and the ratio between mesial (anterior) and distal (posterior) denticles (DSDI). This dataset has been tested used previously in for other similar studies (e.g. [35]). This

The dataset from Larson and Currie [40] includes measurement data for over 1200 small theropod teeth, mainly from the latest Cretaceous of the Western Interior Basin of North America, and have has also been used for similar studies in the past [e.g. 3141, 3242]. Principal measurements included fore-aft basal length (FABL), crown height (CH), basal width (BW), and anterior (ADM) and posterior denticles per millimeter (PDM). The This dataset was modified by removing all entries with at least one variable missing or uncodifiableunable to be evaluated,

Formatted: Not Highlight

~~like~~ such as the lack of a measurement caused by the absence of the related structure (e.g. ADM in teeth without mesial denticles or carina). In this dataset, the taxonomy of the identified teeth was referred to the “family-ranked” clade with the exception of *Richardoestesia*. For example, *Saurornitholestes langstoni* ~~is identified as Saurornitholestinae.~~and *Atrociraptor marshalli* ~~is~~ are both referred to Saurornitholestinae, ~~(~~following [14]~~)~~. The Milk River cf. *Zapsalis* ~~*abradens*~~ is referred to *Saurornitholestes* ~~Dromaeosaurinae~~following Currie and Evans [43]. The Aquilan cf. *Richardoestesia gilmorei*, the Oldman cf. *Richardoestesia gilmorei*, the Horseshoe Canyon cf. *Richardoestesia*, the Lancian cf. *Richardoestesia*, the Aquilan cf. *Richardoestesia isosceles*, and *Richardoestesia isosceles* are all referred to *Richardoestesia*. The ~~examined~~ subclades of theropods included in this dataset, apart from the specimen studied first hand (DMNH 21183) are: Dromaeosaurinae, *Richardoestesia*, Saurornitholestinae and Troodontidae.

We performed ~~a~~two principal components analyse~~i~~s (PCA), a multivariate technique that takes a number of measurements and~~,~~ ~~distilling~~ converting~~s~~ them into a smaller set of values ~~representing~~ that represent~~s~~ the variability of the sample ~~to be~~ plotted in a multivariate space. Following the example of ~~other~~ similar studies (e.g. [9, 35, 39]), the same measurements were also used for a linear discriminant function analyse~~i~~s (DFA) on both the datasets. This method provides a value to assess the degree of confidence on the classification of the clusters in the

Formatted: Font: Italic

Formatted: Not Highlight

morphospace, where 0.5 is no better than random in model accuracy while 1 represents perfect accuracy (100% accuracy [44]). ~~This~~ Both these methods allow~~s~~ the dataset of teeth to be plotted in a morphospace, and to quantitatively ~~estimate~~ compare the degree of overlap and the relative position of DMNH 21183 ~~within~~ with 1) the main clades of Theropoda from the Gerke and Wings [39] dataset and 2) the main dein~~i~~onychosaurian ~~sublcades~~subclades ~~examined clades~~ (e.g., Dromaeosaurinae, Troodontidae) from the Larson and Currie [40] dataset. Multivariate analyses were performed in the software "R" with the "MASS" package [45]. PCA outcomes are reported as results in the relevant section while DFA plots are provided in Supplementary Information (S2-S3 Figs). ~~Check~~See Table S1 for systematic definitions of clades and lineages mentioned here and used throughout the text.

Institutional Abbreviations

AMNH–American Museum of Natural History, New York City, USA; ~~CAGS–Chinese Academy of Geological Sciences, Beijing, China;~~ DMNH–Perot Museum of Nature and Science, Dallas, Texas, USA; IVPP–Institute for Vertebrate Paleontology and Paleoanthropology, Beijing, China; MPC-D–Institute of Paleontology and Geology, Mongolian Academy of Sciences (formerly known as IGM), Ulaanbaatar, Mongolia; ~~YFGP–Yizhou Fossil and Geology Park, Liaoning,~~

China; YPM–Yale Peabody Museum of Natural History, Yale, Connecticut, USA; NHMUK PV–Natural History Museum, London, UK; TMP–Royal Tyrrell Museum of Palaeontology, Drumheller, Alberta, Canada

Results

Systematic Paleontology

Dinosauria [33]

Formatted: Indent: First line: 0"

Theropoda [34]

Dromaeosauridae [1]

Saurornitholestinae [35] indet.

Check Table S1 for systematic definition of clades mentioned here and used throughout the text.

Referred specimen

Formatted: None

DMNH 21183. The anterior portion of a right dentary, preserving two teeth and four alveoli (Fig 3).

Formatted: None, Indent: First line: 0"

Locality

The specimen was collected from exposures of the Prince Creek Formation, at the Pediomys Point locality (Figs 1 and 2). The locality is located along the

Colville River, 8 km upstream from the Liscomb bonebed, North Slope Borough, Alaska, USA (Fig 1). Bulk sediment was collected at the site over multiple field seasons between ~~1988~~ 2005-2007, 2012, and 2014, with screenwashing and sorting of the material conducted at the Perot Museum of Nature and Science (DMNH) in Dallas, Texas, USA.

Geological ~~setting~~ Setting and Depositional Environments

The Prince Creek Formation (PCF) was deposited in the Colville Basin of northern Alaska and provides us with the largest collection of polar dinosaur bones in the world [~~23~~25]. The PCF was originally sub-divided into two subunits or tongues: an older Tuluvak Tongue~~,~~ and a younger Kogosukruk Tongue [~~36~~46]. However, Mull et al. [~~37~~47] revised this nomenclature based on regional stratigraphic correlations and the Prince Creek Formation was redefined to include only the former Kogosukruk Tongue along with some younger, Paleocene strata. The total thickness of the PCF along the Colville River is approximately 450 m [~~38~~48, ~~39~~49]. Biostratigraphic [~~40~~50–~~49~~59], and isotopic analyses [~~50~~60] indicate that the age of the Prince Creek Formation ranges from Campanian to Paleocene. A~~However, a~~ll deposits containing evidence of dinosaurs are Early Maastrichtian in age~~, or~~ and approximately ~~69~~68.5-70 million years old [~~47~~57, ~~49~~59, ~~51~~61–~~53~~63].

The PCF is an alluvial succession composed of conglomerate, sandstone, siltstone, mudstone, carbonaceous shale, coal, and bentonite [4959, 5161, 5464]. The mostly fine-grained sediments record deposition in low energy, suspended load channels and on organic-rich floodplains on a low-gradient coastal plain. Thicker, multi-story meandering trunk channels contain the largest grain sizes and record the highest flow velocities in the area. Smaller meandering and fixed or anastomosed distributary channels formed crevasse splay-complexes adjacent to trunk channels. Abundant organic-rich facies were deposited in low-lying areas between the large channels and splay-complexes. Floodplains contained levees, splays, lakes, ponds, swamps, and soil-forming environments. Volcanic ashfall was common and smectite-rich bentonites were commonly preserved in wet floodplain environments [5565]. Trampling of sediments by dinosaurs was common [5363]. Weakly-developed paleosols similar to modern *Entisols*, *Inceptisols*, andic soils, and potential acid sulfate soils formed on levees, point bars, crevasse splays, and along the margins of floodplain lakes, ponds, and swamps that also supported lowland trees, shrubs, herbs, ferns, moss, and algae [5161, 5565]. Macroscopic and micromorphological features, and illite-smectite mixed-layer clay minerals in paleosols indicate predominantly waterlogged, reducing conditions interrupted by oxidizing conditions and periodic drying out of some soils [5161, 5262, 5464, 5565]. Soils experienced repeated sediment influx from overbank flooding of

nearby distributary channels, and periodic deposition of hyperconcentrated flows [~~52~~62]. Sediments deposited in the most distal areas of the coastal plain contain evidence of marine influence that includes inclined heterolithic stratification in channel point bars and pyrite, jarosite mottles, jarosite halos surrounding root-traces, and brackish-water fauna in floodplain facies [~~54~~64, ~~56~~66].

The 750 m-long outcrop belt at Pediomys Point is located near the upriver end of~~along~~ a slough off the main course of the Colville River (Fig 1). Cretaceous environments preserved at Pediomys Point include a meandering distributary channel that transitions laterally into a silt and mud-filled abandoned channel along with floodplain environments that include crevasse splays, levees, small lakes and swamps, floodplain paleosols, and ashfall deposits ([~~57~~67], Fig 2). Trampled sediments found above a bentonite (Fig 2~~A~~) are similar to those described from strata 4.25 kilometers downstream along the Colville River that are interpreted as adult and juvenile hadrosaur tracks emplaced along a swamp margin [~~53~~63]. Rare brackish-water clams (most likely *Nucula* aff. *N. percrassa* Conrad; see [~~56~~66]) and gastropods found near the top of the stratigraphic succession at Pediomys Point suggest an estuarine or lagoonal environment for those deposits [~~56~~66]. Interfingering of continental-terrestrial and shallow marine deposits, including those of floodbasins, interdistributary bays and estuaries were identified in older deposits of the PCF along the Colville River [~~58~~68] and in younger deposits above

the Liscomb Bonebed near Ocean Point [~~59~~69]. This suggests that the environments at Pediomys Point were transitional between the subaerial coastal plain or delta plain, and shallow marine habitats (Fig 2~~B~~).

Systematic Paleontology

Dinosauria [70]

Theropoda [71]

Dromaeosauridae [1]

Eudromaeosauria [14]

Saurornitholestinae [14] indet.

Referred specimen

DMNH 21183. The anterior portion of a right dentary, preserving two teeth and four alveoli (Fig 3).

Description

DMNH 21183 (Fig 3) (Table 1) is an anterior portion of a right dentary with an unerupted ~~anterior~~mesial-most tooth (d2; Fig 3A–3C) and a ~~posteriorly~~ distally placed partially erupted tooth (d3; Fig 3). The anterior surface of the dentary is damaged, obscuring details of the symphyseal region. Given the position of the

~~most~~ erupted tooth (d3; Fig 3) above the Meckelian foramina (on the medial surface of the dentary, Fig 3B), and above an the anteroventral process of the dentary (ave; Figs 3A and 3B), we identify this as the 3rd dentary tooth in the dentary [e.g. ~~60~~43, ~~61~~72], with the more ~~anteriorly~~ mesially positioned, ~~less~~ ~~erupted~~un~~d~~erupted tooth (d2) being identified as the 2nd. Alveoli 2–4 are preserved (Fig 3A–C), although the margins of the latter (fourth) are obscured by erosion~~damaged edges~~. Both the medial and lateral surfaces of the dentary present a parallel, anteroposteriorly~~-~~-oriented, fibrous bone texture. The anterior margin of the 3rd alveolus (a3; Fig 3A–C) ~~shows~~ has a raised anterior rim that extends dorsally up to approximately mid-height of the crown ~~of the relative tooth~~ (a3). There is a well-preserved subtriangular interdental plate lingual to the septum ~~dividing~~ separating the 2nd and 3rd alveoli (a2–a3), and a bigger, semicircular one between the 3rd and 4th alveoli (a3–a4). The paradental space is relatively ~~shallow~~dorsoventrally short. Laterally, the dorsal rim of the dentary rises into a triangular ridge, which creates a convex surface anterolaterally that sinks at its base into a circular fossa. The alveoli are elliptically shaped, being slightly anteroposteriorly longer than mediolaterally wide ~~slightly more anteroposteriorly elongated rather than mediolaterally so~~ (Fig 3C). The medial side of the dentary features a shallow Meckelian groove (Fig 3B), set slightly more ventrally than mid-height~~mid-way depth of the bone~~ of the bone. The medial side of the dentary

Formatted: Font: 14 pt, Not Italic

Formatted: Font: 14 pt, Not Italic

features an anteriorly located Meckelian foramen (Fig 3B), which may be paired with a second, more ventrally positioned foramen, but damage to the bone surface in this area makes the identification of this feature uncertain. There is an anteroventral expansion in of the dentary margin of the dentary, visible both medially and laterally (Figs 3A and 3B)~~, but~~ that is excavated by a sub-oval fossa on the lateral side (Fig 3A).

Both preserved teeth in DMNH 21183 are ziphodont. The mes~~i~~odistal axis of the 2nd tooth (d2) is more anteromedially oriented in relation to the lateral margin of the dentary. The 2nd tooth is unerupted, but ~~damaging~~ damage to the medial surface of the alveolar wall exposes the most apical half of the tooth crown. While the anterior alveolar margin covers the mesial surface of the 2nd tooth (d2), 13 denticles are visible along the distal carina~~, with 13 denticles~~ (Figs 4A and 4B). The apical-most half of portion of the 3rd tooth (d3) is erupted, with 12 denticles visible on the distal carina (Figs 3A, 3B and 3D).

The ~~preserved~~ teeth ~~show~~ have ~~coarser~~ larger distal denticles than the mesial ones. ~~Although the erupted portion of the crown in both teeth d2 and d3 is exposed only in the apical-most half, with 13 distal denticles (for tooth d2) and 12 denticles (for tooth d3), ~~T~~t~~he unerupted portion of the third tooth crown ~~of tooth 3~~ d3 can be ~~observed~~ seen through a fracture on the medial side of the dentary (Fig 3B). Taking into account the base of this crown, an ~~hypothetical~~ estimate of ~30 denticles per

serrated margin carina can be inferred accounting for the half of the carina being inset in the tooth socketfor the tooth.

Unfortunately, wear of the mesial carina destroyed some morphological details of the denticles. However, apart from some denticles, most of the interdenticular grooves between adjacent denticles are clear, and they are shallow incisions rather than deep sulci (Fig 3D, S1 Fig). In the 2nd tooth this comparison between mesial and distal carina is not possible because the mesial (anterior) margin of the tooth is hidden by sediment and the labial wall of the alveolus (Figs 4A and 4B). The shape of the apices of the distal denticles is are slightly apically hooked, with an orientation toward the apex of the crown and with an externally rounded margin rather than pointed with a sharp tip (Figs 4A–4C).

Parallel, anterocaudally oriented fibrous bone texture is present on the surfaces of both the medial and lateral sides of the dentary in DMNH 21183. External textural features in dinosaur surface bone have been shown to change in relation to ontogeny [29], in a process mirroring internal microscopic remodeling [62]. For example, the skulls of ceratopsian dinosaurs show surface textural changes during ontogeny, from lightly striated to deeply rugose textures [27; 63]. Cortical bone texture with fine grained, longitudinally striated pattern [28] is considered a size-independent criterion as an indication of relative immaturity in non-avian archosaurs [27, 28, 64]. Lightly striated cortical bone grains express

Formatted: Highlight

~~nascent ontogenetic characters in theropods [28], as it is particularly clear from *Tyrannosaurus* [28], *Scipionyx* [65], and *Juravenator* [66, 67]. The same immature bone grain texture described in relatively immature individuals of these taxa is also present in DMNH 21183 (Figs 3A and 3B). This textural feature, in consideration of the diminutive size of the specimen (Table 1), points toward a juvenile status of DMNH 21183.~~

Table 1. Measurements of DMNH ~~21283~~21183

**Elements measured**	**Measurements (mm)**
Dentary anteroposterior length (dorsal view)	14.34
Dentary anteroposterior length (lateral view)	14.59
Maximum dentary mediolateral length	6.15
Maximum dentary dorsoventral depth	9.44
Alveolus II mesodistal length	2.95
Alveolus II labiolingual width	1.27
Alveolus III labiolingual width	2.76
Alveolus III mesodistal length	3.71
Tooth II mesodistal width	1.97
Tooth II labiolingual length	1.05

~~Morphological remarks~~

~~DMNH 21183 is an anterior portion of a right dentary. While the specimen is fragmentary, a list of anatomical characters can be traced and compared to identify its systematic position. Although very few non-dental theropod remains have been found in the Prince Creek formation of Alaska, a general comparison~~

Formatted: Left
Formatted: Left
Formatted: Left
Formatted: Left
Formatted: Left
Formatted: Left
Formatted: Left
Formatted: Left
Formatted: Left
Formatted: Left
Formatted: Left

with other theropod taxa, with a particular focus on those clades previously recognized in these units (Dromaeosauridae, Tyrannosauridae, Troodontidae), is attempted here. The combination of a ziphodont dentition, presence of interdental plates (e.g. [68]), a Meckelian groove and Meckelian foramina is characteristic of theropod dinosaurs (e.g. [69]). The orientation of the anterior-most tooth socket and its unerupted tooth (both anteromedially oriented), the presence of paired Meckelian foramina, and a ventral expansion laterally pierced by a fossa, point toward the identification of the piece as an anterior portion (almost symphyseal, as also shown by the ventral enlargement of the dentary in lateral view) of a theropod dentary. The shallow Meckelian groove present in the specimen is a derived maniraptoran feature [70], in contrast to deep groove seen in the basal tyrannoraptoran (e.g. Tyrannosauroidea) condition [71]. The Meckelian foramen (Fig 3A) is slit-like and not enlarged and rounded like in Tyrannosauridae (e.g. oral mandibular foramen in *Nanuqsaurus* [21] and foramen intramandibularis oralis in *Tyrannosaurus* [72]), a feature seen in Dromaeosauridae (e.g. *Dromaeosaurus*, [73]; e.g. *Acheroraptor*, [61]). The shallow paradental space, a Meckelian groove which is set in the lower half of the medial side of the dentary but not directly on the ventral margin, and the hooked distal denticles of the teeth are coelurosaurian combination features [74, 75]. The oval-lenticular shape of the alveoli from dorsal view (Fig 3C; S1 Fig) rather than box shaped (or more squared off) is more similar

to the condition in derived coelurosaurians (i.e. Maniraptora) rather than in Tyrannosauroidea [26]. The presence of distinct alveoli rather than a connected dorsal furrow excludes derived troodontids [26]. The triangular ridge on the lateral margin of the dentary (Figs. 3A and 4D) is shared with *Buitreraptor* [76]. The raised rim in front of the relatively better-preserved alveolar rim 4 (Fig 3A) resembles the condition of the anterior alveoli in *Saurornitholestes* (e.g. [60]). Interdental plates are notoriously rare in paravian theropods, and fusion or reduction of these structures has been largely considered synapomorphic for Dromaeosauridae or Deinonychosauria as a whole, except for some taxa like Microraptorinae [77], *Austroraptor* [78], *Acheroraptor* [61], and *Archaeopteryx* [79, 80]. The larger of the preserved interdental plates of DMNH 21183 is broadly triangular in shape (like *Acheroraptor*), with a somewhat arched apex and a broader base. The absence of medial crenulation or dorsoventral furrows in the interdental plate excludes it from assignment to Tyrannosauridae. The presence of interdental plates is unique, between North American Dromaeosauridae, apart from *Acheroraptor*.

The lenticular (more mediolaterally compressed than circular) cross section of the teeth in DMNH 21183 differs from the mesial and lateral dentitions of many troodontids, which have a subcircular cross-sectional outline at the crown base [26]. A non-diminutive first dentary tooth, subequal in size with more distally

~~positioned dentary teeth excludes Tyrannosauridae (e.g. [21]) and is typical of more derived Coelurosauria and paravians [26]. The teeth of DMNH 21183 lack concave surfaces adjacent to both carinae (Fig 4) as has been observed in some mesial teeth of the troodontids *Troodon* [81, 82], *Urbacodon* [83], an indeterminate troodontid taxon from Uzbekistan [83], and the troodontid *Xixiasaurus* [26, 84]. As in most paravians, the teeth preserved in DMNH 21183 exhibit short interdenticular sulci (opposite of the well-developed and deep sulci present in Abelisauridae, Tyrannosauridae, and Allosauroidea; Fanti and Therrien [8, 26], whereas short interdenticular sulci have been observed in the microraptorine specimen IVPP V13476 [85], the eudromaeosaurians *Deinonychus* (YPM 5232), *Saurornitholestes* [82] and *Dromaeosaurus* (AMNH 5356; [82, 86], as well as in some troodontids [87, 88] such as *Troodon* (NHMUK PV R.12568).~~

~~The distal denticles with a relative higher density compared to the much coarser denticles in Troodontidae is more similar to the condition in Dromaeosauridae [26]. The presence of coarser distal denticles is a more common feature found in saurornitholestine dromaeosaurids rather than Dromaeosaurinae [30]. An affinity to the sympatric cf. *Troodon* [89] can be excluded on the basis of serrated mesial teeth, which are un-serrated in this troodontid taxon [90]. An affinity with basal dromaeosaurid taxa like the halszkaraptorines and unenlagiines (e.g. *Halszkaraptor* [91], *Mahakala* [92], *Buitreraptor* [76] and *Austroraptor* [93])~~

can be excluded, as the dentition of these taxa are devoid of denticles [26]. Other deinonychosaurian taxa like the troodontids *Mei* [94], *Byronosaurus* [95], *Gobivenator* [96], *Urbacodon* [83], *Xixiasaurus* [84], IVPP V20378 and *Jinfengopteryx* [97], *Almas* [98, 99] and MPC-D 100-1128, the anchiornithines *Anchiornis* (IVPP V16055; [100]), *Eosinopteryx* (YFGP-T5197; [101]), *Aurornis* (YFGP-T5198; [102]) and the basal avialan *Archaeopteryx* (e.g., [103–105]) also have non-denticulate tooth crowns all along their jaws [26], excluding a potential affinity with DMNH 21183. It has to be cautioned though that independent reacquisition of denticulated teeth has been shown in some deinonychosaurian taxa like the anchiornithid *Caihong* and *Liaoningvenator*, in the derived Troodontinae (i.e. the node encompassing *Sinovenator* + more derived forms like *Troodon* and *Pectinodon*), and the clade Microraptorinae + Eudromaeosauria (sensu Turner et al. [92]).

The reduction in size of mesial denticles is similar to the condition in Saurornitholestinae rather than Dromaeosaurinae [60, 73, 106–109]. The shape of the denticles is slightly pointed toward the apex of the crown as in Dromaeosauridae and Troodontidae and not C- or U-shaped like in Tyrannosauridae [26]. The pair of anterior foramina nearby the symphysis recalls a similar condition found in Velociraptorinae like *Acheroraptor* [61].

The reduction or lack of mesial denticulation is shared with some Asian Velociraptorinae like *Tsaagan* [110]. A similar condition to DMNH 21183 where mesial teeth bear un-serrated/absent mesial carinae and denticulated distal carinae is present in many other theropod clades. This feature is observed in the dromaeosaurid *Tsaagan* [110], the troodontids *Linhevenator* [26, 111] and possibly *Saurornithoides* (AMNH 6516 [112]).

Although hooked denticles are primarily present in some derived troodontid taxa [94] as seen in *Pectinodon* [113], *Saurornithoides* [112], *Sinornithoides* [87], *Troodon* (e.g., 81, 82, 113, 114), in indeterminate troodontid taxa from France [108, 115] and Asia [83], and in some anchiornithines like *Caihong* [116], there are also dromaeosaurids that exhibit apically hooked denticles (e.g., [73, 82, 86, 113]). These include the eudromaeosaurians *Atrociraptor* (TMP 1995.166.01; [117]) and *Saurornitholestes* [82, 118]. A main and immediately noticeable difference between denticles in Dromaeosauridae and Troodontidae, is that the latter tend to bear particularly large, bulbous, and widely separated denticles, while Dromaeosauridae have more numerous, smaller and asymmetrically convex or parallelogram-shaped denticles (Fig 4A; [26]). Other deinonychosaurian taxa lack apically hooked denticles, with a morphology that is more symmetrical and apically convex, such as in Microraptorineae (e.g. *Graciliraptor* [IVPP V2339], *Microraptor* [CAGS-20-7-004] and *Sinornithosaurus* [IVPP V12811]), and in

derived eudromaeosaurs such as *Acheroraptor* [61], *Bambiraptor* [119], *Linheraptor* [120], *Tsaagan* [121], *Utahraptor* [122] and *Velociraptor* [11].

The number of 12-13 denticles in the exposed crowns of DMNH 21183 produces an estimated total number of denticles per carina higher than the ~15 present in many troodontids, particularly in taxa more derived than *Sinovenator* (e.g. *Daliansaurus* [123], *Linhevenator* [111], *Pectinodon* [30, 124], *Saurornithoides* [112], *Sinornithoides* [125], *Sinusonasus* [126], *Troodon* [81, 127, 128] and *Zanabazar* [112]). While some tooth crowns of *Saurornitholestes* appear to have less than 15 denticles on the carina [30, 82, 129] quantitative analyses by Larson and Currie [30] indicates that the large majority of *Saurornitholestes* teeth have much more than 15 denticles on the crown (for other remarks on morphological variation in the dental series of this taxon (see [60]). Within Deinonychosauria, taxa with a large number of denticles (≥6 per 1 mm) are *Richardoestesia*, Saurornitholestinae (including *Saurornitholestes*), *Sinovenator* and *Velociraptor* [26, 30, 130–133].

While the position of the mesial margin of the teeth preserved in DMNH 21183 and some degree of surface abrasion preclude detailed morphological observation, the finely serrated mesial carina, with denticles smaller than their distal serial homologues, can be seen particularly well in the 3rd tooth of the dentary (d3; Fig 3D). The presence of distal denticles larger than mesial ones was

long thought to characterize the dentition of Dromaeosauridae, and therefore used as a primary feature to identify Velociraptorinae versus Dromaeosaurinae teeth (e.g., [106–109]). Teeth with fine mesial serrations are usually characterized by a denticle size index (DSDI) higher than 1.2 while teeth that bear carinae with subequal denticle size have usually a DSDI≤0.9. These arbitrary values were proposed by Rauhut and Werner [106] and corresponds in the case of DSDI≈1.2 to approximately more than six mesial denticles for five distal serrations [26]. DMNH 21183 has a quite high DSDI (~2.3; Fig 5), which is well beyond the range of many deinonychosaurian taxa, and in the range of the most finely serrated Saurornitholestinae teeth. A DSDI >1.2 has been reported in the majority of eudromaeosaurians, such as the microraptorines *Changyuraptor* [136], *Graciliraptor* [126], *Sinornithosaurus* [137] and *Tianyuraptor* [138], the dromaeosaurines *Achillobator* [139], *Atrociraptor* [117], *Dakotaraptor* [140] and *Utahraptor* [122], the velociraptorines *Acheroraptor* [61], *Bambiraptor* [119], *Deinonychus* [141], *Linheraptor* [120], *Saurornitholestes* [82], and *Velociraptor* [142], and the European dromaeosaurids *Nuthetes* [143] and *Pyroraptor* [144].The surprisingly high DSDI may be a juvenile trait, since many juvenile theropods have been shown to exhibit particularly fine mesial serrations relative to the distal ones, and evidence from tyrannosaurids [71, 125, 134] has shown progressively decreasing DSDIs through ontogeny [135]. A DSDI >1.2 has been reported in the

majority of eudromaeosaurians, such as the microraptorines *Changyuraptor* [136], *Graciliraptor* [126], *Sinornithosaurus* [137] and *Tianyuraptor* [138], the dromaeosaurines *Achillobator* [139], *Atrociraptor* [117], *Dakotaraptor* [140] and *Utahraptor* [122], the velociraptorines *Acheroraptor* [61], *Bambiraptor* [119], *Deinonychus* [141], *Linheraptor* [120], *Saurornitholestes* [82], and *Velociraptor* [142], and the European dromaeosaurids *Nuthetes* [143] and *Pyroraptor* [144]. Some lateral teeth of the troodontid *Zanabazar* [145] and some isolated crowns assigned to *Troodon* have also a very high DSDI [81]. This is the opposite condition than that typically found in Dromaeosaurinae (DSDI < 0.9), like in the eponym taxon *Dromaeosaurus* (AMNH 5356).

Phylogenetic analysis results

Analysis of Inserting Adding DMNH 21183 in to the Lee et al. [33] data matrix with DMNH 21183 added (121 taxa × 1529 characters) we foundresulted in 384 MPTs with a strict consensusofthat are (Fig 5; S4 Fig) 6043 steps long, with a Consistency Index (CI) = 0.244 and a Retention Index (RI) = 0.587. In tThe strict consensus (Fig 5; S4 Fig), the topology obtained reproduced the one topology hypothesized by from Lee et al. [33], with a standard theropod phylogeny having *Herrerasaurus* as an outgroup, and in order of decreasing inclusiveness a monphyletic Coelophysoidea, Ceratosauria, Megalosauroidea, Allosauroidea, Tyrannosauroidea, Comspognathidae, Ornithomimosauria, Alvarezsauria,

Formatted: Left

Formatted: Font: Italic

~~Therizinosauria, Oviraptorosauria, Scansoriopterygidae, Deinonychosauria and Avialae.~~. DMNH 21183 is recovered in ~~a less inclusive clade of~~ Dromaeosauridae excluding Unenla~~n~~ginae, in a pol~~i~~ytomy with most ~~all~~ other ~~E~~eudromaeosaur~~i~~as (*Dromaeosaurus* + *Utahraptor*) and- ~~***Dromaeosaurus*** -~~ and ~~and~~ Microraptorinae clades.

The addition of DMNH 21183 ~~in the cladistic analysis conducted on~~to the dental-only character matrix ~~from~~ of Hendrickx et al. [35] ~~(107 taxa × 146 characters) with a forced topology (Fig 6A; S5 Fig)~~ produced 2 MPTs ~~generates a strict consensus between 2 MPTs~~ (1314 steps long, CI = 0.194, RI = 0.418)~~,~~. T~~-~~he Strict Consensus of the two trees ~~recovering a politomy between the main averostran clades but a well resolved Coelurosauria. This major avetheropodan clade comprises *Ornitholestes* and Comspognathidae at the base of a tree with a monophyletic Maniraptoriformes, including a politomy between *Haplocheirus*, Alvarezsauridae and the rest of Maniraptora (Therizinosauria + Pennaraptora).~~recovers Paraves ~~is here recovered~~as a trichotomy between Avialae, Troodontidae and Dromaeosauridae (Fig. 6A; S5 Fig). DMNH 21183 is ~~here (Fig 6A; S5 Fig)~~ recovered as the sister ~~clade~~ OTU of *Saurornitholestes*, in a partially resolved Eudromaeosauria (*sensu* Longrich and Currie [14]). This dromaeosaurid clade is represented by a pol~~i~~ytomy between *Atrociraptor*, a monophyletic clade with *Deinonychus*, *Tsaagan* and *Velociraptor*, and another monophyletic clade

Formatted: Not Highlight

Formatted: Font: Italic

Formatted: Font: Italic

Formatted: Font: Italic

Formatted: Font: Italic

Formatted: Not Highlight

Formatted: Font: Italic

Formatted: Font: Italic

Formatted: Font: Italic

Formatted: Font: Italic

Formatted: Font: Not Italic

with *Dromaeosaurus* and *Bambiraptor* as ~~sister group of~~successively closer taxa to the clade *Saurornitholestes* + DMNH 21183.

Lastly, the analysis that includes~~usion of~~ DMNH 21183 in the tooth-crown-based data matrix ~~(102 taxa × 91 characters) from~~ of Hendrickx et al. [35]~~,~~ recovers 5 MPTs (867 steps, CI = 0.183; RI = 0.439).~~ a relatively well-resolved~~The strict consensus tree (Fig 6B; S6 Fig) ~~from 5 MPTs, 867 steps long (CI = 0.183; RI = 0.439). While the resolution is quite poor in the basal part of the theropod tree (outside Averostra), the tree recovers mostly politomies between ceratosaurian and tetanuran clades. Topological resolution increases in Avetheropoda, with monophyletic Allosauroidea, Compsognathidae, Tyrannosauroidea and Maniraptoriformes. This latter clade is reproduced as~~produces a pol~~i~~ytomy between alvarezsaurs, therizinosaurs, oviraptorosaurs and the rest of Maniraptora (Avialae, Troodontidae and Dromaeosauridae). DMNH 21183 is recovered ~~in a more inclusive clade than~~within Eudromaeosauria (*sensu* Longrich and Currie [14]), in a pol~~i~~ytomy between *Atrociraptor*, *Bambiraptor*, *Deinonychus*, *Saurornitholestes*, and an Asian Velociraptorinae clade (*Velociraptor* + *Tsaagan*).

Formatted: Font: Italic
Formatted: Font: Italic
Formatted: Font: Italic
Formatted: Not Highlight
Formatted: Not Highlight
Formatted: Not Highlight
Formatted: Not Highlight
Formatted: Not Highlight
Formatted: Not Highlight
Formatted: Not Highlight
Formatted: Not Highlight
Formatted: Not Highlight
Formatted: Not Highlight
Formatted: Not Highlight
Formatted: Not Highlight
Formatted: Font: Italic
Formatted: Not Highlight
Formatted: Not Highlight
Formatted: Font: Italic
Formatted: Font: Italic
Formatted: Font: Italic
Formatted: Font: Italic
Formatted: Font: Italic
Formatted: Font: Italic

Multivariate analysis results

~~Entering~~ The dentition of ~~We entered~~ DMNH 21183 (the 3rd and more exposed-better preserved tooth; d3 in Fig 3D) was assessed in a morphometric dataset of theropod teeth [39], with ~~and did~~ a PCA analysis returning four4 axes with the following eigenvalues and percentages of total variance explained by each axis: Axis 1 (0.377, 96.583%), Axis 2 (0.006, 1.594%), Axis 3 (0.004, 1.13%), Axis 4 (0.003, 0.69%). Coefficients for the five measurements on each axis are given in Table 2. The majority of the variance is ~~comprised in~~captured in the first two axes of the principal components. The highest variable contribution is represented by CBW (37.79%), followed by CH (~33.70%), CBL (~28.12%), and lastly DSDI (0.38%). ~~A morphospace depicting t~~The position of DMNH 21183 in the first two axes of the theropod teeth morphospace is shown in Fig 7. DMNH 21183 overlaps the dromaeosaurid morphospace in the lower left quadrant of the plot. This convex hull partially overlaps with those of troodontids, noasaurids, and basal theropods ~~between~~among others. This cluster is ~~being~~ set on the opposite side from the centroids of allosauroids, ceratosaurids, and spinosaurids, which occupy most of the center and right area of the teeth morphospace. DMNH 21183 is in the opposite area ~~in~~of the morphospace than tyrannosaurids (Fig 7). A similar spatial arrangement is obtained with the DFA analysis (S2 Fig), in which model accuracy for classification of DMNH 21183 as a dromaeosaurid is of 0.65. To further

Formatted: Not Highlight
Formatted: Not Highlight
Formatted: Not Highlight
Formatted: Not Highlight
Formatted: Not Highlight
Formatted: Not Highlight
Formatted: Not Highlight
Formatted: Not Highlight
Formatted: Not Highlight
Formatted: Not Highlight
Formatted: Not Highlight
Formatted: Not Highlight
Formatted: Not Highlight
Formatted: Not Highlight
Formatted: Not Highlight
Formatted: Not Highlight
Formatted: Not Highlight

explore the position of DMNH 21183 between Dromaeosauridae and the other taxa that were morphometrically close in this first set of multivariate analyses, we used a dataset [~~30~~40] of deinonychosaurian teeth (see Materials and Methods) and ~~running~~ performed a PCA analysis. ~~returned which~~ The analysis returned four~~4~~ axes with the following eigenvalues and percentages of total variance explained by each axis: Axis 1 (15.005, 77.175%), Axis 2 (3.584, 18.433%), Axis 3 (0.559, 2.879%), Axis 4 (0.294, 1.513%). Coefficients for the five measurements on each axis are ~~provided~~ given in Table ~~2~~3.

Table 2. PCA coefficients for morphometric analysis with all theropods.

Measurement	PC1	PC2	PC3	PC4
**CBL**	0.530305	-0.207765	0.108537	0.814757
**CBW**	0.614766	0.7250023	-0.251823	-0.181711
**CH**	0.580509	-0.605216	0.065003	-0.540829
**DSDI**	-0.062033	-0.254791	0.959468	0.1032189

Coefficients of the PCA analysis run on the theropod teeth dataset published by Gerke and Wings [39] with the addition of DMNH 21183. ADM, anterior denticles per millimeter; BW, basal width; CBL, crown base length (in mm); CBW: crown base width; CDA: crown distal angle; CH: crown height (in mm); CH, crown height; DSDI: denticle size difference index, denticles in mesial carina divided by those in distal carina.

Formatted: Left
Formatted Table
Formatted: Font: (Default) Times New Roman
Formatted: Left
Formatted: Font: (Default) Times New Roman, 14 pt
Formatted: Font: (Default) Times New Roman, 14 pt
Formatted: Font: (Default) Times New Roman, 14 pt
Formatted: Font: (Default) Times New Roman
Formatted: Left
Formatted: Font: (Default) Times New Roman, 14 pt, Font color: Auto
Formatted: Font: (Default) Times New Roman, 14 pt, Font color: Auto
Formatted: Font: (Default) Times New Roman, 14 pt, Font color: Auto
Formatted: Font: (Default) Times New Roman, 14 pt, Font color: Auto
Formatted: Font: (Default) Times New Roman
Formatted: Font: (Default) Times New Roman, 14 pt, Font color: Auto
Formatted: Left
Formatted: Font: (Default) Times New Roman
Formatted: Left
Formatted: Font: (Default) Times New Roman, 14 pt, Font color: Auto
Formatted: Font: (Default) Times New Roman, 14 pt, Font color: Auto
Formatted: Left

Table 23. PCA coefficients.PCA coefficients for morphometric analysis with deinionychosaurian taxa.

Measurement	PC1	PC2	PC3	PC4
**FABL**	0.352856	-0.03872	0.568433	0.742211
**CH**	0.838152	0.423491	-0.31658	-0.13392
**BW**	0.176441	-0.00224	0.739112	-0.65006
**PDM**	-0.37665	0.90507	0.174281	0.092801

Formatted: Left
Formatted: Left
Formatted: Left
Formatted: Left
Formatted: Left
Formatted: Left

Coefficients of the PCA analysis run on the theropod deinonychosaurian teeth dataset published by Larson and Currie [3040] with the addition of DMNH 21183. ADM, anterior denticles per millimeter; BW, basal width; CH, crown height; FABL, fore-aft basal length; and PDM, posterior denticles per millimeter.

The majority of the variance is comprised in theexplained by the first two axes of the principal components. The highest variable contribution, (similarly to previous analyses on this dataset [3040–3242],]) is provided represented by CH (~60%) followed by PDM (~30%), FABL (~15%) and lastly ADM (<5%). A morphospace depicting tThe position of DMNH 21183 in the first two axes of the deinonychosaurian teeth morphospace is presented givenshown in Fig 68. DMNH 21183 overlaps the Saurornitholestinae morphospace, being set further away from the centroid clustering the other analyzed taxon taxon with hooked denticles, Troodontidae. It is well outside the convex hull comprising Dromaeosaurinae, which has mostly subequal, rectangular denticles, and is set within the

Saurornitholestinae morphospace in a position slightly toward the *Richardoestesia* morphotype. DFA results in a spatial arrangement~~s~~ of ~~datapoints~~data points similar to the one of the PCA (S3 Fig), and the analyses provides a model accuracy of 0.8 for classification of DMNH 21183 as a saurornitholestine deinonychosaur~~ of 0.8~~. ~~We therefore find t~~This outcome is comparable to those ~~of~~generated by the phylogenetic analyses, and~~-~~ we confidently ~~tentatively~~ refer DMNH 21183 to the Saurornitholestinae.~~,~~ ~~but do not a~~We do not at this time assign it to any currently recognized species within the clade.

One major caveat of these analyses regards the potential bias due to the different ontogenetic stages of the teeth included in the sample [~~31~~41], and most importantly in relation to the likely juvenile growth stage ~~status~~ of DMNH 21183. Because PCA analysis would simply group observations based on measurements, it is likely that teeth belonging to juvenile and adult individuals will cluster in separate areas of the morphospace (see Discussion for a more in depth comparison of denticle size). This issue has been shown ~~previously~~ in the literature (e.g. [~~146~~73, ~~147~~74]) to affect previous PCAs of tyrannosauroid teeth, so we use this morphometric line of evidence as a complementary tool to assess the systematic identification of DMNH 21183.

Discussion

Morphological comparisons and the phylogenetic position of DMNH 21183

~~DMNH 21183 is an anterior portion of a right dentary.~~ While DMNH 21183 ~~the specimen~~ is fragmentary, ~~a list of~~there are enough anatomical characters ~~can be compared~~ preserved in it to ~~identify~~indicate its likely systematic position. Although very few non-dental theropod remains have been found in the Prince Creek ~~f~~Formation of Alaska, a general comparison with other contemporaneous theropod taxa, with a particular focus on those clades previously recognized in the~~se~~ ~~units~~formation (Dromaeosauridae, Tyrannosauridae, Troodontidae)~~,~~ is attempted here. The combination of~~ a~~ ziphodont dentition, presence of interdental plates (e.g. [75]), a Meckelian groove, and Meckelian foramina ~~is~~are characteristic of theropod dinosaurs (e.g. [76]). The orientation of the anterior-most tooth socket and its unerupted tooth (both anteromedially oriented), the presence of paired Meckelian foramina, and a ventral expansion laterally pierced by a fossa, point toward the identification of the ~~piece~~specimen as an anterior portion (almost symphyseal, as also shown by the ventral enlargement of the dentary in lateral view) of a theropod dentary. The shallow Meckelian groove present in the specimen is a derived maniraptoran feature [77], in contrast to the deep groove seen in ~~the~~ basal tyrannoraptorans (e.g. Tyrannosauroidea) ~~condition~~ [78]. The Meckelian foramen (Fig 3B) is slit-like and not enlarged and rounded like in Tyrannosauridae (e.g. oral mandibular foramen in *Nanuqsaurus* [23] and foramen intramandibularis oralis in

Tyrannosaurus [79]), a feature seen in Dromaeosauridae (e.g. *Dromaeosaurus*, [80]; e.g. *Acheroraptor*, [72]). The pair of anterior foramina nearby the symphysis is similar to the condition found in velociraptorines like *Acheroraptor* [72]. The shallow paradental space, a Meckelian groove that is set in the lower half of the medial side of the dentary but not directly on the ventral margin, and the hooked distal denticles of the teeth are coelurosaurian features [81, 82]. The lenticular shape of the alveoli from dorsal view (Fig 3C; S1 Fig), rather than box shaped (or more squared off), is more similar to the condition in derived coelurosaurians (i.e. Maniraptora) rather than in Tyrannosauroidea [83]. The presence of distinct alveoli rather than a connected dorsal furrow excludes derived troodontids [83]. The triangular ridge on the lateral margin of the dentary (Figs. 3A and 4D) is shared with *Buitreraptor* [84], *Velociraptor* (AMNH 6515) and *Tsaagan* [85], although in all these cases the presence of this structure may be an artifact of the damaged lateral rim of the dentary. The raised rim in front of the well-preserved alveolar rim 4 (Fig 3A) resembles the condition of the anterior alveoli in *Saurornitholestes* [43].

Interdental plates are notoriously rare in paravian theropods, and fusion or reduction of these structures has been largely considered synapomorphic for Dromaeosauridae or Deinonychosauria as a whole, except for some taxa like Microraptorinae [86], *Austroraptor* [87], *Acheroraptor* [72], and *Archaeopteryx* [88, 89]. The larger of the preserved interdental plates of DMNH 21183 is ~~broadly~~

Formatted: Font: Italic
Formatted: Not Highlight
Formatted: Font: Italic, Not Highlight
Formatted: Not Highlight
Formatted: Not Highlight

triangular in shape (like *Acheroraptor*), with a somewhat arched apex and a broader base. The absence of medial crenulation or dorsoventral furrows in the interdental plate excludes it from assignment to Tyrannosauridae. The presence of interdental plates fused to the margine of the dentary is shared with *Atrociraptor* [90] and *Saurornitholestes* [43].

Formatted: Font: Italic

Formatted: Font: Italic

The lenticular (more mediolaterally compressed than circular) cross section of the teeth in DMNH 21183 differs from the mesial and lateral dentitions of many troodontids, which have a subcircular cross-sectional outline at the crown base [83]. The teeth of DMNH 21183 lack concave surfaces adjacent to both carinae (Fig 4), as has been observed in some mesial teeth of the troodontids *Troodon* [91, 92], *Urbacodon* [93], an indeterminate troodontid taxon from Uzbekistan [93], and the troodontid *Xixiasaurus* [83, 94] as reported by [83]. As in most paravians, the teeth preserved in DMNH 21183 exhibit short interdenticular sulci (opposite of the well-developed and deep sulci present in Abelisauridae, Tyrannosauridae, and Allosauroidea [9, 83], whereas short interdenticular sulci have been observed by Hendrickx et al. [83] in the microraptorine specimen IVPP V13476 [95], the eudromaeosaurians *Deinonychus* (YPM 5232), *Saurornitholestes* [43], and *Dromaeosaurus* (AMNH 5356; [92, 96], as well as in some troodontids [97, 98] such as *Troodon* (NHMUK PV R.12568).

The distal denticles have a relative higher density in contrast to the much larger denticles in Troodontidae, and are ~~is~~ more similar to the condition in Dromaeosauridae [83]. The presence of coarser distal denticles than mesial denticles is a more common feature found in saurornitholestine dromaeosaurids rather than Dromaeosaurinae [40]. An affinity to the sympatric cf. *Troodon* [26] can be excluded on the basis of serrated mesial teeth, which are ~~un-~~not serrated in the troodontid [99]. An affinity with basal dromaeosaurid taxa like the halszkaraptorines and unenlagiines (e.g. *Halszkaraptor* [100], *Mahakala* [101], *Buitreraptor* [84] and *Austroraptor* [102]) can be excluded, as the dentition of these taxa are devoid of denticles [83]. Other deinonychosaurian taxa like the troodontids *Mei* [103], *Byronosaurus* [104], *Gobivenator* [105], *Urbacodon* [93], *Xixiasaurus* [94], IVPP V20378 and *Jinfengopteryx* [106], *Almas* [107, 108] and MPC-D 100-1128, the anchiornithid *Anchiornis* [109], *Eosinopteryx* [110], *Aurornis* [111], and the basal avialan *Archaeopteryx* (e.g., [112–114]) also have non-denticulate tooth crowns all along their jaws [83], excluding a potential affinity with DMNH 21183. It has to be cautioned though that independent reacquisition of denticulated teeth has been shown in some deinonychosaurian taxa like the anchiornithids *Caihong* and *Liaoningvenator* [83].

The small size of mesial denticles in DMNH 21183~~s~~ is more similar to the condition in Saurornitholestinae rather than Dromaeosaurinae [43, 80, 115–118].

The shape of the denticles is slightly pointed toward the apex of the crown as in Dromaeosauridae and Troodontidae and not C- or U-shaped like in Tyrannosauridae [83].

The reduction or lack of mesial denticulation is shared with some Asian Velociraptorinae like *Tsaagan* [85]. A similar condition to DMNH 21183 where mesial teeth bear unserrated mesial carinae and denticulated distal carinae is present in many other theropod clades. This feature is also seen in the dromaeosaurid *Tsaagan* [85], the troodontids *Linhevenator* [83, 119] and possibly *Saurornithoides* (AMNH 6516 [120]).

Although hooked denticles are primarily present in some derived troodontid taxa [83, 103], there are also dromaeosaurids that have apically hooked denticles (e.g., [80, 92, 96, 121]). These include~~,~~ ~~like~~ the eudromaeosaurians *Atrociraptor* and *Saurornitholestes* [83, 92, 122]. ~~A main and~~An immediately noticeable difference between denticles in Dromaeosauridae and Troodontidae~~,~~ is that the latter tend to bear particularly large, bulbous, and widely separated denticles, while Dromaeosauridae have more numerous, smaller and asymmetrically convex or parallelogram-shaped denticles (Fig 4A; [83]). Other deinonychosaurian taxa lack apically hooked denticles, with a morphology that is more symmetrical and apically convex, such as in Microraptorineae and in some derived eudromaeosaurs

such as *Acheroraptor*, *Bambiraptor*, *Linheraptor*, *Tsaagan*, *Utahraptor* and *Velociraptor* ([83] and references therein).

The number of ~~12-13~~ denticles (12 to 13) in the partially exposed crowns of DMNH 21183 ~~produces an estimated~~indicates that the total number of denticles per carina is ~~higher~~greater than the ~15 present in many troodontids, particularly in taxa more derived than *Sinovenator* [26]. As Hendrickx [83] pointed out, while some tooth crowns of *Saurornitholestes* appear to have less than 15 denticles on the carina [40, 92, 123] quantitative analyses by Larson and Currie [40] indicates that the large majority of *Saurornitholestes* teeth have ~~much~~many more than 15 denticles on the crown (for other remarks on morphological variation in the dental series of this taxon see [43]). Within Deinonychosauria, taxa with a large number of denticles (≥6 per 1 mm) include *Richardoestesia*, Saurornitholestinae (including *Saurornitholestes*), *Sinovenator*, and *Velociraptor* [40, 83, 124–127].

While the position of the mesial margin of the teeth preserved in DMNH 21183 and the ~~and some~~ degree of surface abrasion preclude detailed morphological observation, the finely serrated mesial carina, with denticles smaller than their distal serial homologues, can be seen particularly well in the 3rd tooth (d3; Fig 3D). The presence of distal denticles larger than mesial ones was long thought to characterize the dentition of Dromaeosauridae, and this criterion was used to identify ~~V~~velociraptorin~~a~~e teeth (e.g., [83, 115–118]). Teeth with fine

mesial serrations are usually characterized by a denticle size index (DSDI: the ratio between number of mesial and distal denticles) higher than 1.2 while teeth that bear carinae with subequal denticle size have usually a DSDI close to 1. These arbitrary values were proposed by Rauhut and Werner [115] and corresponds in the case of DSDI≈1.2 to approximately more than six mesial denticles for five distal serrations [83]. DMNH 21183 has a quite high DSDI (~2.3; Fig 9), which is well beyond the range of many deinonychosaurian taxa, and in the range of the most finely serrated ~~S~~saurornitholestin~~a~~e teeth (between 1 and 2.5; Fig 9). A DSDI >1.2 has been reported in the majority of eudromaeosaurians ([83] and references therein). Some lateral teeth of the troodontid *Zanabazar* [128] and some isolated crowns assigned to *Troodon* have also a very high DSDI (outliers with DSDI around 2-3; Fig 9 [40, 83, 91]. This is the opposite condition than that typically found in Dromaeosaurinae (DSDI ~ 1), like in the eponymous taxon *Dromaeosaurus* [83]. The surprisingly high DSDI of DMNH 21183 may be a juvenile trait, since many juvenile theropods have been shown to exhibit particularly fine mesial serrations relative to the distal ones, and evidence from tyrannosaurids [78, 129, 130] ~~has shown~~shows progressively decreasing DSDIs through ontogeny [135].

External textural features in dinosaur surface bone have been shown to change in relation to ontogeny [32], in a process mirroring internal microscopic

remodeling [132]. For example, the skulls of ceratopsian dinosaurs show surface textural changes during ontogeny, from lightly striated to deeply rugose textures [30; 133]. Cortical bone texture with fine~~-~~grained, longitudinally striated pattern [31] is considered a size-independent criterion as an indication of relative immaturity in non-avian archosaurs [30, 31, 134]. Lightly striated cortical bone grain express nascent ontogenetic characters in theropods [31], as it is particularly clear from *Tyrannosaurus* [31], *Scipionyx* [135], and *Juravenator* [136, 137]. The same striated, ~~immature~~fibrous bone grain texture described in relatively immature individuals of these ~~these~~other theropod taxa is also present ~~along with~~in DMNH 21183 (Figs 3A and 3B). This textural feature, in ~~consideration~~combination with ~~of~~ the diminutive size of the specimen (Table 1), is evidence that DMNH 21183 is a juvenile.

After morphological, morphometric, and phylogenetic analyses, DMNH 21183 is here interpreted as specimen of ~~a~~ Saurornitholestinae. ~~Specifically, t~~The recurrent clustering of DMNH 21183 with Eurasian dromaeosaurids (a clade including Eudromaeosauria + Microraptorinae; Figs 5-6) and excluding Unenla~~n~~ginae (in [33]), the~~the~~ sister-~~group~~taxon relationship with *Saurornitholestes* (Fig 6A) in the analysis based on the dentition-only matrix from Hendrickx et al. [83], ~~and~~as well as in a eudromaeosaur clade more ~~in~~exclusive ~~clade of Eudromaeosauria excluding~~than that containing *Dromaeosaurus* in the

Formatted: Not Highlight

Formatted: Not Highlight

Formatted: Not Highlight

Formatted: Not Highlight

Formatted: Not Highlight

Formatted: Not Highlight

Formatted: Font: Italic

Formatted: Font: Italic

analysis based on the tooth-crown-only matrix from Hendrickx et al. [33], all strength~~t~~en the saurornitholestine ~~intepretation~~interpretation of the specimen based on morphological comparisons and teeth morphometrics.

Saurornitholestinae was erected by Longrich and Currie [14] as a subclade of Dromaeosauridae including *Saurornitholestes*, *Bambiraptor* and *Atrociraptor*. ~~While we are aware that not n~~Not all phylogenetic analyses focused on deinonychosaurian interrelationships recover this clade (e.g. [101, 138]), ~~while~~but others do [34, 37] or partially do so [139], and we recognize ~~retain the use of~~ this clade. ~~In this study, with~~ Saurornitholestinae ~~we mean~~is that ~~clade~~lineage of ~~taxa~~eudromaeosaurs closer to *Saurornitholestes* than to *Dromaeosaurus* and *Velociraptor*, and this more inclusive, stem-based concept of the name is ~~particularly~~ useful when discussing ~~of~~ isolated ~~teeth~~dental material which has often been assigned ~~in the relevant literature~~ to *Saurornitholestes* or cf. *Saurornitholestes* (e.g. [40, 96, 140]). There are several referrals to saurornitholestine dromaeosaurs in the latest Cretaceous of North America,~~. These~~ including~~e~~: the Milk River saurornitholestine ~~reported by~~[~~-~~96] from the latest Santonian–earliest Campanian; the Early Campanian Menefee Formation saurornitholestine [41~~(~~]; the Early Campanian Foremost ~~f~~Formation saurornitholestine [141]; and the Middle-Late Campanian Oldman ~~f~~Formation saurornitholestine [141] (Table S3).

Formatted: Font: Italic
Formatted: Font: Italic
Formatted: Font: Italic
Formatted: Font: Italic
Formatted: Font: Italic
Formatted: Font: Italic
Formatted: Font: Italic
Formatted: Font: Italic
Formatted: Font: Not Italic
Formatted: Font: Not Italic
Formatted: Not Highlight

More relevant for comparative purposes to taxa in the Prince Creek Formation, *Atrociraptor marshalli* ~~has been~~was recovered in ~~all~~ phylogenetic analyses ~~on~~of paravian interrelationships as a derived member of Dromaeosauridae and close to the node comprising *Saurornitholestes* (e.g. [37, 60]). In the dental-characters~~-~~only phylogenetic analysis, DMNH 21183 clusters separately ~~to~~from *Atrociraptor* (Fig ~~X~~6A), while the crown-only-characters and full-osteological resultt~~I~~s ~~consider~~recover both *Atrociraptor* and DMNH 21183 in a pol~~i~~ytomy within Eudromaeosauria (Figs~~.~~ ~~X~~5 and ~~X~~6B). T~~Although t~~here are similarities in the apically-hooked denticles ~~associating~~on the teeth of DMNH 21183, *Saurornitholestes*, and *Atrociraptor*, but ~~-~~the generally larger denticles of the latter, particularly in the mesial carina ~~of the latter~~ [90, 142], ~~make the comparison between~~sets it apart from ~~the mesial carinae of~~ *Saurornitholestes* and DMNH 21183.~~-~~ ~~more obvious (although~~However, the implications of ~~an~~ ontogenetic stage on relative~~effect in~~ denticle~~s~~ size ~~has to~~should also be considered in this case~~)~~. On the other hand, the interdental plates in DMNH 21183 more closely resemble ~~more closely~~ those of *Atrociraptor* (TMP 95.166.1 [90]) than those of *Saurornitholestes* (TMP 1988.121.0039 [60]) in having a narrower base. ~~This is not surprising since both~~ *Atrociraptor* and *Saurornitholestes* are recovered in a sister-group relationship ~~in~~by Currie and Evans [60], and the shared similarities between DMNH 21183 and these two taxa may prove predictive ~~similar affinities and~~

Formatted: Not Highlight

Formatted: Font: Italic

Formatted: Font: Not Italic

Formatted: Font: Not Italic

Formatted: Font: Italic

Formatted: Font: Italic

Formatted: Font: Italic

Formatted: Font: Italic

Formatted: Font: Italic

Formatted: Font: Not Italic

Formatted: Font: Not Italic

Formatted: Font: Italic

Formatted: Font: Italic

Formatted: Font: Italic

Formatted: Font: Italic

Formatted: Font: Italic

morphology should the Prince Creek Formation provide additional, more complete dromaeosaurid material comparable to DMNH 21183be found in the PCF.

Paleoecological and paleobiogeographical implications of a juvenile Arctic Saurornitholestinae

The discovery of dinosaur remains at high latitudes, and in particular the abundant dinosaur bone record from the PCF, challenged traditional reptilian models for dinosaurian physiologies and inspired debate centered on the potential for long-distance migrations by dinosaurs [1820, 148143–150145]. Given the breadth of migration patterns in extant animals (e.g., [146]Rappole, 2013), to focus our discussion it is relevant to point out that these migrations for Arctic dinosaurs were inferred to cover latitudinal distances rather than trans-Arctic migrations, which are not even observed with animals today [147]. ISuch increased subsequent interest showed, through a variety of methods such as biomechanic, isotopic analyses, and osteohistology, that these dinosaurs likely had the necessary adaptations for overwintering in the ancient Arctic [1820, 151148, 152149).] and need not have moved to more southerly latitudes. Thus far, these discussions have focused almost exclusively on herbivorous taxa, with one exception that was based

Formatted: Left, Indent: First line: 0"

Formatted: Left

Formatted: Highlight

on ~~a speculative~~the argument that if the dinosaurian prey did not migrate, then the predators were non-migratory as well [~~25~~28].

In their review of the adaptive benefits of migration for modern mammals, Avgar and others [~~155~~150] showed that in the terrestrial realm there is a decided preference for mammalian long-distance migrations to occur among large-bodied herbivores rather than carnivores. One of the suggested reasons for the rarity of migration by mammalian carnivores is that for purposes of energy consumption and mating, these animals need to establish, maintain, and defend territories. Such behavior would preclude the ability to migrate.

~~Using birds as analogue for inferring non-avian theropods' migratory strategies is problematic, as the lack of active flight in the latter preclude an energetically efficient way of covering large distances when constrained by terrestrial locomotion. Large, mostly land-dwelling predatory birds, like for example the shoe-billed stork (*Balaeniceps rex*) or the secretary bird (*Sagittarius serpentarius*), are usually non-migratory and their distribution is more strongly constrained by resource availability [154, 155]. A potentialhypothetical exception to this case wouldcould be made byif one considereding smaller the Arctic non-avian theropods to be flight-capable, as southern latitude migrants, which wcould then fly long distances to exploit the higher productivity of the extreme latitude habitats during the summer, for then migrateing to southern latitude for nesting in~~

Formatted: Highlight
Formatted: Left
Formatted: Highlight
Formatted: Highlight
Formatted: Highlight
Formatted: Highlight
Formatted: Highlight
Formatted: Highlight
Formatted: Highlight
Formatted: Highlight
Formatted: Highlight
Formatted: Highlight
Formatted: Highlight
Formatted: Highlight

the cold season [Herman et al. 2016]. However, there is no analog for such long-distance seasonal migrations in any modern, predatory, non-volant, vertebrate. Also, even though dromaeosaurids Dromaeosaurids were evolutionarily close to avians, and there is some discussion of the flight capabilities of some taxa within this group [151]. -At best, some taxa may have been able to exhibit some rudimentary flight skills (e.g. *Sinornithosaurus* and *Zhenyuanlong* [152–154])., Further, the anatomy of most dromaeosaurids, especially larger species, argues for a flightless lifestyle [89].- That eliminates flight as an energy efficient means for a predatory animal to cover the vast distances required to migrate across an ancient Arcticlarge wide geographic areasregion.

This of course would limit the presence of the record of juvenile individuals in the Arctic, unless increased precocial abilities of flight so far unrealistic for juvenile dromaeosaurids are assumedFurther, similar (if not stronger) biomechanical constraints for long-range migrations would apply for these small theropods as it is been advocated for ornithischian dinosaurs ([Fiorillo and Gangloff, 2001]). The fibrous bone texture of DMNH 21183 strongly suggests that the individual was very young at the time of its death. Given that there is also a demonstrable positive correlation between tooth size and body size, at least in some theropods (such as dromaeosaurids, but does not apply to basal ornithomimosaurs and therizinosaurs with extremely tiny teeth compared to body

Formatted: Highlight

Formatted: Highlight

Formatted: Font: Italic

Formatted: Font: Italic

Formatted: Font: Not Italic

Formatted: Highlight

size [Hendrickx pers.,; comm.]) and other diapsids [155, 156], it is reasonable to conclude that the DMNH 21183 belonged to a very small individual. Further, similar (if not stronger) biomechanical constraints for long-range migrations would apply for these small theropods, as has been advocated for ornithischian dinosaurs ([157, 158]Fiorillo and Gangloff, 2001]). Therefore, this specimen of a young, small individual suggests that dromaeosaurs likely nested in the ancient Arctic or in the close proximity, behavior differently than long-distance migratory animals.

Using these observations for interpreting behavior in predatory Arctic dinosaurs, it is reasonable to assume that Arctic dromaeosaurids employed similar life strategies many birds that nest in the and did not migrate. Given the rarity of Arctic fossil vertebrate material, it is not surprising that direct evidence from the fossil record on potential migratory habits in non-avian theropods are scant and based on secondary evidence. For example, the higher abundance and relatively large size (compared to similar, more southern assemblages) of a troodontid taxon in Alaska [89] seems to imply a higher success at these latitudes compared to the southerner relatives. This advantage might be due to increased success favored by high-latitude adaptations like bigger eyes, conferring enhanced yearly activity even at low-light regime, hence increasing resources exploitation for this taxon. The relatively smaller size of the tyrannosaurid *Nanuqsaurus* [21], compared to the average size of other latest Cretaceous tyrannosaurines, might be due to stronger

Formatted: Not Highlight

Formatted: Not Highlight

Formatted: Highlight

Formatted: Highlight

constrained constraints exerted by a lower carrying capacity in this Arctic ecosystem. Under such a scenario, it is not straightforward to infer for non-avian theropods like the herein described Alaskan Saurornitholestinae either 1) perennial habitat occupation, comprising of mating, nesting and foraging or 2) long-distance migratory habits, potentially driven by either resource tracking or reproductive biology. Although direct evidence of nesting and eggs material in Cretaceous Arctic Alaska are lacking, the implication of the biogeography of several dinosaur taxa (e.g., hadrosaurs, ankylosaurs, ceratopsians, pachycephalosaurs, tyrannosaurs, troodontids, and dromaeosaurs) known in both Siberia and Alaska [23, 156], suggests dispersal capabilities through high-latitude land bridges. As both the theropods and ornithischian taxa so far reported in the Arctic are known to build mound nests or practice brooding [157–160, 161], their perennial subsistence at high-latitude seems realistic [160].

The fibrous bone texture of DMNH 21183 strongly suggests that the individual was very young at the time of its death. Given that there is a demonstrable positive correlation between tooth size and body size, at least in some animals [162, 163], it is reasonable to conclude that the DMNH 21183 belonged to a small individual. Therefore, this specimen of a very young, small individual suggests that dromaeosaurs likely nested in the ancient Arctic and did not migrate. They were year-round inhabitants of the ancient high-latitude north.

Teeth assigned to other Dromaeosaurinae and *Saurornitholestes* have been reported from the PCF [89], implying that these clades were likely inhabitants of the paleoarctic region throughout the year rather than seasonal migrants. The higher abundance of Dromaeosaurinae in these northern assemblages relative to Saurornitholestinae (based solely on dental material), led Fiorillo [89] to the hypothesis that the generalist bauplan in Dromaeosaurinae [73] might have been ideal of a low-resources ecosystem as the one in this region during the Late Cretaceous. The frequency of similar taxa in the Judith River Formation of Montana and Alberta [164] implies some form of niche partitioning in similarly sized North American predatory paravians, with the more abundant Saurornitholestinae marginalizing the other taxa in the predatory guild (mostly cfr. *Richardoestesia*, troodontids and *Dromaeosaurus*). In the polar assemblages on the other hand, Saurornitholestinae appear rarer, with troodontids standing out not only for relative frequency but also for larger size in this assemblage [17, 89]. The extreme rarity of Saurornitholestinae in this assemblage may be due to competitive exclusion with the other two main sympatric paravian taxa. While the "generalist bauplan interpretation" attributed to Dromaeosaurinae by Currie [73] might just be due to an older intepretation based on a lower taxonomic sampling than is available today, their higher frequency (taken at face value) might reflect a higher ecological success. The larger size attained by multiple taxa in this clade (e.g. *Atrociraptor*,

Formatted: Left, Indent: First line: 0"

Achillobator, *Utahraptor*, *Dakotaraptor* [80, 81, 139, 141]), and the dentition reminiscent of larger non-maniraptoran theropods (e.g. low DSDI; [26]) might suggest a higher tendency to hypercarnivory than the smaller saurornitholestine. In addition, the hooked denticulation exhibited in this clade may imply a different ecomorphology in this clade relative to Dromaeosaurinae. Originally interpreted as evidence of hypercarnivory [82], the functional implication of hooked denticles in theropod teeth has been differently evaluated. Fowler et al [165] suggested a role in enhancing grip in smaller feathered or furry animals, within taxa having a high numbers of denticles, while the coarser dentition in troodontids is only superficially convergent but implies a different functional role, for example exploitation of smaller and softer preys, like insects or other invertebrates [166] or omnivory [114]. Torices et al [166] suggested for the fine, apically hooked serration of taxa like *Saurornitholestes*, an adaptation for active hypercarnivory of struggling prey. The potential overlap in prey exploitation between Saurornitholestinae and Dromaeosaurinae, but the higher success in developing larger size of the latter, might have caused the marginalization of the smaller saurornitholestines to secondary trophic levels in this low-resource environment of Arctic Alaska. Similarly, the preadaptation to low-light conditions in troodontids and their larger size might have played an advantageous role in segregating this group from other paravians. Further work has the potential not only to provide the

~~first direct evidence of nesting, early life history and development of Arctic dromaeosaurs, but also of casting more light on the systematic and taxonomy of the Alaskan Saurornitholestinae and their trophic role in this extinct Arctic ecosystem.~~

The identification of the first osteological remains attributed to Saurornitholestinae from the North Slope supports previous observations on the paleoecology and ecosystem structure of Late Cretaceous communities in Arctic Alaska. Previous workers ~~have~~ suggested that gregarious herbivorous dinosaur groups inhabiting the Cretaceous Arctic were non-migratory [158]. The specimen described here (DMNH 21183) is particularly important because it represents a very small and young individual with little or limited ability to engage in long-distance travel or migrations. In modern migratory birds, it is often the case that young birds lack experience and they must rely on learning migratory habits from the adults [159]. Also, as another analog,~~s~~ there is a decided preference for modern mammalian long-distance migrations to occur among large-bodied herbivores rather than carnivores. Taken together,~~,~~ we infer that DMNH 21183 implies a perennial residency of this dromaeosaur clade (Saurornitholestinae) in the Arctic. ~~In addition, it is unlikely that juvenile forms of saurornthnitholestines were capable of long-distance migration while adult forms were not. In modern migratory birds, it is often the case that young birds do not have the experience and~~

Formatted: Left

they must rely on learning from the adults [159]. We infer then that DMNH 21183 implies a perennial residency of this dromaeosaur clade (Saurornitholestinae) in the Arctic.

sThis new data pointDMNH 21183 adds further weight to the palaeobiogeographical connection between closely related Asian and North American eudromaeosaur taxa (with sister clades present in both Asia and the Western Interior Basin of North America). DMNH 21183 is too fragmentary to provide more specific taxonomic distinction within the current record of known dromaeosaurids, but is most similar to saurornitholestines. Given the geological age of *Saurornitholestes langstoni* and *Atrociraptor marshalli*, and the wide-ranging tooth-form taxon *Richardoestesia* (Late Campanian-Late Maastrichtian), we predict that additional specimens and data may eventually provide evidence supporting the establishment of a new dromaeosaurid taxon in the Early to early Late Maastrichtian of Arctic Alaska (Fig 10).

Formatted: Font: Italic

Acknowledgments

We would like to acknowledge Renata Tully (Nikon, Inc.) and Ryan Clubb (Keyence Corp. of America) for microscope and software support. Dori Contreras (Perot Museum of Nature and Science) is also thanked for assistance in data collection and photo editing.Andrey Atuchin is acknowledged for the

commisionedcommissioned reconstruction in Fig 10. Thomas Carr (Carthage College, USA) and Christhophe Hendrickx (Unidad Ejecutora Lillo, CONICET-Fundación Miguel Lillo) are also thanked for their thorough reviews which greatly improved the quality of this manuscript. We acknowledge Renata Tully (Nikon, Inc.) and Ryan Clubb (Keyence Corp. of America) for microscope and software support. The phylogenetic software TNT is being made available withthrough the supporting and sponsorship of the Willi Hennig Society.

The specimen used in this study was collected with funding from the National Science Foundation (OPP 0424594 to Fiorillo) and the National Geographic Society (W221-12 to Fiorillo). Additional funding was provided by the Perot Paleo Club. Lastly, the Arctic Management Unit of the Bureau of Land Management provided administrative support. The specimens discussed here wereas collected under BLM permit number s AA-86864AA 86367, AA-093212, and AA-093848.

Formatted: Not Highlight

References

1. Matthew WD, Brown B. The family Deinodontidae, with notice of a new genus from the Cretaceous of Alberta. Bulletin of the American Museum of Natural History. 1922; 46: 367–385.
2. Colbert EH, Russell DA. The small Cretaceous dinosaur *Dromaeosaurus*. American Museum Novitates. 1969; 2380:1–49.
3. Sereno PC. A rationale for phylogenetic definitions, with application ~~tothe~~to the higher−level taxonomy of Dinosauria. Neues Jahrbuch für Geologie und Paläontologie Abhandlungen. 1998; 210: 41–83.
4. Ostrom JH. The ancestry of birds. Nature. 1973; 242:136.
5. Gauthier JA. Saurischian monophyly and the origin of birds. Memoires of the California Academy of Sciences. 1986; 8:1–55.
6. Metcalf SJ, Vaughan RF, Benton MJ, Cole J, Simms MJ, Dartnall DL. A new Bathonian (Middle Jurassic) microvertebrate site, within the Chipping Norton Limestone Formation at Hornsleaslow Quarry, Gloucestershire. Proceedings of the Geologists' Association. 1992; 103 (4): 321–342. doi:10.1016/S0016-7878(08)80129-0

Formatted: Level 1, Indent: First line: 0.5", Space After: 0 pt, Line spacing: Double

7. Cau A. The assembly of the avian body plan: a 160-million-year long process. Bollettino della Società Paleontologica Italiana. 2018; 57 (1): 1–25. doi:10.4435/BSPI.2018.01

8. Hendrickx C., Hartman S. A., Mateus O. An overview of non-avian theropod discoveries and classification. PalArch's Journal of Vertebrate Palaeontology. 2015; 12 (1): 1-73.

9. Fanti F, Therrien F. Theropod tooth assemblages from the Late Cretaceous Maevarano Formation and the possible presence of dromaeosaurids in Madagascar. Acta Palaeontologica Polonica. 2007; 52 (1): 155–166.

10. Brown CM, Evans DC, Campione NE, O'Brien LJ, Eberth DA. Evidence for taphonomic size bias in the Dinosaur Park Formation (Campanian, Alberta), a model Mesozoic terrestrial alluvial- paralic system. Palaeogeography, palaeoclimatology, palaeoecology. 2013; 372, 108-122. doi: 10.1016/j.palaeo.2012.06.027

11. Cashmore DD, Butler RJ. Skeletal completeness of the non- avian theropod dinosaur fossil record. Palaeontology. 2019; 62: 951–981. doi:10.1111/pala.12436

12. Norell MA, Makovicky PJ. Important features of the dromaeosaurid skeleton II: information from newly collected specimens of *Velociraptor mongoliensis*, in American Museum Novitates. 1999; 3282: 1–45

13. Lü J, Brusatte SL. A large, short-armed, winged dromaeosaurid (Dinosauria: Theropoda) from the Early Cretaceous of China and its implications for feather evolution. Scientific Reports. 2015; 5: 11775. doi:10.1038/srep11775

14. Longrich NR, Currie PJ. A microraptorine (Dinosauria–Dromaeosauridae) from the Late Cretaceous of North America. PNAS. 2009; 106 (13): 5002–7. doi:10.1073/pnas.0811664106

15. Clemens WA, Allison CW. Late Cretaceous terrestrial vertebrate fauna, North Slope, Alaska. Geological Society of America Abstracts with Programs. 1985; 17(7):548.

16. Brouwers EM, Clemens WA, Spicer RA, Ager TA, Carter DL, Sliter WV. Dinosaurs on the North Slope of Alaska: high latitude, latest Cretaceous environments. Science. 1987; 237:1608–1610.

17. Davies K. L. Duck-bill dinosaurs (Hadrosauridae, Ornithischia) from the north slope of Alaska. Journal of~~.~~ Paleontology. 1987; 61: 198–200. doi: https://doi.org/10.1017/S0022336000028341

18. Gangloff RA, Fiorillo AR, Norton DW. The first pachycephalosaurine (Dinosauria) from the Arctic of Alaska and its paleogeographic implications. Journal of Paleontology. 2005; 79: 997–1001.

19. Fiorillo AR, Tykoski RS, Currie PJ, ~~Mccarthy~~McCarthy PJ, Flaig P. Description of two partial *Troodon* braincases from the Prince Creek

Formation (Upper Cretaceous), North Slope Alaska. Journal of Vertebrate Paleontology. 2009; 29 (1): 178–187. doi:10.1080/02724634.2009.10010370

20. Gangloff RA, Fiorillo AR. The record of Arctic dinosaurs from northern Alaska, paleogeographic and paleoecologic implications. Journal of Vertebrate Paleontology. 2003; 23: 53A

21. Brown CM, Druckenmiller P. Basal ornithopod (Dinosauria: Ornithischia) teeth from the Prince Creek Formation (early Maastrichtian) of Alaska. Canadian Journal of Earth Sciences. 2011; 48 (9): 1342–1354. doi:10.1139/e11-017. ISSN 0008-4077

22. Fiorillo AR, Tykoski RS. A new Maastrichtian species of the centrosaurine ceratopsid *Pachyrhinosaurus* from the North Slope of Alaska. Acta Palaeontologica Polonica. 2012; 57(3): 561-573. doi: http://dx.doi.org/10.4202/app.2011.0033

23. Fiorillo AR, Tykoski RS. A diminutive new tyrannosaur from the top of the world. PLoS ONE. 2014; 9(3): e91287. doi:10.1371/journal.pone.0091287

24. Watanabe A, Erickson GM, Druckenmiller PS. An ornithomimosaurian from the upper Cretaceous Prince Creek Formation of Alaska. Journal of Vertebrate Paleontology.. 2013; 33: 1169–1175.

25. Fiorillo AR. Alaska Dinosaurs: an Ancient Arctic World Book. CRC Press, Boca Raton. 2018; 224 p.

26. Fiorillo AR. On the occurrence of exceptionally large teeth of *Troodon* (Dinosauria: Saurischia) from the Late Cretaceous of northern Alaska. PALAIOS. 2008; 23:322–328.

27. Torices A, Currie PJ, Canudo JI, Pereda-Suberbiola X. Theropod dinosaurs from the Upper Cretaceous of the South Pyrenees Basin of Spain. Acta Palaeontologica Polonica. 2015; 60 (3): 611–626.

28. Fiorillo AR, Gangloff RA. Theropod teeth from the Prince Creek Formation (Cretaceous) of northern Alaska, with speculations on arctic dinosaur paleoecology. Journal of Vertebrate Paleontology. 2000; 20: 675–682.

29. Hendrickx C, Mateus O, Araújo R. A proposed terminology of theropod teeth (Dinosauria, Saurischia). Journal of Vertebrate Paleontology. 2015; ;35: e982797. doi:10.1080/02724634.2015.982797

30. Sampson SD, Ryan MJ, Tanke DH. Craniofacial ontogeny in centrosaurine dinosaurs (Ornithischia: Ceratopsidae): taxonomic and behavioral implications. Zool. J. Linn. Soc. 1997; 121: 293–337. doi:10.1111/j.1096-3642.1997.tb00340

31. Carr TD. Craniofacial ontogeny in Tyrannosauridae (Dinosauria, Coelurosauria). Journal of Vertebrate Paleontol Paleontology. 1999, 19: 497–520

32. Hone DWE, Farke AA, Wedel MJ. Ontogeny and the fossil record: what, if anything, is an adult dinosaur? Biology Letters. 2016; 12:20150947.

33. Lee MSY, Cau A, Naish D, Dyke GJ. Sustained miniaturization and anatomical innovation in the dinosaurian ancestors of birds. Science. 2014; 345(6196): 562-566.

34. Cau A, Brougham T, Naish D. The phylogenetic affinities of the bizarre Late Cretaceous Romanian theropod *Balaur bondoc* (Dinosauria, Maniraptora): dromaeosaurid or flightless bird? PeerJ. 2015; 3:e1032. https://doi.org/10.7717/peerj.1032

35. Hendrickx C, Tschopp E, Ezcurra M. d. Taxonomic identification of isolated theropod teeth: the case of the shed tooth crown associated with *Aerosteon* (Theropoda: Megaraptora) and the dentition of Abelisauridae. Cretaceous Research. 2020; 108: 104312. https://doi.org/10.1016/j.cretres.2019.104312.

36. Rauhut OWM, Carrano MT. The theropod dinosaur *Elaphrosaurus bambergi* Janensch, 1920, from the Late Jurassic of Tendaguru, Tanzania. Zoological Journal of the Linnean Society. 2016; 178 (3): 546-610

37. Brusatte S, Lloyd G, Wang S, Norell M. Gradual assembly of avian body plan culminated in rapid rates of evolution across the dinosaur–bird transition. Current Biology. 2014; 24:2386-2392.

38. Goloboff PA, Farris JS, Nixon KC. TNT, a free program for phylogenetic analysis. Cladistics. 2008; 24:774-786.

39. Gerke O, Wings O. Multivariate and cladistic analyses of isolated teeth reveal sympatry of theropod dinosaurs in the Late Jurassic of northern Germany. PLoS ONE. 2016; 11(7): e0158334. https://doi.org/10.1371/journal.pone.0158334

40. Larson DW, Currie PJ. Multivariate analyses of small theropod dinosaur teeth and implications for paleoecological turnover through time. PLoS ONE. 2013; 8:e54329. https://doi.org/10.1371/journal.pone.0054329

41. Williamson TE, Brusatte SL. Small theropod teeth from the Late Cretaceous of the San Juan Basin, northwestern New Mexico and their implications for understanding latest Cretaceous dinosaur evolution. PLoS ONE. 2014; 9(4): e93190. https://doi.org/10.1371/journal.pone.0093190

42. Brownstein CD. New records of theropods from the latest Cretaceous of New Jersey and the Maastrichtian Appalachian fauna. Royal Society Open Science. 2019; 6(11):191206.

43. Currie PJ, Evans DC. Cranial anatomy of new specimens of *Saurornitholestes langstoni* (Dinosauria, Theropoda, Dromaeosauridae) from the Dinosaur Park Formation (Campanian) of Alberta. The Anatomical Record. 2020; doi:10.1002/ar.24241

44. Moore MK. Sex estimation and assessment. In: Elizabeth A. DiGangi, Megan K. Moore, editors. Research Methods: Human Skeletal Biology. Academic Press. 2013; pp. 91-116. doi: https://doi.org/10.1016/B978-0-12-385189-5.00004-2

45. Venables W, Ripley B. Modern Applied Statistics with S. 4th ed. New York: Springer. 2002; pp. 138.

46. Gryc G, Patton WW Jr, Payne TG. Present Cretaceous stratigraphic nomenclature of northern Alaska Journal of the Washington Academy of Sciences. 1951; 41: 159-167.

47. Mull GG, Houseknecht DW, Bird KJ. Revised Cretaceous and Tertiary stratigraphic nomenclature in the Colville Basin, northern Alaska: U.S. Geological Survey Professional Paper. 2003; 1673, 36.

48. Detterman RL, Bickel RS, Grvc G. Geology of the Chandler River region, Alaska. U.S., Geological Survey Professional Papers. 1963; 303-E:219-324.

49. Brosge WP, Whittington CL. Geology of the Umiat-Maybe Creek region, Alaska: U.S. Geological Survey Professional Paper. 1966; 303-H, 501–638.

50. Frederiksen NO, Ager TA, Edwards LE. Comment on "Early Tertiary marine fossils from northern Alaska: implications for Arctic Ocean paleogeography and faunal evolution": Geology. 1986; 14: 802-803.

51. Frederiksen NO. Sporomorph biostratigraphy, floral changes, and paleoclimatology, Eocene and earliest Oligocene of the eastern Gulf Coast U.S. Geological Survey Professional Paper. 1988; 1448: 68.

52. Frederiksen NO, Edwards LE, Ager TA, Sheehan TP. Palynology of Eocene strata in the Sagavanirktok and Canning Formations on the North Slope of Alaska. Palynology. 2002; 26: 59-93.

53. Parrish JT, Spicer RA. Late Cretaceous terrestrial vegetation: a near-polar temperature curve. Geology 1988; 16: 22–25.

54. Frederiksen NO. Pollen zonation and correlation of Maastrichtian marine beds and associated strata, Ocean Point dinosaur locality, North Slope, Alaska: U.S. Geological Survey Bulletin. 1991; 1990-E: E1-E24.

55. Brouwers EM, De Deckker P. Late Maastrichtian and Danian ostracode faunas from Northern Alaska: reconstructions of environment and paleogeography. Palaios. 1993; 8: 140–154.

56. Frederiksen NO, McIntyre DJ. Palynomorph biostratigraphy of mid(?)-Campanian to upper Maastrichtian strata along the Colville River, North Slope of Alaska: U.S. Geological Survey Open-File Report. 2000; 2000-493: 36.

57. Flores RM, Myers MD, Houseknecht DW, Stricker GD, Brizzolara DW, Ryherd TJ, Takahashi KI. Stratigraphy and facies of Upper Cretaceous

Schrader Bluff and Prince Creek Formations in the Colville River Bluffs, North Slope, Alaska: U.S. Geological Survey Professional Paper. 2007; 1748: 52

58. Brandlen E. Paleoenvironmental reconstruction of the Late Cretaceous (Maastrichtian) Prince Creek Formation, near the Kikak-Tegoseak dinosaur quarry, North Slope, Alaska: University of Alaska Fairbanks, M.S. thesis. 2008; p. 225.

59. Fiorillo AR, McCarthy PJ, Flaig PP. Taphonomic and sedimentologic interpretations of the dinosaur-bearing Upper Cretaceous strata of the Prince Creek Formation, Northern Alaska: Insights from an ancient high-latitude terrestrial ecosystem. Palaeogeography, Palaeoclimatology, Palaeoecology, 2010; 295:376-388.

60. Conrad JE, McKee EH, Turrin BD. Age of tephra beds at the Ocean Point dinosaur locality, North Slope, Alaska, based on K-Ar and 40Ar/39Ar analyses. US Geological Survey Bulletin, 1992; 1990: 1–12.

61. Flaig PP, McCarthy PJ, Fiorillo AR. Anatomy, evolution, and paleoenvironmental interpretation of an ancient arctic coastal plain: Integrated paleopedology and palynology from the upper Cretaceous (Maastrichtian) Prince Creek Formation, North Slope, Alaska, USA: in Driese, S.G. and Nordt, L.C., eds.: New Frontiers in Paleopedology and

Terrestrial Paleoclimatology, Paleosols and Soil Surface Analogue Systems, SEPM Special Publication. 2013; 114:179–230.

62. Flaig PP, Fiorillo AR, McCarthy PJ, Dinosaur-bearing hyperconcentrated flows of Cretaceous Arctic Alaska: Recurring catastrophic event beds on a distal paleopolar coastal plain: Palaios. 2014; 29: 594-611. doi: 10.2110/palo.2013.133.

63. Flaig PP, Hasiotis ST, Fiorillo AR. A paleopolar dinosaur track site in the Cretaceous (Maastrichtian) Prince Creek Formation of Arctic Alaska: Track characteristics and probable trackmakers: Ichnos. 2018; 25: 208–220. doi: 10.1080/10420940.2017.1337011

64. Flaig PP, McCarthy PJ, Fiorillo AR. A tidally influenced, high-latitude coastal-plain: the Upper Cretaceous (Maastrichtian) Prince Creek Formation, North Slope, Alaska, in Davidson S, Leleu S, North C. eds., From river to rock record: the preservation of fluvial sediments and their subsequent Interpretation: SEPM Special Publication. 2011; 97: 233–264.

65. Salazar-Jaramillo S, McCarthy PJ, Trainor T, Fowell SJ, Fiorillo AR. Origin of clay minerals in alluvial paleosols, Prince Creek Formation, North Slope, Alaska, USA: influence of volcanic ash on pedogenesis in the Late Cretaceous Arctic. Journal of Sedimentary Research. 2015; 85: 192-208.

66. Suarez CA, Flaig PP, Ludvigson GA, Gonzalez LA, Tian R, Zhou H, McCarthy PJ, Van der Kolk DA., Fiorillo AR. Reconstructing the paleohydrology of a Cretaceous Alaskan paleopolar coastal plain from stable isotopes of bivalves. Paleogeography, Palaeoclimatology, Palaeoecology. 2016; 441, 339-351. doi: 10.1016/j.palaeo.2015.07.025.

67. Eberle JJ, Clemens WA, McCarthy PJ, Fiorillo AR, Erickson GM, Druckenmiller PS. Northern-most record of the Metatheria: a new Late Cretaceous pediomyid from the North Slope of Alaska. Journal of Systematic Palaeontology. 2019; 1–20, doi: 10.1080/14772019.2018.1560369.

68. van der Kolk DA, Flaig PP, Hasiotis ST. Paleoenvironmental reconstruction of a Late Cretaceous, muddy, river-dominated polar deltaic system: Schrader Bluff - Prince Creek Formation transition, Shivugak Bluffs, North Slope of Alaska, U.S.A: Journal of Sedimentary Research. 2015; 85: 903–936. doi: 10.2110/jsr.2015.58.

69. Phillips RL. Depositional environments and processes in Upper Cretaceous non-marine and marine sediments, Ocean Point dinosaur locality, North Slope, Alaska. Cretaceous Research. 2003; 24, 499–523.

70. Owen R. Report on British Fossil Reptiles. Report of the British Association for the Advancement of Science. 1842; 11: 60–204. Part 2.

71. Marsh OC. 1881. Classification of the Dinosauria. American Journal of Science 23:241–244

72. Evans DC, Larson DW, Currie PJ. 2013. A new dromaeosaurid (Dinosauria: Theropoda) with Asian affinities from the latest Cretaceous of North America. Naturwissenschaften. 100 (11): 1041-1049. doi: 10.1007/s00114-013-1107-5

73. Samman T, Powell GL, Currie PJ, Hills LV. Morphometry of the teeth of western North American tyrannosaurids and its applicability to quantitative classification. Acta Palaeontologica Polonica. 2005; 50: 757–776.

74. Buckley LG, Larson DW, Reichel M, Samman T. Quantifying tooth variation within a single population of *Albertosaurus sarcophagus* (Theropoda: Tyrannosauridae) and implications for identifying isolated teeth of tyrannosaurids. Canadian Journal of Earth Science. 2010; 47: 1227–1251.

75. Araújo R, Castanhinha R, Martins R, Mateus O, Hendrickx C, Beckmann F et al. Filling the gaps of dinosaur eggshell phylogeny: Late Jurassic theropod clutch with embryos from Portugal. Scientific Reports. 2013; 3: 1924. https://doi.org/10.1038/srep01924

76. Brusatte SL, Benson RBJ, Hutt S. The osteology of *Neovenator salerii* (Dinosauria: Theropoda) from the Wealden group (Barremian) of the Isle of Wight. Monograph of the Palaeontographical Society. 2008;162(631):1–166.

77. Xu X. Avian ancestors - a review of the phylogenetic relationships of the theropods Unenlagiidae, Microraptoria, *Anchiornis* and Scansoriopterygidae. Ameghiniana. 2013; 50(4), 469.

78. Currie PJ. Cranial anatomy of tyrannosaurid dinosaurs from the Late Cretaceous of Alberta, Canada. Acta Palaeontologica Polonica. 2003; 48 (2): 191–226

79. Brochu CR. Osteology of *Tyrannosaurus rex*: insights from a nearly complete skeleton and high-resolution computed tomographic analysis of the skull. Memoirs of the Society of Vertebrate Paleontology. 2003; 7: 1–138. doi: 10.2307/3889334.

80. Currie PJ. New information on the anatomy and relationships of *Dromaeosaurus albertensis* (Dinosauria: Theropoda). Journal of Vertebrate Paleontology. 1995; 15(3):576-591

81. Senter P. A new look at the phylogeny of Coelurosauria (Dinosauria: Theropoda.) Journal of Systematic Palaeontology. 2007, 5(4): 429-463

82. Choiniere JN, Clark JM, Forster CA, Xu X. A basal coelurosaur (Dinosauria: Theropoda) from the Late Jurassic (Oxfordian) of the Shishugou Formation in Wucaiwan, People's Republic of China, Journal of Vertebrate Paleontology. 2010; 30(6): 1773-1796, doi: 10.1080/02724634.2010.520779

83. Hendrickx C, Mateus O, Araújo R, Choiniere J. The distribution of dental features in non-avian theropod dinosaurs: Taxonomic potential, degree of homoplasy, and major evolutionary trends. Palaeontologia Electronica. 2019; 22.3.74: 1–110. doi: https://doi.org/10.26879/820

84. Gianechini FA, Makovicky PJ, Apesteguía S. The teeth of the unenlagiine theropod *Buitreraptor* from the Cretaceous of Patagonia, Argentina, and the unusual dentition of the Gondwanan dromaeosaurids. Acta Palaeontologica Polonica. 2011; 56:279-290.

85. Norell MA, Clark JM, Turner AH, Makovicky PJ, Barsbold R, Rowe T. 2006. A new dromaeosaurid theropod from Ukhaa Tolgod (Ömnögov, Mongolia). American Museum Novitates. 3545:1-51. doi: 10.1206/0003-0082(2006)3545[1:andtfu]2.0.co;2

86. Xu X, Wang X-L, Wu X-C. A dromaeosaurid dinosaur with a filamentous integument from the Yixian Formation of China. Nature 1999; 401 (6750): 262–266. doi:10.1038/45769

87. Agnolín FL, Novas FE. Unenlagiid theropods: are they members of the Dromaeosauridae (Theropoda, Maniraptora)? Anais da Academia Brasileira de Ciências. 2011;83(1):117–162. doi: 10.1590/s0001-37652011000100008.

88. Rauhut OWM, Foth C, Tischlinger H. The oldest *Archaeopteryx* (Theropoda: Avialiae): a new specimen from the Kimmeridgian/Tithonian boundary of Schamhaupten, Bavaria. PeerJ 2018; 6: e4191. doi:10.7717/peerj.4191
89. Turner AH, Pol D, Clarke J, Erickson G, Norell M. A basal dromaeosaurid and size evolution preceding avian flight. Science. 2007; 317:1378–1381.
90. Currie PJ, Varricchio DJ. A new dromaeosaurid from the Horseshoe Canyon Formation (Upper Cretaceous) of Alberta, Canada. 2004; 112–132. In Currie PJ, Koppelhus EB, Shugar MA and Wright JL. (eds.) Feathered Dragons. Indianapolis: Indiana University Press.
91. Currie PJ. 1987. Bird-like characteristics of the jaws and teeth of troodontid theropods (Dinosauria, Saurischia). Journal of Vertebrate Paleontology. 1987; 7: 72–81
92. Currie PJ, Rigby JK Jr, Sloan R. Theropod teeth from the Judith River Formation of southern Alberta, Canada; 1990; 107–125 in K. Carpenter and P. J. Currie (eds.), Dinosaur Systematics: Approaches and Perspectives. Cambridge University Press, New York.
93. Averianov AO, Sues H-D. A new troodontid (Dinosauria: Theropoda) from the Cenomanian of Uzbekistan, with a review of troodontid records from the territories of the former Soviet Union. Journal of Vertebrate Paleontology.

2007; 27 (1): 87–98. doi:10.1671/0272-4634(2007)27[87:ANTDTF]2.0.CO;2

94. Lü J, Xu L, Liu Y, Zhang X, Jia S, Ji Q. A new troodontid theropod from the Late Cretaceous of central China, and the radiation of Asian troodontids. Acta Palaeontologica Polonica, 2010; 55:381-388.

95. Xu X, Li F. A new microraptorine specimen (Theropoda: Dromaeosauridae) with a brief comment on the evolution of compound bones in theropods. Vertebrata PalAsiatica, 2016; 54:269-285.

96. Larson DW. Diversity and variation of theropod dinosaur teeth from the uppermost Santonian Milk River Formation (Upper Cretaceous), Alberta: a quantitative method supporting identification of the oldest dinosaur tooth assemblage in Canada. Canadian Journal of Earth Sciences. 2008; 45:1455-1468. doi: 10.1139/e08-070

97. Currie PJ, Dong Z. 2001. New information on *Shanshanosaurus huoyanshanensis*, a juvenile tyrannosaurid (Theropoda, Dinosauria) from the Late Cretaceous of China. Canadian Journal of Earth Sciences. 2991; 38:1729-1737. doi: 10.1139/e01-042

98. Sankey JT. Diversity of Latest Cretaceous (Late Maastrichtian) small theropods and birds: teeth from the Lance and Hell Creek Formations, USA. 2008; p. 117-134. In Sankey, JT, Baszio, S. (eds.), Vertebrate Microfossil

Assemblages: Their Role in Paleoecology and Paleobiogeography. Indiana University Press, Bloomington, Indiana.

99. Varricchio DJ, Horner JR, Jackson FD. Embryos and eggs for the Cretaceous theropod dinosaur *Troodon formosus*. Journal of Vertebrate Paleontology, 2002; 22:564-576. Doi: 10.1671/0272-4634(2002)022[0564:eaeftc]2.0.co;2

100. Cau A, Beyrand V, Voeten, D, Fernandez V, Tafforeau P, Stein K, et al. Synchrotron scanning reveals amphibious ecomorphology in a new clade of bird-like dinosaurs. Nature. 2017; 552 (7685): 395–399. doi: 10.1038/nature24679

101. Turner AH, Makovicky PJ, Norell M. A review of dromaeosaurid systematics and paravian phylogeny. Bulletin of the American Museum of Natural History. 2012; 371: 1–206. doi: 10.1206/748.1

102. Novas FE, Pol D, Canale JI, Porfiri JD, Calvo JO. A bizarre Cretaceous theropod dinosaur from Patagonia and the evolution of Gondwanan dromaeosaurids. Proceedings of the Royal Society B: Biological Sciences. 2009; 276:1101-1107. doi: 10.1098/rspb.2008.1554

103. Xu X, Norell MA. A new troodontid dinosaur from China with avian-like sleeping posture. Nature. 2004; 431:838-841. doi: 10.1038/nature02898

104. Makovicky PJ, Norell MA, Clark JM, Rowe T. Osteology and relationships of *Byronosaurus jaffei* (Theropoda: Troodontidae). American Museum Novitates. 2003; 3402:1-32. https://doi.org/10.1206/0003-0082(2003)402%3C0001:oarobj%3E2.0.co;2

105. Tsuihiji T, Barsbold R, Watabe M, Tsogtbaatar K, Chinzorig T, Fujiyama Y, Suzuki S. An exquisitely preserved troodontid theropod with new information on the palatal structure from the Upper Cretaceous of Mongolia. Naturwissenschaften. 2014; 101:131-142. doi: 10.1007/s00114-014-1143-9

106. Ji Q, Ji S, Lü J, You H, Chen W, Liu Y, Liu Y. First avialian bird from China. Geological Bulletin of China. 2005; 24:197-210.

107. Pei R, Li Q, Meng Q, Norell M, Gao K. 2017. New specimens of *Anchiornis huxleyi* (Theropoda, Paraves) from the late Jurassic of northeastern China. Bulletin of the American Museum of Natural History, 411:1–66. doi: 10.1206/0003-0090-411.1.1

108. Pei R, Norell MA, Barta DE, Bever GS, Pittman M, Xu X. Osteology of a new Late Cretaceous troodontid specimen from Ukhaa Tolgod, Ömnögovi Aimag, Mongolia. American Museum Novitates, 2017; 3889:1–47. doi: 10.1206/3889.1

109. Hu D, Hou L, Zhang L, Xu X. A pre-*Archaeopteryx* troodontid theropod from China with long feathers on the metatarsus. Nature, 2009; 461:640–643. doi: 10.1038/nature08322

110. Godefroit P, Demuynck H, Dyke G, Hu D, Escuillié F, Claeys P. Reduced plumage and flight ability of a new Jurassic paravian theropod from China. Nature Communications. 2013; 4:1394. doi: 10.1038/ncomms2389

111. Godefroit P, Cau A, Dong-Yu H, Escuillié F, Wenhao W, Dyke G. A Jurassic avialan dinosaur from China resolves the early phylogenetic history of birds. Nature. 2013; 498:359-362. doi: 10.1038/nature12168

112. Martin LD, Stewart JD, Whetstone KN. The origin of birds: structure of the tarsus and teeth. The Auk. 1980; 97:86-93.

113. Elzanowski A, Wellnhofer P. Cranial morphology of *Archaeopteryx*: evidence from the seventh skeleton. Journal of Vertebrate Paleontology. 1996; 16: 81-94. doi: 10.1080/02724634.1996.10011286

114. Mayr G, Pohl B, Hartman S, Peters DS. The tenth skeletal specimen of *Archaeopteryx*. Zoological Journal of the Linnean Society. 2007; 149:97-116. doi: 10.1111/j.1096-3642.2006.00245.x

115. Rauhut OWM, Werner C. First record of the family Dromaeosauridae (Dinosauria: Theropoda) in the Cretaceous of Gondwana (Wadi Milk

Formation, northern Sudan). Paläontologische Zeitschrift. 1995; 69:475-489. doi: 10.1007/BF02987808

116. Sweetman SC. 2004. The first record of velociraptorine dinosaurs (Saurischia, Theropoda) from the Wealden (Early Cretaceous, Barremian) of southern England. Cretaceous Research, 25:353–364. doi: 10.1016/j.cretres.2004.01.004

117. Vullo R, Néraudeau, D, Lenglet T. Dinosaur teeth from the Cenomanian of Charentes, Western France: evidence for a mixed Laurasian-Gondwanan assemblage. Journal of Vertebrate Paleontology. 2007; 27:931-943. doi10.1671/0272-4634(2007)27[931:dtftco]2.0.co;2

118. van der Lubbe T, Richter U, Knötschke N. Velociraptorine dromaeosaurid teeth from the Kimmeridgian (Late Jurassic) of Germany. Acta Palaeontologica Polonica. 2009; 54:401-408. doi: 10.4202/app.2008.0007

119. Xu X, Tan Q, Sullivan C, Han F, Xiao D. 2011. A short-armed troodontid dinosaur from the Upper Cretaceous of Inner Mongolia and its implications for troodontid evolution. PLoS ONE. 2011; 6:e22916. https://doi.org/10.1371/journal.pone.0022916

120. Norell MA, Makovicky PJ, Bever GS, Balanoff AM, Clark JM, Barsbold R, Rowe T. A review of the Mongolian Cretaceous dinosaur

Saurornithoides (Troodontidae: Theropoda). American Museum Novitates. 2009; 3654:1-63. https://doi.org/10.1206/648.1

121. Longrich N. Small theropod teeth from the Lance Formation of Wyoming, USA. 2008. In Sankey, J.T. and Baszio, S. editors. Vertebrate Microfossil Assemblages: Their Role in Paleoecology and Paleobiogeography. Indiana University Press, Bloomington, Indiana. pp. 135-158.

122. Sankey JT. Late Campanian southern dinosaurs, Aguja Formation, Big Bend, Texas. Journal of Paleontology. 2001; 75:208-215. doi: 10.1017/s0022336000031991

123. Baszio S. Systematic palaeontology of isolated dinosaur teeth from the latest Cretaceous of south Alberta, Canada. Courier Forschungsinstitut Senckenberg. 1997; 196:33-77.

124. Smith JB, Vann DR, Dodson P. Dental morphology and variation in theropod dinosaurs: Implications for the taxonomic identification of isolated teeth. The Anatomical Record Part A: Discoveries in Molecular, Cellular, and Evolutionary Biology. 2005; 285A:699-736. doi: 10.1002/ar.a.20206

125. Zanno LE. A taxonomic and phylogenetic re-evaluation of Therizinosauria (Dinosauria: Maniraptora). Journal of Systematic Palaeontology. 2010; 8:503-543. doi: 10.1080/14772019.2010.488045

126. Rauhut OWM, Foth C, Tischlinger H, Norell MA. Exceptionally preserved juvenile megalosauroid theropod dinosaur with filamentous integument from the Late Jurassic of Germany. Proceedings of the National Academy of Sciences. 2012; 109:11746-11751. doi: 10.1073/pnas.1203238109

127. Choiniere JN, Clark JM, Forster CA, Norell MA, Eberth DA, Erickson GM, et al. A juvenile specimen of a new coelurosaur (Dinosauria: Theropoda) from the Middle-Late Jurassic Shishugou Formation of Xinjiang, People's Republic of China. Journal of Systematic Palaeontology. 2014; 12:177-215. doi: 10.1080/14772019.2013.781067

128. Barsbold R. Saurornithoididae, a new family of small theropod dinosaurs from central Asia and North America. Palaeontologia Polonica, 1974; 30:5–22.

129. Currie PJ, Dong Z. New information on Cretaceous troodontids (Dinosauria, Theropoda) from the People's Republic of China. Canadian Journal of Earth Sciences. 2001; 38:1753–1766. doi: 10.1139/e01-065

130. Tsuihiji T, Watabe M, Tsogtbaatar K, Tsubamoto T, Barsbold R, Suzuki S et al. Cranial osteology of a juvenile specimen of *Tarbosaurus bataar* (Theropoda, Tyrannosauridae) from the Nemegt Formation (Upper Cretaceous) of Bugin Tsav, Mongolia. Journal of Vertebrate Paleontology. 2011; 31:497-517. https://doi.org/10.1080/02724634.2011.557116

131. Carr TD, Williamson TE. Diversity of late Maastrichtian Tyrannosauridae (Dinosauria: Theropoda) from western North America. Zoological Journal of the Linnean Society. 2004; 142:479-523. doi: 10.1111/j.1096-3642.2004.00130.x

132. Tumarkin-Deratzian AR. Histological evaluation of ontogenetic bone surface texture changes in the frill of *Centrosaurus apertus*. In New Perspectives on Horned Dinosaurs, the Royal Tyrrell Museum Ceratopsian Symp. Bloomington, Indiana University Press. 2010; 251–263.

133. Brown CM, Russell AP, Ryan MJ. Pattern and transition of surficial bone texture of the centrosaurine frill and their ontogenetic and taxonomic implications. Journal of. Vertebrate. Paleontology. 2009; 29: 132–141. doi:10.1671/039.029.0119

134. Bennett SC. The ontogeny of *Pteranodon* and other pterosaurs. Paleobiology. 1993; 19 (1): 92–106.

135. Dal Sasso C, Maganuco S. *Scipionyx samniticus* (Theropoda: Compsognathidae) from the Lower Cretaceous of Italy: Osteology, ontogenetic assessment, phylogeny, soft tissue anatomy, taphonomy, and palaeobiology, Memorie della Società italiana di Scienze naturali e del Museo civico di Storia naturale di Milano, 2011; 34 (1).

136. Göhlich UB, Chiappe LM. A new carnivorous dinosaur from the Late Jurassic Solnhofen archipelago. Nature. 2006; 440 (7082): 329–332. doi:10.1038/nature04579.

137. Chiappe LM, Göhlich, U.B. Anatomy of *Juravenator starki* (Theropoda: Coelurosauria) from the Late Jurassic of Germany. Neues Jahrbuch für Geologie und Paläontologie - Abhandlungen. 2010; 258 (3): 257–296. doi:10.1127/0077-7749/2010/0125

138. Hartman S, Mortimer M, Wahl WR, Lomax DR, Lippincott J, Lovelace DM. A new paravian dinosaur from the Late Jurassic of North America supports a late acquisition of avian flight. PeerJ. 2019; 7:e7247 https://doi.org/10.7717/peerj.7247

139. Jasinski SE, Sullivan RM, Dodson P. New dromaeosaurid dinosaur (Theropoda, Dromaeosauridae) from New Mexico and biodiversity of dromaeosaurids at the end of the Cretaceous. Scientific Reports, 2020; 10, 5105. https://doi.org/10.1038/s41598-020-61480-7

140. Fanti F, Miyashita T. A high latitude vertebrate fossil assemblage from the Late Cretaceous of west–central Alberta, Canada: evidence for dinosaur nesting and vertebrate latitudinal gradient. Palaeogeography, Palaeoclimatology, Palaeoecology. 2009; 275(1–4):37–53.

141. Peng J, Russell AP, Brinkman DB. Vertebrate microsite assemblages (exclusive of mammals) from the Foremost and Oldman Formations of the Judith River Group (Campanian) of southeastern Alberta: an illustrated guide. Provincial Museum of Alberta, Natural History Occasional Paper. 2001; 25:1–54.

142. Larson, D. W.; Brinkman, D. B.; Bell, P. R. Faunal assemblages from the upper Horseshoe Canyon Formation, an early Maastrichtian cool-climate assemblage from Alberta, with special reference to the *Albertosaurus sarcophagus* bonebed. Canadian Journal of Earth Sciences. 2010; 47 (9): 1159–1181. doi:10.1139/e10-005

143. Russell DA. The environments of Canadian dinosaurs. Canadian Geographical Journal. 1973; 87: 4–11.

144. Paul GS. Migration. In The Encyclopedia of Dinosaurs, Edited by: Currie P, Padian K. 1997; 444–446. San Diego, CA: Academic Press

145. Bell PR, Snively E. Polar dinosaurs on parade: a review of dinosaur migration, Alcheringa: An Australasian Journal of Palaeontology. 2008; 32(3): 271–284, doi: 10.1080/03115510802096101

146. Rappole, J.H. The avian migrant: the biology of bird migration. Columbia University Press. 2013.

147. Gudmundsson, G.A. and Alerstam, T. Why is there no transpolar bird migration? Journal of Avian Biology. 2018; 93-96.

148. Gangloff RA, Fiorillo AR. Taphonomy and paleoecology of a bonebed from the Prince Creek Formation, North Slope, Alaska. Palaios. 2010; 25: 299–317.

149. Suarez CA, Ludvigson GA, Gonzalez LA, Fiorillo AR, Flaig PP, McCarthy PJ. 2013. Use of multiple isotope proxies for elucidating Arctic Cretaceous paleohydrology. In Bojar AV, Melinte-Dobrinescu MC, Smit J. editors. Isotopic Studies in Cretaceous Research: Geological Society of London Special Publication. 2013; 382:185–202.

150. Avgar T, Street G, Fryxell JM, On the adaptive benefits of mammal migration. Canadian Journal of Zoology. 2014; 92(6): 481-490.

151. Xu X, Zhou Z, Wang X, Kuang X, Zhang F, Du X. Four winged dinosaurs from China. Nature. 2003; 421:335–340.

152. Dyke G, de Kat R, Palmer C, van der Kindere J, Naish D, Ganapathisubramani, B. Aerodynamic performance of the feathered dinosaur *Microraptor* and the evolution of feathered flight. Nature Communications. 2013; 4 (2489). doi:10.1038/ncomms3489

153. Alexander, D. E., Gong, E., Martin, L. D., Burnham, D. A. & Falk, A. R. Model tests of gliding with different hindwing configurations in the four-winged dromaeosaurid *Microraptor gui*. Proceedings of the National Academy of Science. 2010; 107, 2972-2976.

154. Xu X, Wang X-L, Wu X-C. A dromaeosaurid dinosaur with filamentous integument from the Yixian Formation of China. Nature. 1999; 401:262–266.

155. Mateo JA, Lopez- Jurado LF. Dental ontogeny in *Lacerta lepida* (Sauria, Lacertidae) and its relationship to diet. Copeia. 1997; 461–463.

156. Townsend V, Akin J, Felgenhauer B, Dauphine J, Kidder S. Dentition of the ground skink, *Scincella lateralis* (Sauria, Scincidae). Copeia. 1999; (3): 783–788. doi:10.2307/1447615

157. Horner JR. Evidence of colonial nesting and 'site fidelity' among ornithischian dinosaurs. Nature. 1982; 297(5868):675-676.

158. Fiorillo AR, Gangloff RA. The caribou migration model for Arctic hadrosaurs (Ornithischia: Dinosauria): a reassessment. Historical Biology. 2001; 15:323-334.

159. Baker RR. The significance of the lesser black-backed gull to models of bird migration. Bird Study, 1980; 27(1), pp.41-50.

~~1. Matthew WD, Brown B. The family Deinodontidae, with notice of a new genus from the Cretaceous of Alberta. Bulletin of the American Museum of Natural History. 1922; 46: 367–385.~~

~~2. Colbert EH, Russell DA. The small Cretaceous dinosaur *Dromaeosaurus*. American Museum Novitates 1969; 2380:1–49.~~

~~3. Sereno PC. A rationale for phylogenetic definitions, with application tothe higher–level taxonomy of Dinosauria. Neues Jahrbuch für Geologieund Paläontologie Abhandlungen. 1998; 210: 41–83.~~

~~4. Ostrom JH. The ancestry of birds. Nature. 1973; 242:136.~~

~~5. Gauthier JA. Saurischian monophyly and the origin of birds. Memoires of the California Academy of Sciences. 1986; 8:1–55.~~

~~6. Metcalf SJ, Vaughan RF, Benton MJ, Cole J, Simms MJ, Dartnall DL. A new Bathonian (Middle Jurassic) microvertebrate site, within the Chipping Norton Limestone Formation at Hornsleaslow Quarry, Gloucestershire. Proceedings of the~~

Formatted: Indent: First line: 0.5"

Geologists' Association. 1992; 103 (4): 321–342. doi:10.1016/S0016-7878(08)80129-0

7. Cau A. The assembly of the avian body plan: a 160-million-year long process, in Bollettino della Società Paleontologica Italiana. 57 (1): 1–25. doi:10.4435/BSPI.2018.01

8. Fanti F, Therrien F. Theropod tooth assemblages from the Late Cretaceous Maevarano Formation and the possible presence of dromaeosaurids in Madagascar. Acta Palaeontologica Polonica. 2007; 52 (1): 155–166.

9. Brown CM, Evans DC, Campione NE, O'Brien LJ, Eberth DA. Evidence for taphonomic size bias in the Dinosaur Park Formation (Campanian, Alberta), a model Mesozoic terrestrial alluvial‐paralic system. Palaeogeography, palaeoclimatology, palaeoecology. 2013; 372, 108-122. doi: 10.1016/j.palaeo.2012.06.027

10. Cashmore DD, Butler RJ. Skeletal completeness of the non‐avian theropod dinosaur fossil record. Palaeontology. 2019; 62: 951–981. doi:10.1111/pala.12436

11. Norell MA, Makovicky PJ. Important features of the dromaeosaurid skeleton II: information from newly collected specimens of *Velociraptor mongoliensis*, in American Museum Novitates. 1999; 3282: 1–45

12. Lü J, Brusatte SL. A large, short-armed, winged dromaeosaurid (Dinosauria: Theropoda) from the Early Cretaceous of China and its implications for feather evolution. Scientific Reports. 2015; 5: 11775. doi:10.1038/srep11775

13. Clemens WA, Allison CW. Late Cretaceous terrestrial vertebrate fauna, North Slope, Alaska. Geological Society of America Abstracts with Programs. 1985; 17(7):548.

14. Brouwers EM, Clemens WA, Spicer RA, Ager TA, Carter DL, Sliter WV. Dinosaurs on the North Slope of Alaska: high latitude, latest Cretaceous environments. Science. 1987; 237:1608–1610.

15. Davies K. Duck-bill dinosaurs (Hadrosauridae, Ornithischia) from the north slope of Alaska. J. Paleont. 1987; 61: 198–200

Formatted: Font: Italic

16. Gangloff RA, Fiorillo AR, Norton DW. The first pachycephalosaurine (Dinosauria) from the Arctic of Alaska and its paleogeographic implications. Journal of Paleontology. 2005; 79: 997–1001.

17. Fiorillo AR, Tykoski RS, Currie PJ, Mccarthy PJ, Flaig P. Description of two partial *Troodon* braincases from the Prince Creek Formation (Upper Cretaceous), North Slope Alaska. Journal of Vertebrate Paleontology. 2009; 29 (1): 178–187. doi:10.1080/02724634.2009.10010370

18. Gangloff RA, Fiorillo AR. The record of Arctic dinosaurs from northern Alaska, paleogeographic and paleoecologic implications. J Vertebr Paleontol. 2003; 23: 53A

19. Brown CM, Druckenmiller P. Basal ornithopod (Dinosauria: Ornithischia) teeth from the Prince Creek Formation (early Maastrichtian) of Alaska. Canadian Journal of Earth Sciences. 2011; 48 (9): 1342–1354. doi:10.1139/e11-017. ISSN 0008-4077

20. Fiorillo AR, Tykoski RS. A new Maastrichtian species of the centrosaurine ceratopsid *Pachyrhinosaurus* from the North Slope of Alaska. Acta

Formatted: Font: Italic

~~Palaeontologica Polonica. 2012; 57(3): 561-573. doi: http://dx.doi.org/10.4202/app.2011.0033~~

~~21. Fiorillo AR, Tykoski RS. A diminutive new tyrannosaur from the top of the world. PLoS ONE. 2014; 9(3): e91287. doi:10.1371/journal.pone.0091287~~

~~22. Watanabe A, Erickson GM, Druckenmiller PS. An ornithomimosaurian from the upper Cretaceous Prince Creek Formation of Alaska. J Vertebr Paleontol. 2013; 33: 1169–1175.~~

~~23. Fiorillo AR. Alaska Dinosaurs: an ancient Arctic world book. CRC Press, Boca Raton. 2018; 224 p.~~

~~24. Torices A, Currie PJ, Canudo JI, Pereda-Suberbiola X. Theropod dinosaurs from the Upper Cretaceous of the South Pyrenees Basin of Spain. Acta Palaeontologica Polonica. 2015; 60 (3): 611–626.~~

~~25. Fiorillo AR, Gangloff RA. Theropod teeth from the Prince Creek Formation (Cretaceous) of northern Alaska, with speculations on arctic dinosaur paleoecology. J Vertebr Paleontol. 2000; 20: 675–682.~~

26. Hendrickx C, Mateus O, Araújo R, Choiniere J. The distribution of dental features in non-avian theropod dinosaurs: Taxonomic potential, degree of homoplasy, and major evolutionary trends. Palaeontologia Electronica. 2019; 22.3.74: 1–110. doi: https://doi.org/10.26879/820

27. Sampson SD, Ryan MJ, Tanke DH. Craniofacial ontogeny in centrosaurine dinosaurs (Ornithischia: Ceratopsidae): taxonomic and behavioral implications. Zool. J. Linn. Soc. 1997; 121: 293–337. (doi:10.1111/j.1096-3642.1997.tb00340.

28. Carr TD. Craniofacial ontogeny in Tyrannosauridae (Dinosauria, Coelurosauria). J Vertebr Paleontol. 1999, 19: 497–520

29. Hone DWE, Farke AA, Wedel MJ. Ontogeny and the fossil record: what, if anything, is an adult dinosaur? Biol Lett. 2016;12:20150947.

30. Larson DW, Currie PJ. Multivariate analyses of small theropod dinosaur teeth and implications for paleoecological turnover through time. PLoS ONE. 2013; 8:e54329. https://doi.org/10.1371/journal.pone.0054329

31. Williamson TE, Brusatte SL. Small Theropod Teeth from the Late Cretaceous of the San Juan Basin, Northwestern New Mexico and Their Implications for Understanding Latest Cretaceous Dinosaur Evolution. PLoS ONE. 2014; 9(4): e93190. https://doi.org/10.1371/journal.pone.0093190

32. Brownstein CD. New records of theropods from the latest Cretaceous of New Jersey and the Maastrichtian Appalachian fauna. Royal Society Open Science. 2019; 6(11):191206.

33. Owen R. Report on British Fossil Reptiles. Report of the British Association for the Advancement of Science. 1842; 11: 60–204. Part 2.

34. Marsh OC. 1881. Classification of the Dinosauria. American Journal of Science 23:241–244

35. Longrich NR, Currie PJ. A microraptorine (Dinosauria–Dromaeosauridae) from the Late Cretaceous of North America. PNAS. 2009; 106 (13): 5002–7. doi:10.1073/pnas.0811664106

36. Gryc G, Patton WW Jr, Payne TG. 1951. Present Cretaceous stratigraphic nomenclature of northern Alaska Journal of the Washington Academy of Sciences, 41: 159-167

37. Mull GG, Houseknecht DW, Bird KJ. Revised Cretaceous and Tertiary stratigraphic nomenclature in the Colville Basin, northern Alaska: U.S. Geological Survey Professional Paper. 2003; 1673, 36 p.

38. Detterman RL, Bickel RS, Grvc G. Geology of the Chandler River region, Alaska. U.S., Geol Surv Profess Papers. 1963; 303-E:219-324.

39. Brosge WP, Whittington CL. Geology of the Umiat-Maybe Creek region, Alaska: U.S. Geological Survey Professional Paper. 1966; 303-H, 501–638,

40. Frederiksen NO, Ager TA, Edwards LE. Comment on "Early Tertiary marine fossils from northern Alaska: hnplications for Arctic Ocean paleogeography and faunal evolution": Geology. 1986; 14: 802-803.

41. Frederiksen NO. Sporomorph biostratigraphy, floral changes, and paleoclimatology, Eocene and earliest Oligocene of the eastern Gulf Coast U.S. Geol Surv Prof Pap. 1988; 1448: 68

42. Frederiksen NO, Edwards LE, Ager TA, Sheehan TP. Palynology of Eocene strata in the Sagavanirktok and Canning Formations on the North Slope of Alaska. Palynology. 2002; 26: 59-93.

43. Parrish JT, Spicer RA. Late Cretaceous terrestrial vegetation: a near-polar temperature curve. Geology 1988; 16: 22–25.

44. Frederiksen NO. Pollen zonation and correlation of Maastrichtian marine beds and associated strata, Ocean Point dinosaur locality, North Slope, Alaska: U.S. Geological Survey Bulletin. 1991; 1990-E: E1-E24.

45. Brouwers EM, De Deckker P. Late Maastrichtian and Danian Ostracode Faunas from Northern Alaska: Reconstructions of Environment and Paleogeography. Palaios. 1993; 8: 140–154

46. Frederiksen NO, McIntyre DJ. Palynomorph biostratigraphy of mid(?)-Campanian to upper Maastrichtian strata along the Colville River, North Slope of Alaska: U.S. Geological Survey Open-File Report. 2000; 2000-493: 36

47. Flores RM, Myers MD, Houseknecht DW, Stricker GD, Brizzolara DW, Ryherd TJ, Takahashi KI. Stratigraphy and facies of Upper Cretaceous Schrader Bluff and Prince Creek Formations in the Colville River Bluffs, North Slope, Alaska: U.S. Geological Survey Professional Paper. 2007; 1748: 52

48. Brandlen E. Paleoenvironmental reconstruction of the Late Cretaceous (Maastrichtian) Prince Creek Formation, near the Kikak-Tegoseak dinosaur quarry, North Slope, Alaska: University of Alaska Fairbanks, M.S. thesis. 2008; p. 225.

49. Fiorillo AR, McCarthy PJ, Flaig PP. Taphonomic and sedimentologic interpretations of the dinosaur-bearing Upper Cretaceous strata of the Prince Creek Formation, Northern Alaska: Insights from an ancient high-latitude terrestrial ecosystem. Palaeogeography, Palaeoclimatology, Palaeoecology, 2010; 295:376-388.

50. Conrad JE, McKee EH, Turrin BD. Age of tephra beds at the Ocean Point dinosaur locality, North Slope, Alaska, based on K-Ar and 40Ar/39Ar analyses. US Geological Survey Bulletin, 1992; 1990: 1–12.

51. Flaig PP, McCarthy PJ, Fiorillo AR. Anatomy, evolution, and paleoenvironmental interpretation of an ancient arctic coastal plain: Integrated paleopedology and palynology from the upper Cretaceous (Maastrichtian) Prince Creek Formation, North Slope, Alaska, USA: in Driese, S.G. and Nordt, L.C., eds.: New Frontiers in Paleopedology and Terrestrial Paleoclimatology, Paleosols and Soil Surface Analogue Systems, SEPM Special Publication. , 2013; 114:179–230.

52. Flaig PP, Fiorillo AR, McCarthy PJ, Dinosaur-bearing hyperconcentrated flows of Cretaceous Arctic Alaska: Recurring catastrophic event beds on a distal paleopolar coastal plain: Palaios. 2014; 29: 594-611. doi: 10.2110/palo.2013.133.

53. Flaig PP, Hasiotis ST, Fiorillo AR. A paleopolar dinosaur track site in the Cretaceous (Maastrichtian) Prince Creek Formation of Arctic Alaska: Track characteristics and probable trackmakers: Ichnos. 2018; 25: 208–220. doi: 10.1080/10420940.2017.1337011

54. Flaig PP, McCarthy PJ, Fiorillo AR. A tidally influenced, high-latitude coastal-plain: the Upper Cretaceous (Maastrichtian) Prince Creek Formation, North Slope, Alaska, in Davidson S, Leleu S, North C. eds., From river to rock record: the preservation of fluvial sediments and their subsequent Interpretation: SEPM Special Publication. 2011; 97: 233–264.

55. Salazar-Jaramillo S, McCarthy PJ, Trainor T, Fowell SJ, Fiorillo AR. Origin of clay minerals in alluvial paleosols, Prince Creek Formation, North Slope, Alaska, USA: influence of volcanic ash on pedogenesis in the Late Cretaceous Arctic. Journal of Sedimentary Research. 2015; 85: 192-208.

56. Suarez CA, Flaig PP, Ludvigson GA, Gonzalez LA, Tian R, Zhou H, McCarthy PJ, Van der Kolk DA., Fiorillo AR. Reconstructing the paleohydrology of a Cretaceous Alaskan paleopolar coastal plain from stable isotopes of bivalves. Paleogeography, Palaeoclimatology, Palaeoecology. 2016; 441, 339-351. doi: 10.1016/j.palaeo.2015.07.025.

57. Eberle JJ, Clemens WA, McCarthy PJ, Fiorillo AR, Erickson GM, Druckenmiller PS. Northern-most record of the Metatheria: a new Late Cretaceous

pediomyid from the North Slope of Alaska. Journal of Systematic Palaeontology. 2019; 1–20, doi: 10.1080/14772019.2018.1560369.

58. van der Kolk DA, Flaig PP, Hasiotis ST. Paleoenvironmental reconstruction of a Late Cretaceous, muddy, river-dominated polar deltaic system: Schrader Bluff - Prince Creek Formation transition, Shivugak Bluffs, North Slope of Alaska, U.S.A: Journal of Sedimentary Research. 2015; 85: 903–936. doi: 10.2110/jsr.2015.58.

59. Phillips RL. Depositional environments and processes in Upper Cretaceous nonmarine andmarine sediments, Ocean Point dinosaur locality, North Slope, Alaska. Cretac Res. 2003; 24, 499–523.

60. Currie PJ, Evans DC. Cranial Anatomy of New Specimens of *Saurornitholestes langstoni* (Dinosauria, Theropoda, Dromaeosauridae) from the Dinosaur Park Formation (Campanian) of Alberta. Anat Rec. 2020; doi:10.1002/ar.24241

61. Evans DC, Larson DW, Currie PJ. 2013. A new dromaeosaurid (Dinosauria: Theropoda) with Asian affinities from the latest Cretaceous of North

America. Naturwissenschaften. 100 (11): 1041-1049. doi: 10.1007/s00114-013-1107-5

62. Tumarkin-Deratzian AR. Histological evaluation of ontogenetic bone surface texture changes in the frill of *Centrosaurus apertus*. In New Perspectives on Horned Dinosaurs, the Royal Tyrrell Museum Ceratopsian Symp. Bloomington, Indiana University Press. 2010; 251–263.

63. Brown CM, Russell AP, Ryan MJ. Pattern and transition of surficial bone texture of the centrosaurine frill and their ontogenetic and taxonomic implications. J. Vertebr. Paleontol. 2009; 29: 132–141. doi:10.1671/039.029.0119

64. Bennett SC. The ontogeny of *Pteranodon* and other pterosaurs. Paleobiology. 1993; 19 (1): 92–106.

65. Dal Sasso C, Maganuco S. *Scipionyx samniticus* (Theropoda: Compsognathidae) from the Lower Cretaceous of Italy: Osteology, ontogenetic assessment, phylogeny, soft tissue anatomy, taphonomy, and palaeobiology, Memorie della Società italiana di Scienze naturali e del Museo civico di Storia naturale di Milano, 2011; 34 (1).

Formatted: Font: Italic

~~66. Göhlich UB, Chiappe LM. A new carnivorous dinosaur from the Late Jurassic Solnhofen archipelago. Nature. 2006; 440 (7082): 329–332. doi:10.1038/nature04579.~~

~~67. Chiappe LM, Göhlich, U.B. 2010. Anatomy of *Juravenator starki* (Theropoda: Coelurosauria) from the Late Jurassic of Germany. Neues Jahrbuch für Geologie und Paläontologie - Abhandlungen. 258 (3): 257–296. doi:10.1127/0077-7749/2010/0125~~

~~68. Araújo R, Castanhinha R, Martins R, Mateus O, Hendrickx C, Beckmann F et al. Filling the gaps of dinosaur eggshell phylogeny: Late Jurassic Theropod clutch with embryos from Portugal. Sci Rep. 2013; 3: 1924. https://doi.org/10.1038/srep01924~~

~~69. Brusatte SL, Benson RBJ, Hutt S. 2008. The osteology of *Neovenator salerii* (Dinosauria: Theropoda) from the Wealden group (Barremian) of the Isle of Wight. Monograph of the Palaeontographical Society. 2008;162(631):1–166.~~

Formatted: Font: Italic

~~70. Xu X. Avian Ancestors - A Review of the Phylogenetic Relationships of the Theropods Unenlagiidae, Microraptoria, *Anchiornis* and Scansoriopterygidae. Ameghiniana. 2013; 50(4), 469.~~

~~71. Currie PJ. Cranial anatomy of tyrannosaurid dinosaurs from the Late Cretaceous of Alberta, Canada.Acta Palaeontologica Polonica. 2003; 48 (2): 191–226~~

~~72. Brochu CR. Osteology of *Tyrannosaurus rex*: insights from a nearly complete skeleton and high-resolution computed tomographic analysis of the skull. Memoirs of the Society of Vertebrate Paleontology. 2003; 7: 1–138. doi: 10.2307/3889334.~~

~~73. Currie PJ. New information on the anatomy and relationships of *Dromaeosaurus albertensis* (Dinosauria: Theropoda) Journal of Vertebrate Paleontology. 1995; 15(3):576-591~~

~~74. Senter P. A new look at the phylogeny of Coelurosauria (Dinosauria: Theropoda.) Journal of Systematic Palaeontology. 2007, 5(4): 429-463~~

75. Choiniere JN, Clark JM, Forster CA, Xu X. A basal coelurosaur (Dinosauria: Theropoda) from the Late Jurassic (Oxfordian) of the Shishugou Formation in Wucaiwan, People's Republic of China, Journal of Vertebrate Paleontology. 2010; 30(6): 1773-1796, doi: 10.1080/02724634.2010.520779

76. Gianechini FA, Makovicky PJ, Apesteguía S. The teeth of the unenlagiine theropod *Buitreraptor* from the Cretaceous of Patagonia, Argentina, and the unusual dentition of the Gondwanan dromaeosaurids. Acta Palaeontologica Polonica. 2011; 56:279-290.

77. Xu X, Wang X-L, Wu X-C. A dromaeosaurid dinosaur with a filamentous integument from the Yixian Formation of China. Nature 1999; 401 (6750): 262–266. doi:10.1038/45769

78. Agnolín FL, Novas FE. Unenlagiid theropods: are they members of the Dromaeosauridae (Theropoda, Maniraptora)? Anais da Academia Brasileira de Ciências. 2011;83(1):117–162. doi: 10.1590/s0001-37652011000100008.

79. Rauhut OWM, Foth C, Tischlinger H. The oldest *Archaeopteryx* (Theropoda: Avialiae): a new specimen from the Kimmeridgian/Tithonian boundary of Schamhaupten, Bavaria. PeerJ 2018; 6: e4191. doi:10.7717/peerj.4191

80. Turner AH, Pol D, Clarke J, Erickson G, Norell M. A basal dromaeosaurid and size evolution preceding avian flight. Science. 2007; 317:1378–1381.

81. Currie PJ. 1987. Bird-like characteristics of the jaws and teeth of troodontid theropods (Dinosauria, Saurischia). Journal of Vertebrate Paleontology. 1987; 7: 72–81

82. Currie PJ, Rigby JK Jr, Sloan R. Theropod teeth from theJudith River Formation of southern Alberta, Canada; 1990; 107–125 in K. Carpenter and P. J. Currie (eds.), Dinosaur Systematics: Approaches and Perspectives. Cambridge University Press, New York.

83. Averianov AO, Sues H-D. A new troodontid (Dinosauria: Theropoda) from the Cenomanian of Uzbekistan, with a review of troodontid records from the

Formatted: Font: Italic

territories of the former Soviet Union. Journal of Vertebrate Paleontology. 2007; 27 (1): 87–98. doi:10.1671/0272-4634(2007)27[87:ANTDTF]2.0.CO;2

84. Lü J, Xu L, Liu Y, Zhang X, Jia S, Ji Q. A new troodontid theropod from the Late Cretaceous of central China, and the radiation of Asian troodontids. Acta Palaeontologica Polonica, 2010; 55:381-388.

85. Xu X, Li F. A new microraptorine specimen (Theropoda: Dromaeosauridae) with a brief comment on the evolution of compound bones in theropods. Vertebrata PalAsiatica, 2016; 54:269-285.

86. Larson DW. Diversity and variation of theropod dinosaur teeth from the uppermost Santonian Milk River Formation (Upper Cretaceous), Alberta: a quantitative method supporting identification of the oldest dinosaur tooth assemblage in Canada. Canadian Journal of Earth Sciences. 2008; 45:1455-1468. doi: 10.1139/e08-070

87. Currie PJ, Dong Z. 2001. New information on *Shanshanosaurus huoyanshanensis*, a juvenile tyrannosaurid (Theropoda, Dinosauria) from the Late

Cretaceous of China. Canadian Journal of Earth Sciences. 2991; 38:1729-1737. doi: 10.1139/e01-042

88. Sankey JT. Diversity of Latest Cretaceous (Late Maastrichtian) small theropods and birds: teeth from the Lance and Hell Creek Formations, USA. 2008; p. 117-134. In Sankey, JT, Baszio, S. (eds.), Vertebrate Microfossil Assemblages: Their Role in Paleoecology and Paleobiogeography. Indiana University Press, Bloomington, Indiana.

89. Fiorillo AR. On the occurrence of exceptionally large teeth of *Troodon* (Dinosauria: Saurischia) from the Late Cretaceous of northern Alaska. PALAIOS. 2008; 23:322–328.

90. Varricchio DJ, Horner JR, Jackson FD. Embryos and eggs for the Cretaceous theropod dinosaur *Troodon formosus*. Journal of Vertebrate Paleontology, 2002; 22:564-576. Doi: 10.1671/0272-4634(2002)022[0564:eaeftc]2.0.co;2

Formatted: Font: Italic

Formatted: Font: Italic

91. Cau A, Beyrand V, Voeten, D, Fernandez V, Tafforeau P, Stein K, et al. Synchrotron scanning reveals amphibious ecomorphology in a new clade of bird-like dinosaurs. Nature. 2017; 552 (7685): 395–399. doi: 10.1038/nature24679

92. Turner AH, Makovicky PJ, Norell M. A review of dromaeosaurid systematics and paravian phylogeny. Bulletin of the American Museum of Natural History. 2012; 371: 1–206. doi: 10.1206/748.1

93. Novas FE, Pol D, Canale JI, Porfiri JD, Calvo JO. A bizarre Cretaceous theropod dinosaur from Patagonia and the evolution of Gondwanan dromaeosaurids. Proceedings of the Royal Society B: Biological Sciences. 2009; 276:1101-1107. doi: 10.1098/rspb.2008.1554

94. Xu X, Norell MA. A new troodontid dinosaur from China with avian-like sleeping posture. Nature, 2004; 431:838-841. doi: 10.1038/nature02898

95. Makovicky PJ, Norell MA, Clark JM, Rowe T. Osteology and relationships of *Byronosaurus jaffei* (Theropoda: Troodontidae). American Museum Novitates. 2003; 3402:1-32. https://doi.org/10.1206/0003-0082(2003)402%3C0001:oarobj%3E2.0.co;2

96. Tsuihiji T, Barsbold R, Watabe M, Tsogtbaatar K, Chinzorig T, Fujiyama Y, Suzuki S. An exquisitely preserved troodontid theropod with new information on the palatal structure from the Upper Cretaceous of Mongolia. Naturwissenschaften. 2014; 101:131-142. doi: 10.1007/s00114-014-1143-9

97. Ji Q, Ji S, Lü J, You H, Chen W, Liu Y, Liu Y. First avialian bird from China. Geological Bulletin of China. 2005; 24:197-210.

98. Pei R, Li Q, Meng Q, Norell M, Gao K. 2017. New specimens of *Anchiornis huxleyi* (Theropoda, Paraves) from the late Jurassic of northeastern China. Bulletin of the American Museum of Natural History, 411:1–66. doi: 10.1206/0003-0090-411.1.1

99. Pei R, Norell MA, Barta DE, Bever GS, Pittman M, Xu X. Osteology of a new Late Cretaceous troodontid specimen from Ukhaa Tolgod, Ömnögovi Aimag, Mongolia. American Museum Novitates, 2017; 3889:1–47. doi: 10.1206/3889.1

100. Hu D, Hou L, Zhang L, Xu X. A pre- *Archaeopteryx* troodontid theropod from China with long feathers on the metatarsus. Nature, 2009; 461:640–643. doi: 10.1038/nature08322

101. Godefroit P, Demuynck H, Dyke G, Hu D, Escuillié F, Claeys P. Reduced plumage and flight ability of a new Jurassic paravian theropod from China. Nature Communications. 2013; 4:1394. doi: 10.1038/ncomms2389

102. Godefroit P, Cau A, Dong-Yu H, Escuillié F, Wenhao W, Dyke G. A Jurassic avialan dinosaur from China resolves the early phylogenetic history of birds. Nature. 2013; 498:359-362. doi: 10.1038/nature12168

103. Martin LD, Stewart JD, Whetstone KN. The origin of birds: structure of the tarsus and teeth. The Auk. 1980; 97:86-93.

104. Elzanowski A, Wellnhofer P. Cranial morphology of *Archaeopteryx*: evidence from the seventh skeleton. Journal of Vertebrate Paleontology. 1996; 16: 81-94. doi: 10.1080/02724634.1996.10011286

Formatted: Font: Italic

Formatted: Font: Italic

105. Mayr G, Pohl B, Hartman S, Peters DS. The tenth skeletal specimen of *Archaeopteryx*. Zoological Journal of the Linnean Society. 2007; 149:97-116. doi: 10.1111/j.1096-3642.2006.00245.x

106. Rauhut OWM, Werner C. First record of the family Dromaeosauridae (Dinosauria: Theropoda) in the Cretaceous of Gondwana (Wadi Milk Formation, northern Sudan). Paläontologische Zeitschrift. 1995; 69:475-489. doi: 10.1007/BF02987808

107. Sweetman SC. 2004. The first record of velociraptorine dinosaurs (Saurischia, Theropoda) from the Wealden (Early Cretaceous, Barremian) of southern England. Cretaceous Research, 25:353–364. doi: 10.1016/j.cretres.2004.01.004

108. Vullo R, Néraudeau, D, Lenglet T. Dinosaur teeth from the Cenomanian of Charentes, Western France: evidence for a mixed Laurasian-Gondwanan assemblage. Journal of Vertebrate Paleontology. 2007; 27:931-943. doi10.1671/0272-4634(2007)27[931:dtftco]2.0.co;2

109. van der Lubbe T, Richter U, Knötschke N. Velociraptorine dromaeosaurid teeth from the Kimmeridgian (Late Jurassic) of Germany. Acta Palaeontologica Polonica. 2009; 54:401-408. doi: 10.4202/app.2008.0007

110. Norell MA, Clark JM, Turner AH, Makovicky PJ, Barsbold R, Rowe T. A new dromaeosaurid theropod from Ukhaa Tolgod (Ömnögov, Mongolia). American Museum Novitates. 2006; 3545:1-51. doi: 10.1206/0003-0082(2006)3545[1:andtfu]2.0.co;2

111. Xu X, Tan Q, Sullivan C, Han F, Xiao D. 2011. A short-armed troodontid dinosaur from the Upper Cretaceous of Inner Mongolia and its implications for troodontid evolution. PLoS ONE. 2011; 6:e22916. https://doi.org/10.1371/journal.pone.0022916

112. Norell MA, Makovicky PJ, Bever GS, Balanoff AM, Clark JM, Barsbold R, Rowe T. A review of the Mongolian Cretaceous dinosaur *Saurornithoides* (Troodontidae: Theropoda). American Museum Novitates. 2009; 3654:1-63. https://doi.org/10.1206/648.1

Formatted: Font: Italic

113. Longrich N. Small theropod teeth from the Lance Formation of Wyoming, USA. 2008; 135-158. In Sankey, J.T. and Baszio, S. (eds.), Vertebrate Microfossil Assemblages: Their Role in Paleoecology and Paleobiogeography. Indiana University Press, Bloomington, Indiana.

114. Holtz TRJ, Brinkman DL, Chandler CL. Denticle morphometrics and a possibly omnivorous feeding habit for the theropod dinosaur *Troodon*. Gaia, 1998; 15:159-166.

115. Vullo R, Néraudeau D. Additional dinosaur teeth from the Cenomanian (Late Cretaceous) of Charentes, southwestern France. Comptes Rendus Palevol. 2010; 9:121-126. doi: 10.1016/j.crpv.2010.03.001

116. Hu D, Clarke JA, Eliason CM, Qiu R, Li Q, Shawkey MD, et al. A bony-crested Jurassic dinosaur with evidence of iridescent plumage highlights complexity in early paravian evolution. Nature Communications. 2018; 9:217. doi: 10.1038/s41467-017-02515-y

117. Currie PJ, Varricchio DJ. A new dromaeosaurid from the Horseshoe Canyon Formation (Upper Cretaceous) of Alberta, Canada. 2004; 112–132. In

Currie PJ, Koppelhus EB, Shugar MA and Wright JL. (eds.) Feathered Dragons. Indianapolis: Indiana University Press.

118. Sankey JT. 2001. Late Campanian southern dinosaurs, Aguja Formation, Big Bend, Texas. Journal of Paleontology, 75:208-215. doi: 10.1017/s0022336000031991

119. Burnham DA. New Information on *Bambiraptor feinbergi* (Theropoda: Dromaeosauridae) from the Late Cretaceous of Montana. 2004; 67-111. In Currie PJ, Koppelhus EB, Shugar MA and Wright JL. (eds.) Feathered Dragons. Indianapolis: Indiana University Press.

120. Xu X, Pittman MD, Sullivan C, Choiniere JN, Tan QW, Clark JM, Norell MA, Wang S. The taxonomic status of the Late Cretaceous dromaeosaurid *Linheraptor exquisitus* and its implications for dromaeosaurid systematics. Vertebrata PalAsiatica. 2015; 53:29-62.

121. Norell MA, Clark JM, Turner AH, Makovicky PJ, Barsbold R, Rowe T. A new dromaeosaurid theropod from Ukhaa Tolgod (Omnogov, Mongolia). American Museum Novitates. 2006; 3545: 1-51.

Formatted: Font: Italic

Formatted: Font: Italic

122. Kirkland JI, Gaston R, Burge D. A large dromaeosaur (Theropoda) from the Lower Cretaceous of eastern Utah. Hunteria. 1993; 2:1–16.

123. Shen C, Lü J, Liu S, Kundrát M, Brusatte SL, Gao H. A new troodontid dinosaur from the Lower Cretaceous Yixian Formation of Liaoning Province, China. Acta Geologica Sinica-English Edition. 2017; 91:763-780. https://doi.org/10.1111/1755-6724.13307

124. Carpenter K. Baby dinosaurs from the Late Cretaceous Lance and Hell Creek formations and a description of a new species of theropod, in Contributions to Geology, University of Wyoming. 1982; 20(2): 123–134

125. Currie PJ, Dong Z. New information on Cretaceous troodontids (Dinosauria, Theropoda) from the People's Republic of China. Canadian Journal of Earth Sciences. 2001; 38:1753–1766. doi: 10.1139/e01-065

126. Xu X, Wang X-L. A New Dromaeosaur (Dinosauria: Theropoda) from the Early Cretaceous Yixian Formation of Western Liaoning. Vertebrata PalAsiatica. 2004; 42(2): 11–119.

~~127. Leidy J. Notice of remains of extinct reptiles and fishes, discovered by Dr. Hayden FV in the Bad Lands of the Judith River, Nebraska Territory. PNAS. 1856; 8:72-73.~~

~~128. Russell LS. The dentary of *Troödon*, a genus of theropod dinosaurs. Journal of Paleontology. 1948; 22:625-629.~~

~~129. Baszio S. Systematic palaeontology of isolated dinosaur teeth from the latest Cretaceous of south Alberta, Canada. Courier Forschungsinstitut Senckenberg. 1997; 196:33-77.~~

~~130. Smith JB, Vann DR, Dodson P. 2005. Dental morphology and variation in theropod dinosaurs: Implications for the taxonomic identification of isolated teeth. The Anatomical Record Part A: Discoveries in Molecular, Cellular, and Evolutionary Biology, 285A:699-736. doi: 10.1002/ar.a.20206~~

~~131. Zanno LE. A taxonomic and phylogenetic re-evaluation of Therizinosauria (Dinosauria: Maniraptora). Journal of Systematic Palaeontology. 2010; 8:503-543. doi: 10.1080/14772019.2010.488045~~

132. Rauhut OWM, Foth C, Tischlinger H, Norell MA. Exceptionally preserved juvenile megalosauroid theropod dinosaur with filamentous integument from the Late Jurassic of Germany. Proceedings of the National Academy of Sciences. 2012; 109:11746-11751. doi: 10.1073/pnas.1203238109

133. Choiniere JN, Clark JM, Forster CA, Norell MA, Eberth DA, Erickson GM, et al. A juvenile specimen of a new coelurosaur (Dinosauria: Theropoda) from the Middle-Late Jurassic Shishugou Formation of Xinjiang, People's Republic of China. Journal of Systematic Palaeontology. 2014; 12:177-215. doi: 10.1080/14772019.2013.781067

134. Tsuihiji T, Watabe M, Tsogtbaatar K, Tsubamoto T, Barsbold R, Suzuki S et al. Cranial osteology of a juvenile specimen of *Tarbosaurus bataar* (Theropoda, Tyrannosauridae) from the Nemegt Formation (Upper Cretaceous) of Bugin Tsav, Mongolia. Journal of Vertebrate Paleontology. 2011; 31:497-517. https://doi.org/10.1080/02724634.2011.557116

135. Carr TD, Williamson TE. Diversity of late Maastrichtian Tyrannosauridae (Dinosauria: Theropoda) from western North America.

~~Zoological Journal of the Linnean Society. 2004; 142:479-523. doi: 10.1111/j.1096-3642.2004.00130.x~~

~~136. Han G, Chiappe LM, Ji S-A, Habib M, Turner AH, Chinsamy A et al. 2014. A new raptorial dinosaur with exceptionally long feathering provides insights into dromaeosaurid flight performance. Nature Communications, 5:1-9. doi: 10.1038/ncomms5382~~

~~137. Xu X, Wu X-C. Cranial morphology of *Sinornithosaurus millenii* Xu et al. 1999 (Dinosauria: Theropoda: Dromaeosauridae) from the Yixian Formation of Liaoning, China. Canadian Journal of Earth Sciences. 2001; 38:1739-1752. doi: 10.1139/e01-082~~

~~138. Zheng X, Xu X, You H, Zhao Q, Dong Z. A short-armed dromaeosaurid from the Jehol Group of China with implications for early dromaeosaurid evolution. Proceedings of the Royal Society B: Biological Sciences. 2010; 277:211-217. doi: 10.1098/rspb.2009.1178.~~

~~139. Perle A, Norell M, Clark JM. A new maniraptoran theropod, *Achillobator giganticus* (Dromaeosauridae), from the Upper Cretaceous of~~

Burkhant, Mongolia. Contributions from the Geology and Mineralogy Chair, National Museum of Mongolia. 1999; 101:1-105.

140. DePalma RA, Burnham DA, Martin LD, Larson PL, Bakker RT. The first giant raptor (Theropoda: Dromaeosauridae) from the Hell Creek Formation. Paleontological Contributions, 2015; 1-16. doi: 10.17161/paleo.1808.18764

141. Ostrom JH. Osteology of *Deinonychus antirrhopus*, an unusual theropod from the Lower Cretaceous of Montana. Bulletin Peabody Museum of Natural History: Peabody Museum of Natural History, Yale University. 1969; 30:1-165.

142. Barsbold R, Osmólska H. The skull of *Velociraptor* (Theropoda) from the Late Cretaceous of Mongolia. Acta Palaeontologica Polonica. 1999; 44:189–219.

143. Milner AC. Theropod dinosaurs of the Purbeck limestone group, Southern England. Special Papers in Palaeontology. 2002; 68:191-202.

144. Allain R, Taquet P. A new genus of Dromaeosauridae (Dinosauria, Theropoda) from the Upper Cretaceous of France. Journal of Vertebrate Paleontology. 2000; 20:404-407. doi: 10.1671/0272-4634(2000)020[0404:angodd]2.0.co;2

145. Barsbold R. Saurornithoididae, a new family of small theropod dinosaurs from central Asia and North America. Palaeontologia Polonica, 1974; 30:5–22.

146. Samman T, Powell GL, Currie PJ, Hills LV. Morphometry of the teeth of western North American tyrannosaurids and its applicability to quantitative classification. Acta Palaeontologica Polonica. 2005; 50: 757–776.

147. Buckley LG, Larson DW, Reichel M, Samman T. Quantifying tooth variation within a single population of *Albertosaurus sarcophagus* (Theropoda: Tyrannosauridae) and implications for identifying isolated teeth of tyrannosaurids. Canadian Journal of Earth Science. 2010; 47: 1227–1251.

148. Russell DA. The environments of Canadian dinosaurs. Canadian Geographical Journal. 1973; 87: 4–11.

149. Paul GS. Migration. In The Encyclopedia of Dinosaurs, Edited by: Currie P, Padian K. 1997; 444–446. San Diego, CA: Academic Press

150. Bell PR, Snively E. 2008. Polar dinosaurs on parade: a review of dinosaur migration, Alcheringa: An Australasian Journal of Palaeontology, 2008; 32(3): 271–284, doi: 10.1080/03115510802096101

151. Gangloff RA, Fiorillo AR. Taphonomy and paleoecology of a bonebed from the Prince Creek Formation, North Slope, Alaska. Palaios. 2010; 25: 299–317.

152. Suarez CA, Ludvigson GA, Gonzalez LA, Fiorillo AR, Flaig PP, McCarthy PJ. 2013. Use of multiple isotope proxies for elucidating Arctic Cretaceous paleohydrology. In Bojar AV, Melinte-Dobrinescu MC, Smit J. (eds.). Isotopic Studies in Cretaceous Research: Geological Society of London Special Publication. 2013; 382:185–202.

153. Avgar T, Street G, Fryxell JM, On the adaptive benefits of mammal migration. Canadian Journal of Zoology. 2014; 92(6): 481-490.

154. Guillet A. Distribution and conservation of the shoebill (*Balaeniceps rex*) in the southern Sudan. Biological Conservation. 1978; 13: 39-49.

155. Curry-Lindahl K. Bird Migration in Africa: Movements between Six Continents. 1981; 2. New York: Academic Press

156. Fiorillo AR. The dinosaurs of arctic Alaska. Sci Am. 2004; 291: 84–91.

157. Horner JR, Makela R. Nest of juveniles provides evidence of family structure among dinosaurs. Nature. 1979; 282(5736):296-298.

158. Horner JR. Evidence of colonial nesting and 'site fidelity' among ornithischian dinosaurs. Nature. 1982; 297(5868):675-676.

159. Norell MA, Clark JM, Chiappe LM, Dashzeveg D. A nesting dinosaur. Nature. 1995; 378:774-776.

160. Tanaka K, Zelenitsky DK, Therrien F, Kobayashi Y. Nest substrate reflects incubation style in extant archosaurs with implications for dinosaur nesting habits. Sci Rep. 2018; 8, 3170. doi: 10.1038/s41598-018-21386-x

161. Varricchio DJ. Reproduction and Parenting in Paul, G.S. (ed.). The Scientific American Book of Dinosaurs. New York: St. Martin's Press. 2000; 279-293.

162. Mateo JA, Lopez- Jurado LF. Dental ontogeny in *Lacerta lepida* (Sauria, Lacertidae) and its relationship to diet. Copeia 1997; 461–463.

163. Townsend V, Akin J, Felgenhauer B, Dauphine J, Kidder S. Dentition of the Ground Skink, *Scincella lateralis* (Sauria, Scincidae). Copeia, 1999; (3): 783–788. doi:10.2307/1447615

164. Fiorillo AR, Currie PJ. Theropod teeth from the Judith River Formation (Upper Cretaceous) of south-central Montana. Journal of Vertebrate Paleontology. 1994; 14 (1): 74–80. doi: 10.1080/02724634.1994.10011539

165. Fowler DW, Freedman EA, Scannella JB, Kambic RE. The Predatory ecology of *Deinonychus* and the origin of flapping in birds. PLoS ONE. 2011; 6(12): e28964. doi: 10.1371/journal.pone.0028964

166. Torices A, Wilkinson R, Arbour VM, Ruiz-Omeñaca JI, Currie PJ. Puncture-and-pull biomechanics in the teeth of predatory coelurosaurian dinosaurs. Current Biology. 2018; 28:1467-1474.e2. doi: 10.1016/j.cub.2018.03.042

Formatted: Level 1, Indent: First line: 0.5", Space After: 0 pt, Line spacing: Double

Figure captions

Figure 1. Locality map (A) of Pediomys point (red star) in the North Slope of Alaska, USA. Coordinates: N 70.018667°, W 151.591488°. Stratigraphic sections schematised in Fig 2 are reported here in B, with PdP representing the fossil bone-bearing section.

Figure 2. Geological setting. Stratigraphic sections at Pediomys point. Black star represents DMNH 21183.

Figure 3. DMNH 21183. Anterior portion of a dromaeosaurid dentary in lateral (A), medial (B), dorsal view (C) views and close up of the interdental plates and 3rd tooth in medial view (D). Curved arrows represent features hidden on that view. Abbreviations: a2, 2nd alveolus; a3. 3rd alveolus; d2, 2nd dentary tooth; d3, 3rd dentary tooth; d4, 4th dentary tooth; ave, antero-ventral process; idp, interdental plate; lr, lateral ridge; mg, Meckelian groove; mf, Meckelian ~~foramen~~foramina. Scale bar: 2 mm.

Figure 4. Closeup ~~of~~ on the dentary teeth and lateral ridge ~~morphological details~~ in DMNH 21183. Detail of the 2nd dentary tooth

highlighting the distal carina under normal light (A) and fluorescent microscopy (B). Details of the distal denticles (C) and ~~closeup~~close-up of the lateral ridge (lr) close to the alveolar margin in lateral views (D). Abbreviation: lr, lateral ridge. Scale bar: 0.5 mm.

Figure 5. Phylogenetic position of DMNH 21183~~ in Lee et al. [33]~~. Strict consensus topology of the shortest trees recovered by the parsimony analyses of the phylogenetic dataset ~~by~~of Lee et al. [33] with the addition of DMNH 21183 (384 MPTs, 6043 steps, CI= 0.244, RI= 0.587). The main clades of Theropoda outside Deinonychosauria are ~~collapesed~~collapsed for space constraints. Full topology available in S4 Fig. Numbers adjacent to nodes are the bootstrap values. Red box highlights the node containing DMNH 21183 (red arrow).

Formatted: Not Highlight

Formatted: Not Highlight

Figure 6. Phylogenetic position of DMNH 21183 in Hendrickx et al. [35]. Strict consensus topology of the shortest trees recovered by the parsimony analyses showing the position of DMNH 21183 in (A) the dentition-only character matrix from Hendrickx et al. [35] (2 MPTs, 1314 steps, CI= 0.194, RI= 0.418)~~ (A)~~, and ~~in~~ (B) the tooth-crown-only character matrix from Hendrickx et al. [35] (5 MPTs, 867 steps, CI = 0.183, RI = 0.439~~B~~). The overall topology was constrained in both analyses, with DMNH 21183 allowed to float. The main clades of Theropoda

Formatted: Not Highlight

Formatted: Not Highlight

Formatted: Not Highlight

outside Deinonychosauria are collapesed for space constraints. Full topology available in S5-S6 Figs. Numbers adjacent to nodes are the bootstrap values. Red box highlights the node containing DMNH 21183 (red arrow) in Eudromaeosauria.

Figure 7. Position of DMNH 21183 in the theropod teeth morphospace. Principal components analysis of theropod teeth morphospace based on the dataset from Gerke and Wings [39]. DMNH 21183 is marked by a yellow star. Abbreviations: crown height (CH), crown basal width (CBW), ratio between mesial (anterior) and distal (posterior) denticles (DSDI). Morphometric dataset prepared as explained in methods and including measurements from DMNH 21183 reported in Supplementary Information (S2 Dataset).

Formatted: Not Highlight

~~**Figure 5. Denticle size index comparison.** Box plot showing a comparison of the relative denticle size index (DSDI) between DMNH 21183 and four clades of deinonychosaurian theropods. DMNH 21183 is marked as a star. Dots represent outlier of each clade's distribution.~~

Figure ~~6~~8. Position of DMNH 21183 in the ~~theropod~~ paravian teeth morphospace. Principal components analysis of theropod teeth morphospace based on the dataset from Larson and Currie [30]. DMNH 21183 is marked ~~as~~ by a

blue star. Abbreviations: fore-aft basal length (FABL), crown height (CH), basal width (BW), anterior (ADM) and posterior denticles per ~~millimetre~~millimeter (PDM). Morphometric dataset prepared as explained in methods and including measurements from DMNH 21183 reported in Supplementary Information (Dataset ~~S1~~S2).

Figure 9. Denticle size index comparison between deinonychosaurian taxa. Box plot showing a comparison of the relative denticle size index (DSDI) between DMNH 21183 and four clades of deinonychosaurian theropods from the dataset in Larson and Currie [30]. DMNH 21183 is marked as a star. Dots represent outlier of each clade's distribution.

Figure 10. Life reconstruction of the Alaskan ~~S~~saurornitholestin~~a~~e in its environment. Artistic restoration by scientific illustrator Andrey Atuchin ~~of~~depicts a riparian ~~paleoenvironment~~setting ~~from~~in the Prince Creek Formation, ~~as described in~~matching the ~~G~~geological ~~setting~~evidence ~~section~~described ~~of~~in this paper. DMNH 21183 ~~is~~comes from the juvenile dromaeosaurid ~~in~~on the branch close to ~~an~~the adult, while a subadult (foreground) ~~preys on~~stalks an individual of *Unnuakomys hutchisoni* [67], a methatherian known ~~taxon~~ from this

Formatted: Font: Not Bold

Formatted: Font: Italic

Formatted: Font: Not Italic

Formatted: Font: Not Italic

Formatted: Font: Not Italic

~~formation~~locality. Individuals of the sympatric ceratopsid *Pachyrhinosaurus perotorum* [22] rest in the background.

Table 1. Measurements of DMNH 21183.

Table 2. Coefficients of the PCA analysis run on the theropod teeth dataset published by ~~Larson~~ Gerke and ~~Currie~~ Wings [39~~30~~].

Table 3. Coefficients of the PCA analysis run on the deinonychosaurian teeth dataset published by Larson and Currie [30].

Supporting information

S1 Fig. Magnified close-up views of the 2nd tooth in DMNH 21183. Close-up of the mesial carina in anterior view (A, C), highlighting the ~~the~~ denticle-bearing anterior carina (ac). Magnified lingual view of the tooth (B) highlighting the anterior (ac) and posterior (pc) carinae (D). Dotted line (C) highlights the interdenticular sulci. Scale bar: 100 µm.

S2 Fig. Discriminant Functional Analysis of DMNH 21183 in Gerke and Wings [39]. Discriminant Functional Analysis of DMNH 21183 in the theropod teeth morphospace generated with the morphometric dataset provided in Gerke and

Formatted: Font: Italic

Formatted: Highlight

Formatted: Font: Bold

Formatted: Font: Not Bold

Wings [39]. Abbreviationds: LD, linear dimension. DMNH 21183 indicated by a green star.

S3 Fig. Discriminant Functional Analysis of DMNH 21183 in Larson and Currie [40]. Discriminant Functional Analysis of DMNH 21183 in the deinonychosaurian teeth morphospace generated with the morphometric dataset provided in Larson and Currie [40]. Abbreviationds: LD, linear dimension. DMNH 21183 indicated by a pink star.

S4 Fig. Phylogenetic position of DMNH 21183 in Lee et al. [33]. Strict consensus topology of the shortest trees recovered by the parsimony analyses showing the position of DMNH 21183 in the matrix from Lee et al. [33] (384 MPTs, 6043 steps, CI= 0.244, RI= 0.587). Numbers adjacent to nodes are the bootstrap values. Red box highlights the node containing DMNH 21183 (red arrow).

S5 Fig. Phylogenetic position of DMNH 21183 in Hendrickx et al. [35] Strict consensus topology of the shortest trees recovered by the parsimony analyses showing the position of DMNH 21183 in the dentition-only character matrix from Hendrickx et al. [35] (2 MPTs, 1314 steps, CI= 0.194, RI= 0.418). The overall

Formatted: Font: Not Bold

Formatted: Font: Not Bold

Formatted: Not Highlight

Formatted: Not Highlight

topology was constrained with DMNH 21183 allowed to float. Numbers adjacent to nodes are the bootstrap values. Red box highlights the node containing DMNH 21183 (red arrow) in Eudromaeosauria.

S6 Fig. Phylogenetic position of DMNH 21183 in Hendrickx et al. [35] Strict consensus topology of the shortest trees recovered by the parsimony analyses showing the position of DMNH 21183 in the tooth-crown-only character matrix from Hendrickx et al. [35] (5 MPTs, 867 steps, CI = 0.183, RI = 0.439). The overall topology was constrained with DMNH 21183 allowed to float. Numbers adjacent to nodes are the bootstrap values. Red box highlights the more inclusive node containing DMNH 21183 (red arrow) in Eudromaeosauria.

S1 Table. Systematics used in this study. Systematics names and relative phylogenetic definition used in this study.

S1 Dataset. Phylogenetic character scoring of DMNH 21183. Character scoring in the phylogenetic matrices from Lee et al. [33] and Hendrickx et al. [35].

Formatted: Not Highlight

Formatted: Font: Bold

Formatted: Font: Bold

Formatted: Font: Not Bold

Formatted: Font: Not Bold

Formatted: Font: Not Bold

Formatted: Font: Not Bold

S2 Dataset. Morphometric data. Morphometric scoring for DMNH 21183 and modified datasets for multivariate analyses (PCA and DFA) in Gerke and Wings [39] and Larson and Currie [40].

S3 Dataset. Latest Cretaceous dromaeosaurids in North America. Faunal list compilation of all dromaeosaurid taxa in the latest Cretaceous (84.5–66.043 million years ago) with geographic, stratigraphic, chronological, and literature information attached.

Formatted: Font: Not Bold

Formatted: Font: Not Bold

Formatted: Font: Not Bold

Formatted: Font: Bold

Formatted: Font: Bold

Formatted: Font: Not Bold

Formatted: Font: Not Bold

Formatted: Font: Bold

5. Review Comments to the Author

Please use the space provided to explain your answers to the questions above. You may also include additional comments for the author, including concerns about dual publication, research ethics, or publication ethics. (Please upload your review as an attachment if it exceeds 20,000 characters)

Reviewer #1: *In their manuscript entitled "The first juvenile dromaeosaurid (Dinosauria: Theropoda) from Arctic Alaska", Chiarenza and colleagues report, describe and identify the anterior portion of a dentary (DMNH 21183) from the Early Maastrichtian Prince Creek Formation of northern Alaska. The specimen is referred to a juvenile saurornitholestine dromaeosaurid based on comparative anatomy and a morphometric analysis using the best preserved tooth (d3), an identification I agree with. This paper is well-written, well-organized and most of the figures are very nice. The descriptive part is thorough and the introduction, geological settings and discussion section are sound and detailed. The methodology used to identify the material could certainly be improved though, and I have some problems with the way the text discussing the phylogenetic distribution of the material based on dental features is written. If I believe that this contribution only provides a limited amount of information for our knowledge on theropods/paravian paleogeography and palaeoecology, I would nonetheless suggest to accept Chiarenza et al.'s work with moderate revisions, urging the authors to take into consideration my main recommendations:*

- *My main problem with this paper lies in the author's method to identify the material. First, I wonder why they did not perform a cladistic analysis using the most recent datamatrix on coelurosaurs/paravian relationships. I understand that their material is very limited but wonder if it does not display enough apomorphic features to help resolve its phylogenetic affinity. At least, the authors should specify why they do not want to conduct a cladistic analysis to explore the phylogenetic distribution of DMNH 21183. Second, they decide to perform a PCA based on crown-measurements of d3 to help in the identification of the material. Yet, I believe there are more appropriate methods to identify theropod teeth such as discriminant and cluster analyses using a dataset of crown-based measurements, or (even better according to me) a cladistic analysis using a dentition-based datamatrix and a fully constrained topological tree (see my paper on the Aerosteon tooth and the dentition of Abelisauridae recently published in Cretaceous Research on how to perform those analyses). That being said, the major problem I see is that the best preserved tooth of this small portion of mandible, d3, is a partially erupted tooth and I wonder how the authors managed to accurately take measurements of FABL, CH and BW on this crown. In addition, it does not make much sense to me to use a dataset restricted to paravians and to omit specimens from which the mesial carina does not include denticles. I would therefore recommend the authors to perform a first analysis with all taxa and measurements from Larson & Currie's dataset, then use a more restrictive one based on relevant arguments on why tyrannosauroids, for instance, should be excluded. I would also strongly advise them to perform discriminant and cluster analyses on the dataset of crown-based measurements and, ideally, a cladistic analysis using my most recent dentition-based datamatrix (from the Aerosteon paper), coding their dental material as coming from the dentary and the mesial dentition. I am quite confident that the result of the cladistic analysis would further*

support a saurornitholestine affinity.

We appreciate the positive comments from the Reviewer. We decided to largely follow his recommendation in proceeding with a set of phylogenetic tests before a quantitative-morphometric analysis of the material. In this resubmission we first included DMNH 21183 in the phylogenetic dataset from Lee et al. (2014), a dataset with a wide taxonomic sampling but also with a large pool of dentary characters to maximize the scorings for our fragmentary specimen. We then included the dental coding from the specimen in the dentition-only and crown-tooth-only data matrices from Hendrickx et al. (2020), an additional set of analyses which strengthen our previous interpretation based on comparative anatomy and morphometrics. We also embraced the recommendations related to morphometric by first performing multivariate analyses on the teeth morphometric dataset by Gerke and Wings (2016) and then moving on the less inclusive Larson and Currie (2016). We want to point out that the choice of Gerke and Wings (2016) was based on the need for a purely morphometric (that is quantitative) dataset to compare our specimen, as we wanted to keep consistency with the measurements from Larson and Currie (2013) but also wanted to have an independent test which could exclude any qualitative observations (making it fully independent from the comparative and phylogenetic results). We strongly acknowledge the reviewer in his suggestions, as these additional analyses not only strengthened our interpretation, but also provided scope for expanded discussion on this specimen.

- In the methodology section, the authors should specify the result(s) of the cladistic analysis of what publications they follow when commenting on paravian phylogeny and the phylogenetic position of the different dromaeosaurids. Likewise, they should add the fact that it is the third dentary tooth of DMNH 21183 that is included in the PCA.

We thank the Reviewer for this suggestion, this is now been included in the Material and Methods section.

- In the chapter on morphological remarks (which I would suggest to rename "Phylogenetic position of DMNH 21183"), in the section in which the authors discuss the distribution of dental features displayed by DMNH 21183, they obviously use my recent paper on the distribution of dental features in theropods (Palaeontologica Electronica) as a main reference. I thank them for that but, if my paper is often cited, it frustrates me to see that some parts of the text directly comes from my paper without citing it. In many parts of the text, the authors just copied and pasted portions of my PE publication, using the exact same citations and specimen numbers, while I highly doubt that they have personally examined the material I have, or looked at the references the way I have. For fairness (and because sometimes it almost looks like plagiarism), I would really like the authors to rephrase the text that directly comes from my paper using the later as the main bibliographical source, and remove all specimens (those coming directly from my paper) that they did not examine themselves. In this section of the MS, the DSDI for crowns having mesial and distal roughly of the same size is also close to 1 and not lower than 0.9.

We thank the reviewer for these suggestions: we proceeded in renaming the section, paraphrasing further the content referring to his work, adding additional citations to them and reporting all of his edits (e.g. related to the DSDI of some taxa). We hope that this resubmission fully reflects the credit due to his scientific production.

- I don't have any comments to make regarding the Introduction, Geological setting and Depositional Environments, and Discussion sections, which really seem to be sound and well-written. I would only recommend to move the part of the text that discuss the juvenile features displayed by the specimen, present in the description section, to the Results or Discussion sections (I always prefer discussing the affinities of a specimen and its ontogenetic stage in the Discussion section, personally).

Thanks for the appreciative comments. We also agreed with the recommendation regarding the rearrangement of the text and proceeded doing so in this resubmission.

- Regarding figures, I would only suggest the authors to make the convex hull and the arrows of the variables used in the PCA better visible to the reader, in figure 6. Many taxa must also be italicized, while the capital letters of some titles of articles should be removed, in the bibliography section.

Thanks for these observations, we modified these figures and followed the same for the new ones added to this resubmission.

I provided additional corrections, remarks and suggestion in the pdf version of their MS, and ask the authors to take them into consideration.

We thank the reviewer for these thorough edits. We accepted and modified all relevant portions of the main manuscript text accordingly.

Kind regards,

Christophe Hendrickx, San Miguel de Tucumán, the 14th of March 2020.

Reviewer #2: March 30, 2020

Dear Editor,

I submit to you my review of PONE-D-20-05495, "The first juvenile dromaeosaurid (Dinosauria: Theropoda) from Arctic Alaska" by Chiarenza et al. The authors report on the first skeletal material of a dromaeosaurid from the Arctic, which is an important data point; fortunately, the specimen, although fragmentary, has some important features that enables comparisons with other taxa. I think that the article is publishable with minor revisions. Most of the fixes center on writing style.

In terms of major concerns, I find the second paragraph of the Discussion to be somewhat convoluted and difficult to follow; I think it can be redrafted into a more straightforward style so that the main points aren't lost to the reader.

The third paragraph makes the claim that the Arctic saurornitholestines were nonmigratory; in my view, the evidence is mute on that point and the authors should present a more critical assessment of such claims; for example, what evidence is required to support the claim? Is that evidence available based on the fossil at hand, really?

Finally, the fourth paragraph continues the argument, but from what strikes me as – at best - tangential lines of evidence. Again, I encourage the authors to re-visit this section with a critical bent of mind. Other comments on the discussion appear in the specific comments below.

Unless I missed it, there is one significant gap in the discussion, and that is comparison with the dromaeosaurid fauna of the Horseshoe Canyon Formation of Alberta, which is equivalent in geological age to the PCF. I suggest that the authors make an explicit comparison with the HCF dromaeosaurids to arrive at a snapshot of the distribution of the clade at that time in the northern half of Laurasia. I think that is the opportunity that will lock in future citations of the article, once it is published.

Finally, the authors identify a feature (lateral ridge of the dentary) in the new fossil that is also only seen elsewhere in the Gondwanan Buitreraptor, but later in the ms, they consider the fossil referable to Saurornitholestinae based on a morphometric analysis of teeth. I ask that the authors acknowledge these conflicting hypotheses of identity and to explicitly provide their rationale for preferring one hypothesis over the other, if that is what they decide, in the end, to do.

My statistical experience does not extend to PCA analyses, so I am unable to comment on that part of their work.

The authors may know my identity: Thomas D. Carr

We would thank the Reviewer for his suggestions. We proceeded in this resubmission with a double check in writing style and grammar. Given also the recommendations from Reviewer 1, we decided to expand the study with additional phylogenetic and morphometric analyses. As a consequence, the Discussion section has been now partially reworded and reorganized, hoping to make its style more readable by the audience. We appreciate the suggestion to expand our discussion on paleoecology and biogeography of other contemporary dromaeosaurids. We did so not only by expanding the relevant discussion paragraphs, but also reporting some additional data (Dataset S3) to compare more easily the temporal and geographic distribution of North American dromaeosaurids during the latest Cretaceous. Regarding the anatomical observations, after we expanded our search because of the suggestions from Reviewer 1, we found that the structure we previously considered a potential synapomorphy between *Buitreraptor* and DMNH 21183 as to be more widespread in Dromaeosauridae. In particular we noticed the presence of similar lateral ridges in many other dentary rims of dromaeosaurids (e.g. *Velociraptor*

AMNH 6515 right side, *Tsaagan* IGM 100/1015 right side) which are partially emphasized by damaging of the bone surface. We also want to remark that the absence of denticulation in Unenlaginae (as reported in the former draft of the paper) already excludes the identification of DMNH 21183 as a closer taxon to, for example *Buitreraptor*, than to more derived dromaeosaurids (e.g. Eudromaeosauria). We then modified the text accordingly to reflect these observations. We also added more osteological information that popped up while scoring the specimen for phylogenetic analyses, strengthening our previous interpretation based on comparative anatomy. We are also sure that the reviewer will appreciate the efforts in providing a suite of phylogenetic tests to our hypotheses, supporting our previous interpretation related to the systematic position of this Alaskan theropod.

Lastly we expanded the section related to the paleoecology of Arctic dromaeosaurs which the reviewer previously considered speculative. We provided more context based particularly on comparison with modern theropods' migratory habits in the Arctic region. We also added information from the literature on growth rates and other indicative physiological observations that would back up our interpretation that these Arctic theropods indeed would spend their full solar year in the Arctic without migrating to southern latitudes. We removed, as the Reviewer recommended, some more speculative ecomorphological content, in order to simplify and focus further our Discussion section. We furthermore appreciated the effort in proof-reading our manuscript, and provided below a point by point check of the edits added to this resubmission.

Specific comments:
Line 17: delete "the"; taxonomic names are formal names and so are not preceded by "the."

Done

Line 19: replace "diminutive" with "small"; avoid jargon.

Done.

Line 20: delete "morphological", replace "based on" with "with."

Done

Line 23: replace "status" with "growth stage"; as far as I can tell relative maturity isn't a status. Replace "exploration" with "comparison."

Done

Line 62: Replace "taxonomical" with "taxonomic."

Done

Line 65: delete "purported", unless you are doubtful of its uniqueness.

Done

Line 95: replace "like" with "such as."

Done

Line 104: replace "examined" with "studied first hand" and modify the sentence accordingly.

Edit done and sentence rephrased to: "The subclades of theropods included in this dataset, apart from specimen studied first hand (DMNH 21183) are:"

Line 106: replace "performed" with "did."

Done

Line 107: insert a comma after "measurements"; find an accurate replacement for "distilling".

Replaced with 'converting"

Line 108: replace "representing" with "that represents"; delete "to be."

Done

Line 110: replace "estimate…of" with "compare"; replace "within" with "with."

Done.

Line 208: is it "foramen" or "foramina"; the figure labels only one foramen. Please clarify.

Thanks for spotting this, it is foramina: clarified and edited in the relevant figure caption (Fig. 3)

Line 211: is there any evidence for the first alveolus? How do you justify the identification of the first preserved tooth as the second tooth in sequence? Clarify.

We reported in the description this explanation, following direct comparison with the condition in both *Acheroraptor* (Evans and Larson, 2013) and *Saurornitholestes* (Currie and Evans, 2020): "Given the position of the most erupted tooth (d3; Fig 3) above the Meckelian foramina (on the medial surface of the dentary, Fig 3B), and above an anteroventral process of the dentary (ave; Figs 3A and 3B), we identify this as the 3rd tooth in the dentary [e.g. 60, 61], with the anteriorly positioned, less erupted tooth (d2) being identified as the 2nd."

Refs:

Currie PJ, Evans DC. Cranial Anatomy of New Specimens of *Saurornitholestes langstoni* (Dinosauria, Theropoda, Dromaeosauridae) from the Dinosaur Park Formation (Campanian) of Alberta. Anat Rec. 2020; doi:10.1002/ar.24241

Evans DC, Larson DW, Currie PJ. 2013. A new dromaeosaurid (Dinosauria: Theropoda) with Asian affinities from the latest Cretaceous of North America. Naturwissenschaften. 100 (11): 1041-1049. doi: 10.1007/s00114-013-1107-5

Line 215: replace "shows" with "has."

Done

Line 216: delete "of…tooth."

Done.

Line 217: replace "dividing" with "separating."

Done

Line 222: rephrase, "slightly anteroposteriorly longer than mediolaterally wide."

Done

Line 224: replace "mid-way depth" with "midheight."

Done

Line 225: replace "a" with "an."

Done

Line 229: replace ", but" with "that is"; the word "but" is used to introduce an exception or contradiction, not a similarity or an elaboration upon a point.

Fixed accordingly

Line 231: replace "mesodistal" with "mesiodistal."

Done

Line 233: replace "damaging" with "damage."

Done

Line 235: insert "13" ahead of "denticles."

Done

Line 236: delete ", with 13 denticles."

Done

Line 239: word choice – does "coarser" mean "larger"? If so, say so to remove the ambiguity.

Changed accordingly with larger.

Line 242: replace "observed" with "seen."

Done

Line 244: replace "a hypothetical" with "an"; how was this estimate arrived at? Briefly justify.

Fixed and added: accounting for the half of the carina being inset in the tooth socket: for clarity.

Line 246: delete "morphological."

Done

Line 253: what, exactly, is rounded and not pointed? I suspect this is a grammatical issue; please fix.

Replaced "rounded" with "with a sharp tip"

Line 263: replace "grains" with "grain."

Done

Line 266. Replace "in" with "along with."

Done

Line 267: replace "points…21183" with "is evidence that DMNH 21183 is a juvenile." Avoid passive voice – in science, it is ok to be declarative! In contrast, passive voice gives the impression that you are backing away from your evidence, which defeats the purpose of your hard work.

Thanks for pointing that out, changed accordingly.

Line 274: delete "traced and."

Done.

Line 294: delete "combination."

Done

Lines 294,295: "oval" and "lenticular" mean the same thing; pick one to avoid redundancy.

Replaced by just lenticular

Lines 300,301: replace "relatively better" with "well."

Done.

Line 310: replace "between" with "among."

Done

Lines 315,316: how is it that you can comment on the form of the first dentary tooth after you've made it clear that it is completely missing? Clarify.

Sentence removed.

Line 324: replace "observed" with "reported."

Done.

Line 328: replace "with" with "have"; replace "compared" with "in contrast." Also, does "coarser" just mean "larger?" Clarify.

All changed accordingly and larger chosen over "coarser".

Line 333: replace "this" with "the"; delete "taxon."

Done

Line 335: what about the ridge?!

Please see comment above.

Line 339: make sure that "anchiornithines" is the correct moniker to use. I.e., is there an "Anchiornithinae"?

Replaced with anchiornithid as Anchiornithidae has been favored by Hu et al. 2018 over the original definition of "Anchiornithinae" by Xu et al. 2016

Refs:

Xu; et al. (2016). "An Updated Review of the Middle-Late Jurassic Yanliao Biota: Chronology, Taphonomy, Paleontology and Paleoecology". Acta Geologica Sinica. 90 (6): 2229–2243. doi:10.1111/1755-6724.13033

Dongyu Hu; Julia A. Clarke; Chad M. Eliason; Rui Qiu; Quanguo Li; Matthew D. Shawkey; Cuilin Zhao; Liliana D'Alba; Jinkai Jiang; Xing Xu (2018). "A bony-crested Jurassic dinosaur with evidence of iridescent plumage highlights complexity in early paravian evolution". Nature Communications. 9 (1): Article number 217.

Line 343: replace "It….that" with "However,"; replace "been shown" with "occurred."

Done

Line 345: pluralize "anchiornithid."

Done

Lines 245 to 346: delete ", in….Eudromaeosauria."

Done

Line 349: replace "reduction in" with "small" since there is no evolutionary or developmental process described here – you really don't know if the size is plesiomorphic or the juvenile condition; avoid process-based terms when describing static, context-free morphology.

Thanks for the tip, and changed accordingly.

Line 353: replace "recalls a similar" with "is similar to the."

Done

Line 354: pluralize "Velociraptorinae."

Done

Line 357: "in-serrated/absent" are equivalent terms – pick one to reduce the annoyance of your reader. Concision is always appreciated in heavy osteodental descriptions!

Changed with unserrated.

Line 358: replace "observed" with "also seen in"; again, avoid passive voice.

Changed.

Line 365: replace "exhibit" with "have."

Done.

Line 387: replace "are" with "include.'

Changed.

Line 392: delete "of the dentary." Avoid redundancy.

Done

Line 394: replace "therefore" with "this criterion was"; delete "as…feature."

Done

Line 395: delete "versus Dromaeosaurinae."

Done

Line 397: briefly define DSDI/

Added "the ratio between number of mesial and distal denticles:

Line 402: is there a citation for the saurornitholestine teeth? What is their value?

Added and referenced relevant figure with DSDI plot

Lines 410-411: move these up to line 402 so that your reader can see how the saurornitholestine values compare right away. By the way, is there a difference between Saurornitholestinae and Velociraptorinae? Is see that Saurornitholestes is a velociraptorine, so Velociraptor is not a saurornitholestine?

Moved and modified also accordingly to some "in text" requests from Reviewer 1

Line 414: what is the DSDI? Give the value!

Added "outliers with DSDI around 2-3" and referenced relevant figure.

Lines 419 to 422: methods are best written in past tense. Please fix.

Fixed

Line 424: replace "provided" with "given."

Done

Lines 434 to 437: fix the commas.

Commas removed and replaced by bracketed explanation

Line 436: replace "provided" with" represented."

Done

Line 438: replace "presented" with "given."

Done

Lines 444 to 446: didn't you earlier state that the lateral ridge allies the specimen with

Buitreraptor? How do you weight the dental data against the osteological evidence? Justify, especially since you are dealing with a juvenile specimen.

As mentioned in the response above, this has now been changed after new observations and reanalysis consequent to phylogenetic scoring.

Line 449: please stop using "status" for "growth stage."

Fixed

Lines 480 to 482: Given that dromaeosaurids are part of a clade of flying theropods, why couldn't have they just flown along their migratory routes? Explain.

While we believe it is un-parsimonious to attribute volant habits to members of Eudromaeosauria, we improved the context of this section of the Discussion, backing up our interpretation that it is unlikely that these theropods nested in southern latitudes, then migrated to northern latitudes in the summer. The occurrence of a young individual of such a small-size itself is evidence that this individual wouldn't have come from so far away as a migration entails, as it would have been dimensionally incapable of covering such large distance (e.g. Fiorillo & Gangloff 2001). The individual would not have at attained the minimum physical maturity necessary for such journey. Similar reasoning has been historically presented for hadrosaurid hatchlings by Horner (1982). While some basal members of Dromaeosauridae might have exhibited some sort of rudimentary (i. e. non-active flight), that might have been at best something close to what has been postulated for members of Microraptorinae (e.g. *Sinornithosaurus* and *Zhenyuanlong* (Xu et al, 1999; Alexander et al. 2010, Dyke et al. 2013), no such arguments have been made for saurornitholestines. Further, the anatomy of most dromaeosaurids, especially larger species, argues for a flightless lifestyle (Turner et al. 2007). That eliminates flight as an energy efficient means for these small predatory animals to cover the vast distances required to migrate across large wide geographic areas. Lastly, in response to a comment by one of the reviewers about the possibility of the young being volant, we point out that it is unlikely that the young forms migrated and the adult forms did not as there is no modern analog for such a model. We briefly discuss that within modern birds, the adults teach their young the necessary tools for migration (e.g. Baker, 1980).

Ref:

Horner JR. Evidence of colonial nesting and 'site fidelity' among ornithischian dinosaurs.

Nature. 1982; 297(5868):675-676.

Dyke G, de Kat R, Palmer C, van der Kindere J, Naish D, Ganapathisubramani, B. Aerodynamic performance of the feathered dinosaur *Microraptor* and the evolution of feathered flight. Nature Communications. 2013; 4 (2489). doi:10.1038/ncomms3489

Alexander, D. E., Gong, E., Martin, L. D., Burnham, D. A. & Falk, A. R. Model tests of gliding with different hindwing configurations in the four-winged dromaeosaurid *Microraptor gui*. Proceedings of the National Academy of Science. 2010; 107, 2972-2976.

Xu X, Wang X-L, Wu X-C. A dromaeosaurid dinosaur with filamentous integument from the Yixian Formation of China. Nature. 1999; 401:262–266.

Turner AH, Pol D, Clarke J, Erickson G, Norell M. A basal dromaeosaurid and size evolution preceding avian flight. Science. 2007; 317:1378–1381.

Line 488: replace "southerner" with "southern."

Done

Lines 485 to 488: why is large size evidence for "success"? Justify. What is meant by "success"? Clarify.

We removed this section as we in the end agreed with the consideration that most of this reasoning was too speculative and outside the scope of the study.

Line 493: change "stronger constrained" to "greater constraints." What sort of constraints? Clarify.

Same as above

Lines 494 to 495: convoluted; fix.

Fixed.

Line 498: "eggs" should be singular.

Fixed

Lines 494 to 505: perhaps it is because it is late in the day, but I find this section to be very

difficult to follow. Please redraft with a clearer argument and straightforward sentence structure.

Thanks for the suggestion. We redrafted this section to simplify the reasoning and expunge the speculative content as recommended by the reviewer.

Line 506: avoid passive voice; fix.

Fixed.

Lines 510 to 512: this can be shortened significantly; fix.

Done

Lines 513 to 515: the mere presence of taxon does not imply migration any more than it doesn't imply migration. Fix.

Done

Line 523: should there be an "r" in "cfr"? Isn't it just "cf."? Where's the period? Fix.

Fixed

Line 525: replace "appear" with "are."

Fixed.

Line 526: replace "but" with "and" since you aren't marking a difference or exception.

Fixed

Line 544: a period should follow "al"; e.g., Osborn et al. (1905); replace "for" with "that."

Done

Line 545: insert "is" after the taxon name.

Added

Lines 513 to 555: I find this section to be, uncomfortably, speculative, but I leave it up to the judgment of the author and co-authors whether or not it needs to be reined in. Perhaps more citations in this section would put any concerns to rest. It just strikes me as a bridge too far when all of it is merely based on the relative abundance of teeth and – as far as I can tell - inconclusive functional inferences of tooth morphology. Please reconsider this section in a more critical light.

See comments above.

Lines 560 to 562: You don't really know that it wasn't migratory; it could be that all small dromaeosaurids were fully capable of flight early in growth. Please reconsider this point more critically – does the inference really have support?

See comments above.

Lines 564 to 565: again, you have no rationale to think this – if dromaeosaurids were volant, there's literally nothing to stop them from migrating. It's possible flight brought deinonychosaurians their global distribution. Regardless, I don't think you have the evidence to make the claim, unless there's something obvious that I missed earlier.

See comments above.

Figure 3: "a3" is not labeled; label both interdental plates; label the circular fossa.

Done.
